# Chiral Imidazo[1,5‑*a*]pyridine-Based Ligands for the Au-Catalyzed Enantioselective Intramolecular Hydrocarboxylation of Allenes

**DOI:** 10.1021/jacsau.5c00885

**Published:** 2025-09-11

**Authors:** Chloé Stoll, Céline Besnard, Clément Mazet

**Affiliations:** † Department of Organic Chemistry, 27212 University of Geneva, 30 quai Ernest Ansermet, 1211 Geneva, Switzerland; ‡ Laboratory of Crystallography, 27212 University of Geneva, 24 quai Ernest Ansermet, 1211 Geneva, Switzerland

**Keywords:** Privileged ligands, chiral ImPy ligands, gold catalysis, enantioselective catalysis, hydrocarboxylation, allenes

## Abstract

We report the synthesis of gold­(I) complexes supported by imidazo­[1,5-*a*]­pyridine-based ligands featuring chiral aniline moieties at N(2). The synthesis is short, efficient, and modular, allowing for precise control over the steric and electronic properties of the coordination sphere of the metal. The design of these atypical chiral ligands was validated in the Au­(I)-catalyzed enantioselective intramolecular hydrocarboxylation of allenes, which furnished tricyclic N(1)–C(2)-fused oxazino-indolones in high yield and unprecedented levels of enantiocontrol.

## Graphical abstract

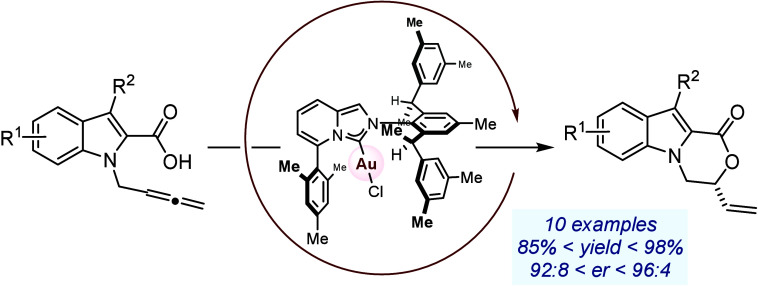

The notion of ‘privileged ligand’ was introduced in the field of asymmetric catalysis by Yoon and Jacobsen in 2003.^1,2^ While this concept applies to chiral ligands that afford high levels of enantioselectivity for a variety of reactions that are mechanistically unrelated, there are certainly several achiral ligands that could be termed ‘privileged’ in the context of nonselective catalysis for their ability to provide excellent reactivity across a broad range of catalytic transformations operating by distinct mechanisms. For instance, the dialkylbiaryl phosphines—commonly termed Buchwald ligands (**BL**)—have found widespread applications in homogeneous catalysis using a diversity of transition metals across the *d*-block of the periodic table (Figure 1-A, left). Over the years, important design elements of these ligands have been identified both empirically and theoretically.^3–5^ Notably, the flexibility offered in adjusting the steric and electronic properties of the ligand permits reconciling the seemingly antagonistic requirements facilitating oxidative addition and reductive elimination, two fundamental elementary steps in cross-coupling reactions.^6^ Another distinct features of the Buchwald ligands is their propensity to act as pseudo-bidentate ligands via π interactions between the metal and the ‘lower’ aryl ring, with coordination modes varying from η^1^ to η^6^.^3–8^ Moreover, the orientation of this arene unit below the coordination sites where the reactions occur has been proposed to stabilize reactive intermediates during catalysis. *N*-heterocyclic carbenes (**NHC**) have established themselves as another versatile ligand class for use in (non-stereoselective) transition metal catalysis (Figure 1-A, right).^9–11^ Their pronounced σ donor character leads to stronger metal–ligand bonds compared to phosphine ligands and, consequently, to enhanced catalytic activity. Particularly attractive is the ease of tuning of the steric environment of NHCs, which in contrast to cone-shaped P-ligands, is projected toward the coordination sphere of the transition metal and enables better control on the reactive sites. The synthesis of chiral NHCs is well-established, usually short and modular, and an impressive number of their successful use in enantioselective catalysis have been reported.^10–12^ The preparation of chiral dialkylbiaryl phosphines is comparatively less trivial and only a handful of examples have been described to date.^13–16^ Recent years have witnessed the development of transition metal complexes supported by imidazo­[1,5-*a*]­pyridine–based ligands (**ImPy**) (Figure 1-A, middle).^17–20^ Formally, this scaffold can be perceived as a merger of Buchwald ligands and *N*-heterocyclic carbenes, which should ideally retain their best attributes (strong σ donor ability, modular steric environment, and presence of a ‘lower’ aryl ring for π interactions with the metal). Moreover, their ease of synthesis and the possibility to vary independently the nature of the substituents at C(5) and N(2) constitute additional attractive features of these ligands.

**Figure 1:**
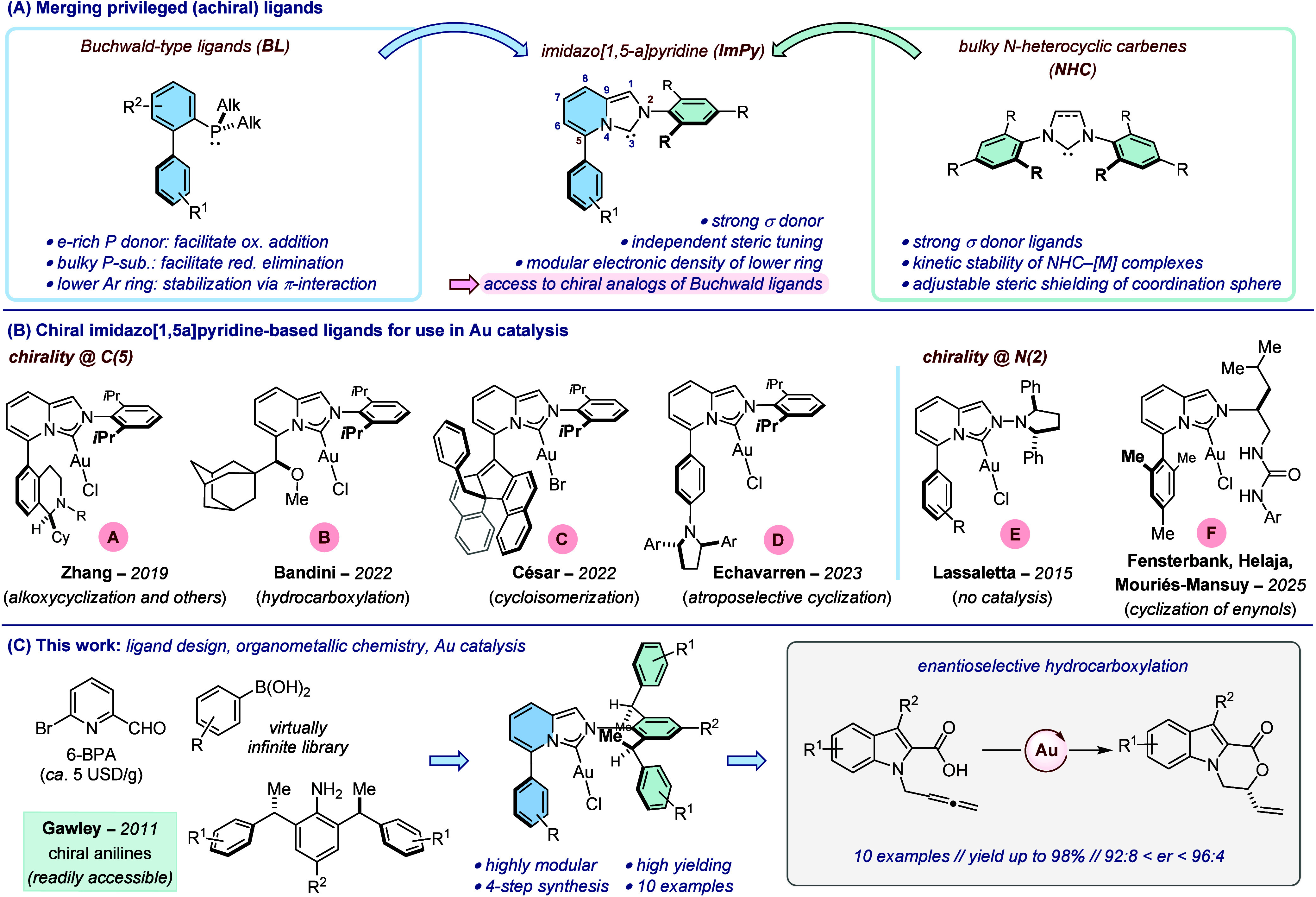
(A) Merging privileged achiral ligand. (B) Examples of Au­(I) complexes supported by chiral ImPy ligands. (C) This work: design of novel chiral @ N(2) ImPy ligands and application in the enantioselective Au­(I)-catalyzed hydrocarboxylation of allenes.

Following pioneering studies from the Echavarren laboratory, cumulated efforts from several research groups have established the privileged character of Buchwald-type ligands in gold catalysis.^21–26^ Their ability to stabilize neutral and cationic linear gold π-complexes as well as carbene and/or cationic intermediates provides a distinct advantage over other ligand classes. The principal challenge in enantioselective gold catalysis is to exert stereocontrol at a reactive site that is *trans* to the donor atom of the chiral ancillary ligand. Consequently, due to the structural resemblances between dialkylbiaryl phosphines and imidazo­[1,5-*a*]­pyridine-based ligands, several chiral gold complexes have been designed around the scaffold of the latter (and applied in mechanistically unrelated catalytic reactions) (Figure 1-B). For instance, the Zhang group reported gold­(I) complexes (**A**) supported by bifunctional ImPy ligands displaying both axial and central chirality, which gave promising levels of enantioselectivity in an array of Au-catalyzed processes.^27^ The chiral ligands developed by Bandini and co-workers featured a sterically demanding secondary alkyl ether moiety in close proximity of the metal center (**B**). These were applied in an original Au-catalyzed hydrocarboxylation of allenes with enantiomeric ratio up to 81:19.^28^ The high enantioinduction obtained by Crassous, Bastin, César and co-workers in a benchmark cycloisomerization of *N*-tethered 1,6-enynes served to validate the design of gold complexes, which combined central, axial and helical chirality (**C**).^29^ As part of a study focused on the synthesis and evaluation of gold­(I) complexes supported by chiral dialkylbiaryl phosphines, the Echavarren group reported the synthesis of imidazo­[1,5-*a*]­pyridine-based ligands where *C*_2_-symmetric 2,5-diarylpyrrolidine units had been directly attached to the lower aryl ring to create a chiral environment in close proximity to the reactive sites (**D**). The ability of these systems to impart high levels of enantioinduction was established in a Au-catalyzed intramolecular cyclization of 1,6-arylenynes.^30^ Quite notably, in all these structures, the chirality element(s) have been introduced at the C(5) position of the imidazolium core, keeping sterically demanding aryl rings at N(2). To the best of our knowledge, examples of chiral [(ImPy)­Au] complexes where the stereogenic element has been connected to N(2) (such as in complexes **E** and **F**) and that have been evaluated in asymmetric catalysis remain very rare (**F**, Figure 1-B).^31–34^

We report herein the synthesis of gold­(I) complexes supported by imidazo­[1,5-*a*]­pyridine-based ligands incorporating chiral aniline moieties at N(2) and their application in the Au-catalyzed enantioselective hydrocarboxylation of allenes originally developed by Bandini (Figure 1-C).^28^

Starting from cheap and commercially available 6-bromopyridine-2-carboxaldehyde (BPA, **1**) as central building block, the synthesis of 10 imidazo­[1,5-*a*]­pyridine ligand precursors was accomplished via a 3-step linear sequence (Figure 2). Two complementary sets of conditions were identified to introduce a variety of aryl substituents at C(5) by means of Pd-catalyzed Suzuki cross-coupling reactions between the appropriate boronic acid and **1**, with yields ranging from 40% to 79%. Subsequent Schiff-base condensations were achieved in high yield using procedures adapted from the literature and a subset of chiral *C*_2_-symmetric 2,6-di­(1-arylethyl)­aniline derivatives (**3**)–the synthesis of which was originally disclosed by Gawley and co-workers and generalized by the Shi and Cramer groups.^35–41^ The imino-pyridines thus obtained (**4**) were engaged in a final cyclization using chlorotrimethylsilane and paraformaldehyde in toluene at room temperature to afford the targeted imidazolium salts (**5**) as off-white crystalline solids in good to quantitative yields. Complexation experiments were conducted by reacting the imidazolium salts with AuCl•SMe_2_ in THF at room temperature followed by addition of 2.0 equivalents of of K_2_CO_3_ to yield, after 48 h, the neutral and air-stable gold complexes (**6**).^34^ High quality crystals for single-crystal X-ray diffraction analyses were obtained for complexes (*R*,*R*)-**6c**, (*R*,*R*)-**6e** and (*R*,*R*)-**6f** (Figure 3). The principal structural parameters of all three complexes are reminiscent to those observed in neutral gold complexes supported by either Buchwald or ImPy ligands.^28–30,32,38^ Specifically, they display a (i) slightly distorted linear coordination geometry around the metal center with the chlorine atom bent away from the ‘lower’ aryl ring, (ii) characteristic metal–carbon and metal–chloride bond lengths, and (iii) they exhibit similar Au–C_*ipso*_ distances–a feature commonly analyzed in transition metal complexes bearing Buchwald ligands to gauge the potential influence of the ‘lower’ aryl ring on reactivity. A buried volume (%V_*bur*_) of 55.3% was calculated for complex (*R*,*R*)-**6c**. Smaller values by ca. 9% were obtained for complexes (*R*,*R*)-**6e** and (*R*,*R*)-**6f** (51.6% and 51.5% respectively).^42–48^

**Figure 2:**
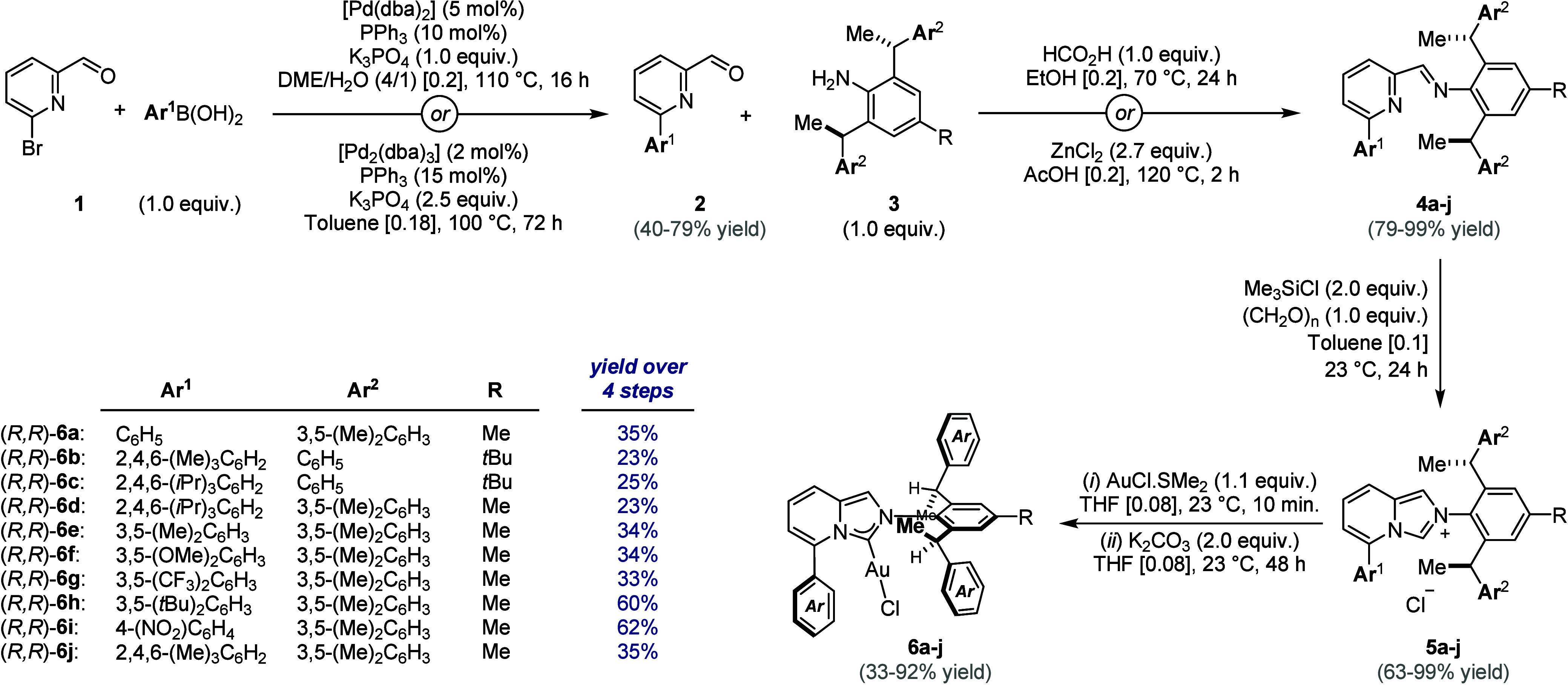
Three-step synthetic sequence leading to imidazolium salts **5a**–**j** (0.15–0.60 mmol) and synthesis of the corresponding neutral Au­(I) complexes (*R*,*R*)-**6a**–**j** (0.05–0.10 mmol).

**Figure 3:**
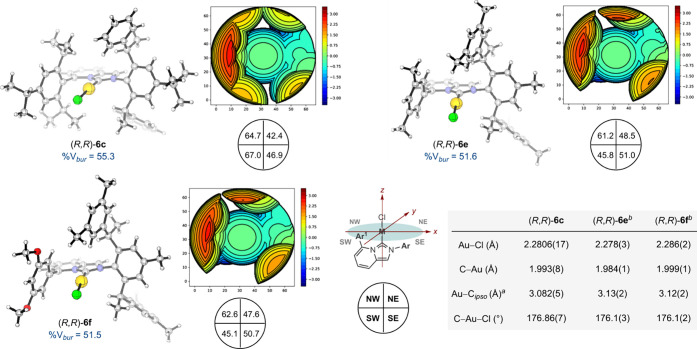
X-ray analyses and selected structural parameters for complexes (*R*,*R*)-**6c**, (*R*,*R*)-**6e**, and (*R*,*R*)-**6f**. Steric maps for buried volume (%V_*bur*_) are viewed along the *z* axis. Atomic radii: bondi radii scaled by 1.17. Sphere radius: 3.5 Å. Distance of the coordination point from the center of the sphere: 0.0. Mesh spacing: 0.01. Quadrant values in %. NW: North West; NE: North East; SW: South West; SE: South East. ^*a*^C_*ipso*_: *ipso* carbon atom of the ‘lower’ aryl ring (Ar^1^). ^*b*^Average values of the 2 non-equivalent molecules per unit cell.

The ten neutral gold complexes were subsequently evaluated in the Au-catalyzed enantioselective intramolecular hydrocarboxylation of allenes recently disclosed by Monari, Ollevier, Bandini and co-workers (Figure 4).^28^ It must be underscored that our initial survey began with the four precatalysts (*R*,*R*)-**6a**–**d** and that all subsequent structures were designed semi-empirically on the basis of the initial results obtained. Using **7a** as model substrate and the optimized protocol reported in the literature, precatalysts (*R*,*R*)-**6a** and (*R*,*R*)-**6b** generated little or no product–the latter inducing only a very modest level of enantiocontrol (60:40 *er*). Noticeably, complex (*R*,*R*)-**6c**, which has the same aniline moiety (Ar^2^) but a more demanding 2,4,6-tris-*iso*-propyl aryl ring (Ar^1^), significantly increased reactivity while inducing the same level of selectivity. With (*R*,*R*)-**6d**–which shares the same substituent at C(5) but possesses a bulkier aniline unit–the reactivity was maintained and the enantiomeric ratio markedly improved (93:7 *er*). At this stage of our investigations, it was decided to keep the aniline fragment constant and to vary the steric and electronic properties of the aryl ring at C(5), readily installed by a Pd-catalyzed Suzuki cross-coupling reaction in the first step of the ligand synthesis (*vide supra*). We found that the presence of substituents in the two *meta* positions of Ar^1^ imparted systematically high enantiocontrol (92:8 < *er* < 94.5:5.5). Reactivity remained excellent with methyl and methoxy groups but diminished with the more demanding trifluoromethyl and *tert*-butyl substituents (compare (*R*,*R*)-**6e**–**h**). Whereas the presence of an electron-withdrawing group in the *para* position led to excellent yield of the oxazino-indolone (**8a**) and a 92:8 *er* ((*R*,*R*)-**6i**), the best balance between reactivity and selectivity was obtained when installing a mesityl unit at C(5) ((*R*,*R*)-**6j**: 81% yield, 96:4 *er*). The enantiomeric ratio increased to 97:3 *er* by performing the reaction at 0 °C. Further variations of the solvent, the time and evaluation of other silver salts did not improve this result (see SI for details).^49^

**Figure 4:**
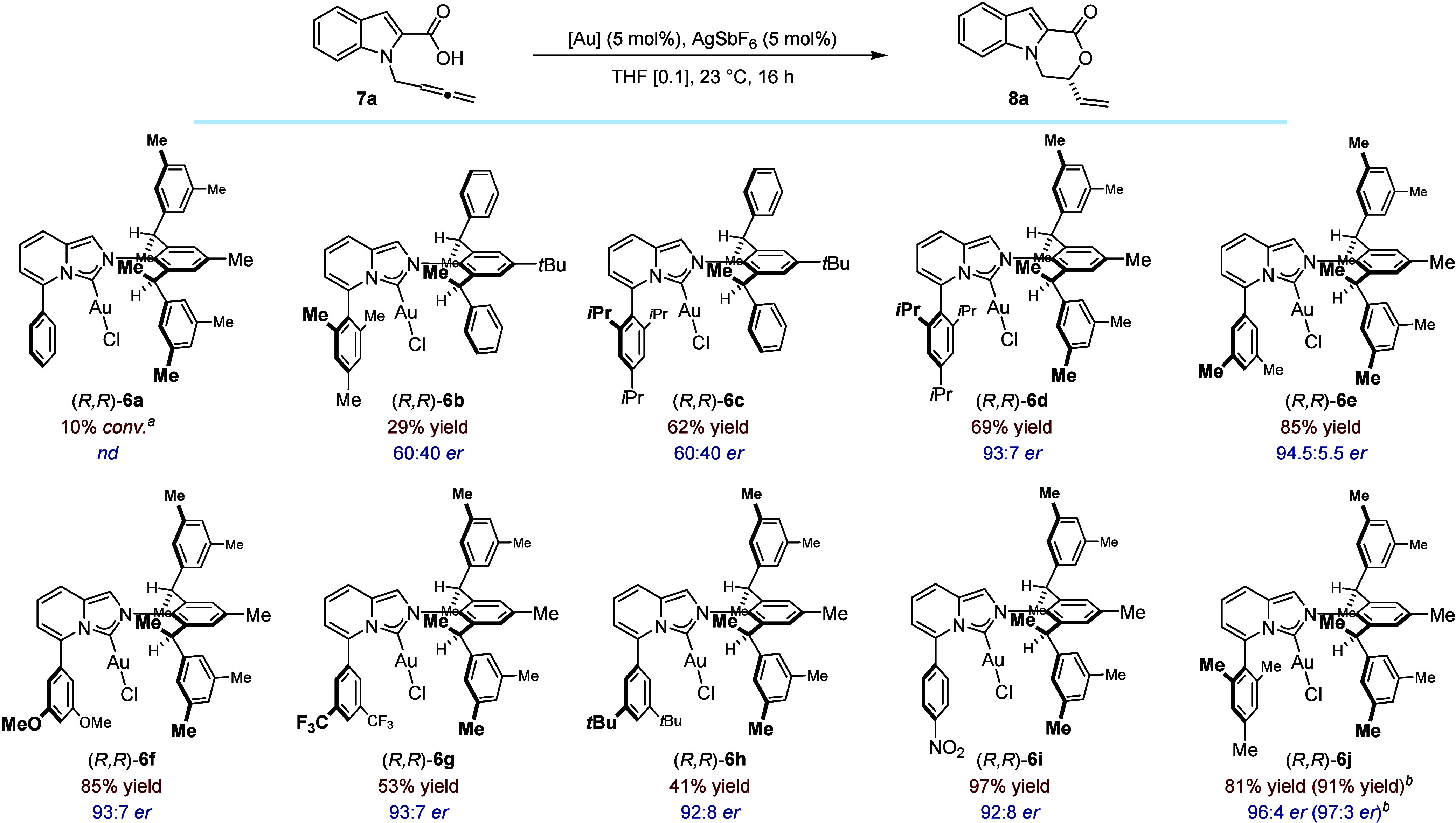
Catalyst survey of the Au­(I)-catalyzed enantioselective hydrocarboxylation of allenes (0.15 mmol scale). Yield of isolated product after purification. Enantiomeric ratio determined by HPLC using a chiral stationary phase. ^*a*^Determined by ^1^H NMR against an internal standard. ^*b*^In parentheses, reaction run at 0 °C.

We noticed that the steric map obtained for (*R*,*R*)-**6c** shows very little difference between the crowed NW and SW quadrants (64.7%; 67.0%) as well as between the more open NE and SE quadrants (42.4%; 46.9%). This precatalyst induces a modest enantiomeric ratio (60:40 *er*). In contrast, complexes (*R*,*R*)-**6e** and (*R*,*R*)-**6f** display similar steric maps with both a more pronounced level of asymmetry between all four quadrants. Even though based on a ground-state analysis (i.e., crystal structures), it is tempting to attribute–at least partially–the higher level of enantioinduction imparted by **6e** and **6f** to this structural difference.

The generality of the enantioselective Au­(I)-catalyzed hydrocarboxylation reaction was delineated by subjecting a variety of *N*-allenyl-indole-2-carboxylic acids to the optimized conditions using precatalyst (*R*,*R*)-**6j** (Figure 5). While the reactivity was systematically very high, more remarkably perhaps, excellent levels of enantiomeric ratio were obtained independently of the position and nature of the substitution of the indole core of the substrate. For instance, introduction of methoxy or methyl substituents in the 5-position (**8b**, **8d**) or 4-position (**8c**) gave the desired fused oxazino-indolone in nearly quantitative yield and with consistently high selectivity. Similarly, installing a methyl group at the more demanding C(3) or C(7) positions did not affect the catalytic performance significantly (**8e**, **8f**). We found that diverse halides (F, Cl and Br) were compatible with the optimized reaction conditions, leading to the cyclized products in high yield and enantiomeric ratio (**8g**-**8j**). Finally, the absolute configuration of **8j** was determined by X-ray crystallographic analysis, and that of all other products of catalysis was deduced by analogy.

**Figure 5:**
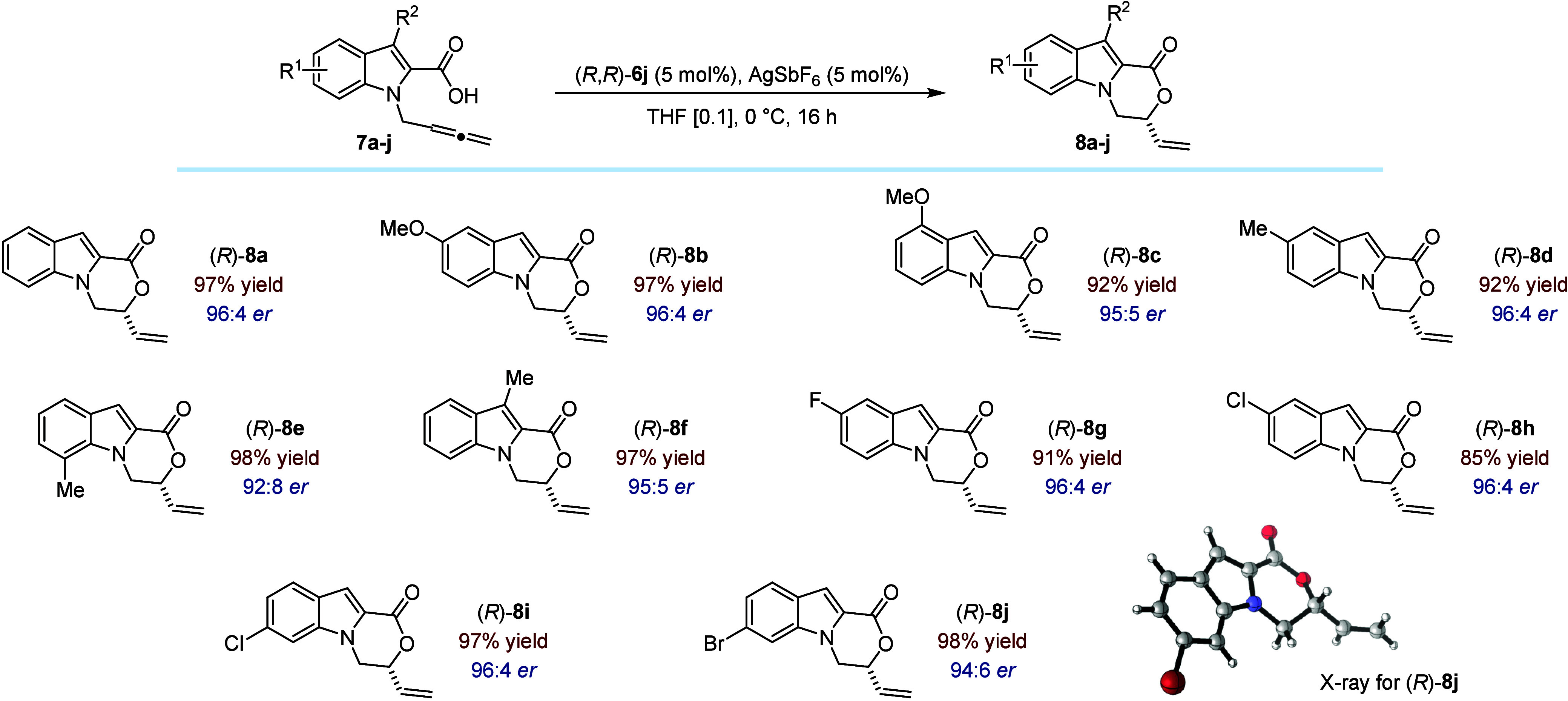
Scope of the Au­(I)-catalyzed enantioselective hydrocarboxylation of allenes (0.30 mmol scale). Yield of isolated product after purification. Enantiomeric ratio determined by HPLC using a chiral stationary phase.

In summary, we have prepared a collection of chiral imidazo­[1,5-*a*]­pyridine-based ligands incorporating chiral aniline moieties at N(2) as well as the corresponding neutral gold­(I) complexes. The design of this original scaffold has been validated in the Au-catalyzed enantioselective hydrocarboxylation of allenes. The highly modular nature of the synthetic route enabled to readily obtain unprecedented levels of enantiomeric ratio for this most challenging transformation. Current efforts in our group are directed at identifying other selective metal-based transformations where these ligands would be uniquely effective.

## Supplementary Material



## Notes

The Supporting Information is available free of charge on the ACS Publications Web site. (PDF). The Supporting Information is available free of charge at https://pubs.acs.org/doi/10.1021/jacsau.5c00885.

Experimental procedures, characterization of all new compounds, spectroscopic data and X-ray crystallographic data (PDF)
