# Supplementary material for: Chiral Imidazo[1,5‑a]pyridine-Based Ligands for the Au-Catalyzed Enantioselective Intramolecular Hydrocarboxylation of Allenes
Source: JACS Au. 2025 Sep 11;5(10):4681–7. doi: 10.1021/jacsau.5c00885 (PMC12569711; doi:10.1021/jacsau.5c00885)

Chiral Imidazo[1,5-*a*]pyridine-based Ligands for the Au-Catalyzed Enantioselective Intramolecular Hydrocarboxylation of Allenes

Chloé Stoll,† Céline Besnard,‡ Clément Mazet†*

† Department of Organic Chemistry, University of Geneva, 30 quai Ernest Ansermet, 1211 Geneva, Switzerland.

‡ Laboratory of Crystallography, University of Geneva, 24 quai Ernest Ansermet, 1211 Geneva, Switzerland.

clement.mazet@unige.ch

Table of Contents

1. General information 3

2. Suzuki coupling (2a-2h) 4

3. Preparation of imines ((*R*,*R*)-4a-(*R*,*R*)-4j) 8

4. Preparation of imidazolium salts ((*R*,*R*)-5a-(*R*,*R*)-5j) 17

5. Complexation to Au(I) ((*R*,*R*)-6a-(*R*,*R*)-6j) 26

6. Substrate synthesis 36

7. Optimization of the Au(I)-catalyzed enantioselective hydrocarboxylation of allenes 39

8. Scope of the Au(I)-catalyzed enantioselective hydrocarboxylation of allenes 43

10. X-Ray Analyses 55

11. Supplementary experiments 63

12. References 66

13. NMR of new compounds 68

1. General information

Unless otherwise noted, all reactions were carried out under an inert atmosphere of nitrogen using either a two-manifold vacuum/inert gas lines or a M.Braun glove-box. Solvents were dried over activated alumina columns and further degassed by three successive "freeze-pump-thaw" cycles. Commercial reagents were purchased from Fluorochem, Fluka, ABCR, Acros, or Strem and used without purification unless otherwise noted Liquid reagents were transferred with stainless steel syringes or cannula. Thin layer chromatography (TLC) was performed on plates of silica pre-coated with 0.25 mm Kieselgel 60 F_{254} from Merck. Flash chromatography was performed using silica gel SiliaFlash® P60 (230-400 mesh) from Silicycle.

NMR spectra were acquired at the University of Geneva NMR platform (https://www.unige.ch/sciences/chiorg/nmr/) using a 500 MHz Avance III Bruker NMR spectrometer equipped with a helium-cooled cryogenic 5-mm DCH ^{13}C-^{1}H/D Bruker probe, a 400 MHz Avance III HD NanoBay spectrometer equipped with a N_2 prodigy cryogenic 5 mm CPP BB(F)-H-D probe or a 300 MHz Avance III, HD NanoBay spectrometer, equipped with a 5 mm PA BBO, BB(F)-H-D probe. ^{1}H NMR spectra were referenced to $CDCl_3$ (7.26 ppm) and $^{13}C\{^{1}H\}$ NMR spectra were referenced to $CDCl_3$ (77.16 ppm). $^{19}F\{^{1}H\}$ NMR chemical shifts are reported in ppm with absolute reference relative to ^{1}H. LRMS data were obtained at the mass spectrometry facility of the University of Geneva (http://www.unige.ch/sciences/sms/). HRMS data were obtained on a Xevo G2 TOF spectrometer (ionization mode: ESI positive polarity; mobile phase: MeOH 100 µL/min). Mass spectrum is calibrated using the MS lockspray system (LeuEnk calibration solution). Infrared spectra were obtained on a Perkin–Elmer 1650 FT-IR spectrometer using neat samples on a diamond ATR Golden Gate sampler. Melting points were recorded on a Büchi SMP-20 melting point apparatus using open glass capillaries.

The enantiomeric ratio (*er*) were determined by HPLC analyses. HPLC analyses were performed on a Shimadzu CTO-20AA equipped with DAICEL OD-H, OZ-H, OJ-H, AD-H and IC columns. Retention times (t_R) are given in minutes. Optical rotations were recorded using an OMNI Lab JASCO P-1030 polarimeter or a Perkin Elmer 241 Polarimeter using 589 nm emission band of a sodium lamp or 346 and 475 nm emission band of a mercury lamp.

Starting materials **3a**[1–3], **3b**[2,4], **7a-d**[11] and **7f-j**[11] were synthetized according to literature procedures.

2. Suzuki coupling (2a-2h)

General procedure I (GP I)

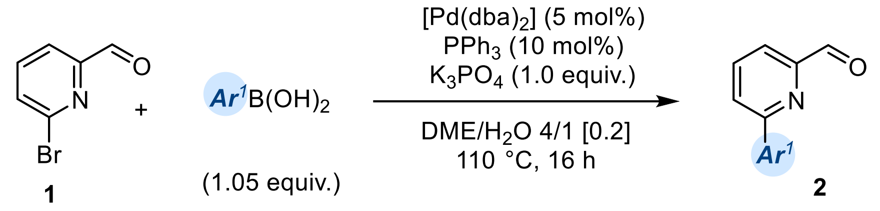

The protocol was adapted from a literature procedure.[5] In a glovebox, a J-Young Schlenk tube was charged with [Pd(dba)$_2$] (58 mg, 100 µmol, 5 mol%), PPh_3 (53 mg, 200 µmol, 10 mol%) and 1,2-DME (0.2 M). After stirring for 10 min., 6-bromo-2-pyridinecarboxaldehyde (372 mg, 2.0 mmol, 1.0 equiv.) was added and the J-Young Schlenk tube was sealed and taken out of the glovebox. After stirring for 30 min. at 23 °C, a solution of K_3PO_4 (446 mg, 2.1 mmol, 1.05 equiv.) in degassed water (0.2 M) was added, directly followed by the appropriate boronic acid (1.05 equiv.) and the tube was placed in a pre-heated oil bath (110 °C). After 16 h, the reaction was cooled to 23 °C and quenched with water (10 mL). The aqueous phase was extracted with CH_2Cl_2 (3 × 10 mL). The organic layers were combined, washed with brine (40 mL) and dried over anhydrous Na_2SO_4. The mixture was filtered and concentrated under reduced pressure. Purification by flash chromatography afforded the desired compound.

General procedure II (GP I)

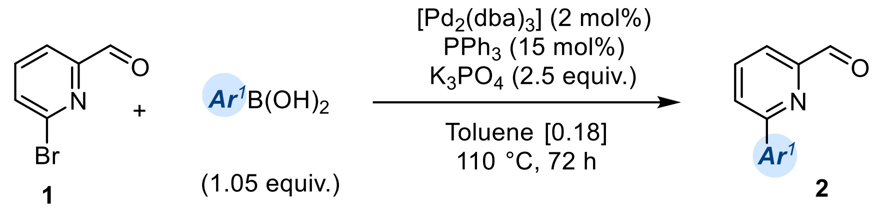

The protocol was adapted from a literature procedure.[6] In a glovebox, a J-Young Schlenk tube was charged with [Pd$_2$(dba)$_3$] (37 mg, 40 µmol, 2 mol%), PPh_3 (79 mg, 300 µmol, 15 mol%), 6-bromo-2-pyridinecarboxaldehyde (372 mg, 2.0 mmol, 1.0 equiv.) and toluene (0.18 M). Next, K_3PO_4 (446 mg, 2.1 mmol, 1.05 equiv.) and the appropriate boronic acid (1.05 equiv.) were added, the J-Young Schlenk tube was sealed, taken out of the glovebox and placed in a pre-heated oil bath (110 °C). After 72 h, the reaction was cooled to 23 °C and quenched with water (10 mL). The aqueous phase was extracted with CH_2Cl_2 (3 × 10 mL). The organic layers were combined, washed with brine (40 mL) and dried over anhydrous Na_2SO_4. The mixture was filtered and concentrated under reduced pressure. Purification by flash chromatography afforded the desired compound.

6-phenylpicolinaldehyde (2a)

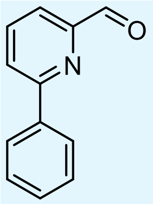

Following GP I, using [Pd(dba$_2$)] (58 mg, 100 µmol, 5 mol%), PPh_3 (53 mg, 200 µmol, 10 mol%), K_3PO_4 (446 mg, 2.1 mmol, 1.05 equiv.), 6-bromo-2-pyridinecarboxaldehyde (372 mg, 2.0 mmol, 1.0 equiv.) and phenyl boronic acid (256 mg, 2.1 mmol, 1.05 equiv.). After flash chromatography (pentane/Et_2O, 9:1), compound **2a** was obtained in pure form as a white solid (259 mg, 1.4 mmol, 71% yield). The spectral data were in agreement with those reported in the literature.[7]

6-(3,5-dimethylphenyl)picolinaldehyde (2b)

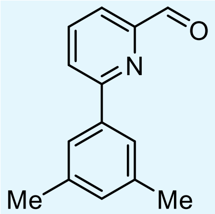

Following GP I, using [Pd(dba$_2$)] (58 mg, 100 µmol, 5 mol%), PPh_3 (53 mg, 200 µmol, 10 mol%), K_3PO_4 (446 mg, 2.1 mmol, 1.05 equiv.), 6-bromo-2-pyridinecarboxaldehyde (372 mg, 2.0 mmol, 1.0 equiv.) and 3,5-dimethylphenyl boronic acid (315 mg, 2.1 mmol, 1.05 equiv.). After flash chromatography (pentane/Et_2O, 9:1), compound **2b** was obtained in pure form as a white solid (317 mg, 1.5 mmol, 75% yield). The spectral data were in agreement with those reported in the literature.[8]

6-(3,5-dimethoxyphenyl)picolinaldehyde (2c)

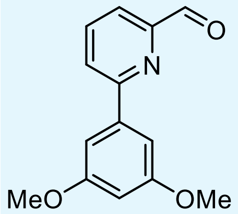

Following GP I, using [Pd(dba$_2$)] (58 mg, 100 µmol, 5 mol%), PPh_3 (53 mg, 200 µmol, 10 mol%), K_3PO_4 (446 mg, 2.1 mmol, 1.05 equiv.), 6-bromo-2-pyridinecarboxaldehyde (372 mg, 2.0 mmol, 1.0 equiv.) and 3,5-dimethoxyphenyl boronic acid (382 mg, 2.1 mmol, 1.05 equiv.). After flash chromatography (pentane/Et_2O, 95:5), compound **2c** was obtained in pure form as a white solid (349 mg, 1.4 mmol, 72% yield). The spectral data were in agreement with those reported in the literature.[7]

6-(3,5-bis(trifluoromethyl)phenyl)picolinaldehyde (2d)

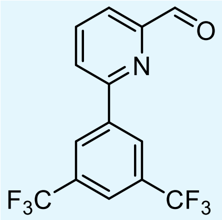

Following GP I, using [Pd(dba$_2$)] (58 mg, 100 µmol, 5 mol%), PPh_3 (53 mg, 200 µmol, 10 mol%), K_3PO_4 (446 mg, 2.1 mmol, 1.05 equiv.), 6-bromo-2-pyridinecarboxaldehyde (372 mg, 2.0 mmol, 1.0 equiv.) and 3,5-bis(trifluoromethyl)phenyl boronic acid (542 mg, 2.1 mmol, 1.05 equiv.). After flash chromatography (pentane/Et_2O, 9:1), compound **2d** was

obtained in pure form as a white solid (482 mg, 1.5 mmol, 76% yield). The spectral data were in agreement with those reported in the literature.[7]

6-(3,5-di-tert-butylphenyl)picolinaldehyde (2e)

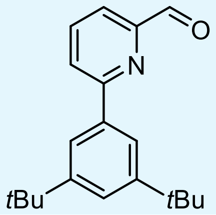

Following GP II, using [$Pd_2(dba_3)$] (37 mg, 40 μmol, 2 mol%), PPh_3 (79 mg, 300 μmol, 15 mol%), K_3PO_4 (1.06 g, 5.0 mmol, 2.5 equiv.), 6-bromo-2-pyridinecarboxaldehyde (372 mg, 2.0 mmol, 1.0 equiv.) and 3,5-di-tert-butylphenyl boronic acid (492 mg, 2.1 mmol, 1.05 equiv.). After flash chromatography (pentane/Et_2O, 95:5), compound **2e** was obtained in pure form as a white solid (469 mg, 1.6 mmol, 79% yield).

^{1}H NMR (400 MHz, 298 K, $CDCl_3$) δ (ppm) = 10.20 (d, $^4J_{HH}$ = 0.5 Hz, 1H), 7.96 – 7.90 (m, 3H), 7.90 (d, $^4J_{HH}$ = 1.8 Hz, 2H), 7.57 (t, $^4J_{HH}$ = 1.8 Hz, 1H), 1.42 (s, 18H).

^{13}C{^{1}H} NMR (101 MHz, 298 K, $CDCl_3$) δ (ppm) =194.3 (CH), 159.2 (C_4), 152.7 (C_4), 151.5 (2 × C_4), 137.7 (C_4), 137.6 (CH), 124.9 (CH), 123.9 (CH), 121.5 (2 × CH), 119.4 (CH), 35.1 (2 X C, C), 31.5 (2 × CH_3).

HRMS (ESI+) *m/z*: calculated for $C_{20}H_{26}NO$ $[M+H]^+$: 296.2009; found: 296.2006.

IR (neat) ν (cm^{-1}): 2960, 2866, 1715, 1584, 1475, 1363, 1249, 883, 812, 714.

mp: 150 °C.

6-(2,4,6-triisopropylphenyl)picolinaldehyde (2f)

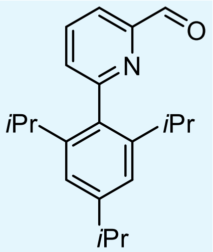

Following GP II, using [$Pd_2(dba_3)$] (37 mg, 40 μmol, 2 mol%), PPh_3 (79 mg, 300 μmol, 15 mol%), K_3PO_4 (1.06 g, 5.0 mmol, 2.5 equiv.), 6-bromo-2-pyridinecarboxaldehyde (372 mg, 2.0 mmol, 1.0 equiv.) and 2,4,6-triisopropylphenyl boronic acid (521 mg, 2.1 mmol, 1.05 equiv.). After flash chromatography (pentane/Et_2O, 95:5), compound **2f** was obtained in pure form as a white solid (246 mg, 0.8 mmol, 40% yield). The spectral data were in agreement with those reported in the literature.[6]

6-mesitylpicolinaldehyde (2g)

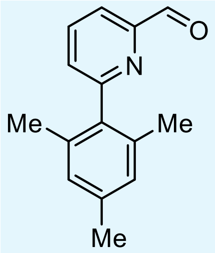

Following GP II, using [$Pd_2(dba_3)$] (37 mg, 40 μmol, 2 mol%), PPh_3 (79 mg, 300 μmol, 15 mol%), K_3PO_4 (1.06 g, 5.0 mmol, 2.5 equiv.), 6-bromo-2-pyridinecarboxaldehyde (372 mg, 2.0 mmol, 1.0 equiv.) and 2,4,6-trimethylphenyl boronic acid (344 mg, 2.1 mmol, 1.05 equiv.). After flash chromatography (pentane/Et_2O, 95:5), compound **2g** was obtained in pure form as a white solid (312 mg, 1.4 mmol, 69% yield). The spectral data were in agreement with those reported in the literature.[9]

6-(4-nitrophenyl)picolinaldehyde (2h)

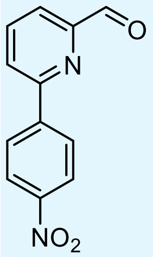

Following GP I, using [$Pd(dba_2)$] (58 mg, 100 μmol, 5 mol%), PPh_3 (53 mg, 200 μmol, 10 mol%), K_3PO_4 (446 mg, 2.1 mmol, 1.05 equiv.), 6-bromo-2-pyridinecarboxaldehyde (372 mg, 2.0 mmol, 1.0 equiv.) and 4-nitrophenyl boronic acid (351 mg, 2.1 mmol, 1.05 equiv.). After flash chromatography (pentane/Et_2O, 7:3), compound **2h** was obtained in pure form as a yellow solid (316 mg, 1.4 mmol, 69% yield).

^{1}H NMR (400 MHz, 298 K, $CDCl_3$) δ (ppm) = 10.20 (d, $^4J_{HH}$ = 0.5 Hz, 1H), 8.41 – 8.36 (m, 2H), 8.33 – 8.28 (m, 2H), 8.08 – 8.00 (m, 3H).

^{13}C{^{1}H} NMR (101 MHz, 298 K, $CDCl_3$) δ (ppm) = 193.3 (CH), 155.3 (C_4), 153.0 (C_4), 148.6 (C_4), 143.9 (C_4), 138.4 (CH), 127.9 (2 × CH), 125.0 (CH), 124.2 (2 × CH), 121.1 (CH).

HRMS (ESI+) *m/z*: calculated for $C_{12}H_9N_2O_3$ $[M+H]^+$: 229.0608; found: 229.0607.

IR (neat) ν (cm^{-1}): 2820, 1707, 1516, 1341, 1107, 851, 805, 754.

mp: 136 °C.

3. Preparation of imines ((*R*,*R*)-4a-(*R*,*R*)-4j)

General procedure III (GP III)

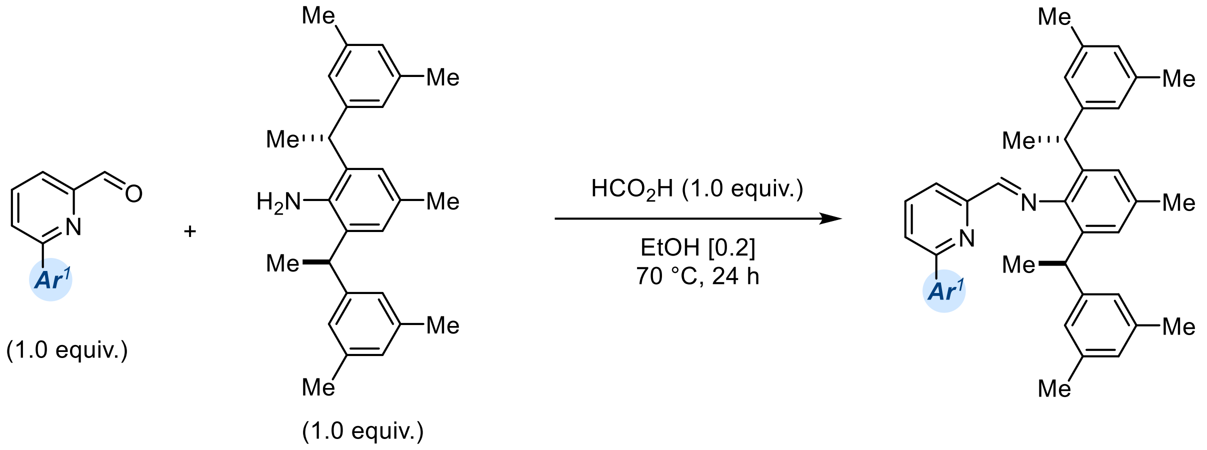

The protocol was adapted from a literature procedure.[10] The appropriate aldehyde (0.5 mmol, 1.0 equiv.) was dissolved in absolute EtOH (0.2 M) followed by appropriate amine (0.5 mmol, 1.0 equiv.) and formic acid (19 µL, 0.5 mmol, 1.0 equiv.). The reaction was heated at 70 °C for 24 h. The reaction was cooled to 23 °C and concentrated under reduce pressure. The product was purified by recrystallization by dissolving the crude mixture in a minimum amount of hot EtOH and letting it slowly cool to 23 °C. The crystals were collected by filtration and washed with cold EtOH.

General procedure IV (GP IV)

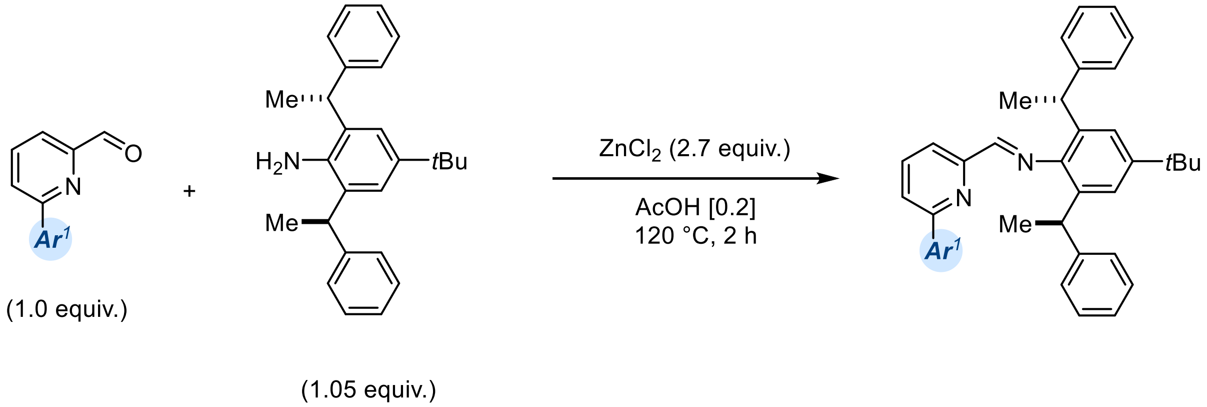

The protocol was adapted from a literature procedure.[2] A solution of the appropriate amine (1.05 equiv.), the appropriate aldehyde (1.0 equiv.) and zinc chloride (2.7 equiv.) were dissolved in acetic acid (0.2 M) and refluxed for 2 h. Acetic acid was distilled off under reduced pressure (130 °C, 800 to 480 mbar). The crude mixture was transferred to a separatory funnel using CH_2Cl_2 and saturated $NaHCO_3$ solution. The phases were separated and the aqueous phase was extracted CH_2Cl_2 (2 × 10 mL). The collected organic phases were washed once

with a saturated $NaHCO_3$ solution, water, brine and dried over Na_2SO_4 to afford the desired compound, which was used in the next step without further purification.

(*E*)-*N*-(2-((*R*)-1-(3,5-dimethylphenyl)ethyl)-4-methyl-6-((*R*)-1-(*m*-tolyl)ethyl)phenyl)-1-(6-phenylpyridin-2-yl)methanimine ((*R*,*R*)-4a)

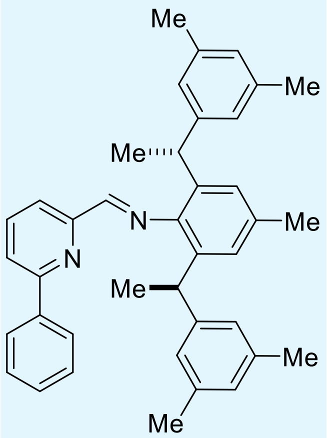

Following GP III, using 6-phenylpicolinaldehyde (92 mg, 0.5 mmol, 1.0 equiv.), amine (186 mg, 0.5 mmol, 1.0 equiv.) and formic acid (19 µL, 0.5 mmol, 1.0 equiv.). After recrystallization, compound **4a** was obtained in pure form as a yellow solid (235 mg, 0.44 mmol, 88% yield).

^{1}H NMR (400 MHz, 298 K, $CDCl_3$) δ (ppm) = 8.09 (dd, $^3J_{HH}$ = 7.7 Hz, $^4J_{HH}$ = 1.1 Hz, 1H), 8.06 – 8.01 (m, 2H), 7.89 (td, $^3J_{HH}$ = 7.7 Hz, $^4J_{HH}$ = 0.7 Hz, 1H), 7.83 (dd, $^3J_{HH}$ = 7.7 Hz, $^4J_{HH}$ = 1.1 Hz, 1H), 7.72 (d, $^4J_{HH}$ = 0.7 Hz, 1H), 7.53 – 7.45 (m, 3H), 7.08 (s, 2H), 6.77 – 6.73 (m, 2H), 6.73 – 6.68 (m, 4H), 4.03 (q, $^3J_{HH}$ = 7.2 Hz, 2H), 2.40 (s, 3H), 2.18 – 2.12 (m, 12H), 1.51 (d, $^3J_{HH}$ = 7.2 Hz, 6H).

^{13}C{^{1}H} NMR (101 MHz, 298 K, $CDCl_3$) δ (ppm) = 165.3 (CH), 157.0 (C_4), 154.4 (C_4), 147.5 (C_4), 146.5 (2 × C_4), 138.9 (C_4), 137.5 (2 × C_4), 137.1 (C_4), 134.5 (2 × C_4), 132. 8 (C_4), 129.2 (CH), 128.8 (2 × CH), 127.4 (2 × CH), 126.9 (2 × CH), 125.7 (4 × CH), 125.6 (2 × CH), 121.6 (CH), 119.4 (CH), 39.5 (2 × CH), 22.0 (2 × CH_3), 21.6 (CH_3), 21.3 (4 × CH_3).

HRMS (ESI+) *m/z*: calculated for $C_{39}H_{41}N_2$ $[M+H]^+$: 537.3265; found: 537.3248.

IR (neat) ν (cm^{-1}): 2965, 1642, 1600, 1447, 1203, 846, 760, 693.

mp: 66 °C.

$[\alpha]^{20}_D$ = 104.1 (*c* = 0.6 in CH_2Cl_2).

(*E*)-*N*-(4-(tert-butyl)-2,6-bis((*R*)-1-phenylethyl)phenyl)-1-(6-mesitylpyridin-2-yl)methanimine ((*R*,*R*)-4b)

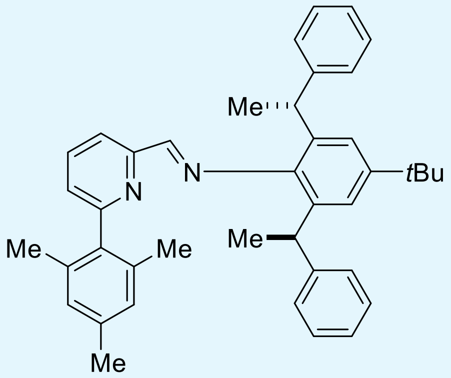

Following GP IV, using 6-mesitylpicolinaldehyde (128 mg, 0.57 mmol, 1.0 equiv.), amine (215 mg, 0.6 mmol, 1.05 equiv.) and zinc chloride (209 mg, 1.53 mmol, 2.7 equiv.). Compound **4b** was obtained in pure form as a brown foam (305 mg, 0.54 mmol, 95% yield).

^{1}H NMR (400 MHz, $CDCl_3$) δ (ppm) = 8.07 (dd, $^{3}J_{HH}$ = 7.8 Hz, $^{4}J_{HH}$ = 1.1 Hz, 1H), 7.84 (td, $^{3}J_{HH}$ = 7.8 Hz, $^{4}J_{HH}$ = 0.8 Hz, 1H), 7.69 (d, $^{4}J_{HH}$ = 0.6 Hz, 1H), 7.30 – 7.26 (m, 3H), 7.16 – 7.08 (m, 4H), 7.08 – 7.01 (m, 6H), 6.95 (s, 2H), 4.14 (q, $^{3}J_{HH}$ = 7.2 Hz, 2H), 2.33 (s, 3H), 2.04 (s, 6H), 1.54 (d, $^{3}J_{HH}$ = 7.2 Hz, 6H), 1.33 (s, 9H).

^{13}C{^{1}H} NMR (101 MHz, $CDCl_3$) δ (ppm) = 164.6 (CH), 159.8 (C_4), 154.4 (C_4), 147.3 (C_4), 146.9 (2 × C_4), 146.2 (C_4), 137.8 (C_4), 137.3 (C_4), 136.7 (CH), 135.9 (2 × C_4), 133.7 (2 × C_4), 128.6 (2 × CH), 128.2 (4 × CH), 127.5 (4 × CH), 126.3 (CH), 125.8 (CH), 122.2 (CH), 119.0 (CH), 39.8 (2 × CH), 34.8 (C_4), 31.7 (CH_3), 22.2 (2 × CH_3), 21.2 (CH_3), 20.4 (2 × CH_3).

HRMS (ESI+) *m/z*: calculated for $C_{41}H_{44}N_2$ $[M+H]^+$ 565.3583; found 565.3595.

IR (neat) ν (cm^{-1}): 2963, 1641, 1565, 1450, 1187, 881, 816, 757, 698.

mp: 63 °C.

$[\alpha]^{20}_{D}$ = 89.4 (*c* = 0.3 in CH_2Cl_2).

(*E*)-*N*-(4-(tert-butyl)-2,6-bis((*R*)-1-phenylethyl)phenyl)-1-(6-(2,4,6-triisopropylphenyl)pyridin-2-yl)methanimine ((*R*,*R*)-4c)

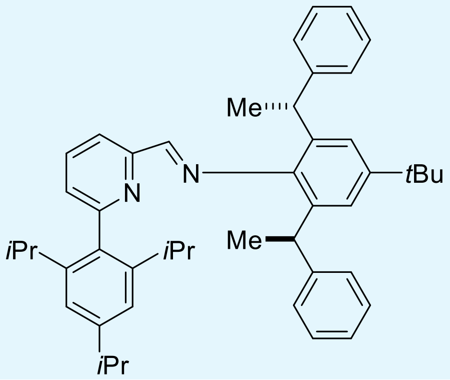

Following GP IV, using 6-(2,4,6-triisopropylphenyl)picolinaldehyde (460 mg, 1.49 mmol, 1.0 equiv.), amine (585 mg, 1.64 mmol, 1.1 equiv.) and zinc chloride (547 mg, 4.01 mmol, 2.7 equiv.). Compound **4c** was obtained in pure form as a yellow foam (916 mg, 1.41 mmol, 95% yield).

^{1}H NMR (400 MHz, $CDCl_3$) δ (ppm) = 8.11 (dd, $^3J_{HH}$ = 7.7 Hz, $^4J_{HH}$ = 1.1 Hz, 1H), 7.83 (td, $^3J_{HH}$ = 7.7 Hz, $^4J_{HH}$ = 0.7 Hz, 1H), 7.73 (d, $^4J_{HH}$ = 0.7 Hz, 1H), 7.34 (dd, $^3J_{HH}$ = 7.7 Hz, $^4J_{HH}$ = 1.1 Hz, 1H), 7.23 (s, 2H), 7.13 – 7.00 (m, 12H), 4.16 (q, $^3J_{HH}$ = 7.2 Hz, 2H), 2.93 (hept, $^3J_{HH}$ = 6.9 Hz, 1H), 2.50 (dh, $^3J_{HH}$ = 13.2, 6.6 Hz, 2H), 1.54 (d, $^3J_{HH}$ = 7.2 Hz, 6H), 1.31 (s, 9H), 1.28 (d, $^3J_{HH}$ = 6.9 Hz, 6H), 1.1 – 1.08 (m, 12H).

^{13}C{^{1}H} NMR (101 MHz, $CDCl_3$) δ (ppm) = 164.6 (CH), 159.8 (C_4), 154.4 (C_4), 147.3 (C_4), 146.9 (2 × C_4), 146.2 (C_4), 137.8 (C_4), 137.3 (C_4), 136.7 (CH), 135.9 (2 × C_4), 133.7 (2 × C_4), 128.6 (2 × CH), 128.2 (4 × CH), 127.5 (4 × CH), 126.3 (CH), 125.8 (CH), 122.2 (CH), 119.0 (CH), 39.8 (2 × CH), 34.8 (C_4), 31.7 (CH_3), 22.2 (2 × CH_3), 21.2 (CH_3), 20.4 (2 × CH_3).

HRMS (ESI+) *m/z*: calculated for $C_{47}H_{56}N_2$ $[M+H]^+$ 649.4521; found 649.4561.

IR (neat) ν (cm^{-1}): 2961, 2867, 1637, 1566, 1448, 1362, 1185, 1062, 880, 754, 701.

mp: 195 °C.

$[\alpha]^{20}_D$ = 26.7 (*c* = 0.5 in CH_2Cl_2).

(*E*)-*N*-(2,6-bis((*R*)-1-(3,5-dimethylphenyl)ethyl)-4-methylphenyl)-1-(6-(2,4,6-triisopropylphenyl)pyridin-2-yl)methanimine ((*R*,*R*)-4d)

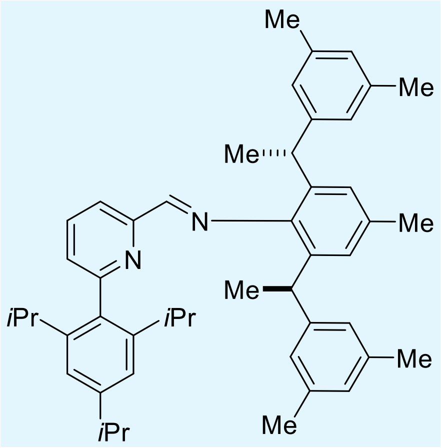

Following GP III, using 6-(2,4,6-triisopropylphenyl)picolinaldehyde (155 mg, 0.5 mmol, 1.0 equiv.), amine (186 mg, 0.5 mmol, 1.0 equiv.) and formic acid (19 µL, 0.5 mmol, 1.0 equiv.). After recrystallization, compound **4d** was obtained in pure form as a yellow solid (264 mg, 0.40 mmol, 80% yield).

^{1}H NMR (400 MHz, 298 K, $CDCl_3$) δ (ppm) = 8.13 (dd, $^3J_{HH}$ = 7.7 Hz, $^4J_{HH}$ = 1.1 Hz, 1H), 7.88 (t, $^3J_{HH}$ = 7.7 Hz, 1H), 7.78 (s, 1H), 7.39 (dd, $^3J_{HH}$ = 7.7 Hz, $^4J_{HH}$ = 1.1 Hz, 1H), 7.14 – 7.09 (m, 2H), 7.05 (s, 2H), 6.69 (s, 6H), 4.11 (q, $^3J_{HH}$ = 7.2 Hz, 2H), 2.96 (h. app. p, $^3J_{HH}$ = 6.9 Hz, 1H), 2.58 – 2.45 (m, 2H), 2.38 (s, 3H), 2.13 (s, 12H), 1.53 (d, $^3J_{HH}$ = 7.2 Hz, 6H), 1.31 (d, $^3J_{HH}$ = 6.9 Hz, 6H), 1.19 – 1.12 (m, 12H).

^{13}C{^{1}H} NMR (101 MHz, 298 K, $CDCl_3$) δ (ppm) = 164.8 (CH), 159.8 (C_4), 154.2 (C_4), 149.1 (C_4), 147.4 (C_4), 146.6 (2 × C_4), 146.4 (C_4), 146.1 (C_4), 137.3 (4 × C_4), 136.0 (CH), 135.8 (C_4), 134.6 (2 × C_4), 132.8 (C_4), 127.3 (2 × CH), 126.3 (CH), 125.7 (4 × CH), 125.6 (2 × CH), 121.0 (CH), 120.9 (CH), 119.1 (CH), 39.3 (2 × CH), 34.5 (CH), 30.5 (CH), 30.4 (CH), 24.6 (CH_3),

24.2 (CH_3), 24.1 (CH_3), 24.1 (CH_3), 24.0 (CH_3), 23.9 (CH_3), 22.0 (2 × CH_3), 21.5 (CH_3), 21.2 (4 × CH_3).

HRMS (ESI+) *m/z*: calculated for $C_{48}H_{59}N_2$ $[M+H]^+$: 663.4678; found: 663.4691.

IR (neat) ν (cm^{-1}): 2958, 1606, 1450, 1362, 1099, 845, 705.

mp: 186 °C.

$[\alpha]^{20}_D$ = 37.5 (*c* = 0.3 in CH_2Cl_2).

(*E*)-*N*-(2,6-bis((*R*)-1-(3,5-dimethylphenyl)ethyl)-4-methylphenyl)-1-(6-(3,5-dimethylphenyl)pyridin-2-yl)methanimine ((*R*,*R*)-4e)

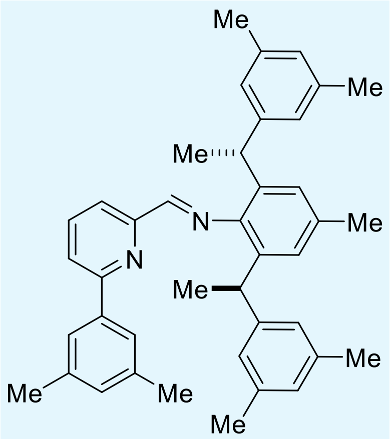

Following GP III, using 6-(3,5-dimethylphenyl)picolinaldehyde (106 mg, 0.5 mmol, 1.0 equiv.), amine (186 mg, 0.5 mmol, 1.0 equiv.) and formic acid (19 µL, 0.5 mmol, 1.0 equiv.). After recrystallization, compound **4e** was obtained in pure form as a yellow solid (242 mg, 0.43 mmol, 86% yield).

^{1}H NMR (400 MHz, 298 K, $CDCl_3$) δ (ppm) = 8.12 (dd, $^3J_{HH}$ = 7.7 Hz, $^4J_{HH}$ = 1.2 Hz, 1H), 7.90 (t, $^3J_{HH}$ = 7.7 Hz, 1H), 7.83 (dd, $^3J_{HH}$ = 7.7 Hz, $^4J_{HH}$ = 1.2 Hz, 1H), 7.77 (d, $^4J_{HH}$ = 1.2 Hz, 1H), 7.67 (s, 2H), 7.13 (s, 1H), 7.12 (s, 2H), 6.80 (s, 2H), 6.75 (s, 4H), 4.07 (q, $^3J_{HH}$ = 7.2 Hz, 2H), 2.47 (s, 6H), 2.44 (s, 3H), 2.19 (s, 12H), 1.54 (dd, $^3J_{HH}$ = 7.2, 1.4 Hz, 6H).

^{13}C{^{1}H} NMR (101 MHz, 298 K, $CDCl_3$) δ (ppm) = 165.5 (CH), 157.4 (C_4), 154.3 (C_4), 147.6 (C_4), 146.7 (2 × C_4), 138.9 (C_4), 138.3 (2 × C_4), 137.5 (3 × C_4), 137.0 (CH-3), 134.5 (2 × C_4), 132.7 (C_4), 130.9 (CH), 127.5 (2 × CH), 125.8 (4 × CH), 125.6 (2 × CH), 124.9 (2 × CH), 121.7 (CH), 119.2 (CH), 39.5 (2 × CH), 22.0 (2 × CH_3), 21.6 (CH_3), 21.5 (2 × CH_3), 21.4 (4 × CH_3).

HRMS (ESI+) *m/z*: calculated for $C_{41}H_{45}N_2$ $[M+H]^+$: 565.3578; found: 565.3575.

IR (neat) ν (cm^{-1}): 2968, 1448, 1371, 1135, 845, 808, 704.

mp: 70 °C.

$[\alpha]^{20}_D$ = 96.3 (*c* = 0.3 in CH_2Cl_2).

(*E*)-*N*-(2,6-bis((*R*)-1-(3,5-dimethylphenyl)ethyl)-4-methylphenyl)-1-(6-(3,5-dimethoxyphenyl)pyridin-2-yl)methanimine ((*R*,*R*)-4f)

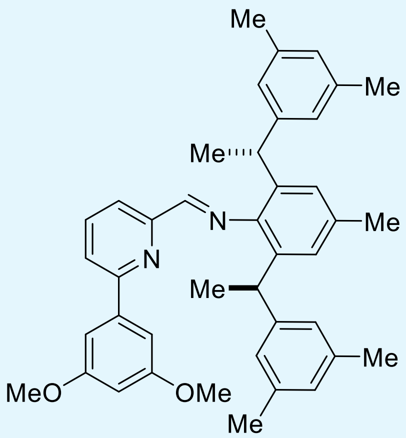

Following GP III, using 6-(3,5-dimethoxyphenyl)picolinaldehyde (122 mg, 0.5 mmol, 1.0 equiv.), amine (186 mg, 0.5 mmol, 1.0 equiv.) and formic acid (19 µL, 0.5 mmol, 1.0 equiv.). After recrystallization, compound **4f** was obtained in pure form as a yellow solid (236 mg, 0.40 mmol, 79% yield).

^{1}H NMR (400 MHz, 298 K, $CDCl_3$) δ (ppm) = 8.11 (dd, $^{3}J_{HH}$ = 7.8 Hz, $^{4}J_{HH}$ = 1.0 Hz, 1H), 7.89 (td, $^{3}J_{HH}$ = 7.8, Hz, $^{4}J_{HH}$ = 0.6 Hz, 1H), 7.81 (dd, $^{3}J_{HH}$ = 7.8 Hz, $^{4}J_{HH}$ = 1.0 Hz, 1H), 7.70 (d, $^{4}J_{HH}$ = 0.6 Hz, 1H), 7.20 (d, $^{4}J_{HH}$ = 2.3 Hz, 2H), 7.09 (s, 2H), 6.75 (dt, $^{4}J_{HH}$ = 1.8, 0.9 Hz, 2H), 6.71 – 6.68 (m, 4H), 6.58 (t, $^{4}J_{HH}$ = 2.3 Hz, 1H), 4.03 (q, $^{3}J_{HH}$ = 7.2 Hz, 2H), 3.90 (s, 6H), 2.41 (s, 3H), 2.15 (s, 12H), 1.52 (d, $^{3}J_{HH}$ = 7.2 Hz, 6H).

^{13}C{^{1}H} NMR (101 MHz, 298 K, $CDCl_3$) δ (ppm) = 165.3 (CH), 161.2 (C_4), 156.6 (C_4), 154.2 (C_4), 147.6 (C_4), 146.6 (2 × C_4), 141.0 (C_4), 137.5 (4 x C_4), 137.1 (CH), 134.5 (2 × C_4), 132.8 (C_4), 127.4 (2 × CH), 125.7 (4 × CH), 125.6 (2 × CH), 121.7 (CH), 119.6 (CH), 105.0 (2 × CH), 101.4 (CH), 55.5 (2 × CH_3), 39.5 (2 × CH_3), 22.0 (2 × CH_3), 21.5 (CH_3), 21.3 (4 × CH_3).

HRMS (ESI+) *m/z*: calculated for $C_{41}H_{45}N_2$ $[M+H]^+$: 565.3578; found: 565.3575.

IR (neat) ν (cm^{-1}): 2963, 1644, 1597, 1453, 1203, 1152, 845, 806.

mp: 72 °C.

$[\alpha]^{20}_{D}$ = 70.3 (*c* = 0.4 in CH_2Cl_2).

(*E*)-*N*-(2,6-bis((*R*)-1-(3,5-dimethylphenyl)ethyl)-4-methylphenyl)-1-(6-(3,5-bis(trifluoromethyl)phenyl)pyridin-2-yl)methanimine ((*R*,*R*)-4g)

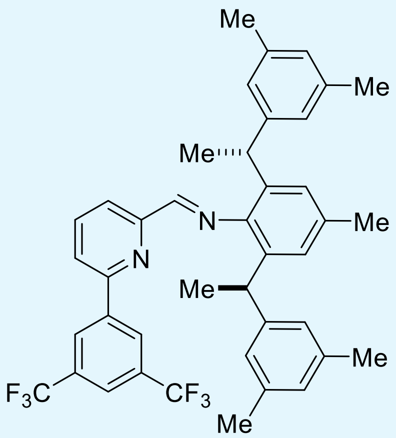

Following GP III, using 6-(3,5-bis(trifluoromethyl)phenyl)picolinaldehyde (16 mg, 0.05 mmol, 1.0 equiv.), amine (19 mg, 0.05 mmol, 1.0 equiv.) and formic acid (1.9 µL, 0.05 mmol, 1.0 equiv.). Compound **4g** was used without further purification for the next step.

(E)-N-(2,6-bis((R)-1-(3,5-dimethylphenyl)ethyl)-4-methylphenyl)-1-(6-(3,5-di-tert-butylphenyl)pyridin-2-yl)methanimine ((*R*,*R*)-4h)

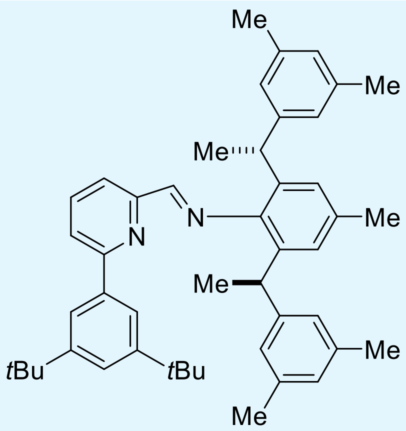

Following GP III, using 6-(3,5-di-tert-butylphenyl)picolinaldehyde (148 mg, 0.5 mmol, 1.0 equiv.), amine (186 mg, 0.5 mmol, 1.0 equiv.) and formic acid (19 µL, 0.5 mmol, 1.0 equiv.). After recrystallization, compound **4h** was obtained in pure form as a yellow solid (292 mg, 0.45 mmol, 90% yield).

^{1}H NMR (400 MHz, 298 K, $CDCl_3$) δ (ppm) = 8.13 (dd, $^3J_{HH}$ = 7.7 Hz, $^4J_{HH}$ = 1.1 Hz, 1H), 7.95 – 7.89 (m, 1H), 7.88 – 7.82 (m, 3H), 7.73 (d, $^4J_{HH}$ = 0.7 Hz, 1H), 7.57 (t, $^4J_{HH}$ = 1.8 Hz, 1H), 7.12 (s, 2H), 6.80 – 6.76 (m, 2H), 6.74 – 6.70 (m, 4H), 4.05 (t, $^3J_{HH}$ = 7.2 Hz, 2H), 2.43 (s, 3H), 2.17 (s, 12H), 1.55 (d, $^3J_{HH}$ = 7.2 Hz, 6H), 1.45 (s, 18H).

^{13}C{^{1}H} NMR (101 MHz, 298 K, $CDCl_3$) δ (ppm) = 165.8 (CH), 158.4 (C_4), 154.2 (C_4), 151.2 (2 × C_4), 147.8 (C_4), 146.6 (2 × C_4), 138.5 (C_4), 137.5 (4 × C_4), 136.9 (CH), 134.5 (2 × C_4), 132.7 (C_4), 127.4 (2 × CH), 125.8 (4 × CH), 125.5 (2 × CH), 123.4 (CH), 122.0 (CH), 121.5 (2 × CH), 118.9 (CH), 39.5 (2 × CH), 35.1 (2 × CH), 31.6 (2 × CH_3), 21.9 (2 × CH), 21.6 (CH), 21.3 (4 × CH_3).

HRMS (ESI+) *m/z*: calculated for $C_{47}H_{57}N_2$ $[M+H]^+$: 649.4521; found: 649.4515.

IR (neat) ν (cm^{-1}): 2964, 1647, 1568, 1462, 1249, 845, 809, 708.

mp: 104 °C.

[α]$^{20}_{D}$ = 72.7 (*c* = 0.5 in CH_2Cl_2).

(*E*)-*N*-(2,6-bis((*R*)-1-(3,5-dimethylphenyl)ethyl)-4-methylphenyl)-1-(6-(4-nitrophenyl)pyridin-2-yl)methanimine ((*R*,*R*)-4i)

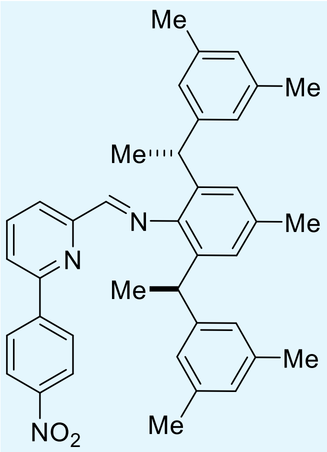

Following GP III, using 6-(4-nitrophenylpicolinaldehyde (114 mg, 0.5 mmol, 1.0 equiv.), amine (186 mg, 0.5 mmol, 1.0 equiv.) and formic acid (19 µL, 0.5 mmol, 1.0 equiv.). After recrystallization, compound **4i** was obtained in pure form as a yellow solid (289 mg, 0.5 mmol, 99% yield).

^{1}H NMR (400 MHz, 298 K, $CDCl_3$) δ (ppm) = 8.41 – 8.35 (m, 2H), 8.25 – 8.20 (m, 2H), 8.18 (dd, $^{3}J_{HH}$ = 7.7 Hz, $^{4}J_{HH}$ = 1.1 Hz, 1H), 7.98 (td, $^{3}J_{HH}$ = 7.7 Hz, $^{4}J_{HH}$ = 0.6 Hz, 1H), 7.92 (dd, $^{3}J_{HH}$ = 7.7 Hz, $^{4}J_{HH}$ = 1.1 Hz, 1H), 7.68 (s, 1H), 7.11 (s, 2H), 6.78 – 6.75 (m, 2H), 6.71 (d, $^{4}J_{HH}$ = 1.6 Hz, 4H), 4.02 (q, $^{3}J_{HH}$ = 7.2 Hz, 2H), 2.42 (s, 3H), 2.15 (s, 12H), 1.52 (d, $^{3}J_{HH}$ = 7.2 Hz, 6H).

^{13}C{^{1}H} NMR (101 MHz, 298 K, $CDCl_3$) δ (ppm) = 164.7 (CH), 154.8 (C_4), 154.3 (C_4), 148.3 (C_4), 147.3 (C_4), 146.6 (2 × C_4), 144.6 (C_4), 137.5 (5 × C_4), 137.5 (CH), 134.4 (C_4), 133.0 (C_4), 127.6 (2 × CH), 127.4 (2 × CH), 125.7 (6 × CH), 124.0 (2 × CH), 122.1 (CH), 120.8 (CH), 39.7 (2 × CH), 22.0 (4 × CH_3), 21.6 (CH_3), 21.3 (2 × CH_3).

HRMS (ESI+) *m/z*: calculated for $C_{39}H_{40}N_3O_2$ $[M+H]^+$: 579.3724; found: 579.3726.

IR (neat) ν (cm^{-1}): 2967, 1601, 1519, 1454, 1345, 845, 810, 754.

mp: 93 °C.

[α]$^{20}_{D}$ = 127.6 (*c* = 0.4 in CH_2Cl_2).

(*E*)-*N*-(2,6-bis((*R*)-1-(3,5-dimethylphenyl)ethyl)-4-methylphenyl)-1-(6-mesitylpyridin-2-yl)methanimine ((*R*,*R*)-4j)

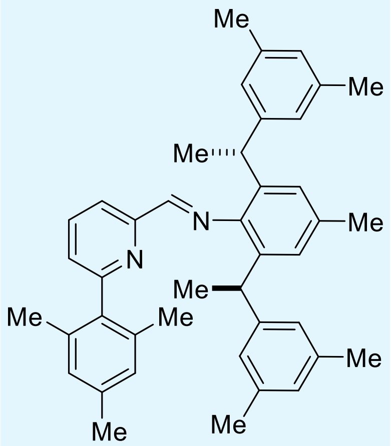

Following GP III, using 6-mesitylpicolinaldehyde (113 mg, 0.5 mmol, 1.0 equiv.), amine (186 mg, 0.5 mmol, 1.0 equiv.) and formic acid (19 µL, 0.5 mmol, 1.0 equiv.). After recrystallization, compound **4j** was obtained in pure form as a yellow solid (259 mg, 0.45 mmol, 89% yield).

^{1}H NMR (400 MHz, 298 K, $CDCl_3$) δ (ppm) = 8.11 (dd, $^3J_{HH}$ = 7.9 Hz, $^4J_{HH}$ = 1.1 Hz, 1H), 7.92 – 7.87 (m, 1H), 7.81 (s, 1H), 7.32 (dd, $^3J_{HH}$ = 7.6 Hz, $^4J_{HH}$ = 1.1 Hz, 1H), 7.05 (s, 2H), 6.98 (s, 2H), 6.71 (s, 6H), 4.10 (q, $^3J_{HH}$ = 7.2 Hz, 2H), 2.39 (s, 3H), 2.35 (s, 3H), 2.15 (s, 12H), 2.07 (s, 6H), 1.53 (d, $^3J_{HH}$ = 7.2 Hz, 6H).

^{13}C{^{1}H} NMR (101 MHz, 298 K, $CDCl_3$) δ (ppm) = 164.7 (CH), 159.7 (C_4), 154.5 (C_4), 147.3 (C_4), 146.5 (2 × C_4), 137.7 (C_4), 137.3 (4 × C_4), 137.2, (C_4) 136.5, 135.7 (2 × C_4), 134.6 (2 × C_4), 132.8 (C_4), 128.5 (2 × CH), 127.4 (2 × CH), 126.1 (CH), 125.7 (2 × CH), 125.6 (4 × CH), 119.1 (CH), 39.3 (2 × CH), 22.0 (2 × CH_3), 21.5 (CH_3), 21.3 (4 × CH_3), 21.1 (CH_3), 20.2 (2 × CH_3).

HRMS (ESI+) *m/z*: calculated for $C_{42}H_{46}N_2$ $[M+H]^+$: 579.3734; found: 579.3726.

IR (neat) ν (cm^{-1}): 2964, 1638, 1566, 1452, 1373, 846, 705.

mp: 72 °C.

$[\alpha]^{20}_D$ = 92.2 (*c* = 0.4 in CH_2Cl_2).

4. Preparation of imidazolium salts ((*R*,*R*)-5a-(*R*,*R*)-5j)

General procedure V (GP V)

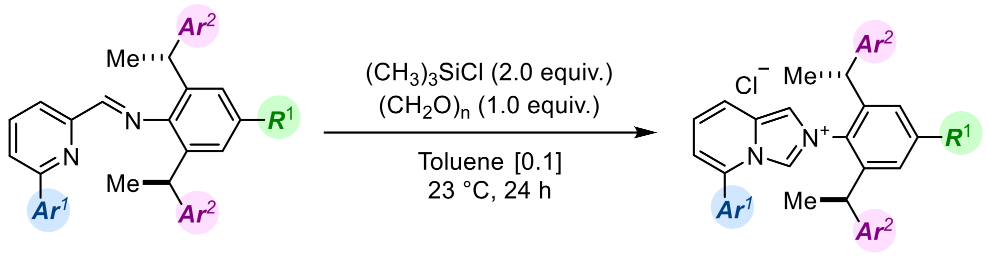

The protocol was adapted from a literature procedure.[6] To a solution of pyridine-imine (1.0 equiv.) and paraformaldehyde (1.0 equiv.) in toluene (0.1 M) chlorotrimethylsilane (2.0 equiv.) was added dropwise. The reaction was stirred at 23 °C for 24 h. The mixture was concentrated under reduce pressure and purified by precipitation in EtOAc and pentane.

2-(2,6-bis((*R*)-1-(3,5-dimethylphenyl)ethyl)-4-methylphenyl)-5-(phenyl)imidazo[1,5-*a*]pyridin-2-ium chloride ((*R*,*R*)-5a)

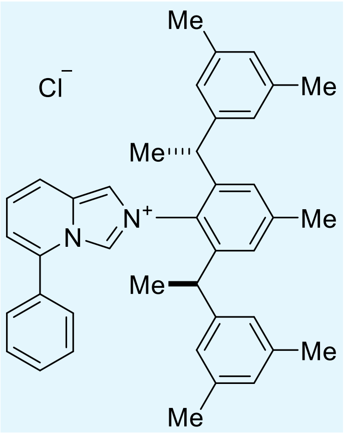

Following GP V, using pyridine-imine **4a** (188 mg, 0.35 mmol, 1.0 equiv.), paraformaldehyde (11 mg, 0.35 mmol, 1.0 equiv.) and chlorotrimethylsilane (89 µL, 0.7 mmol, 2.0 equiv.). Compound **5a** was obtained in pure form as a yellow solid (181 mg, 0.31 mmol, 88% yield).

^{1}H NMR (400 MHz, $CDCl_3$) δ (ppm) = 8.69 (s, 1H), 8.60 (s, 1H), 7.52 (t, $^3J_{HH}$ = 7.3 Hz, 1H), 7.47 – 7.31 (m, 4H), 7.29 (d, $^4J_{HH}$ = 1.6 Hz, 1H), 7.05 (s, 1H), 7.01 – 6.88 (m, 3H), 6.64 (s, 1H), 6.45 (s, 1H), 6.28 (s, 2H), 6.19 (s, 2H), 3.84 – 3.70 (m, 2H), 2.56 (s, 3H), 1.97 (s, 6H), 1.90 (s, 6H), 1.48 (t, $^3J_{HH}$ = 7.2 Hz, 6H).

^{13}C{^{1}H} NMR (101 MHz, $CDCl_3$)) δ (ppm) = 146.1 (C_4), 143.5 (C_4), 142.7 (C_4), 142.0 (C_4), 141.7 (C_4), 138.4 (2 × C_4), 138.1 (2 × C_4), 134.0 (C_4), 131.1 (CH), 130.6 (C_4), 130.5 (C_4), 129.6 (2 × CH), 129.5 (CH), 128.4 (CH), 128.4 (CH), 127.7 (2 × CH), 127.6 (3 × CH), 124.9 (3 × CH), 124.19 (2 × CH), 123.8 (2 × CH), 123.6 (CH), 120.4 (CH), 118.7 (CH), 118.6 (CH), 42.1 (CH), 39.9 (CH), 23.0 (CH_3), 21.9 (CH_3), 21.6 (CH_3), 21.1 (2 × CH_3), 21.1 (2 × CH_3).

HRMS (ESI+) *m/z*: calculated for $C_{40}H_{41}N_2$ $[M–Cl]^+$ 549.3270; found 542.3248.

IR (neat) ν (cm^{-1}): 2967, 1651, 1599, 1447, 1154, 848, 761, 706.

mp: 159 °C (dec.).

[α]^{20}$_D$ = 112.5 (*c* = 0.5 in CH_2Cl_2).

2-(4-(tert-butyl)-2,6-bis((R)-1-phenylethyl)phenyl)-5-mesitylimidazo[1,5-*a*]pyridin-2-ium chloride ((*R*,*R*)-5b)

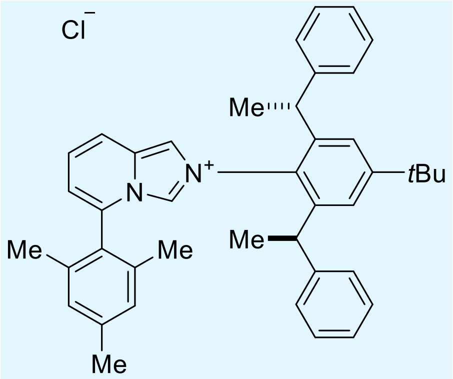

Following GP V, using pyridine-imine **4a** (82 mg, 0.15 mmol, 1.0 equiv.), paraformaldehyde (4.4 mg, 0.15 mmol, 1.0 equiv.) and chlorotrimethylsilane (37 µL, 0.29 mmol, 2.0 equiv.). Compound **5b** was obtained in pure form as a white solid (88 mg, 0.15 mmol, 99% yield).

^{1}H NMR (400 MHz, $CDCl_3$) δ (ppm) = 8.90 (d, $^4J_{HH}$ = 2.0 Hz, 1H), 8.71 (d, $^3J_{HH}$ = 9.3 Hz, 1H), 7.53 (d, $^4J_{HH}$ = 2.1 Hz, 1H), 7.47 (dd, $^3J_{HH}$ = 9.4, 6.9 Hz, 1H), 7.28 (d, $^4J_{HH}$ = 2.1 Hz, 1H), 7.24 – 7.21 (m, 2H), 7.21 – 7.16 (m, 2H), 7.06 – 7.02 (m, 3H), 6.99 – 6.93 (m, 3H), 6.93 – 6.89 (m, 2H), 6.73 – 6.69 (m, 2H), 3.97 (q, $^3J_{HH}$ = 7.0 Hz, 1H), 3.38 – 3.29 (m, 1H), 2.32 (s, 3H), 1.94 (s, 3H), 1.79 (s, 1H), 1.59 (d, $^3J_{HH}$ = 7.0 Hz, 3H), 1.45 (d, $^3J_{HH}$ = 7.2 Hz, 3H), 1.32 (s, 9H).

^{13}C{^{1}H} NMR (101 MHz, $CDCl_3$) δ 155.2 (C_4), 145.6 (C_4), 143.2 (C_4), 142.4 (C_4), 141.5 (C_4), 140.8 (C_4), 137.4 (C_4), 136.4 (C_4), 132.7 (C_4), 131.9 (C_4), 130.0 (CH), 129.7 (CH), 129.5 (2 × CH), 129.1 (2 × CH), 128.8 (2 × CH), 127.3 (CH), 127.1 (2 × CH), 126.7 (CH), 126.5 (2 × CH), 126.3 (CH), 125.6 (CH), 124.4 (CH), 124.0 (CH), 121.9 (C_4), 120.9 (CH), 120.8 (CH), 120.1 (CH), 40.0 (CH), 39.9 (CH), 35.5 (C_4), 31.3 (CH_3), 23.2 (CH_3), 22.7 (CH_3), 21.3 (CH_3), 20.2 (CH_3), 19.1 (CH_3).

HRMS (ESI+) *m/z*: calculated for $C_{42}H_{45}N_2$ $[M–Cl]^+$ 577.3583 found 577.3612.

IR (neat) ν (cm^{-1}): 2965, 2871, 1654, 1601, 1449, 1174, 744, 701, 656, 574.

mp: 69 °C (dec.).

[α]^{20}$_D$ = 103.7 (*c* = 0.3 in CH_2Cl_2).

2-(4-(tert-butyl)-2,6-bis((R)-1-phenylethyl)phenyl)-5-(2,4,6-triisopropylphenyl)imidazo[1,5-*a*]pyridin-2-ium chloride ((*R*,*R*)-5c)

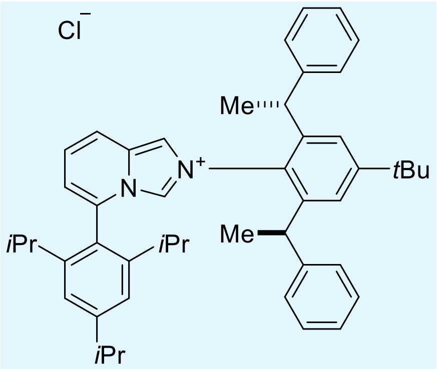

Following GP V, using pyridine-imine **4c** (400 mg, 0.61 mmol, 1.0 equiv.), paraformaldehyde (19 mg, 0.61 mmol, 1.0 equiv.) and chlorotrimethylsilane (156 µL, 1.23 mmol, 2.0 equiv.). Compound **5c** was obtained in pure form as a white solid (145 mg, 0.59 mmol, 97% yield).

^{1}H NMR (400 MHz, $CDCl_3$) δ (ppm) = 8.72 (d, $^3J_{HH}$ = 9.3 Hz, 1H), 8.60 (d, $^4J_{HH}$ = 1.9 Hz, 1H), 7.98 (d, $^4J_{HH}$ = 1.7 Hz, 1H), 7.60 (dd, $^3J_{HH}$ = 9.4, 6.9 Hz, 1H), 7.31 (d, $^4J_{HH}$ = 2.1 Hz, 1H), 7.25 – 7.18 (m, 5H), 7.18 – 7.10 (m, 4H), 7.08 (dd, $^3J_{HH}$ = 6.9 Hz, $^4J_{HH}$ = 0.9 Hz, 1H), 6.96 – 6.89 (m, 2H), 6.85 (dd, $^3J_{HH}$ = 7.8 Hz, $^4J_{HH}$ = 1.7 Hz, 2H), 3.49 (dd, $^3J_{HH}$ = 16.9, 7.1 Hz, 2H), 2.96 (p, $^3J_{HH}$ = 6.9 Hz, 1H), 2.34 (p, $^3J_{HH}$ = 6.8 Hz, 1H), 2.19 (p, $^3J_{HH}$ = 6.8 Hz, 1H), 1.66 (d, $^3J_{HH}$ = 7.0 Hz, 3H), 1.47 (d, $^3J_{HH}$ = 7.1 Hz, 3H), 1.30 (d, $^3J_{HH}$ = 6.9 Hz, 6H), 1.23 (s, 12H), 1.19 (d, $^3J_{HH}$ = 6.7 Hz, 3H), 1.08 (d, $^3J_{HH}$ = 6.8 Hz, 3H), 0.76 (d, $^3J_{HH}$ = 6.9 Hz, 3H).

^{13}C{^{1}H} NMR (101 MHz, $CDCl_3$) δ 155.4 (C_4), 153.2 (C_4), 148.4 (C_4), 147.9 (C_4), 143.7 (C_4), 143.4 (C_4), 143.0 (C_4), 141.4 (C_4), 132.5 (C_4), 132.2 (C_4), 129.5 (C_4), 129.3 (C_4), 128.6 (C_4), 127.5 (C_4), 127.2 (C_4), 126.8 (C_4), 126.8 (C_4), 126.1 (CH), 125.3 (C_4), 124.4 (C_4), 123.9 (CH), 122.7 (C_4), 122.5 (C_4), 121.9 (CH), 121.2 (CH), 121.1 (C_4), 120.8 (CH), 40.3 (CH), 39.3 (CH), 35.4 (C_4), 34.7 (CH), 31.6 (CH), 31.4 (CH), 31.1 (CH), 25.7 (CH_3), 25.4 (CH_3), 24.0 (CH_3), 24.0 (CH_3), 23.9 (CH_3), 23.7 (CH_3), 23.3 (CH_3), 23.0 (CH_3).

HRMS (ESI+) *m/z*: calculated for $C_{48}H_{57}N_2$ [M–Cl]$^+$ 661.4521 found 661.4517.

IR (neat) ν (cm^{-1}): 2961, 2869, 1603, 1459, 1364, 1182, 880, 744, 700, 656.

mp: 131 °C (dec.).

[α]$^{20}_D$ = 92.8 (*c* = 0.6 in CH_2Cl_2).

2-(2,6-bis((*R*)-1-(3,5-dimethylphenyl)ethyl)-4-methylphenyl)-5-(2,4,6-triisopropylphenyl)imidazo[1,5-*a*]pyridin-2-ium chloride ((*R*,*R*)-5d)

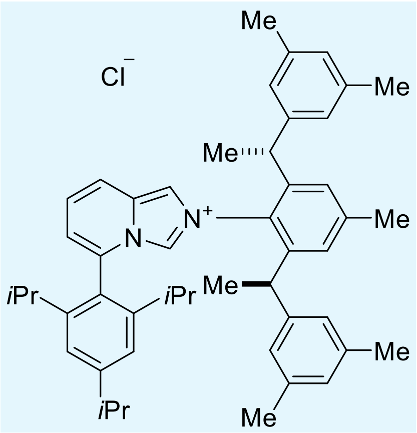

Following GP V, using pyridine-imine **4d** (232 mg, 0.35 mmol, 1.0 equiv.), paraformaldehyde (11 mg, 0.35 mmol, 1.0 equiv.) and chlorotrimethylsilane (89 µL, 0.70 mmol, 2.0 equiv.). Compound **5d** was obtained in pure form as a yellow solid (245 mg, 0.34 mmol, 98% yield).

^{1}H NMR (400 MHz, $CDCl_3$) δ (ppm) = 8.42 (d app. s, 1H), 8.03 (s, 1H), 7.82 (s, 1H), 7.64 (d, $^{3}J_{HH}$ = 7.0 Hz, 1H), 7.22 (d, $^{4}J_{HH}$ = 1.6 Hz, 1H), 7.19 – 7.13 (m, 2H), 7.10 (d, $^{3}J_{HH}$ = 6.1 Hz, 1H), 6.95 (d, $^{4}J_{HH}$ = 1.7 Hz, 1H), 6.81 (s, 1H), 6.75 (s, 1H), 6.47 (s, 2H), 6.27 (s, 2H), 3.40 (q, $^{3}J_{HH}$ = 6.7 Hz, 1H), 3.33 (d, $^{3}J_{HH}$ = 6.7 Hz, 1H), 2.97 (hept, $^{3}J_{HH}$ = 6.9 Hz, 1H), 2.43 (hept app. p, $^{3}J_{HH}$ = 6.8 Hz, 1H), 2.36 (s, 3H), 2.18 – 2.10 (m, 13H), 1.55 (d, $^{3}J_{HH}$ = 6.7 Hz, 3H), 1.44 (d, $^{3}J_{HH}$ = 6.7 Hz, 3H), 1.35 – 1.26 (m, 9H), 1.21 (d, $^{3}J_{HH}$ = 6.6 Hz, 3H), 1.07 (d, $^{3}J_{HH}$ = 6.4 Hz, 3H), 0.78 (d, $^{3}J_{HH}$ = 6.6 Hz, 3H).

^{13}C{^{1}H} NMR (101 MHz, $CDCl_3$) δ 153.2 (C_4), 148.3 (C_4), 147.6 (C_4), 143.8 (2 × C_4), 143.7 (C_4), 143.0 (C_4), 142.3 (C_4), 141.1 (C_4), 138.8 (2 × C_4), 137.9 (2 × C_4), 132.7 (C_4), 131.5 (C_4), 129.5 (C_4), 129.0 (CH), 128.8 (CH), 128.4 (CH), 127.1 (CH), 126.3 (CH), 124.9 (2 × CH), 124.2 (2 × CH), 122.6 (CH), 122.5 (CH), 121.6 (CH), 121.3 (CH), 120.1 (CH), 120.0 (CH), 40.6 (CH), 38.8 (CH), 34.5 (CH), 31.5 (CH), 31.3 (CH), 25.6 (CH_3), 25.1 (CH_3), 23.9 (CH_3), 23.8 (CH_3), 23.8 (CH_3), 23.7 (CH_3), 22.9 (CH_3), 22.7 (CH_3), 21.8 (CH_3), 21.3 (2 × CH_3), 21.3 (2 × CH_3).

HRMS (ESI+) *m/z*: calculated for $C_{49}H_{59}N_2$ $[M–Cl]^+$ 675.4673 found 675.4693.

IR (neat) ν (cm^{-1}): 2960, 1603, 1452, 1376, 1197, 847, 785.

mp: 111 °C (dec.).

$[\alpha]^{20}_{D}$ = 163.2 (*c* = 0.4 in CH_2Cl_2).

2-(2,6-bis((*R*)-1-(3,5-dimethylphenyl)ethyl)-4-methylphenyl)-5-(3,5-dimethylphenyl)imidazo[1,5-*a*]pyridin-2-ium chloride ((*R*,*R*)-5e)

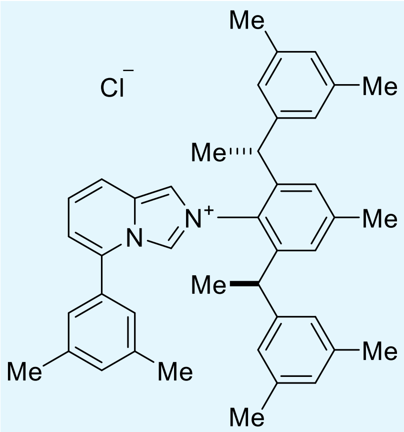

Following GP V, using pyridine-imine **4e** (226 mg, 0.40 mmol, 1.0 equiv.), paraformaldehyde (12 mg, 0.40 mmol, 1.0 equiv.) and chlorotrimethylsilane (102 µL, 0.80 mmol, 2.0 equiv.). Compound **5e** was obtained in pure form as a white solid (155 mg, 0.25 mmol, 63% yield).

^{1}H NMR (400 MHz, $CDCl_3$) δ (ppm) = 8.85 (s, 1H), 8.70 (s, 1H), 7.43 (s, 1H), 7.37 (s, 1H), 7.24 (s, 1H), 7.17–7.11 (m, 2H), 6.98 (d, $^3J_{HH}$ = 5.9 Hz), 6.68 (s, 2H), 6.60 (s, 1H), 6.53 (s, 1H), 6.31 (s, 2H), 6.18 (s, 2H), 3.94 (s, 1H), 3.64 (s, 1H), 2.55 (s, 3H), 2.36 (s, 6H), 2.00 (s, 6H), 1.94 (s, 6H),1.48 (d, $^3J_{HH}$ = 6.6 Hz, 6H).

^{13}C{^{1}H} NMR (101 MHz, $CDCl_3$) δ 145.9 (C_4), 143.3 (C_4), 142.4 (C_4), 141.9 (C_4), 141.9 (C_4), 139.6 (2 × C_4), 138.2 (2 × C_4), 138.1 (2 × C_4), 134.4 (C_4), 132.6 (CH), 131.4 (C_4), 130.7 (C_4), 130.6 (C_4), 128.8 (CH), 128.5 (CH), 128.5 (CH), 127.4 (CH), 125.5 (2 × CH), 124.9 (CH), 124.2 (2 × CH), 123.9 (2 × CH), 123.2 (CH), 120.8 (CH), 119.3 (CH), 118.4 (CH), 41.5 (CH), 40.3 (CH), 22.9 (CH_3), 22.0 (CH_3), 21.9 (CH_3), 21.3 (2 × CH_3), 21.2 (2 × CH_3), 21.1 (2 × CH_3).

HRMS (ESI+) *m/z*: calculated for $C_{42}H_{45}N_2$ $[M–Cl]^+$ 577.3578 found 577.3550.

IR (neat) ν (cm^{-1}): 2966, 1601, 1454, 11374, 1153, 1040, 847, 707.

mp: 213 °C (dec.).

$[\alpha]^{20}_D$ = 108.4 (*c* = 0.5 in CH_2Cl_2).

2-(2,6-bis((*R*)-1-(3,5-dimethylphenyl)ethyl)-4-methylphenyl)-5-(3,5-dimethoxyphenyl)imidazo[1,5-*a*]pyridin-2-ium ((*R*,*R*)-5f)

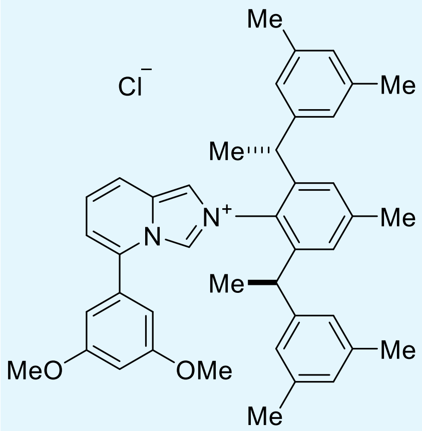

Following GP V, using pyridine-imine **4f** (209 mg, 0.35 mmol, 1.0 equiv.), paraformaldehyde (11 mg, 0.35 mmol, 1.0 equiv.) and chlorotrimethylsilane (89 µL, 0.7 mmol, 2.0 equiv.). Compound **5f** was obtained in pure form as a yellow solid (192 mg, 0.30 mmol, 85% yield).

^{1}H NMR (400 MHz, $CDCl_3$) δ (ppm) = 8.75 – 8.50 (m, 2H), 7.43 – 7.32 (m, 2H), 7.26 – 7.16 (m, 2H), 7.03 (d, $^{3}J_{HH}$ = 5.6 Hz, 1H), 6.61 – 6.47 (m, 3H), 6.28 (s, 2H), 6.21 – 6.12 (m, 4H), 3.85 (s, 7H), 3.65 (d, $^{3}J_{HH}$ = 7.7 Hz, 1H), 2.54 (s, 3H), 1.98 (s, 6H), 1.92 (s, 6H), 1.52–1.40 (m, 6H).

^{13}C{^{1}H} NMR (101 MHz, $CDCl_3$) δ 161.8 (C_4), 145.6 (C_4), 143.4 (C_4), 142.3 (C_4), 141.9 (2 × C_4), 138.3 (2 × C_4), 138.2 (2 × C_4), 134.0 (C_4), 132.2 (C_4), 131.2 (C_4), 130.6 (C_4), 128.8 (CH), 128.8 (CH), 128.5 (CH), 127.5 (CH), 124.9 (CH), 124.2 (2 × CH), 123.6 (2 × CH), 123.4 (CH), 120.6 (CH), 118.8 (CH), 118.4 (CH), 106.8 (2 × CH), 100.7 (CH), 55.7 (2 × CH), 41.6 (CH), 40.3 (CH), 22.8 (CH_3), 22.0 (CH_3), 21.8 (CH_3), 21.1 (2 × CH_3), 20.9 (2 × CH_3).

HRMS (ESI+) *m/z*: calculated for $C_{42}H_{45}N_2O_2$ $[M–Cl]^+$ 609.3476 found 609.3464.

IR (neat) ν (cm^{-1}): 2967, 1591, 1453, 1203, 1155, 1061, 846.

mp: 186 °C (dec.).

$[\alpha]^{20}_{D}$ = 97.6 (*c* = 0.4 in CH_2Cl_2).

2-(2,6-bis((*R*)-1-(3,5-dimethylphenyl)ethyl)-4-methylphenyl)-5-(3,5-bis(trifluoromethyl)phenyl)imidazo[1,5-*a*]pyridin-2-ium ((*R*,*R*)-5g)

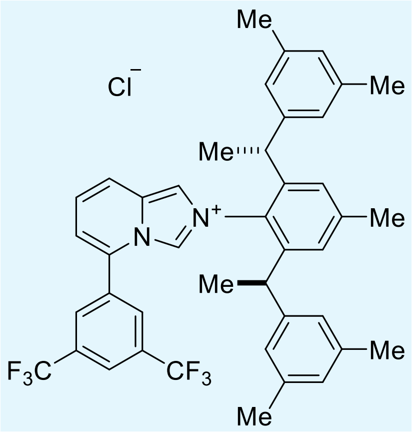

Following GP V, using pyridine-imine **4g** (209 mg, 0.35 mmol, 1.0 equiv.), paraformaldehyde (11 mg, 0.35 mmol, 1.0 equiv.) and chlorotrimethylsilane (89 µL, 0.7 mmol, 2.0 equiv.). Compound **5g** was used without further purification.

2-(2,6-bis((*R*)-1-(3,5-dimethylphenyl)ethyl)-4-methylphenyl)-5-(3,5-di-tert-butylphenyl)imidazo[1,5-*a*]pyridin-2-ium chloride ((*R*,*R*)-5h)

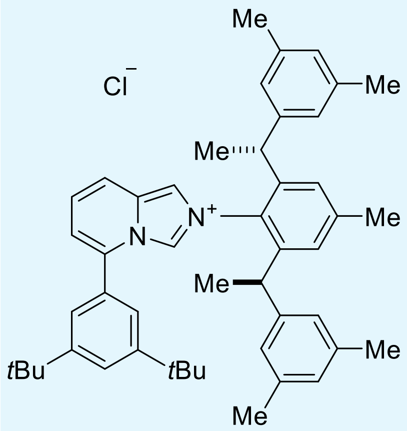

Following GP V, using pyridine-imine **4h** (227 mg, 0.35 mmol, 1.0 equiv.), paraformaldehyde (11 mg, 0.35 mmol, 1.0 equiv.) and chlorotrimethylsilane (89 µL, 0.7 mmol, 2.0 equiv.). Compound **5h** was obtained in pure form as a white solid (167 mg, 0.24 mmol, 68% yield).

^{1}H NMR (400 MHz, $CDCl_3$) δ (ppm) 8.70 – 8.52 (m, 2H), 7.62 (t, $^{4}J_{HH}$ = 1.7 Hz, 1H), 7.45 – 7.32 (m, 2H), 7.23 – 7.13 (m, 4H), 7.02 (d, $^{3}J_{HH}$ = 6.6 Hz, 1H), 6.73 (s, 1H), 6.39 (d, $^{4}J_{HH}$ = 3.4 Hz, 3H), 6.12 (s, 2H), 4.02 (d app. s, 1H), 3.50 (q, $^{3}J_{HH}$ = 7.0 Hz, 1H), 2.51 (s, 3H), 2.12 (s, 6H), 1.80 (s, 6H), 1.55 – 1.43 (m, 6H), 1.36 (s, 18H).

^{13}C{^{1}H} NMR (101 MHz, $CDCl_3$) δ (ppm) = 152.8 (C_4), 145.8 (C_4), 143.0 (C_4), 142.2 (C_4), 141.9 (C_4), 141.7 (C_4), 138.6 (2 × C_4), 137.7 (2 × C_4), 135.0 (C_4), 131.6 (C_4), 130.5 (C_4), 130.3 (C_4), 128.7 (CH), 128.0 (CH), 127.6 (CH), 127.4 (CH), 125.6 (CH), 125.1 (CH), 124.4 (2 × CH), 123.8 (2 × CH), 122.7 (CH), 121.5 (2 × CH), 120.4 (CH), 118.8 (CH), 118.7 (CH), 40.5 (CH), 40.1 (CH), 35.1 (C_4), 31.4 (CH_3), 22.6 (CH_3), 22.3 (CH_3), 22.0 (CH_3), 21.3 (2 × CH_3), 20.8 (2 × CH_3).

HRMS (ESI+) *m/z*: calculated for $C_{48}H_{57}N_2$ $[M–Cl]^+$ 661.4516 found 661.4517.

IR (neat) ν (cm^{-1}): 2962, 1598, 1455, 1364, 1203, 1152, 847, 709.

mp: 148 °C. (dec.).

[α]^{20}$_D$ = 17.4 (*c* = 0.7 in CH_2Cl_2).

2-(2,6-bis((*R*)-1-(3,5-dimethylphenyl)ethyl)-4-methylphenyl)-5-(4-nitrophenyl)imidazo[1,5-*a*]pyridin-2-ium chloride ((*R*,*R*)-5i)

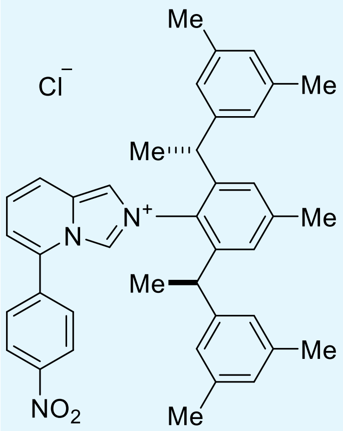

Following GP V, using pyridine-imine **4i** (204 mg, 0.35 mmol, 1.0 equiv.), paraformaldehyde (11 mg, 0.35 mmol, 1.0 equiv.) and chlorotrimethylsilane (89 µL, 0.70 mmol, 2.0 equiv.). Compound **5i** was obtained in pure form as a yellow solid (219 mg, 0.35 mmol, 99% yield).

^{1}H NMR (400 MHz, $CDCl_3$) δ(ppm) = 8.38 – 8-30 (m, 1H), 8.26 (d, $^3J_{HH}$ = 7.5 Hz, 2H), 8.11 (s, 1H), 7.71 – 7.63 (m, 1H), 7.43 – 7.31 (m, 5H), 7.20 (d, $^3J_{HH}$ = 6.4 Hz, 1H), 6.65 (s, 1H), 6.51 (s, 1H), 6.35 (s, 2H), 6.23 (s, 2H), 4.06 (s, 1H), 3.62 (d, $^3J_{HH}$ = 7.0 Hz, 1H1), 2.58 (s, 3H), 2.01 (s, 6H), 1.96 (s, 6H), 1.54–1.44 (m, 6H).

^{13}C{^{1}H} NMR (101 MHz, $CDCl_3$) δ(ppm) = 148.8 (C_4), 146.2 (C_4), 144.8 (C_4), 142.8 (C_4), 142.1 (C_4), 141.1 (C_4), 138.6 (2 × C_4), 138.0 (2 × C_4), 136.5 (C_4), 132.4 (C_4), 130.6 (C_4), 130.5 (C_4), 129.4 (2 × CH), 129.3 (CH), 128.2 (CH), 128.1 (CH), 127.5 (CH), 125.1 (CH), 124.9 (CH), 124.6 (2 × CH), 124.5 (CH), 124.0 (2 × CH), 120.6 (CH), 120.1 (CH), 118.2 (CH), 41.5 (CH), 39.9 (CH), 23.0 (CH_3), 22.0 (CH_3), 21.8 (CH_3), 21.2 (2 × CH_3), 21.1 (2 × CH_3).

HRMS (ESI+) *m/z*: calculated for $C_{40}H_{40}N_3O_2$ $[M–Cl]^+$ 594.3116 found 594.3112.

IR (neat) ν (cm^{-1}): 2968, 1599, 1518, 1345, 1155, 849, 679.

mp: 216 °C (dec.).

[α]^{20}$_D$ = 107.9 (*c* = 0.4 in CH_2Cl_2).

2-(2,6-bis((*R*)-1-(3,5-dimethylphenyl)ethyl)-4-methylphenyl)-5-mesitylimidazo[1,5-*a*]pyridin-2-ium chloride ((*R*,*R*)-5j)

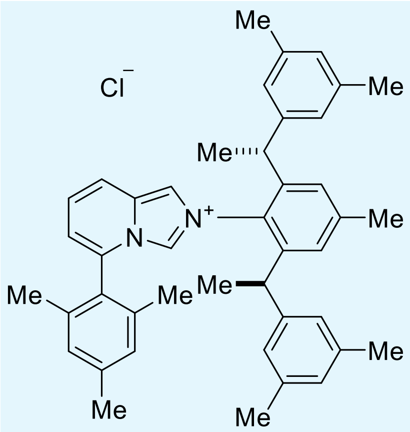

Following GP V, using pyridine-imine **4j** (259 mg, 0.45 mmol, 1.0 equiv.), paraformaldehyde (14 mg, 0.45 mmol, 1.0 equiv.) and chlorotrimethylsilane (114 µL, 0.9 mmol, 2.0 equiv.). Compound **5j** was obtained in pure form as a yellow solid (221 mg, 0.36 mmol, 79% yield).

^{1}H NMR (400 MHz, $CDCl_3$) δ (ppm) = 8.53 (d. app. s, 1H), 8.40 (s, 1H), 7.56 – 7.47 (m, 1H), 7.37 (s, 1H), 7.26 – 7.22 (m, 1H), 7.14 (d, $^4J_{HH}$ = 1.6 Hz, 1H), 7.06 – 6.97 (m, 2H), 6.93 (s, 1H), 6.81 (s, 1H), 6.70 (s, 1H), 6.39 (s, 2H), 6.31 (s, 2H), 3.74 (s, 1H), 3.30 (d, $^3J_{HH}$ = 6.6 Hz, 1H), 2.45 (s, 3H), 2.34 (s, 3H), 2.18 (s, 6H), 2.07 (s, 6H), 2.02 (d, $^4J_{HH}$ = 2.3 Hz, 3H), 1.63 (s, 3H), 1.54 (d, $^3J_{HH}$ = 5.1 Hz, 1H), 1.43 (d, $^3J_{HH}$ = 6.6 Hz, 3H).

^{13}C{^{1}H} NMR (101 MHz, $CDCl_3$) δ (ppm) = 144.9 (C_4), 143.5 (C_4), 142.1 (C_4), 142.1 (C_4), 141.5 (C_4), 141.3 (C_4), 138.6 (2 × C_4), 138.1 (2 × C_4), 137.2 (C_4), 136.4 (C_4), 132.8 (C_4), 131.3 (C_4), 129.9 (C_4), 129.5 (CH), 129.4 (CH), 128.8 (CH), 128.5 (CH), 127.9 (CH), 127.7 (CH), 126.2 (C_4), 125.8 (CH), 124.6 (2 × CH), 124.2 (2 × CH), 122.1 (CH), 120.4 (CH), 120.3 (CH), 119.5 (CH), 40.0 (CH), 39.1 (CH), 23.1 (CH_3), 22.6 (CH_3), 21.9 (CH_3), 21.4 (2 × CH_3), 21.3 (2 × CH_3), 21.3 (CH_3), 19.3 (CH_3), 19.2 (CH_3).

HRMS (ESI+) *m/z*: calculated for $C_{43}H_{47}N_2$ $[M–Cl]^+$ 591.3739 found 591.3729.

IR (neat) ν (cm^{-1}): 2967, 1601, 1452, 1376, 1197, 847, 785.

mp: 111 °C (dec.).

$[\alpha]^{20}_D$ = 175.1 (*c* = 0.6 in CH_2Cl_2).

5. Complexation to Au(I) ((*R*,*R*)-6a-(*R*,*R*)-6j)

General procedure VI (GP VI)

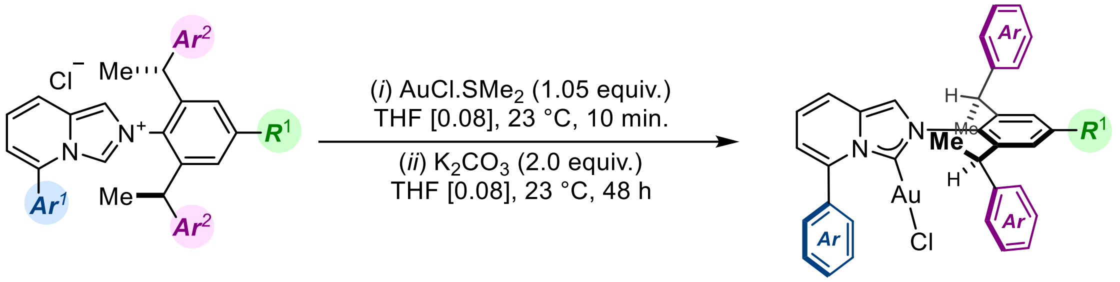

The protocol was adapted from the literature.[5] In a glovebox, a J-Young Schlenk tube was charged with the appropriate imidazolium salt (1.0 equiv.) and AuCl.SMe$_2$ (1.05 equiv.) followed by THF (0.08 M). After stirring for 10 min at 23 °C, K_2CO_3 (3.0 equiv.) was added. The J-Young Schlenk tube was closed and taken out of the glovebox. The reaction was stirred for the appropriate amount of hours, filtered through Celite, washed with CH_2Cl_2 (3 × 10 mL) and dried under reduced pressure. The residue was dissolved in a minimum amount of CH_2Cl_2 (ca. 1 mL) and the desired gold complex was precipitated by addition of pentane (ca. 10 mL) and isolated as an off white solid after drying under vacuum.

(2-(2,6-bis((*R*)-1-(3,5-dimethylphenyl)ethyl)-4-methylphenyl)-5-(phenyl)-2,3-dihydroimidazo[1,5-*a*]pyridin-3-ylidene)gold(I) chloride ((*R*,*R*)-6a)

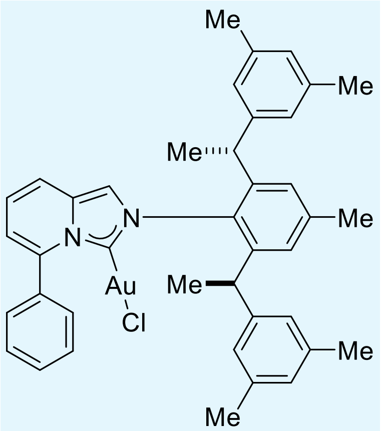

Following GP VI, using imidazolium salt **5a** (59 mg, 0.1 mmol, 1.0 equiv.), AuCl.SMe$_2$ (31 mg, 0.105 mmol, 1.05 equiv.) and K_2CO_3 (42 mg, 0.3 mmol, 3.0 equiv.). Compound **6a** was obtained in pure form as a white solid (50 mg, 0.64 mmol, 64% yield).

^{1}H NMR (400 MHz, $CDCl_3$) δ (ppm) = 7.62 – 7 .51 (m, 3H), 7.47 – 7.37 (m, 1H), 7.26 – 7.22 (m, 2H), 7.20 (dd, $^3J_{HH}$ = 9.2 Hz, $^4J_{HH}$ = 1.2 Hz, 1H), 7.03 (d, $^4J_{HH}$ = 1.8 Hz, 1H), 6.94 (dd, $^3J_{HH}$ = 9.2, 6.6 Hz, 1H), 6.80 (d, $^4J_{HH}$ = 1.7 Hz, 1H), 6.71 – 6.67 (m, 1H), 6.62 (d, $^4J_{HH}$ = 1.7 Hz, 2H), 6.59 (s, 1H), 6.51 (dd, $^3J_{HH}$ = 6.6 Hz, $^4J_{HH}$ = 1.2 Hz, 1H), 6.37 – 6.31 (m, 2H), 4.07 (q, $^3J_{HH}$ = 7.1 Hz, 1H), 3.56 (q, $^3J_{HH}$ = 7.1 Hz, 1H), 2.43 (s, 3H), 2.21 (s, 6H), 2.12 (s, 6H), 1.53 (d, $^3J_{HH}$ = 7.1 Hz, 3H), 1.445 (d, $^3J_{HH}$ = 7.1 Hz, 3H).

$^{13}C\{^1H\}$ NMR (101 MHz, $CDCl_3$) δ(ppm) = 165.8 (C_4), 146.2 (C_4), 144.2 (C_4), 142.2 (C_4), 141.9 (C_4), 139.8 (C_4), 139.6 (C_4), 138.0 (2 × C_4), 137.8 (2 × C_4), 135.1 (C_4), 134.3 (C_4), 130.8 (C_4), 130.0 (CH), 129.9 (CH), 129.5 (CH), 129.0 (CH), 128.7 (CH), 128.0 (CH), 127.4 (CH), 127.2 (CH), 126.9 (CH), 125.2 (2 × CH), 124.9 (2 × CH), 122.6 (CH), 116.9 (CH), 115.8 (CH), 114.1 (CH), 40.1 (CH), 39.1 (CH), 22.5 (CH_3), 22.3 (CH_3), 21.9 (CH_3), 21.4 (2 × CH_3), 21.2 (2 × CH_3).

HRMS (ESI+) *m/z*: calculated for $C_{40}H_{40}N_2Au$ $[M–Cl]^+$ 745.2852 found 745.2858.

IR (neat) ν (cm^{-1}): 2966, 1601, 1475, 1372, 1066, 1034.

mp: 200 °C (dec.).

$[\alpha]^{20}_D$ = 111.2 (c = 0.3 in CH_2Cl_2).

(2-(4-(tert-butyl)-2,6-bis((*R*)-1-phenylethyl)phenyl)-5-mesityl-2,3-dihydroimidazo[1,5-*a*]pyridin-3-ylidene)gold(I) chloride ((*R*,*R*)-6b)

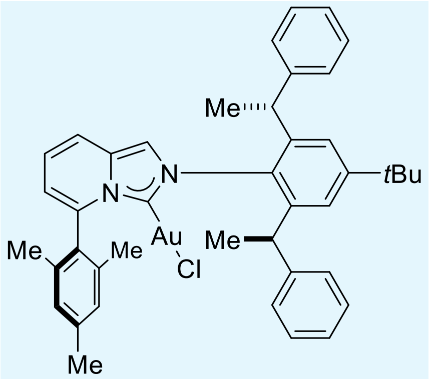

Following GP VI, using imidazolium salt **5b** (31 mg, 0.05 mmol, 1.0 equiv.), $AuCl.SMe_2$ (16 mg, 0.053 mmol, 1.05 equiv.) and K_2CO_3 (21 mg, 0.15 mmol, 3.0 equiv.). Compound **6b** was obtained in pure form as a white solid (14 mg, 0.018 mmol, 35% yield).

^1H NMR (400 MHz, $CDCl_3$) δ(ppm) = 7.38 (d, $^4J_{HH}$ = 2.2 Hz, 1H), 7.22 – 7.15 (m, 5H), 7.15 – 7.07 (m, 6H), 7.03 (s, 1H), 6.97 (dd, $^3J_{HH}$ = 9.2, 6.5 Hz, 1H), 6.81 – 6.75 (m, 2H), 6.58 (s, 1H), 6.51 (dd, $^3J_{HH}$ = 6.6 Hz, $^4J_{HH}$ = 1.3 Hz, 1H), 3.98 (q, $^3J_{HH}$ = 7.1 Hz, 1H), 3.60 (q, $^3J_{HH}$ = 7.1 Hz, 1H), 2.44 (s, 3H), 2.18 (s, 3H), 1.95 (s, 3H), 1.58 (d, $^3J_{HH}$ = 7.1 Hz, 3H), 1.49 (d, $^3J_{HH}$ = 7.1 Hz, 3H), 1.30 (s, 9H).

$^{13}C\{^1H\}$ NMR (101 MHz, $CDCl_3$) δ(ppm) = 164.6 (C_4), 152.9 (C_4), 146.3 (C_4), 143.9 (C_4), 142.5 (C_4), 141.2 (C_4), 140.8 (C_4), 138.4 (C_4), 136.9 (C_4), 136.6 (C_4), 134.5 (C_4), 130.7 (C_4), 130.5 (C_4), 129.0 (CH), 128.8 (CH), 128.5 (2 × CH), 128.3 (2 × CH), 127.6 (2 × CH), 127.1 (2 × CH), 126.2 (CH), 126.0 (CH), 124.3 (CH), 123.0 (CH), 123.0 (CH), 116.7 (CH), 116.4 (CH), 114.7 (CH), 40.2 (CH), 38.9 (CH), 35.1 (C_4), 31.3 (CH), 22.7 (CH_3), 22.4 (CH_3), 21.4 (CH_3), 20.0 (CH_3).

HRMS (ESI+) *m/z*: calculated for $C_{42}H_{44}N_2$ $[M–Au-Cl]^+$ 567.3500 found: 567.3558.

IR (neat) ν (cm^{-1}): 2963, 1602, 1479, 1451, 1178, 1029, 700.

mp: 135 °C (dec.).

[α]$^{20}_{575}$ = 132.4 (*c* = 0.4 in CH_2Cl_2).

(2-(4-(tert-butyl)-2,6-bis((*R*)-1-phenylethyl)phenyl)-5-(2,4,6-triisopropylphenyl)-2,3-dihydroimidazo[1,5-*a*]pyridin-3-ylidene)gold(I) chloride ((*R*,*R*)-6c)

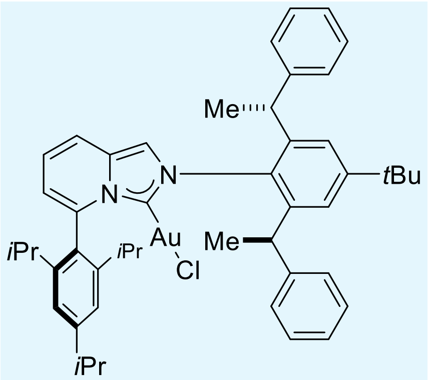

Following GP VI, using imidazolium salt **5c** (35 mg, 0.05 mmol, 1.0 equiv.), $AuCl.SMe_2$ (16 mg, 0.053 mmol, 1.05 equiv.) and K_2CO_3 (21 mg, 0.15 mmol, 3.0 equiv.). Compound **6c** was obtained in pure form as a white solid (23 mg, 0.026 mmol, 51% yield).

^{1}H NMR (400 MHz, $CDCl_3$) δ (ppm) = 7.34 – 7.29 (m, 3H), 7.25 – 7.07 (m, 9H), 7.02 – 6.94 (m, 2H), 6.87 – 6.79 (m, 2H), 6.69 (s, 1H), 6.57 (dd, $^3J_{HH}$ = 6.6 Hz, $^4J_{HH}$ = 1.2 Hz), 3.89 (q, $^3J_{HH}$ = 7.1 Hz, 1H), 3.53 (q, $^3J_{HH}$ = 7.1 Hz, 1H), 2.99 (h, $^3J_{HH}$ = 6.9 Hz, 1H), 2.50 (h app. p, $^3J_{HH}$ = 6.9 Hz, 1H), 2.37 (h, $^3J_{HH}$ = 6.9 Hz, 1H), 1.59 (d, $^3J_{HH}$ = 7.1 Hz, 3H), 1.50 (d, $^3J_{HH}$ = 7.1 Hz, 3H), 1.44 (d, $^3J_{HH}$ = 6.9 Hz, 3H), 1.39 (dd, $^3J_{HH}$ = 6.9 Hz, $^4J_{HH}$ = 3.7 Hz, 6H), 1.25 (s, 9H), 1.20 (d, $^3J_{HH}$ = 6.9 Hz, 3H), 1.09 (dd, $^3J_{HH}$ = 6.9 Hz, $^4J_{HH}$ = 3.1 Hz, 6H).

^{13}C{^{1}H} NMR (101 MHz, $CDCl_3$) δ (ppm) = 165.4 (C_4), 152.9 (C_4), 152.2 (C_4), 147.0 (C_4), 146.8 (C_4), 146.2 (C_4), 143.6 (C_4), 143.2 (C_4), 141.2 (C_4), 138.1 (C_4), 134.3 (C_4), 130.6 (C_4), 128.9 (C_4), 128.3 (CH), 128.2 (CH), 127.8 (CH), 127.1 (CH), 126.1 (CH), 126.0 (CH), 124.5 (CH), 122.9 (CH), 122.8 (CH), 122.2 (CH), 121.8 (CH), 117.2 (CH), 116.7 (CH), 114.7 (CH), 40.0 (CH), 38.5 (CH), 35.0 (C_4), 34.7 (CH), 31.8 (2 × CH), 31.2 (CH), 25.3 (CH_3), 25.3 (CH_3), 24.5 (CH_3), 24.3 (CH_3), 23.9 (CH_3), 23.4 (CH_3), 22.6 (CH_3), 22.1 (CH_3).

HRMS (ESI+) *m/z*: calculated for $C_{45}H_{56}N_2Au$ $[M–Cl]^+$ 857.4104 found: 857.4083.

IR (neat) ν (cm^{-1}): 2960, 1603, 1451, 1362, 1169, 875, 760, 701.

mp: 238 °C (dec.).

[α]$^{20}_{D}$ = 52.1 (*c* = 0.6 in CH_2Cl_2).

(2-(2,6-bis((*R*)-1-(3,5-dimethylphenyl)ethyl)-4-methylphenyl)-5-(2,4,6-triisopropylphenyl)-2,3-dihydroimidazo[1,5-*a*]pyridin-3-ylidene)gold(I) chloride ((*R*,*R*)-6d)

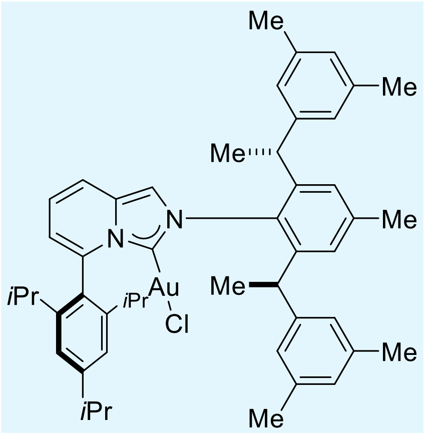

Following GP VI, using imidazolium salt **5d** (71 mg, 0.1 mmol, 1.0 equiv.), $AuCl.SMe_2$ (31 mg, 0.105 mmol, 1.05 equiv.) and K_2CO_3 (42 mg, 0.3 mmol, 3.0 equiv.). Compound **6d** was obtained in pure form as an off-white solid (68 mg, 0.75 mmol, 75% yield).

^{1}H NMR (400 MHz, $CDCl_3$) δ (ppm) = 7.25 – 7.20 (m, 2H), 7.17 (d, $^4J_{HH}$ = 1.7 Hz, 1H), 7.12 (d, $^4J_{HH}$ = 1.9 Hz, 1H), 7.02 – 6.96 (m, 3H), 6.77 – 6.70 (m, 3H), 6.60 – 6.55 (m, 2H), 6.36 (d, $^4J_{HH}$ = 1.7 Hz, 2H), 3.98 (q, $^3J_{HH}$ = 7.1 Hz, 1H), 3.33 (q, $^3J_{HH}$ = 7.1 Hz, 1H), 3.01 (hept, $^3J_{HH}$ = 6.9 Hz, 1H), 2.60 (h, $^3J_{HH}$ = 6.8 Hz, 1H), 2.40 (h app. p, $^3J_{HH}$ = 6.8 Hz, 1H), 2.31 (s, 3H), 2.23 (s, 6H), 2.17 (s, 6H), 1.55 – 1.43 (m, 9H), 1.40 (dd, $^3J_{HH}$ = 6.9, 4.8 Hz, 6H), 1.22 (d, $^3J_{HH}$ = 6.8 Hz, 3H), 1.15 (d, $^3J_{HH}$ = 6.8 Hz, 3H), 1.09 (d, $^3J_{HH}$ = 6.8 Hz, 3H).

^{13}C{^{1}H} NMR (101 MHz, $CDCl_3$) δ (ppm) = 165.2 (C_4), 152.2 (C_4), 147.0 (C_4), 146.7 (C_4), 146.3 (C_4), 144.2 (C_4), 143.5 (C_4), 141.5 (C_4), 140.1 (C_4), 138.1 (C_4), 137.8 (2 × C_4), 137.7 (2 × C_4), 134.4 (C_4), 130.5 (C_4), 129.0 (C_4), 127.9 (CH), 127.8 (CH), 127.6 (CH), 126.5 (CH), 125.6 (CH), 124.8 (CH), 122.6 (CH), 122.3 (CH), 121.7 (CH), 117.1 (CH), 116.7 (CH), 114.8 (CH), 40.0 (CH-), 37.7 (CH), 34.7 (CH), 31.8 (CH), 31.8 (CH), 25.3 (CH_3), 25.3 (CH_3), 24.5 (CH_3), 24.4 (CH_3), 23.9 (CH_3), 23.4 (CH_3), 22.3 (CH_3), 22.0 (CH_3), 21.8 (CH_3), 21.2 (2 × CH_3), 21.2 (2 × CH_3).

HRMS (ESI+) *m/z*: calculated for $C_{49}H_{58}N_2Au$ $[M–Cl]^+$ 871.4261 found 871.4178.

IR (neat) ν (cm^{-1}): 2959, 1603, 1458, 1379, 1168, 1066, 842, 792, 707.

mp: 244 °C (dec.).

$[\alpha]^{20}_D$ = 140.1 (*c* = 0.2 in CH_2Cl_2).

(2-(2,6-bis((*R*)-1-(3,5-dimethylphenyl)ethyl)-4-methylphenyl)-5-(3,5-dimethylphenyl)-2,3-dihydroimidazo[1,5-*a*]pyridin-3-ylidene)gold(I) chloride ((*R*,*R*)-6e)

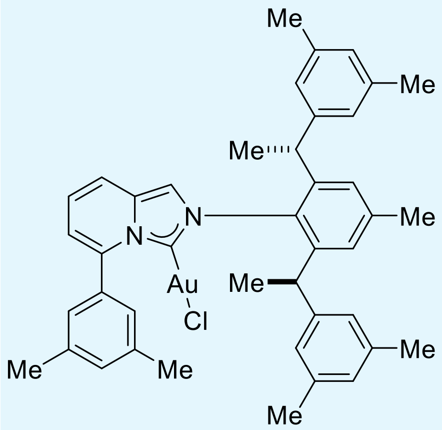

Following GP VI, using imidazolium salt **5e** (61 mg, 0.1 mmol, 1.0 equiv.), $AuCl.SMe_2$ (31 mg, 0.105 mmol, 1.05 equiv.) and K_2CO_3 (42 mg, 0.3 mmol, 3.0 equiv.). Compound **6e** was obtained in pure form as a pale yellow solid (68 mg, 0.84 mmol, 84% yield).

^{1}H NMR (400 MHz, $CDCl_3$) δ (ppm) = 7.23 (d, $^4J_{HH}$ = 1.8 Hz, 1H), 7.19 – 7.15 (m, 2H), 7.11 (s, 1H), 7.04 (d, $^4J_{HH}$ = 1.8 Hz, 1H), 6.98 – 6.89 (m, 2H), 6.79 (s, 1H), 6.68 (d, $^4J_{HH}$ = 1.8 Hz, 1H), 6.61 (d, $^4J_{HH}$ = 1.5 Hz, 2H), 6.57 (s, 1H), 6.49 (dd, $^3J_{HH}$ = 6.6 Hz, $^4J_{HH}$ = 1.3 Hz, 1H), 6.32 – 6.28 (m, 2H), 4.09 (q, $^3J_{HH}$ = 7.1 Hz, 1H), 3.58 (q, $^3J_{HH}$ = 7.1 Hz, 1H), 2.44 (s, 3H), 2.41 (s, 3H), 2.35 (s, 3H), 2.20 (s, 6H), 2.11 (s, 6H), 1.52 (d app. s, 3H), 1.45 (d, $^3J_{HH}$ = 7.1 Hz, 3H).

^{13}C{^{1}H} NMR (101 MHz, $CDCl_3$) δ (ppm) = 165.6 (C_4), 146.3 (C_4), 144.3 (C_4), 142.1 (C_4), 141.9 (C_4), 140.2 (C_4), 139.7 (C_4), 138.7 (C_4), 138.3 (C_4), 137.9 (2 × C_4), 137.7 (2 × C_4), 135.2 (C_4), 133.7 (C_4), 131.5 (CH), 130.8 (C_4), 128.2 (CH), 127.6 (CH), 127.3 (CH), 127.2 (CH), 127.0 (CH), 126.9 (CH), 125.1 (2 × CH), 124.9 (2 × CH), 122.5 (CH), 116.6 (CH), 115.4 (CH), 114.0 (CH), 40.1 (CH), 39.2 (CH), 22.5 (CH_3), 22.3 (CH_3), 22.0 (CH_3), 21.6 (CH_3), 21.3 (CH_3), 21.2 (CH_3).

HRMS (ESI+) *m/z*: calculated for $C_{42}H_{45}N_2Au$ $[M–Cl]^+$ 773.3170 found 773.3159.

IR (neat) ν (cm^{-1}): 2963, 2920, 1601, 1455, 1372, 1039, 846, 786, 704.

mp: 129 °C (dec.).

$[\alpha]^{20}_D$ = 158.1 (c = 0.4 in CH_2Cl_2).

(2-(2,6-bis((*R*)-1-(3,5-dimethylphenyl)ethyl)-4-methylphenyl)-5-(3,5-dimethoxylphenyl)-2,3-dihydroimidazo[1,5-*a*]pyridin-3-ylidene)gold(I) chloride ((*R*,*R*)-6f)

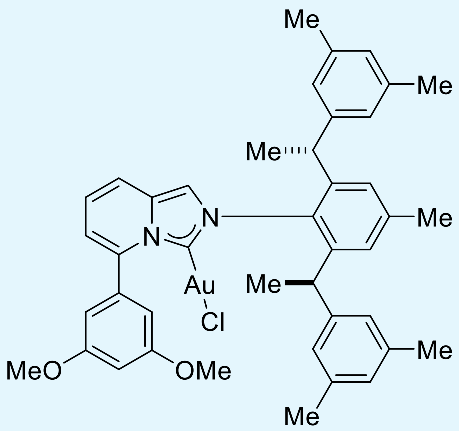

Following GP VI, using imidazolium salt **5f** (65 mg, 0.1 mmol, 1.0 equiv.), $AuCl.SMe_2$ (31 mg, 0.105 mmol, 1.05 equiv.) and K_2CO_3 (42 mg, 0.3 mmol, 3.0 equiv.). Compound **6f** was obtained in pure form as a pale yellow solid (60 mg, 0.71 mmol, 71% yield).

^{1}H NMR (400 MHz, $CDCl_3$) δ (ppm) = 7.23 – 7.17 (m, 2H), 6.97 (d, $^4J_{HH}$ = 1.8 Hz, 1H), 6.93 (dd, $^3J_{HH}$ = 9.2, 6.6 Hz, 1H), 6.77 (s, 1H), 6.73 – 6.65 (m, 5H), 6.59 (s, 1H), 6.56 – 6.51 (m, 2H), 6.34 (d, $^4J_{HH}$ = 1.8 Hz, 2H), 4.08 (q, $^3J_{HH}$ = 7.0 Hz, 1H), 3.88 (s, 3H), 3.81 (s, 3H), 3.54 (q, $^3J_{HH}$ = 7.0 Hz, 1H), 2.41 (s, 3H), 2.21 (s, 6H), 2.11 (s, 6H), 1.55 (d, $^3J_{HH}$ = 7.0 Hz, 3H), 1.45 (d, $^3J_{HH}$ = 7.0 Hz, 3H).

^{13}C{^{1}H} NMR (101 MHz, $CDCl_3$) δ (ppm) = 165.7 (C_4), 161.3 (C_4), 161.1 (C_4), 146.2 (C_4), 144.0 (C_4), 142.6 (C_4), 141.8 (C_4), 139.8 (C_4), 139.5 (C_4), 137.9 (C_4), 137.8 (C_4), 135.4 (C_4), 135.0 (C_4), 130.7 (C_4), 128.2 (CH), 127.4 (CH), 127.2 (CH), 126.8 (2 × CH), 125.2 (2 × CH), 124.9 (CH), 122.5 (CH), 117.0 (CH), 115.4 (CH), 114.2 (CH), 109.1 (CH), 108.6 (CH), 101.7 (CH), 55.6 (CH), 55.6 (CH), 40.1 (CH), 38.9 (CH), 22.4 (2 × CH_3), 22.3 (2 × CH_3), 21.9 (CH_3), 21.3 (CH_3), 21.2 (CH_3).

HRMS (ESI+) *m/z*: calculated for $C_{42}H_{44}N_2O_2Au$ $[M–Cl]^+$ 805.3063 found 805.3016.

IR (neat) ν (cm^{-1}): 2967, 1591, 1454, 1203, 1156, 1064, 844, 789.

mp: 122 °C (dec.).

$[\alpha]^{20}_D$ = 23.9 (*c* = 0.6 in CH_2Cl_2).

2-(2,6-bis((*R*)-1-(3,5-dimethylphenyl)ethyl)-4-methylphenyl)-5-(3,5-di-trifluoromethyl-butylphenyl)-2,3-dihydroimidazo[1,5-*a*]pyridin-3-ylidene)gold(I) chloride ((*R*,*R*)-6g)

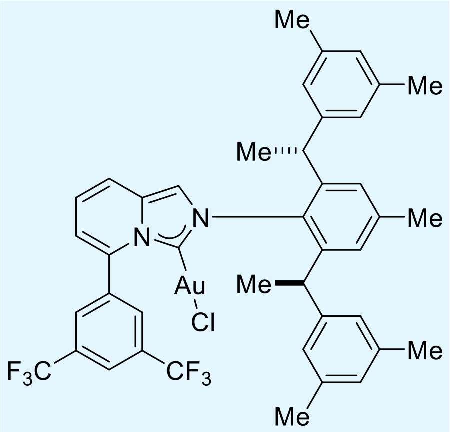

Following GP VI, using imidazolium salt **5g** (34 mg, 0.47 mmol, 1.0 equiv.), $AuCl.SMe_2$ (15 mg, 0.05 mmol, 1.05 equiv.) and K_2CO_3 (20 mg, 0.14 mmol, 3.0 equiv.). Compound **6g** was obtained in pure form as an off-white solid (14 mg, 0.15 mmol, 33% yield).

^{1}H NMR (400 MHz, $CDCl_3$) δ (ppm) = 8.12 – 8.03 (m, 1H), 7.86 (s, 1H), 7.71 (s, 1H), 7.34 (d, $^3J_{HH}$ = 9.1 Hz, 1H), 7.24 (d, $^4J_{HH}$ = 1.8 Hz, 1H), 7.10 (d, $^4J_{HH}$ = 1.8 Hz, 1H), 6.99 (dd, $^3J_{HH}$ = 9.2, 6.5 Hz, 1H), 6.78 (s, 1H), 6.72 (s, 1H), 6.70 (s, 1H), 6.58 – 6.53 (m, 1H), 6.48 – 6.42 (m, 2H), 6.35 – 6.28 (m, 2H), 4.02 (q, $^3J_{HH}$ = 7.1 Hz, 1H), 3.59 (q, $^3J_{HH}$ = 7.0 Hz, 1H), 2.45 (s, 3H), 2.14 (s, 6H), 2.12 (s, 6H), 1.55 (d, $^3J_{HH}$ = 7.1 Hz, 3H), 1.45 (d, $^3J_{HH}$ = 7.0 Hz, 3H).

^{13}C{^{1}H} NMR (101 MHz, $CDCl_3$) δ (ppm) = 165.8 (C_4), 146.1 (C4), 144.1 (C_4), 142.0 (C_4), 141.6 (C_4), 140.1 (C_4), 138.1 (2 × C_4), 137.8 (2 × C_4), 136.2 (C_4), 135.9 (C_4), 134.8 (C_4), 132.4 ($^2J_{C-F}$ = 34.3, Hz, C_4), 132.1 ($^2J_{C-F}$ = 34.3, Hz, C_4), 130.4 (CH), 130.5 (C_4), 130.1 (CH),128.1 (CH), 127.5 (CH), 127.1 (CH), 127.0 (CH), 124.8 (2 × CH), 124.8 (2 × CH), 124.1 (CH), 123.0 ($^1J_{C-F}$ = 273.4 Hz, CF_3), 122.1 (CH), 118.4 (CH), 117.0 (CH), 114.7 (CH), 40.1 (CH), 39.8 (CH), 22.6 (CH_3), 22.2 (CH_3), 22.0 (CH), 21.2 (2 × CH_3), 21.1 (2 × CH_3).

^{19}F{1H} NMR (282 MHz, $CDCl_3$) δ (ppm) = -62.8, -62.9 (CF_3).

HRMS (ESI+) *m/z*: calculated for $C_{42}H_{38}N_2AuF_6$ $[M–Cl]^+$ 881.2600 found 881.2599.

IR (neat) ν (cm^{-1}): 2966, 1599, 1475, 1364, 1249, 867, 846, 707.

mp: 126 °C (dec.).

$[\alpha]^{20}_D$ = 100.8 (*c* = 0.4 in CH_2Cl_2).

(2-(2,6-bis((*R*)-1-(3,5-dimethylphenyl)ethyl)-4-methylphenyl)-5-(3,5-di-tert-butylphenyl)-2,3-dihydroimidazo[1,5-*a*]pyridin-3-ylidene)gold(I) chloride ((*R*,*R*)-6h)

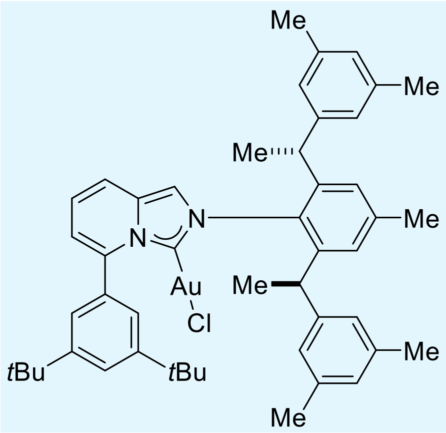

Following GP VI, using imidazolium salt **5h** (70 mg, 0.1 mmol, 1.0 equiv.), $AuCl.SMe_2$ (31 mg, 0.105 mmol, 1.05 equiv.) and K_2CO_3 (42 mg, 0.3 mmol, 3.0 equiv.). Compound **6h** was obtained in pure form as an off-white solid (14 mg, 0.15 mmol, 99% yield).

^{1}H NMR (400 MHz, $CDCl_3$) δ (ppm) = 7.62 (t, $^4J_{HH}$ = 1.7 Hz, 1H), 7.35 (t, $^4J_{HH}$ = 1.7 Hz, 1H), 7.23 (dd, $^3J_{HH}$ = 9.2 Hz, $^4J_{HH}$ = 1.2 Hz, 1H), 7.21 (t, $^4J_{HH}$ = 1.7 Hz, 1H), 7.15 (d, $^4J_{HH}$ = 1.9 Hz, 1H), 6.96 (dd, $^3J_{HH}$ = 9.3, 6.6 Hz, 1H), 6.87 (d, $^4J_{HH}$ = 1.9 Hz, 1H), 6.74 – 6.71 (m, 5H), 6.56 (dd, $^3J_{HH}$ = 6.6 Hz, $^4J_{HH}$ = 1.2 Hz, 1H), 6.46 – 6.44 (m, 2H), 3.99 (q, $^3J_{HH}$ = 7.1 Hz, 1H), 3.60 (q, $^3J_{HH}$ = 7.1 Hz, 1H), 2.35 (s, 3H), 2.18 (s, 6H), 2.16 (s, 6H), 1.57 (d, $^3J_{HH}$ = 7.1 Hz, 3H), 1.44 (d, $^3J_{HH}$ = 7.1 Hz, 3H), 1.42 (s, 9H), 1.36 (s, 9H).

^{13}C{^{1}H} NMR (101 MHz, $CDCl_3$) δ (ppm) = 166.4 (C_4), 151.5 (C_4), 151.2 (C_4), 146.1 (C_4), 143.8 (C_4), 143.0 (C_4), 142.0 (C_4), 140.9 (C_4), 139.8 (C_4), 137.8 (2 × C_4), 137.8 (2 × C_4), 134.8 (C_4), 133.4 (C_4), 130.9 (C_4), 128.0 (CH), 127.5 (CH), 127.3 (CH), 126.8 (CH), 125.3 (2 × CH), 125.0 (2 × CH), 124.4 (CH), 123.8 (CH), 123.3 (CH), 122.8 (CH), 116.5 (CH), 115.4 (CH), 114.0 (CH), 39.9 (CH), 38.7 (CH), 35.0 (C_4), 35.0 (C_4), 31.4 (CH), 22.3 (CH_3), 22.1 (CH_3), 21.8 (CH_3), 21.3 (2 × CH_3), 21.2 (2 × CH_3).

HRMS (ESI+) *m/z*: calculated for $C_{48}H_{56}N_2Au$ $[M–Cl]^+$ 857.4104 found 857.4083.

IR (neat) ν (cm^{-1}): 2962, 1599, 1475, 1364, 1249, 867, 846, 707.

mp: 123 °C (dec.).

$[\alpha]^{20}_D$ = 131.8 (*c* = 0.4 in CH_2Cl_2).

(2-(2,6-bis((*R*)-1-(3,5-dimethylphenyl)ethyl)-4-methylphenyl)-5-(4-nitrophenyl)-2,3-dihydroimidazo[1,5-*a*]pyridin-3-ylidene)gold(I) chloride ((*R*,*R*)-6i)

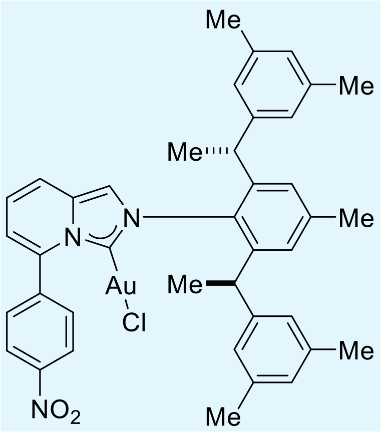

Following GP VI, using imidazolium salt **5i** (63 mg, 0.1 mmol, 1.0 equiv.), AuCl.SMe$_2$ (31 mg, 0.105 mmol, 1.05 equiv.) and K_2CO_3 (42 mg, 0.3 mmol, 3.0 equiv.). Compound **6i** was obtained in pure form as a yellow solid (76 mg, 0.92 mmol, 92% yield).

^{1}H NMR (400 MHz, $CDCl_3$) δ (ppm) = 8.49 – 8.35 (m, 1H), 8.22 (d, $^3J_{HH}$ = 7.4 Hz, 1H), 7.73 – 7.65 (m, 1H), 7.32 (d, $^3J_{HH}$ = 8.5 Hz, 1H), 7.10 (d, $^4J_{HH}$ = 1.9 Hz, 1H), 7.29–7.25 (m, 2H) 6.97 (dd, $^3J_{HH}$ = 9.3, 6.6 Hz, 1H), 6.84 (s, 1H), 6.70 (s, 1H), 6.63 (s, 1H), 6.56 – 6.49 (m, 3H), 6.30 (d, $^4J_{HH}$ = 1.6 Hz, 2H), 4.03 (q, $^3J_{HH}$ = 7.1 Hz, 1H), 3.56 (q, $^3J_{HH}$ = 7.1 Hz, 1H), 2.46 (s, 3H), 2.20 (s, 6H), 2.12 (s, 6H), 1.52 (d, $^3J_{HH}$ = 7.1 Hz, 3H), 1.46 (d, $^3J_{HH}$ = 7.1 Hz, 3H).

^{13}C{^{1}H} NMR (101 MHz, $CDCl_3$) δ (ppm) = 165.8 (C_4), 148.9 (C_4), 146.1 (C_4), 144.4 (C_4), 141.8 (C_4), 141.6 (C_4), 140.1 (2 × C_4), 138.2 (2 × C_4), 137.8 (2 × C_4), 137.1 (C_4), 134.9 (C_4), 131.3 (CH), 130.73 (CH), 130.5 (C_4), 128.0 (CH), 127.5 (CH), 127.2 (CH), 127.1 (CH), 125.1 (2 × CH), 124.8 (2 × CH), 124.43 (CH), 123.93 (CH), 122.2 (CH), 118.1 (CH), 116.4 (CH), 114.5 (CH), 40.2 (CH), 39.4 (CH), 22.7 (CH_3), 22.3 (CH_3), 22.0 (CH_3), 21.4 (CH_3), 21.2 (CH_3).

HRMS (ESI+) *m/z*: calculated for $C_{49}H_{39}N_3O_2Au$ [M–Cl]$^+$ 790.2703 found 790.2710.

IR (neat) ν (cm^{-1}): 2971, 1600, 1520, 1347, 1198, 1076, 1036, 850, 707.

mp: 135 °C (dec.).

$[\alpha]^{20}_{575}$ = 159.0 (*c* = 0.3 in CH_2Cl_2).

(2-(2,6-bis((*R*)-1-(3,5-dimethylphenyl)ethyl)-4-methylphenyl)-5-mesityl-2,3-dihydroimidazo[1,5-*a*]pyridin-3-ylidene)gold(I) chloride ((*R*,*R*)-6j)

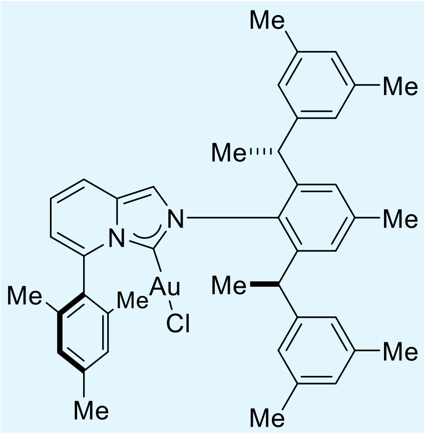

Following GP VI, using imidazolium salt **5j** (63 mg, 0.1 mmol, 1.0 equiv.), AuCl.SMe$_2$ (31 mg, 0.105 mmol, 1.05 equiv.) and K_2CO_3 (42 mg, 0.3 mmol, 3.0 equiv.). Compound **6j** was obtained in pure form as an off-white solid (59 mg, 0.72 mmol, 72% yield).

^{1}H NMR (400 MHz, CDCl$_3$) δ(ppm) = 7.21 (dd, $^3J_{HH}$ = 9.2 Hz, $^4J_{HH}$ = 1.2 Hz, 1H), 7.15 (d, $^4J_{HH}$ = 1.9 Hz, 1H), 7.12 (s, 1H), 7.03 – 7.01 (m, 1H), 7.00 – 6.94 (m, 1H), 6.92 (d, $^4J_{HH}$ = 1.6 Hz, 2H), 6.83 (d, $^4J_{HH}$ = 1.9 Hz, 1H), 6.77 (s, 1H), 6.72 (s, 1H), 6.55 – 6.50 (m, 2H), 6.34 (d, $^4J_{HH}$ = 1.6 Hz, 2H), 3.99 (q, $^3J_{HH}$ = 7.1 Hz, 1H), 3.40 (q, $^3J_{HH}$ = 7.1 Hz, 1H), 2.44 (s, 3H), 2.35 (s, 3H), 2.24 (d, $^4J_{HH}$ = 1.7 Hz, 9H), 2.13 (s, 6H), 1.94 (s, 3H), 1.53 (d, $^3J_{HH}$ = 7.1 Hz, 3H), 1.44 (d, $^3J_{HH}$ = 7.1 Hz, 3H).

^{13}C{^{1}H} NMR (101 MHz, CDCl$_3$) δ(ppm) = 164.7 (C$_4$), 146.3 (C$_4$), 143.7 (C$_4$), 143.5 (C$_4$), 141.6 (C$_4$), 140.8 (C$_4$), 140.0 (C$_4$), 138.4 (C$_4$), 137.9 (C$_4$), 137.8 (C$_4$), 137.0 (C$_4$), 136.6 (C$_4$), 134.5 (C$_4$), 130.7 (C$_4$), 130.5 (C$_4$), 129.0 (CH), 128.9 (CH), 128.0 (CH), 127.7 (CH), 127.6 (CH), 126.6 (CH), 125.5 (CH), 124.9 (CH), 122.9 (CH), 116.7 (CH), 116.2 (CH), 114.7 (CH), 38.1 (CH), 22.4 (CH), 22.2 (CH$_3$), 21.8 (CH$_3$), 21.5 (CH$_3$), 21.3 (2 × CH$_3$), 21.2 (2 × CH$_3$), 20.2 (CH$_3$), 19.8 (CH$_3$).

HRMS (ESI+) *m/z*: calculated for $C_{43}H_{46}N_2Au$ [M–Cl]$^+$ 787.3321 found 787.3290.

IR (neat) ν (cm^{-1}): 2964, 1601, 1472, 1372, 1159, 1036, 847, 708.

mp: 124 °C (dec.).

$[\alpha]^{20}_{436}$ = 300.6 (*c* = 0.3 in CH_2Cl_2).

6. Substrate synthesis

Starting materials **7a**-**7d** and **7f**-**7j** were prepared according to literature procedure.[11]

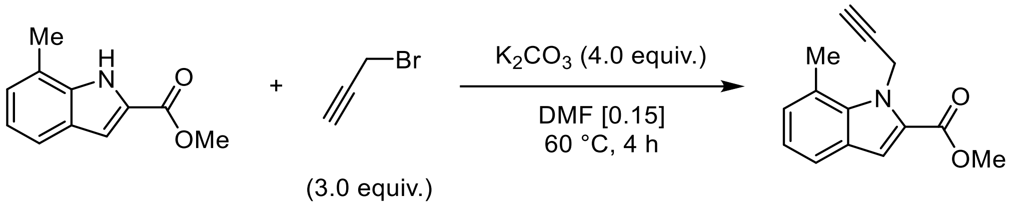

Methyl 7-methyl-1-(prop-2-yn-1-yl)-1H-indole-2-carboxylate

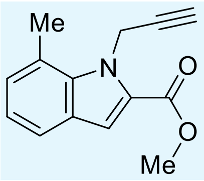

This procedure was adapted from the literature.[11] In a Schlenk flask under nitrogen, methyl 7-methyl-1H-indole-2-carboxylate (284 mg, 1.5 mmol, 1.0 equiv.) was dissolved in DMF (0.15 M) and K_2CO_3 (622 mg, 4.5 mmol, 3.0 equiv.) was added. Next, propargyl bromide (80% in toluene, 0.67 mL, 6 mmol, 4.0 equiv.) was added dropwise, the Schlenk flask was closed and the reaction heated to 60 °C. After 16h, the reaction was cool to 23 °C and quenched with a saturated solution of sodium bicarbonate. The mixture was extracted with EtOAc (3 × 10 mL) and the organic phase was washed with distilled water (2 × 20 mL) and brine (1 × 20 mL). The combined organic phases were dried over Na_2SO_4, filtered and concentrated under reduce pressure. Purification by flash chromatography (cyclohexane/EtOAc, 20:1) afforded the pure compound as a white solid (291 mg, 0.98 mmol, 85%).

^{1}H NMR (400 MHz, $CDCl_3$) δ (ppm) = 7.53 (ddd, $^3J_{HH}$ = 7.7 Hz, $^4J_{HH}$ = 1.5, 0.7 Hz, 1H), 7.35 (s, 1H), 7.14 – 7.04 (m, 2H), 5.67 (d, $^4J_{HH}$ = 2.4 Hz, 2H), 3.94 (s, 3H), 2.90 (d, $^4J_{HH}$ = 0.7 Hz, 3H), 2.34 (t, $^4J_{HH}$ = 2.4 Hz, 1H).

^{13}C{^{1}H} NMR (101 MHz, $CDCl_3$) δ (ppm) = 162.4 (C_4), 138.3 (C_4) , 128.6 (CH), 127.2 (C_4), 127.1 (C_4), 122.1 (C_4), 121.3 (CH), 120.9 (CH), 112.9 (CH), 80.4 (C_4), 72.9 (CH), 51.8 (CH_3), 35.9 (CH_2), 19.8 (CH_3).

HRMS (ESI+) *m/z*: calculated for $C_{12}H_{11}N$ [M–COOMe]$^+$ 169.0866 found: 169.0877.

IR (neat) ν (cm^{-1}): 3270, 1711, 1486, 1440, 1318, 1254, 1200, 1102, 938, 740.

mp: 88 °C.

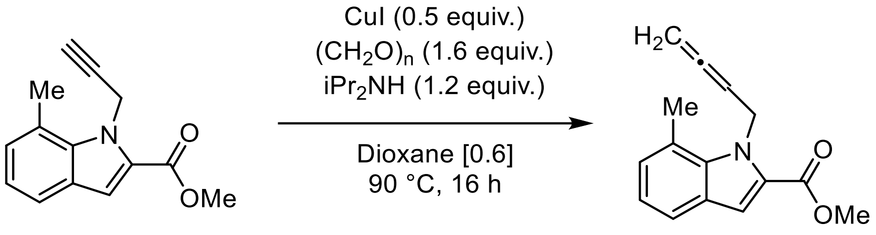

Methyl 1-(buta-2,3-dien-1-yl)-7-methyl-1H-indole-2-carboxylate

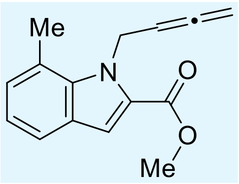

This procedure was adapted from the literature.[11] In the glovebox, a J-Young flask was charged with copper iodide (119 mg, 0.63 mmol, 0.5 equiv.) followed by 1,4-dioxane (0.6 M). Next, methyl 7-methyl-1-(prop-2-yn-1-yl)-1H-indole-2-carboxylate (285 mg, 1.25 mmol, 1.0 equiv.) and paraformaldehyde were added. The J-Young flask was sealed and taken out of the glovebox and connected to a double manifold vacuum/nitrogen Schlenk line before adding diisopropylamine (210 µL, 1.5 mmol, 1.2 equiv.). The J-Young flask was closed and stirred at 90 °C for 16 hours. After cooling to 23 °C, the reaction was quenched with water (5 mL) and diluted with EtOAc (5 mL) and extracted with EtOAc (3 × 5 mL). The combined organic phases were washed with a diluted ammonia solution (2 × 25 mL) and extracted with a separatory funnel. The organic phase was collected, dried over Na_2SO_4, filtered and concentrated under reduce pressure. Purification by flash chromatography (cyclohexane/EtOAc 10:1) afforded the pure compound as a white solid (238 mg, 0.99 mmol, 79%)

^{1}H NMR (400 MHz, $CDCl_3$) δ (ppm) = 7.54 – 7.50 (m, 1H), 7.33 (s, 1H), 7.08 – 7.01 (m, 2H), 5.47 (dt, $^4J_{HH}$ = 5.4 Hz, $^4J_{HH}$ = 3.2 Hz, 2H), 5.38 (qd, $^3J_{HH}$ = 6.5, 5.4 Hz, 1H), 4.68 (dt, $^3J_{HH}$ = 6.5 Hz, $^4J_{HH}$ = 3.2 Hz, 2H), 3.92 (s, 3H), 2.80 (t, $^4J_{HH}$ = 0.8 Hz, 3H).

^{13}C{^{1}H} NMR (101 MHz, $CDCl_3$) δ (ppm) = 207.7 (C_4), 162.4 (C_4), 138.3 (C_4), 128.3 (2 × Ch), 127.4 (C_4), 127.0 (C_4), 122.0 (C_4), 120.8 (CH), 112.3 (CH), 90.1 (CH), 77.4 (CH_2), 51.7 (CH_3), 44.0 (CH_2), 20.4 (CH_3).

HRMS (ESI+) *m/z*: calculated for $C_{15}H_{16}NO_2$ $[M+H]^+$ 242.1176 found 242.1169.

IR (neat) ν (cm^{-1}): 2974, 1958, 1697, 1450 1249, 1189, 1093, 840, 742.

mp: 70 °C.

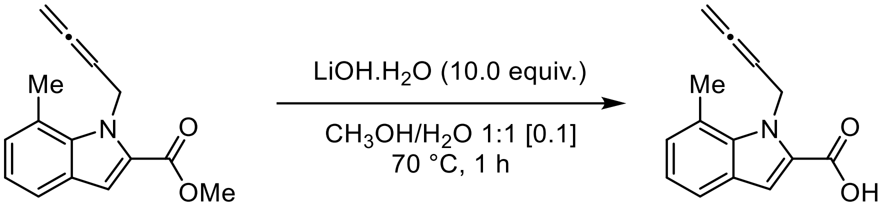

1-(buta-2,3-dien-1-yl)-7-methyl-1H-indole-2-carboxylic acid (7e)

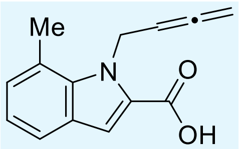

This procedure was adapted from the literature.[11] In a round bottom flask, methyl 1-(buta-2,3-dien-1-yl)-7-methyl-1H-indole-2-carboxylate (238 mg, 0.99 mmol, 1.0 equiv) was added followed by methanol (0.1 M). The flask was heated to 65 °C and a solution of LiOH.H_2O (414 mg, 9.9 mmol, 10.0 equiv) in H_2O (0.1 M) was added. After 1h at 65 °C, the reaction was cooled to 23 °C and the mixture was concentrated under reduce pressure. The aqueous phase was washed with CH_2Cl_2 (3 × 5 mL). The remaining aqueous phase was acidified with HCl 1M until pH = 4 before extracting it further with EtOAc (3 × 10 mL). The combined organic phases were dried over Na_2SO_4, filtered and concentrated under reduce pressure to afford the expected compound as a white solid (207 mg, 0.9 mmol, 92%)

^{1}H NMR (400 MHz, $CDCl_3$) δ (ppm) = 7.58 – 7.54 (m, 1H), 7.51 (d, $^4J_{HH}$ = 0.9 Hz, 1H), 7.15 – 7.03 (m, 2H), 5.49 (dt, $^3J_{HH}$ = 6.1 Hz, $^4J_{HH}$ = 3.1 Hz, 2H), 5.41 (td, $^3J_{HH}$ = 6.4, 5.4 Hz, 1H), 4.71 (dt, $^3J_{HH}$ = 6.3 Hz, $^4J_{HH}$ = 3.1 Hz, 2H), 2.81 (d, $^4J_{HH}$ = 0.7 Hz, 3H).

^{13}C{^{1}H} NMR (101 MHz, $CDCl_3$) δ (ppm) = 207.8 (C_4), 166.3 (C_4), 139.0 (C_4), 129.0 (CH), 127.0 (C_4), 126.3 (C_4), 122.1 (C_4), 121.1 (CH), 121.0 (CH), 114.5 (CH), 90.0 (CH), 77.5 (CH_2), 44.1 (CH_2), 20.4 (CH_3).

HRMS (ESI+) *m/z*: calculated for $C_{13}H_{14}N$ [M–COOH+H]$^+$ 184.1121 found 184.1116.

IR (neat) ν (cm^{-1}): 2964, 1955, 1673, 1265, 1207, 897, 736.

mp: 118 °C.

7. Optimization of the Au(I)-catalyzed enantioselective hydrocarboxylation of allenes

General procedure VII (GP VII)

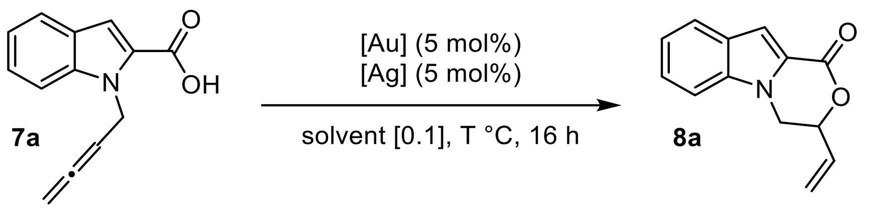

The procedure was adapted from the literature.[11]

Note: this reaction must be carried out in the dark (no light in the glovebox; Schlenk covered with an aluminum foil).

In a glovebox, a J-Young Schlenk tube was charged with the appropriate gold complex (7.5 µmol, 5 mol%) followed by the appropriate silver salt (7.5 µmol, 5 mol%) and the appropriate solvent (0.1 M). After stirring for 10 min. at 23 °C, the appropriate allene (32 mg, 0.15 mmol,1.0 equiv.) was added neat in one portion. The J-Young Schlenk tube was closed and taken out of the glovebox. The reaction was stirred for 16 hours at the appropriate temperature. The reaction mixture was concentrated under reduce pressure and purified by flash chromatography (cyclohexane/EtOAc, 5:2) to afford the desired compound. The enantiomeric ratio was determined by HPLC.

Reaction optimization

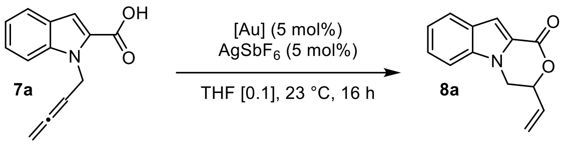

Complexes survey

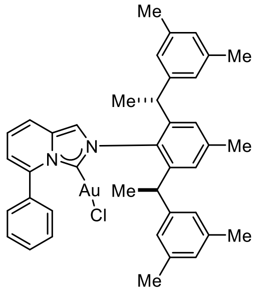

(*R*,*R*)-**6a**
10% conv.
nd

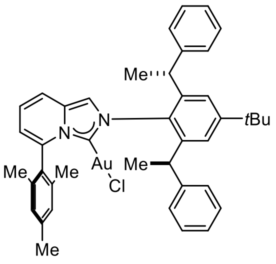

(*R*,*R*)-**6b**
29% yield
60:40 *er*

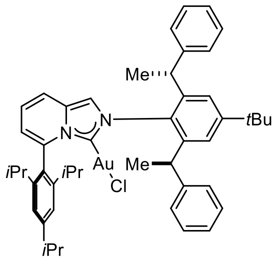

(*R*,*R*)-**6c**
62% yield
60:40 *er*

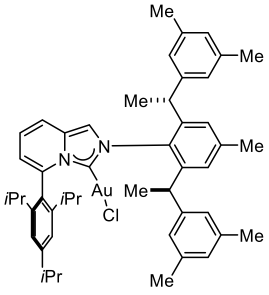

(*R*,*R*)-**6d**
69% yield
93:7 *er*

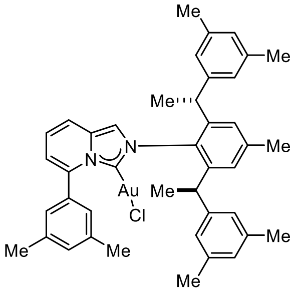

(*R*,*R*)-**6e**
85% yield
94.5:5.5 *er*

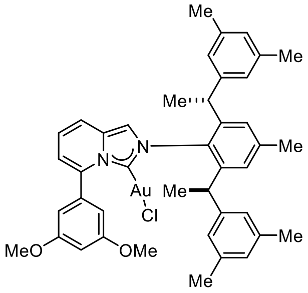

(*R*,*R*)-**6f**
85% yield
93:7 *er*

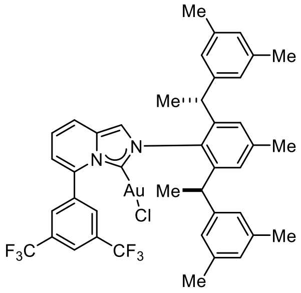

(*R*,*R*)-**6g**
53% yield
93:7 *er*

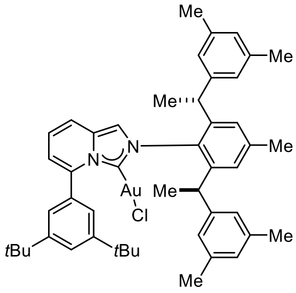

(*R*,*R*)-**6h**
41% yield
92:8 *er*

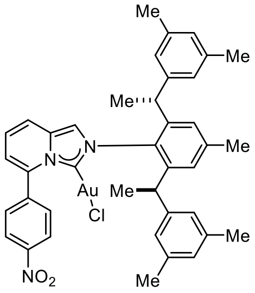

(*R*,*R*)-**6i**
97% yield
92:8 *er*

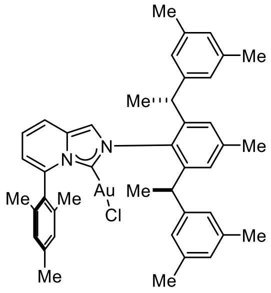

(*R*,*R*)-**6j**
81% yield
96:4 *er*

Figure S1. Au-catalyzed enantioselective hydrocarboxylation of allene (0.15 mmol). Isolated yield of **8a** after purification by flash chromatography. *er* determined by HPLC using a chiral stationary phase (AD, 2.5% *i*PrOH in *n*-hexane, 1mL/min.).

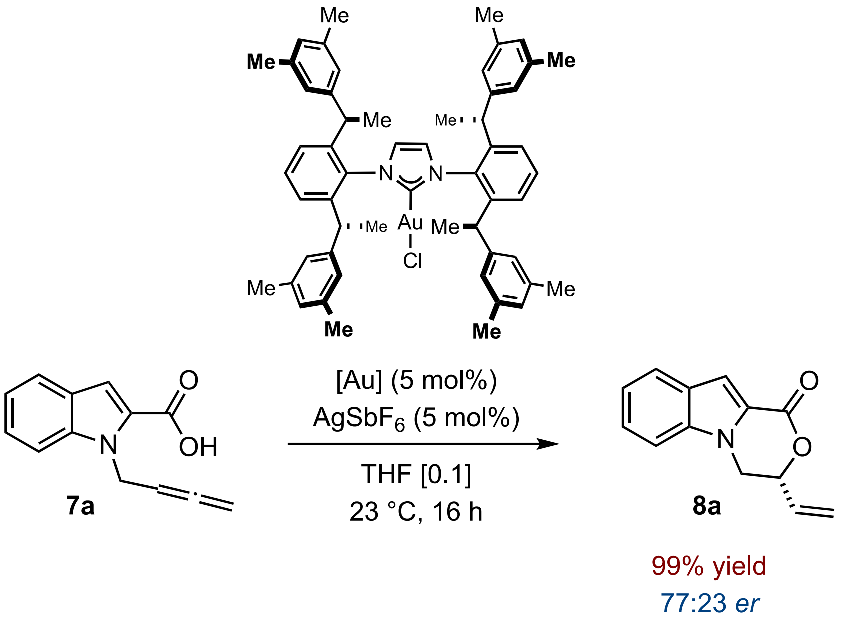

Figure S2. Au-catalyzed enantioselective hydrocarboxylation of allene (0.15 mmol) using a [(NHC)AuCl] complex supported by a C_2-symmetric ligand. Isolated yield of **8a** after purification by flash chromatography. *er* determined by HPLC using a chiral stationary phase (AD, 2.5% *i*PrOH in *n*-hexane, 1mL/min.).

Table S1. Screening of temperature, solvent and silver salt

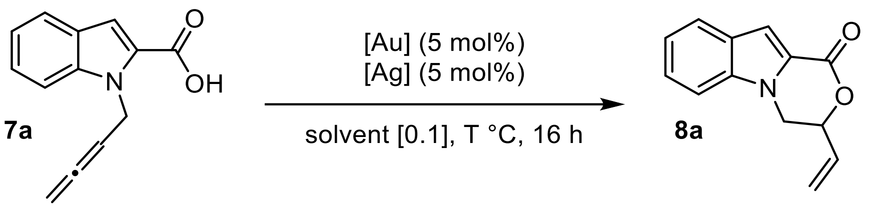

Entry	Temperature (°)	Solvent	Silver salt	Yield **8a** (%)[b]	*er*[c]
**1**	23	THF	$AgSbF_6$	81	96:4
**2**	0	THF	$AgSbF_6$	91	97:3
**3**	0	Et_2O	$AgSbF_6$	0	*nd*[d]
**4**	0	2-MeTHF	$AgSbF_6$	86	97:3
**5**	0	MTBE	$AgSbF_6$	0	/
**6**	23	1,4-dioxane	$AgSbF_6$	0	/
**7**	0	THF	$AgBF_4$	91	95:5
**8**	0	THF	AgOTf	81	95:5

[a] Reactions conditions: allene (0.15 mmol). [b] After purification by column chromatography. [c] Determined by HPLC using a chiral stationary phase (AD, 2.5% *i*PrOH in *n*-hexane, 1mL/min.). [d] Not determined.

8. Scope of the Au(I)-catalyzed enantioselective hydrocarboxylation of allenes

General procedure VIII (GP VIII)

The procedure was adapted from the literature.[11]

Note: this reaction must be carried out in the dark (no light in the glovebox; Schlenk covered with an aluminum foil).

In a glovebox, a J-Young Schlenk tube was charged with the gold complex (*R*,*R*)-**6j** (13 mg, 15 µmol, 5 mol%) followed by $AgSbF_6$ (5 mg, 15 µmol, 5 mol%) and THF (0.1 M). After stirring for 10 min at 23 °C, the appropriate allene (0.3 mmol,1.0 equiv.) was added neat in one portion. The J-Young Schlenk tube was closed and taken out of the glovebox. The reaction was stirred for 16 hours at 0 °C. The reaction mixture was concentrated under reduce pressure and purified by flash chromatography (cyclohexane/EtOAc, 5:2) to afford the desired compound. The enantiomeric ratio was determined by HPLC.

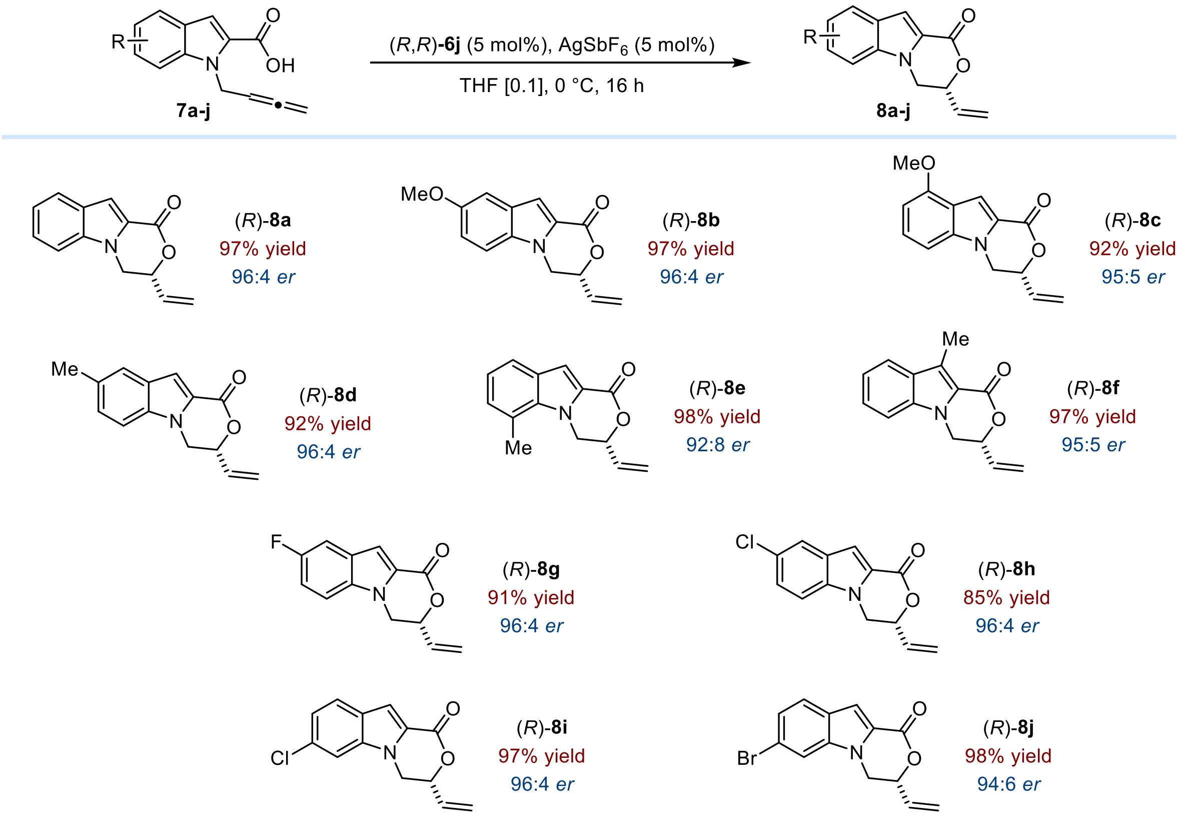

Figure S3. Enantioselective Au-catalyzed hydrocarboxylation of allene (0.3 mmol). Isolated yield after purification by flash chromatography. *er* determined by HPLC using a chiral stationary phase.

(*R*)-3-vinyl-3,4-dihydro-1*H*-[1,4]oxazino[4,3-*a*]indol-1-one ((*R*)-**8a**)

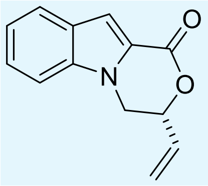

Following GP VIII using 1-(buta-2,3-dien-1-yl)-1*H*-indole-2-carboxylic acid (64 mg, 0.3 mmol, 1.0 equiv.), (*R*,*R*)-**6j** (13 mg, 15 µmol, 5 mol%) and $AgSbF_6$ (5 mg, 15 µmol, 5 mol%) in THF (0.1 M). Purification by Flash chromatography (cyclohexane/EtOAc, 5:2) afforded (*R*)-**8a** as a white solid in pure form (62 mg, 0.29 mmol, 97%).

The spectroscopic data were in agreement with those reported in the literature.[11]

HPLC: AD, 2.5% *i*PrOH in *n*-hexane, 1 mL/min. 96:4 *er.*

$[\alpha]^{20}_D$ = –41.9 (c = 0.4 in CH_2Cl_2).

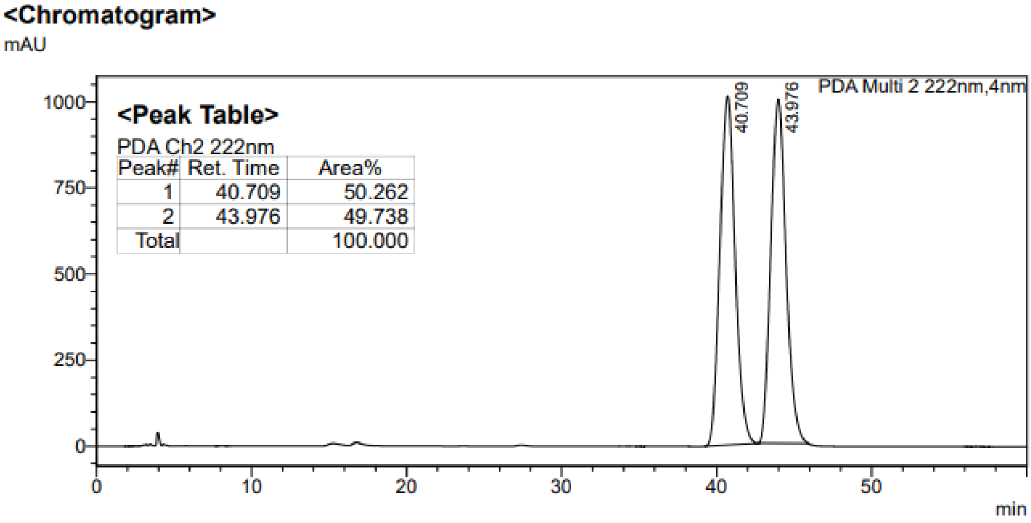

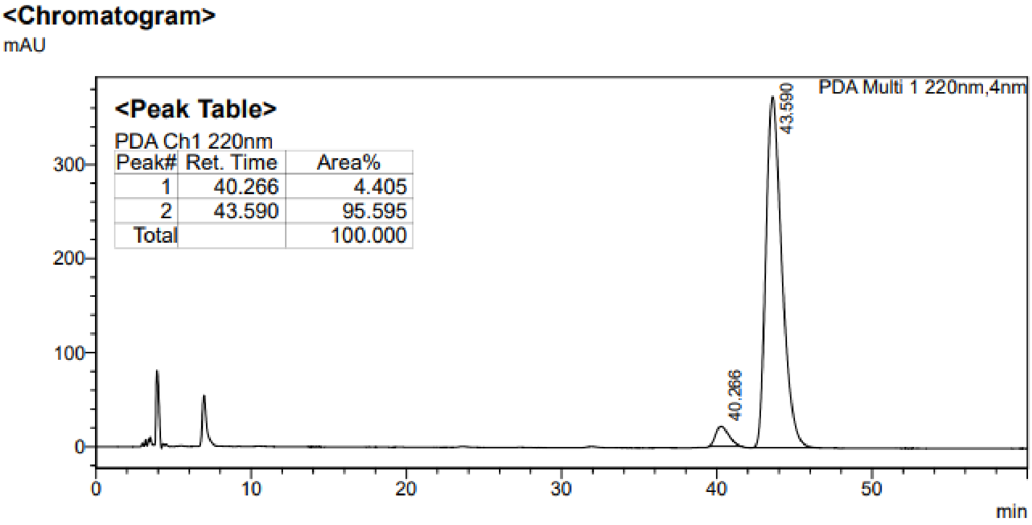

Figure S4. HPLC traces for compound (*R*)-**8a**.

(*R*)- 8-methoxy-3-vinyl-3,4-dihydro-1*H*-[1,4]oxazino[4,3-*a*]indol-1-one ((*R*)-**8b**)

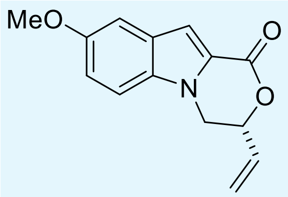

Following GP VIII using 1-(buta-2,3-dien-1-yl)-5-methoxy-1*H*-indole-2-carboxylic acid (73 mg, 0.3 mmol, 1.0 equiv.), (*R*,*R*)-**6j** (13 mg, 15 µmol, 5 mol%) and $AgSbF_6$ (5 mg, 15 µmol, 5 mol%) in THF [0.1 M]. Purification by Flash chromatography (cyclohexane/EtOAc, 5:2) afforded (*R*)-**8b** as a white solid in pure form (71 mg, 0.29 mmol, 97%.

The spectroscopic data were in agreement with those reported in the literature.[11]

HPLC: AD, 2.5% *i*PrOH in *n*-hexane, 1 mL/min. 96:4% *er.*

$[\alpha]^{20}_D$ = –41.9 (c = 0.5 in CH_2Cl_2).

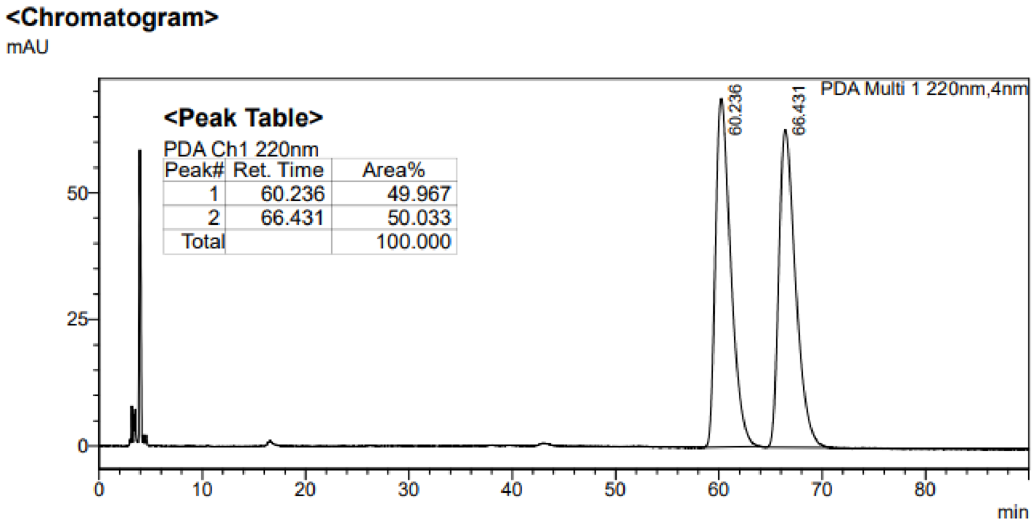

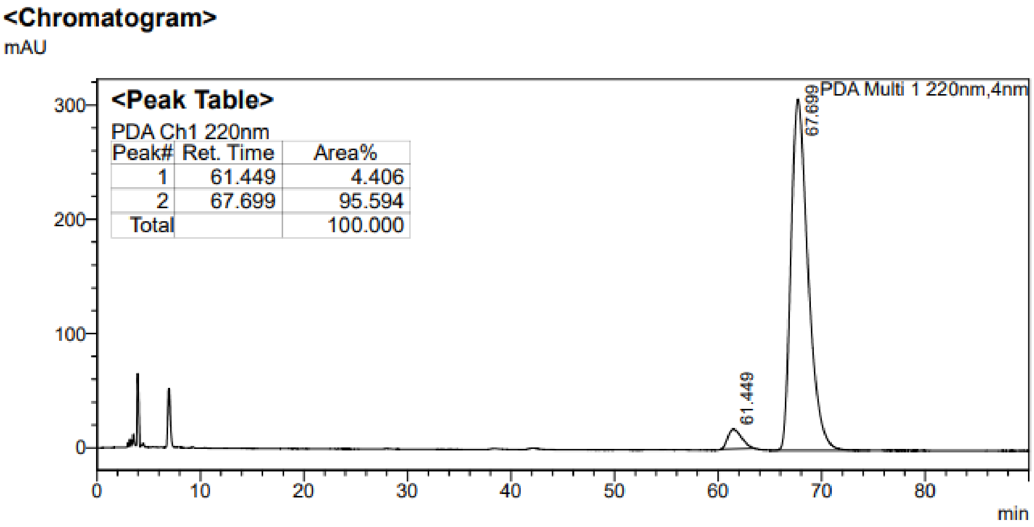

Figure S5. HPLC traces for compound (*R*)-**8b.**

(*R*)- 9-methoxy-3-vinyl-3,4-dihydro-1*H*-[1,4]oxazino[4,3-*a*]indol-1-one ((*R*)-**8c**)

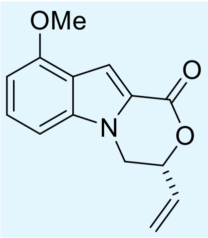

Following GP VIII using 1-(buta-2,3-dien-1-yl)-4-methoxy-1*H*-indole-2-carboxylic acid (73 mg, 0.3 mmol, 1.0 equiv.), (*R*,*R*)-**6j** (13 mg, 15 µmol, 5 mol%) and $AgSbF_6$ (5 mg, 15 µmol, 5 mol%) in THF [0.1 M]. Purification by Flash chromatography (cyclohexane/EtOAc, 5:2) afforded (*R*)-**8c** as a white solid in pure form (67 mg, 0.28 mmol, 92%).

The spectroscopic data were in agreement with the one reported in the literature.[11]

HPLC: OD, 20% *i*PrOH in *n*-hexane, 1 mL/min. 95:5 *er.*

$[\alpha]^{20}_D$ = –40.6 (*c* = 0.4 in CH_2Cl_2).

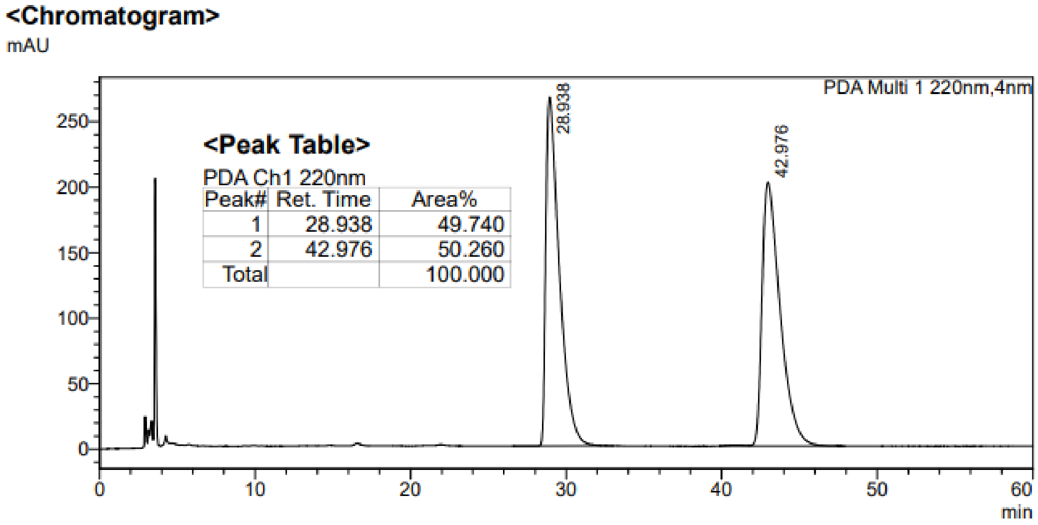

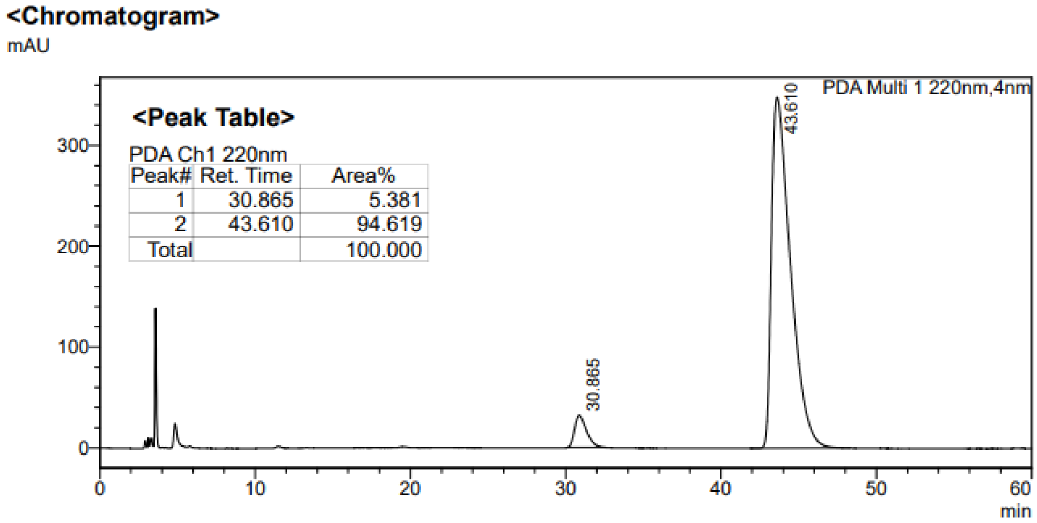

Figure S6. HPLC traces for compound (*R*)-**8c.**

(*R*)- 8-methyl-3-vinyl-3,4-dihydro-1*H*-[1,4]oxazino[4,3-*a*]indol-1-one ((*R*)-**8d**)

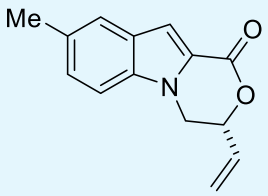

Following GP VIII using 1-(buta-2,3-dien-1-yl)-5-methyl-1*H*-indole-2-carboxylic acid (68 mg, 0.3 mmol, 1.0 equiv.), (*R*,*R*)-**6j** (13 mg, 15 µmol, 5 mol%) and $AgSbF_6$ (5 mg, 15 µmol, 5 mol%) in THF [0.1 M]. Purification by Flash chromatography (cyclohexane/EtOAc, 5:2) afforded (*R*)-**8d** as a white solid in pure form (63 mg, 0.28 mmol, 92%).

The spectroscopic data were in agreement with the one reported in the literature.[11]

HPLC: AD, 2.5% *i*PrOH in *n*-hexane, 1 mL/min. 96:4 *er.*

[α]$^{20}_{D}$ = –44.9 (*c* = 0.5 in CH_2Cl_2).

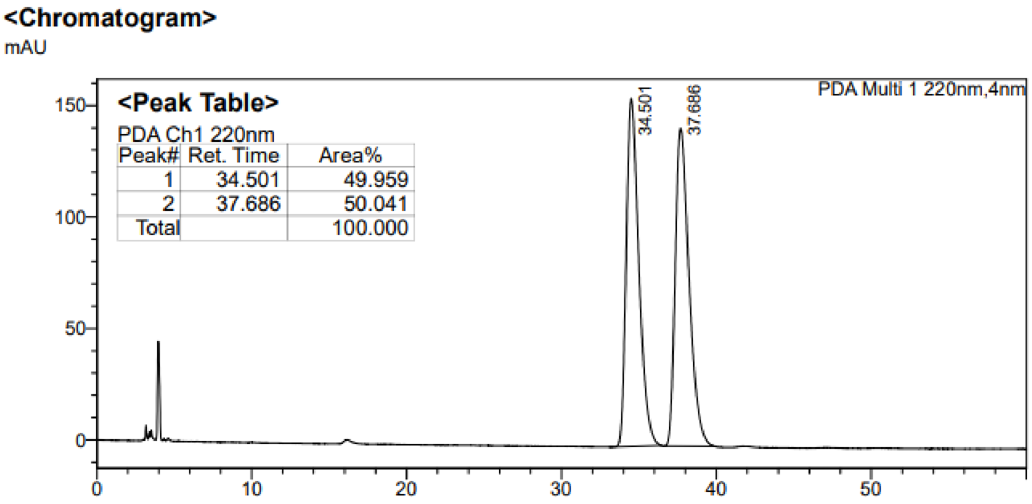

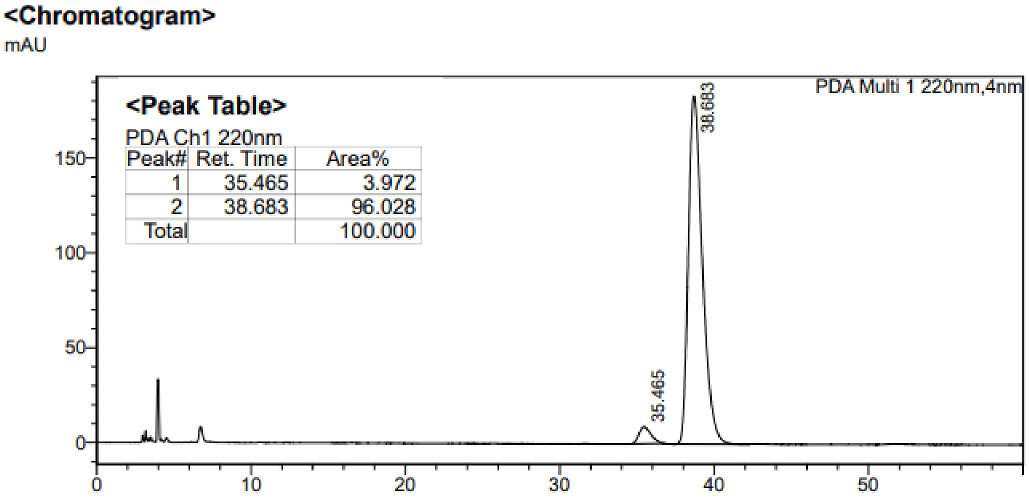

Figure S7. HPLC traces for compound (*R*)-**8d**.

(*R*)- 6-methyl-3-vinyl-3,4-dihydro-1*H*-[1,4]oxazino[4,3-*a*]indol-1-one ((*R*)-**8e**)

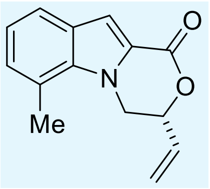

Following GP VIII using 1-(buta-2,3-dien-1-yl)-7-methyl-1*H*-indole-2-carboxylic acid (68 mg, 0.3 mol, 1.0 equiv.), (*R*,*R*)-**6j** (13 mg, 15 µmol, 5 mol%) and $AgSbF_6$ (5 mg, 15 µmol, 5 mol%) in THF [0.1 M]. Purification by Flash chromatography (cyclohexane/EtOAc 5:2) afforded (*R*)-**8e** as a white solid in pure form (67 mg, 0.29 mmol, 98%).

^{1}H NMR (400 MHz, $CDCl_3$) δ (ppm) = 7.57 (ddd, $^3J_{HH}$ = 7.7 Hz, $^4J_{HH}$ = 1.6, 0.7 Hz, 1H), 7.47 (s, 1H), 7.11 (ddt, $^3J_{HH}$ = 6.2 Hz, $^4J_{HH}$ = 1.6, 0.8 Hz, 1H), 7.10 – 7.05 (m, 1H), 6.04 (ddd, $^3J_{HH}$ = 17.2, 10.7, 5.8 Hz, 1H), 5.63 (ddd, $^3J_{HH}$ = 17.2 Hz, $^4J_{HH}$ = 1.5, 0.8 Hz, 1H), 5.47 (ddd, $^3J_{HH}$ = 10.7 Hz, $^4J_{HH}$ = 1.3, 0.8 Hz, 1H), 5.26 (dddt, $^3J_{HH}$ = 9.2, 6.0, Hz, $^4J_{HH}$ = 3.4, 1.4 Hz, 1H), 4.84 (dd, $^3J_{HH}$ = 12.9 Hz, $^4J_{HH}$ = 3.4 Hz, 1H), 4.42 (dd, $^3J_{HH}$ = 12.9, 9.3 Hz, 1H), 2.73 (d, $^4J_{HH}$ = 0.7 Hz, 3H).

^{13}C{^{1}H} NMR (101 MHz, $CDCl_3$) δ (ppm) = 159.5 (C_4), 136.1 (C_4), 132.0 (CH), 128.5 (CH), 127.6 (C_4), 123.8 (C_4), 121.5 (C_4), 121.5 (CH), 121.3 (CH), 120.4 (CH_2), 111.4 (CH), 77.7 (CH), 47.2 (CH_2), 19.7 (CH_3).

HRMS (ESI+) *m/z*: calculated for $C_{14}H_{14}NO_2$ $[M+H]^+$ 228.1019 found 228.1018.

IR (neat) ν (cm^{-1}): 2921, 1713, 1540, 1356, 1201, 1088, 948, 744.

mp: 146 °C.

HPLC: OD, 10% *i*PrOH in *n*-hexane, 1 mL/min. 92:8 *er.*

$[\alpha]^{20}_D$ = –13.4 (*c* = 0.5 in CH_2Cl_2).

<Chromatogram>

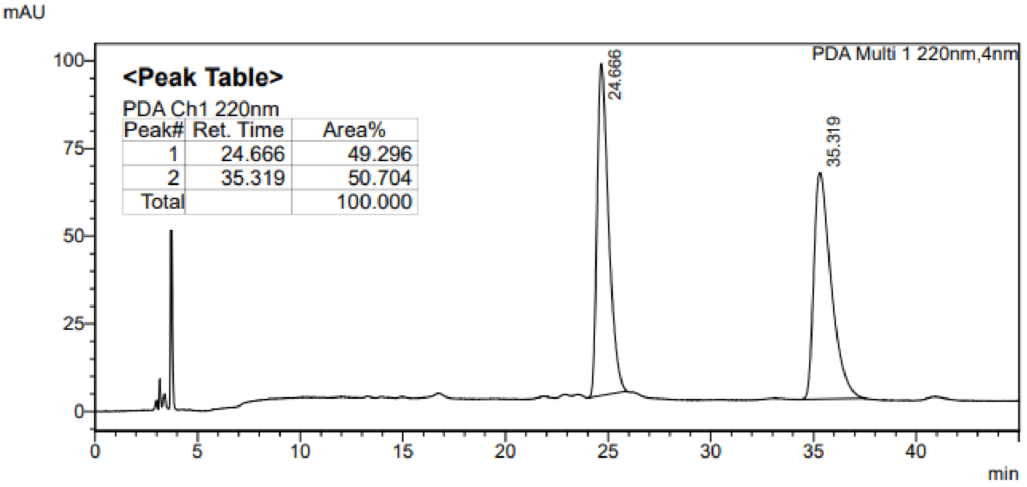

<Peak Table>

PDA Ch1 220nm

Peak#	Ret. Time	Area%
1	24.666	49.296
2	35.319	50.704
Total		100.000

<Chromatogram>

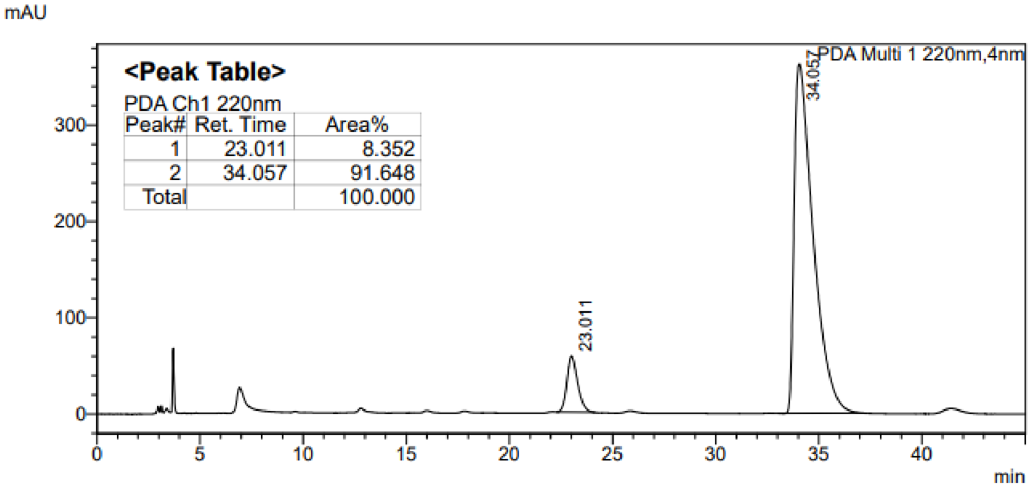

<Peak Table>

PDA Ch1 220nm

Peak#	Ret. Time	Area%
1	23.011	8.352
2	34.057	91.648
Total		100.000

Figure S8. HPLC traces for compound (*R*)-**8e.**

(*R*)- 10-methyl-3-vinyl-3,4-dihydro-1*H*-[1,4]oxazino[4,3-a]indol-1-one ((*R*)-**8f**)

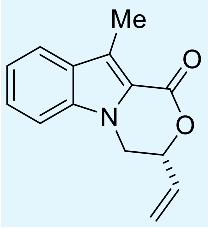

Following GP VIII using 1-(buta-2,3-dien-1-yl)-3-methyl-1*H*-indole-2-carboxylic acid (68 mg, 0.3 mol, 1.0 equiv.), (*R*,*R*)-**6j** (13 mg, 15 µmol, 5 mol%) and $AgSbF_6$ (5 mg, 15 µmol, 5 mol%) in THF [0.1 M]. Purification by Flash chromatography (cyclohexane/EtOAc, 5:2) afforded (*R*)-**8f** in pure form (66 mg, 0.29 mmol, 97%) as a white solid.

The spectroscopic data were in agreement with the one reported in the literature.[11]

HPLC: OD, 2.5% *i*PrOH in *n*-hexane, 1 mL/min. 95:5 *er.*

$[\alpha]^{20}_D$ = –51.8 (*c* = 0.5 in CH_2Cl_2).

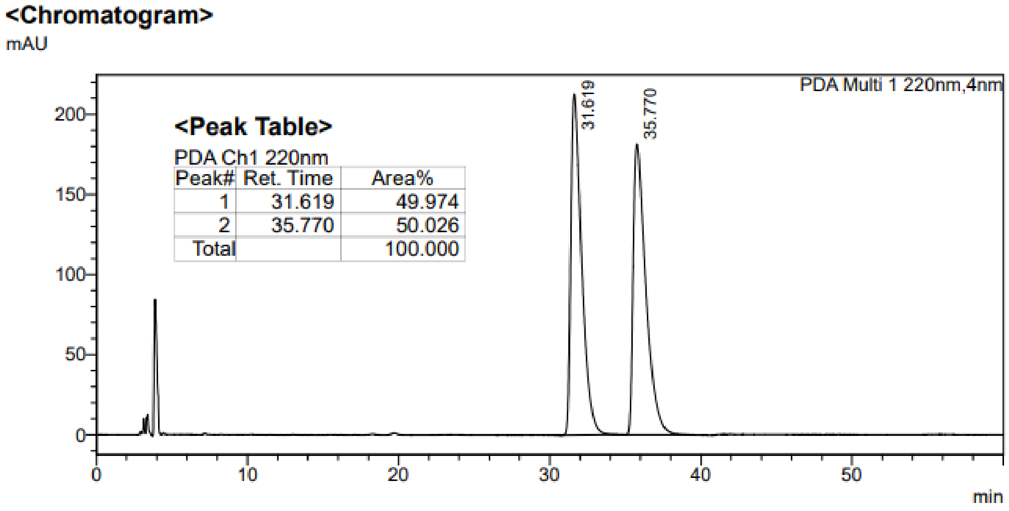

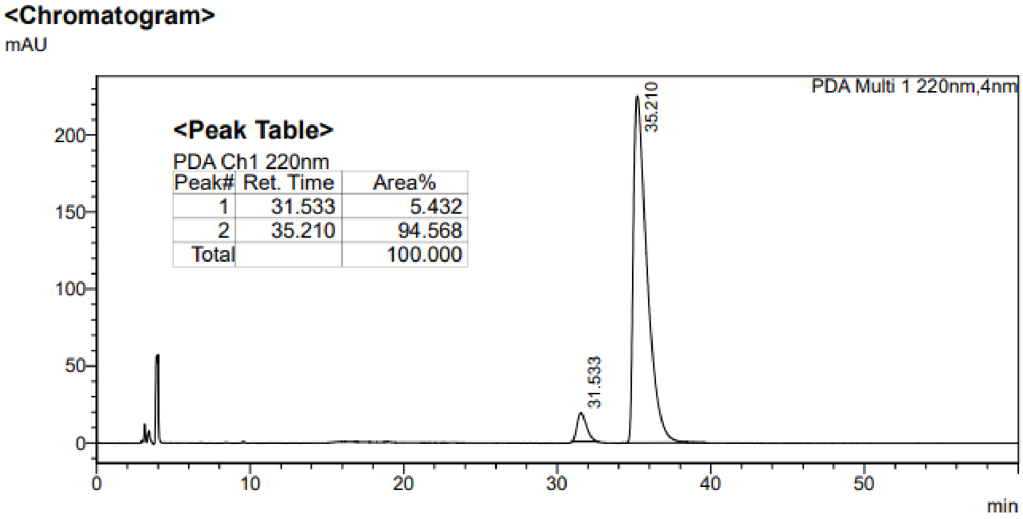

Figure S9. HPLC traces for compound (*R*)-**8f.**

(*R*)- 8-fluoro-3-vinyl-3,4-dihydro-1*H*-[1,4]oxazino[4,3-*a*]indol-1-one ((*R*)-**8g**)

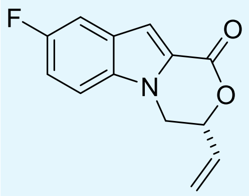

Following GP VIII using 1-(buta-2,3-dien-1-yl)-5-(trifluoromethyl)-1*H*-indole-2-carboxylic acid (69 mg, 0.3 mmol, 1.0 equiv.), (*R*,*R*)-**6j** (13 mg, 15 µmol, 5 mol%) and $AgSbF_6$ (5 mg, 15 µmol, 5 mol%) in THF [0.1 M]. Purification by Flash chromatography (cyclohexane/EtOAc 5:2) afforded (*R*)-**8g** as a white solid in pure form (63 mg, 0.27 mmol, 91%).

The spectroscopic data were in agreement with the one reported in the literature.[11]

HPLC: AD, 2.5% *i*PrOH in *n*-hexane, 1 mL/min. 96:4 *er.*

[α]^{20}$_D$ = –43.7 (*c* = 0.4 in CH_2Cl_2).

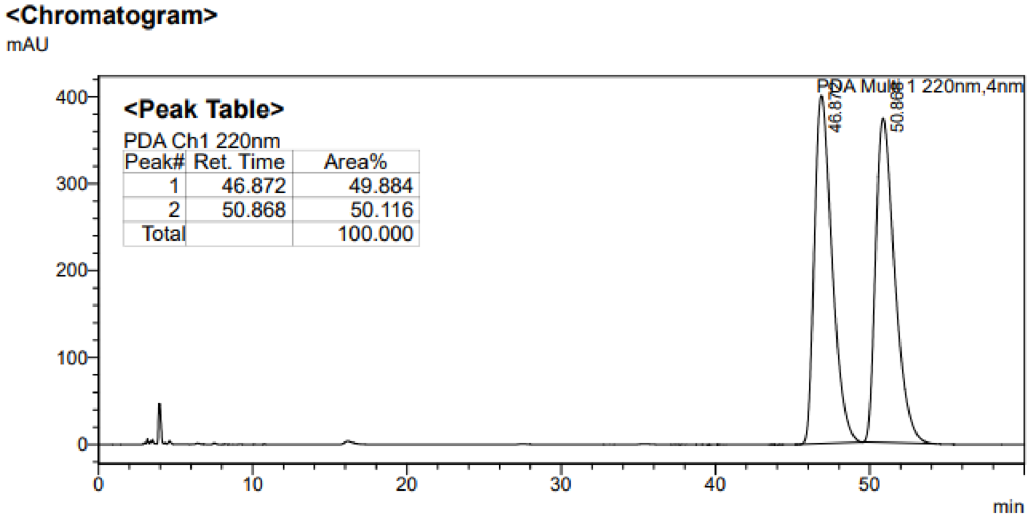

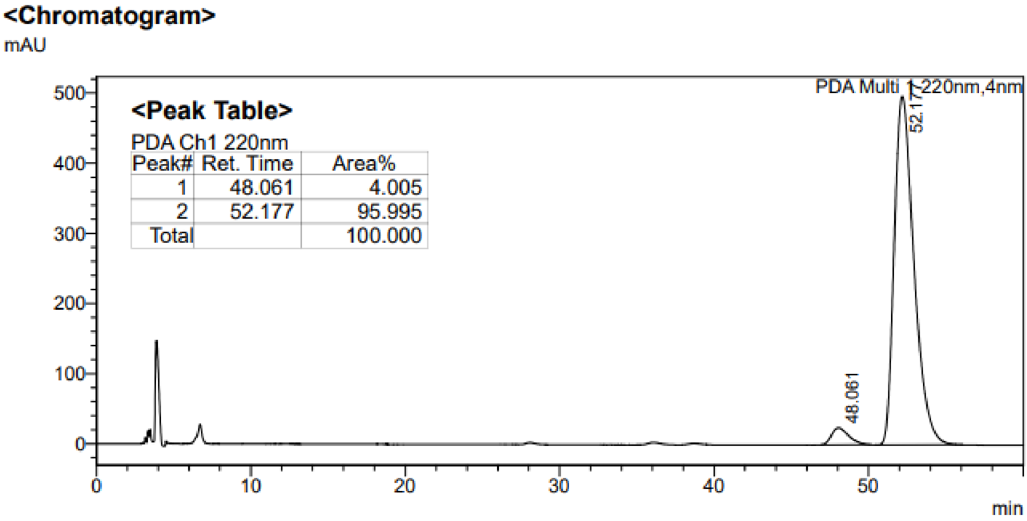

Figure S10. HPLC traces for compound (*R*)-**8g.**

(*R*)- 8-chloro-3-vinyl-3,4-dihydro-1*H*-[1,4]oxazino[4,3-*a*]indol-1-one ((*R*)-**8h**)

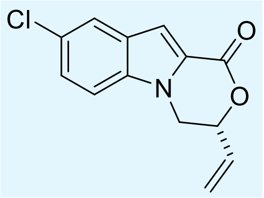

Following GP VIII using 1-(buta-2,3-dien-1-yl)-5-chloro-1*H*-indole-2-carboxylic acid (69 mg, 0.3 mmol, 1.0 equiv.), (*R*,*R*)-**6j** (13 mg, 15 µmol, 5 mol%) and $AgSbF_6$ (5 mg, 15 µmol, 5 mol%) in THF [0.1 M]. Purification by Flash chromatography (cyclohexane/EtOAc 5:2) afforded (*R*)-**8h** as a white solid in pure form (63 mg, 0.26 mmol, 85%).

The spectroscopic data were in agreement with the one reported in the literature.[11]

HPLC: OD, 10% *i*PrOH in *n*-hexane, 1 mL/min. 96:4 *er*.

$[\alpha]^{20}_D$ = –41.5 (c = 0.4 in CH_2Cl_2).

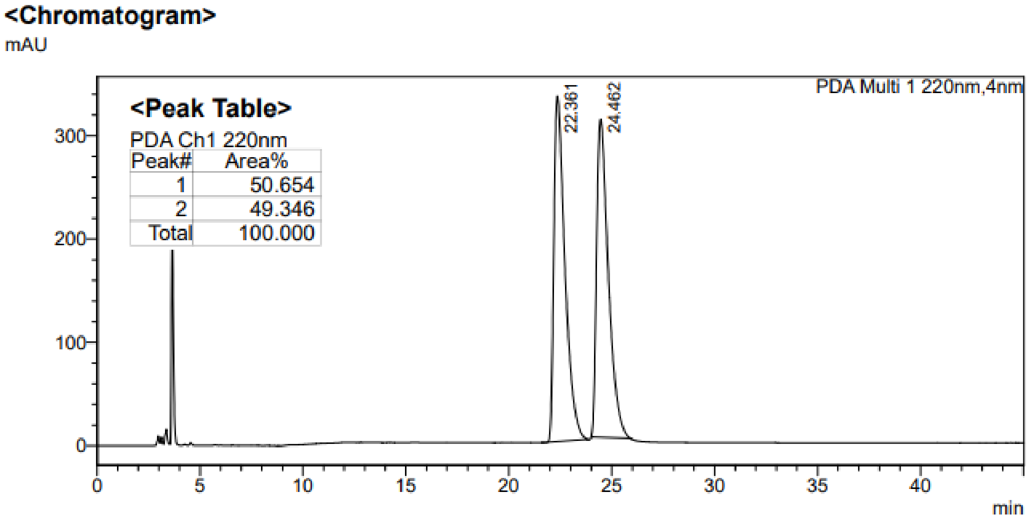

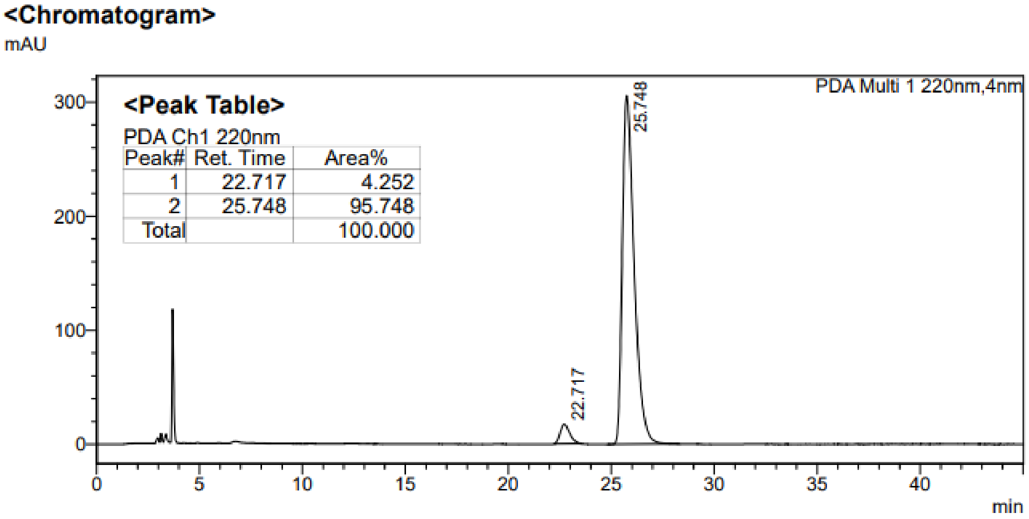

Figure S11. HPLC traces for compound (*R*)-**8h.**

(*R*)- 7-chloro-3-vinyl-3,4-dihydro-1*H*-[1,4]oxazino[4,3-*a*]indol-1-one ((*R*)-**8i**)

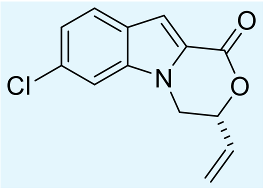

Following GP VIII using 1-(buta-2,3-dien-1-yl)-6-chloro-1*H*-indole-2-carboxylic acid (69 mg, 0.3 mmol, 1.0 equiv.), (*R*,*R*)-**6j** (13 mg, 15 µmol, 5 mol%) and $AgSbF_6$ (5 mg, 15 µmol, 5 mol%) in THF [0.1 M]. Purification by Flash chromatography (cyclohexane/EtOAc 5:2) afforded (*R*)-**8i** as a white solid in pure form (72 mg, 0.29 mmol, 97%).

The spectroscopic data were in agreement with the one reported in the literature.[11]

HPLC: IC, 10% *i*PrOH in *n*-hexane, 1 mL/min. 96:4 *er*.

$[\alpha]^{20}_D$ = –21.5 (c = 0.4 in CH_2Cl_2).

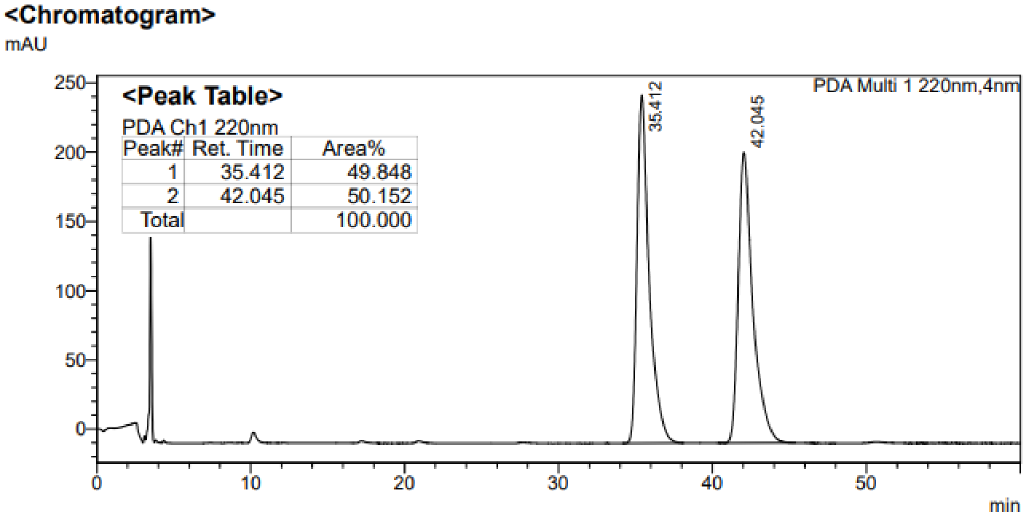

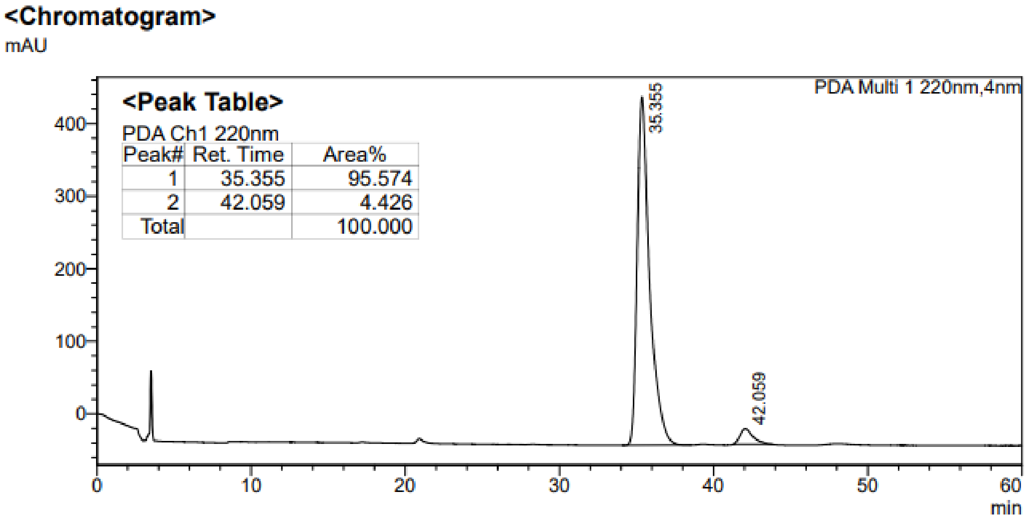

Figure S12. HPLC traces for compound (*R*)-**8i.**

(*R*)- 7-bromo-3-vinyl-3,4-dihydro-1*H*-[1,4]oxazino[4,3-*a*]indol-1-one ((*R*)-**8j**)

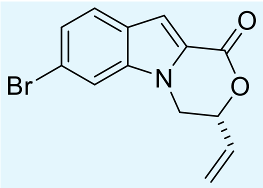

Following GP VIII using 1-(buta-2,3-dien-1-yl)-6-bromo-1*H*-indole-2-carboxylic acid (88 mg, 0.3 mmol, 1.0 equiv.), (*R*,*R*)-**6j** (13 mg, 15 µmol, 5 mol%) and $AgSbF_6$ (5 mg, 15 µmol, 5 mol%) in THF [0.1 M]. Purification by Flash chromatography (cyclohexane/EtOAc 5:2) afforded (*R*)-**8j** as a white solid in pure form (86 mg, 0.29 mmol, 98%).

The spectroscopic data were in agreement with the one reported in the literature.[11]

HPLC: IC, 10% *i*PrOH in *n*-hexane, 1 mL/min. 94:6 *er.*

$[\alpha]^{20}_D$ = –21.5 (*c* = 0.4 in CH_2Cl_2).

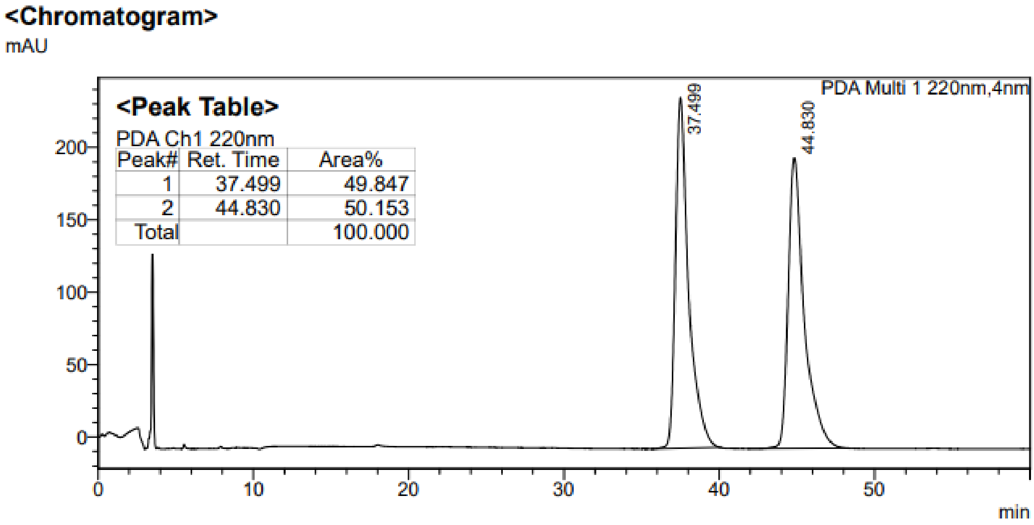

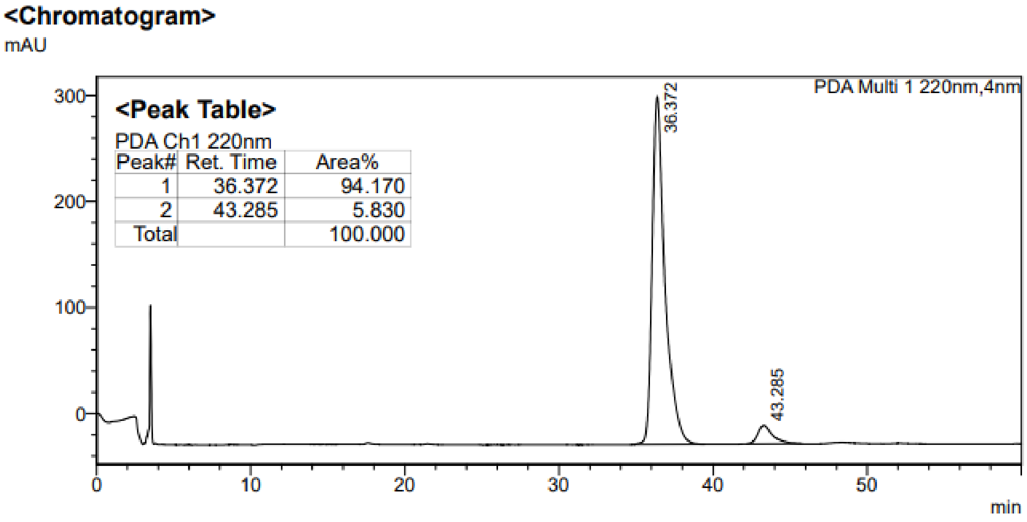

Figure S13. HPLC traces for compound (*R*)-**8j.**

10. X-Ray Analyses

Table S2. Crystal structure and data refinement for (*R*,*R*)-**6c**

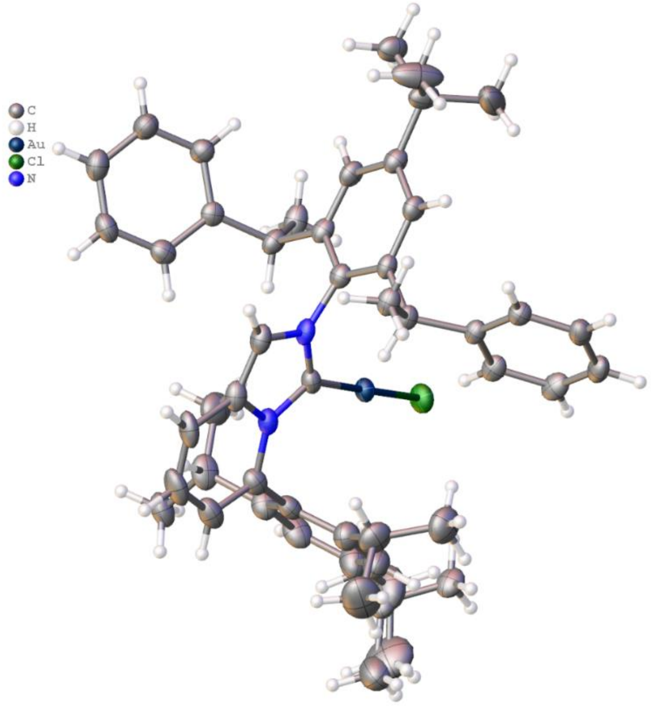

View of the asymmetric unit with displacement parameters at 50 percent probability level.

Comments:

One isopropyl is disordered and was refimed using 2 components. The following restraints were used:

SADI C15B C14 C15A C14

SADI C17B C15B C17A C15A C16A C15A C18B C15B

RIGU C14 C15B C18B C17B

RIGU C14 C15A C17A C16A

SIMU 0.04 0.08 1 C15A C15B C16A C18B C17A C17B

There is a large hole in the structure (20 % of the volume). A mask was calculated and shows that it accounts for 276 electrons. However, no predominant feature was visible in the difference Fourier map in this hole and due to the already low R values, the squeeze/bypass procedure, which only slightly inproved the model, was not applied.

CCDC Number	2471938
Empirical formula	$C_{48}H_{56}AuClN_2O_{0.17}$
Formula weight	896.03
Temperature/K	120.01(12)
Crystal system	hexagonal
Space group	$P6_5$
a/Å	27.67030(5)
b/Å	27.67030(5)
c/Å	11.31586(2)
α/°	90
β/°	90
γ/°	120
Volume/Å^3	7503.18(3)
Z	6
ρ_{calc}g/cm^3	1.190
μ/mm^{-1}	6.228
F(000)	2732.0
Crystal size/mm^3	0.274 × 0.03 × 0.015
Radiation	Cu Kα (λ = 1.54184)
2Θ range for data collection/°	7.378 to 152.148
Index ranges	$-34 \le h \le 34, -34 \le k \le 34, -8 \le l \le 13$
Reflections collected	280946
Independent reflections	8871 [R_{int} = 0.0477, R_{sigma} = 0.0116]
Data/restraints/parameters	8871/56/507
Goodness-of-fit on F^2	1.103
Final R indexes [I>=2σ (I)]	R_1 = 0.0280, wR_2 = 0.0835
Final R indexes [all data]	R_1 = 0.0284, wR_2 = 0.0838
Largest diff. peak/hole / e Å^{-3}	0.75/-1.60
Flack parameter	-0.015(8)

Table S3. Crystal structure and data refinement for (*R*,*R*)-**6e**

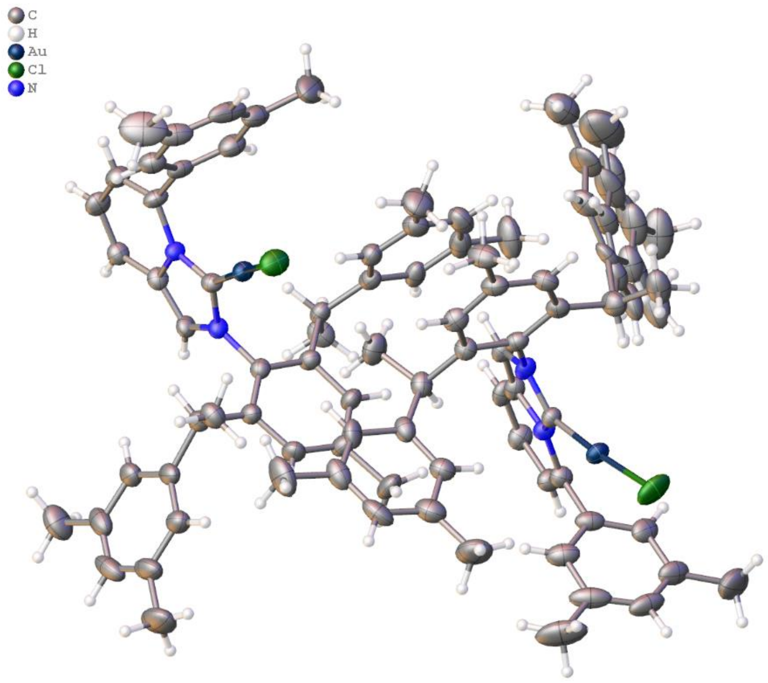

View of the asymmetric unit with displacement parameters at 50 percent probability level.

Comments

One part of the molecule is disordered and was refined using two components. The following restraints were applied:

SADI C38 C39 C39B C38B C43B C38B C43 C38 C43 C42 C41 C42 C40 C41 C42B C43B = C42B C41B C40 C39 C40B C41B C39B C40B

SADI C42B C44B C44 C42 C45 C40 C40B C45B

RIGU C40 C42 C45 C38 C41 C43 C44 C39 C36

RIGU C38B C42B C45B C43B C41B C39B C44B C40B C36

The data was squeezed. The agreement factors before using the squeeze/bypass procedure were R = 0.04 and wR = 0.12, with a Flack parameter of –0.020(11). The squeezed part is probably pentane (chain partly visible in the difference Fourier map before squeezing). In the solvent mask 284 electrons were found in a volume of 1552 Å^3 in 2 voids per unit cell. This corresponds to about 7 pentane molecules per unit cell.

CCDC Number	2471929
Empirical formula	$C_{42}H_{44}AuClN_2$
Formula weight	809.21
Temperature/K	120.00(10)
Crystal system	orthorhombic
Space group	$P2_12_12$
a/Å	28.88633(10)
b/Å	24.86992(8)
c/Å	11.40629(4)
α/°	90
β/°	90
γ/°	90
Volume/Å^3	8194.29(5)
Z	8
ρ_{calc}g/cm^3	1.312
μ/mm^{-1}	7.546
F(000)	3248.0
Crystal size/mm^3	0.196 × 0.155 × 0.004
Radiation	Cu Kα (λ = 1.54184)
2Θ range for data collection/°	4.688 to 149.666
Index ranges	-36 ≤ h ≤ 36, -30 ≤ k ≤ 31, -14 ≤ l ≤ 12
Reflections collected	246017
Independent reflections	16735 [R_{int} = 0.0481, R_{sigma} = 0.0177]
Data/restraints/parameters	16735/198/922
Goodness-of-fit on F^2	1.044
Final R indexes [I>=2σ (I)]	R_1 = 0.0283, wR_2 = 0.0749
Final R indexes [all data]	R_1 = 0.0293, wR_2 = 0.0753
Largest diff. peak/hole / e Å^{-3}	0.93/-1.28
Flack parameter	0.003(7)

Table S4. Crystal structure and data refinement for (*R*,*R*)-**6f**

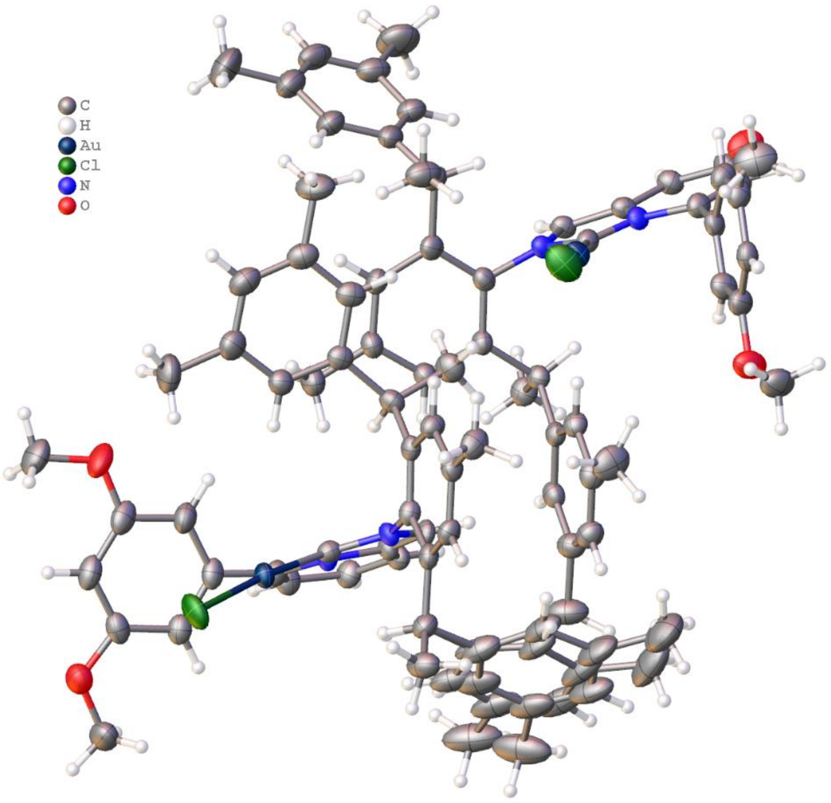

View of the asymmetric unit with displacement parameters at 50 percent probability level.

CCDC Number	2471940
Empirical formula	$C_{42}H_{44}AuClN_2O_2$
Formula weight	841.21
Temperature/K	120.00(10)
Crystal system	orthorhombic
Space group	$P2_12_12$
a/Å	28.76056(12)
b/Å	25.09307(9)
c/Å	11.48106(5)
α/°	90
β/°	90
γ/°	90
Volume/Å^3	8285.77(6)
Z	8
ρ_{calc}g/cm^3	1.349
μ/mm^{-1}	7.521
F(000)	3376.0
Crystal size/mm^3	0.43 × 0.125 × 0.035
Radiation	Cu Kα (λ = 1.54184)
2Θ range for data collection/°	4.674 to 152.892
Index ranges	$-35 \le h \le 36, -31 \le k \le 31, -14 \le l \le 9$
Reflections collected	228685
Independent reflections	17120 [R_{int} = 0.0438, R_{sigma} = 0.0182]
Data/restraints/parameters	17120/427/953
Goodness-of-fit on F^2	1.062
Final R indexes [I>=2σ (I)]	R_1 = 0.0244, wR_2 = 0.0616
Final R indexes [all data]	R_1 = 0.0267, wR_2 = 0.0623
Largest diff. peak/hole / e Å^{-3}	0.90/-0.80
Flack parameter	-0.021(6)

Table S5. Crystal structure and data refinement for (*R*)-**8j**

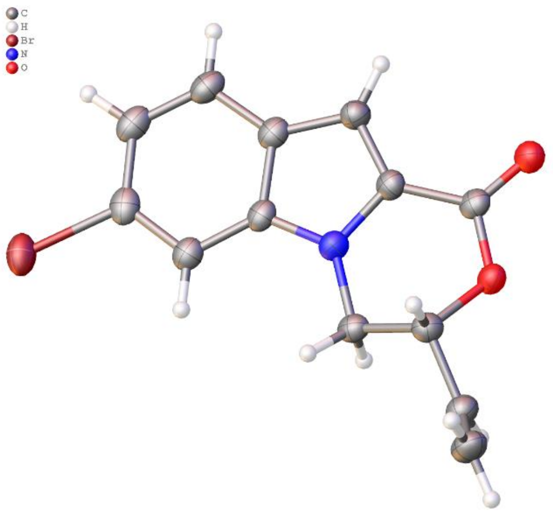

View of the asymmetric unit with displacement parameters at 50 percent probability level.

Comments

One part of the ligand was disordered and refined using two components. The following restraints/constraints were used:

SADI C8 C10B C8 C10A

SADI C16A C15A C16B C15B C13A C12A C13B C12B

SADI C11B C10B C11A C10A C12A C11A C12B C11B C14B C12B C14A C12A C15B C14B = C15A C14A C17A C15A C17B C15B C17B C10B C17A C10A

RIGU C13B C12B C11B C10B C17B C15B C16B C14B

RIGU C13A C12A C11A C10A C17A C15A C16A C14A

SIMU 0.04 0.08 1 C10B C17B C11B C14B C15B C12B C16B C13B C10A C17A C15A = C16A C14A C12A C11A C13A

EADP C11A C11B

Some very disordered pentane molecules were also present and the squeeze/bypass method was used to take care of this. A solvent mask was calculated and 246 electrons were found in a volume of 1512 Å^3. This is consistent with the presence of 6 pentane molecules per Unit Cell (according to the electron count 4 in the largest hole and 2 in the smallest hole), which accounts for 252 electrons per unit cell.

CCDC Number	2471941
Empirical formula	$C_{13}H_{10}BrNO_2$
Formula weight	292.13
Temperature/K	120.00(10)
Crystal system	orthorhombic
Space group	$C222_1$
a/Å	7.37270(10)
b/Å	18.6339(3)
c/Å	17.0634(2)
α/°	90
β/°	90
γ/°	90
Volume/$Å^3$	2344.21(6)
Z	8
$ρ_{calc}g/cm^3$	1.655
$μ/mm^{-1}$	4.695
F(000)	1168.0
Crystal size/mm^3	0.448 × 0.29 × 0.024
Radiation	Cu Kα (λ = 1.54184)
2Θ range for data collection/°	9.492 to 149.6
Index ranges	-6 ≤ h ≤ 8, -23 ≤ k ≤ 23, -21 ≤ l ≤ 21
Reflections collected	34796
Independent reflections	2385 [R_{int} = 0.0807, R_{sigma} = 0.0269]
Data/restraints/parameters	2385/0/155
Goodness-of-fit on F^2	1.101
Final R indexes [I>=2σ (I)]	R_1 = 0.0321, wR_2 = 0.0884
Final R indexes [all data]	R_1 = 0.0340, wR_2 = 0.0899
Largest diff. peak/hole / e $Å^{-3}$	0.56/-0.87
Flack parameter	-0.06(3)

11. Supplementary experiments

2-vinyl-4*H*-benzo[d][1,3]dioxin-4-one (9)

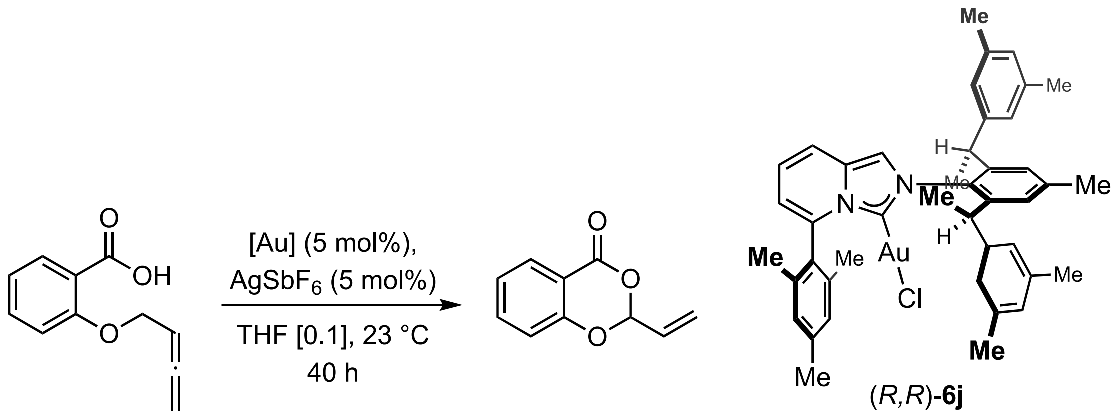

(*R,R*)-**6j**
23% yield, 64:36 *er*

The reaction was performed following the protocol described in the literature.[11]

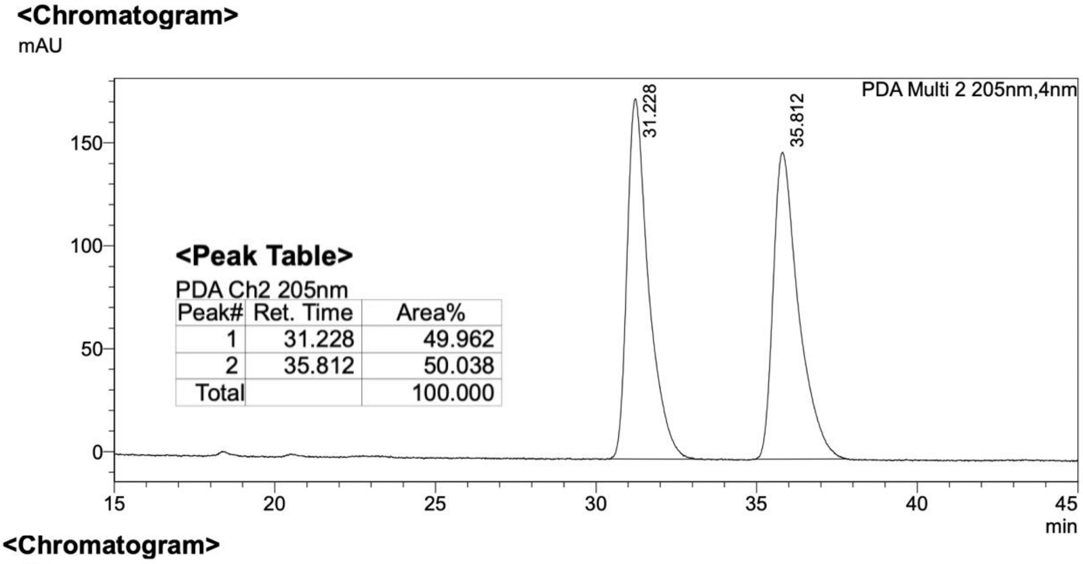

Peak#	Ret. Time	Area%
1	31.228	49.962
2	35.812	50.038
Total		100.000

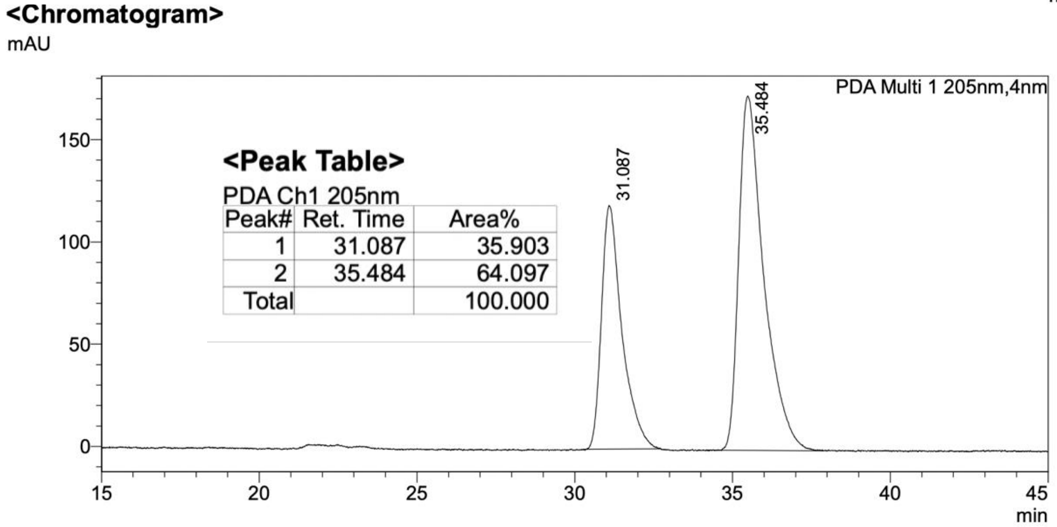

Peak#	Ret. Time	Area%
1	31.087	35.903
2	35.484	64.097
Total		100.000

Figure S14: HPLC traces for compound **9** (IC, 5% *i*PrOH in *n*-hexanes, 1 mL/min).

(3-methylcyclobut-1-ene-1,3-diyl)dibenzene (10)

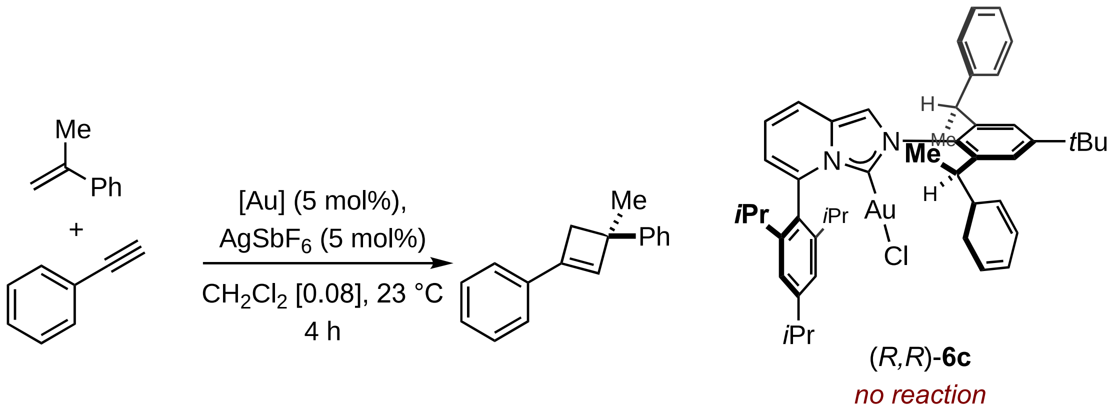

The reaction was performed following the protocol described in the literature.[12]

Methyl (1*R*,2*S*)-1,2-diphenylcyclopropane-1-carboxylate (11)

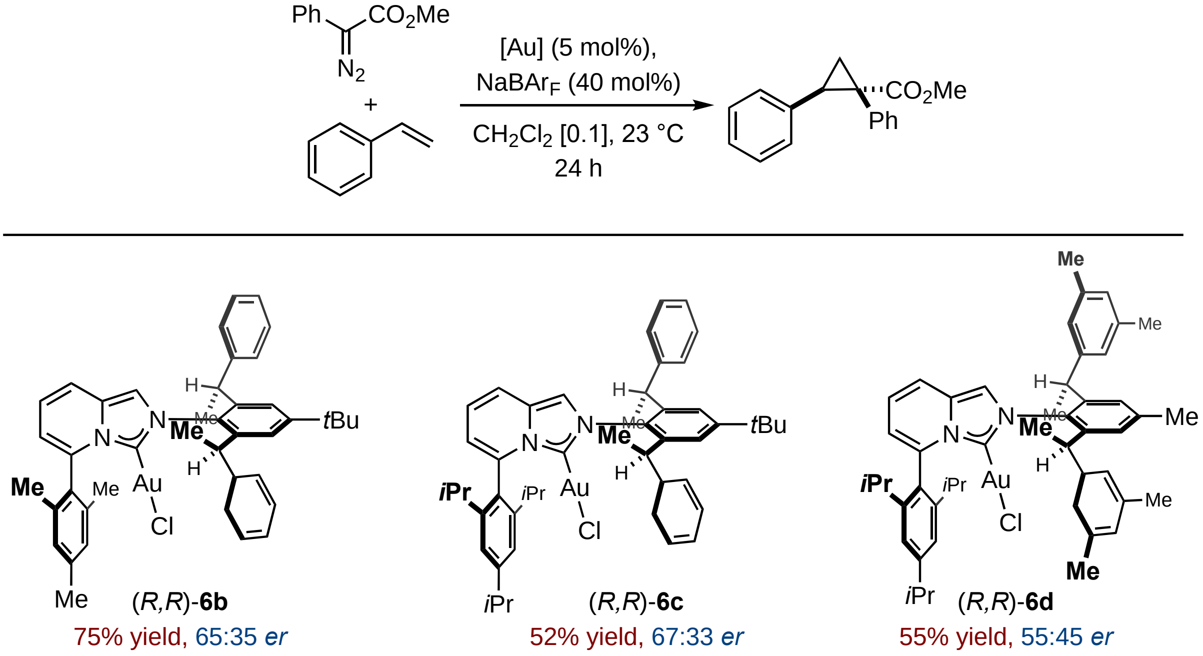

The reaction was performed following the protocol described in the literature.[13]

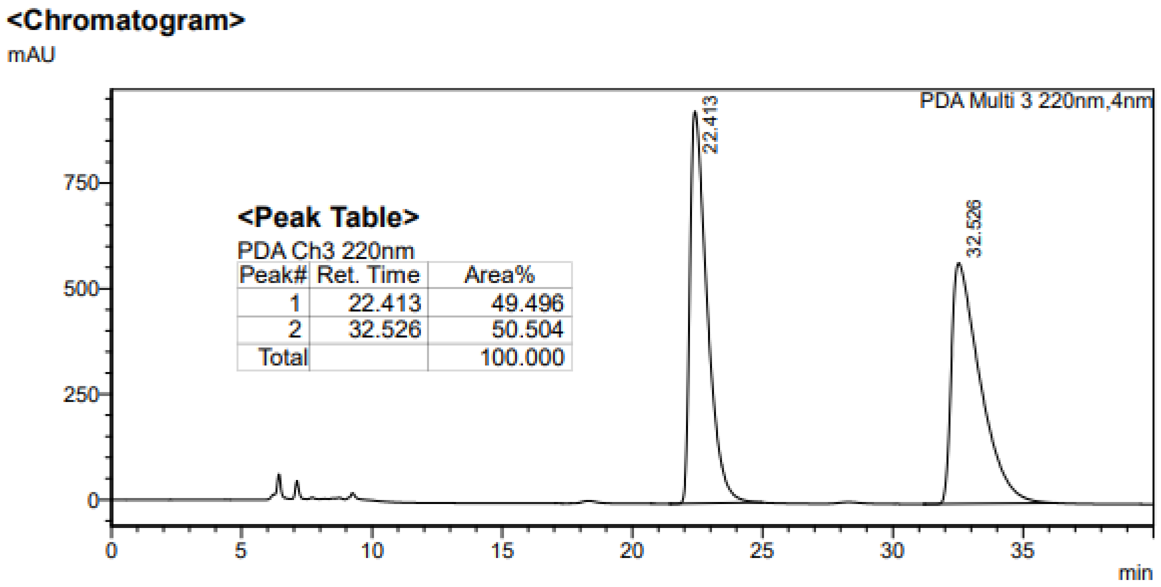

<Peak Table>

PDA Ch3 220nm

Peak#	Ret. Time	Area%
1	22.413	49.496
2	32.526	50.504
Total		100.000

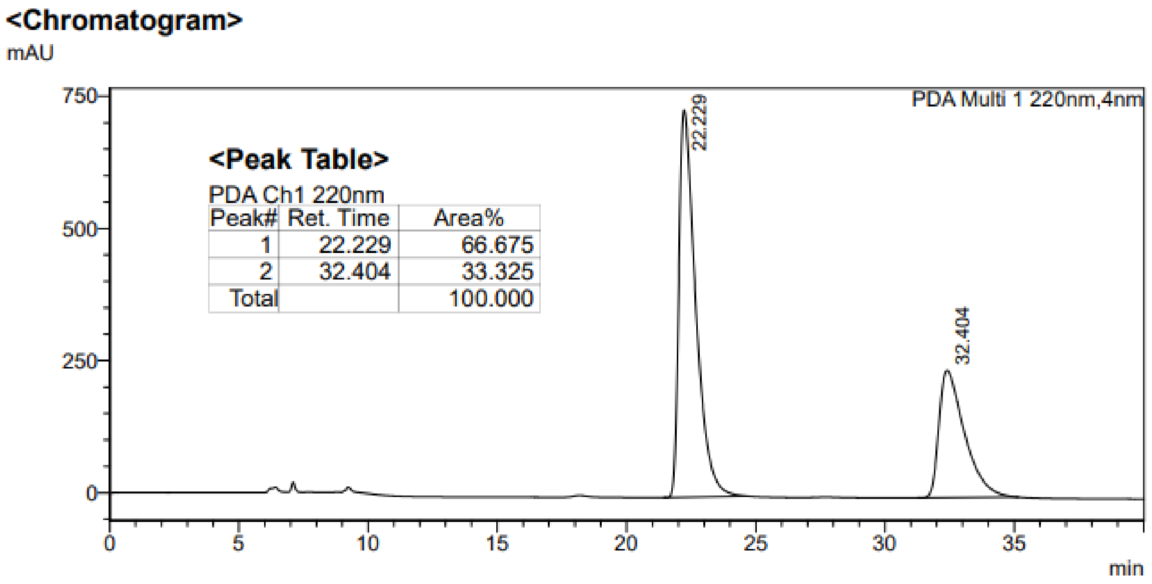

<Peak Table>

PDA Ch1 220nm

Peak#	Ret. Time	Area%
1	22.229	66.675
2	32.404	33.325
Total		100.000

Figure S15: HPLC traces for compound **11** using complexes (*R*,*R*)-**6c** (OJ, 1% *i*PrOH in *n*-hexanes, 0.5 mL/min).

12. References

(1) Zhao, W.; Huang, L.; Guan, Y.; Wulff, W. D. Three-Component Asymmetric Catalytic Ugi Reaction—Concinnity from Diversity by Substrate-Mediated Catalyst Assembly. *Angew. Chem. Int. Ed.* **2014**, *53*, 3436–3441.

(2) Diesel, J.; Finogenova, A. M.; Cramer, N. Nickel-Catalyzed Enantioselective Pyridone C–H Functionalizations Enabled by a Bulky N-Heterocyclic Carbene Ligand. *J. Am. Chem. Soc.* **2018**, *140*, 4489–4493.

(3) Wang, Z.-C.; Luo, X.; Zhang, J.-W.; Liu, C.-F.; Koh, M. J.; Shi, S.-L. Enantioselective C–C Cross-Coupling of Unactivated Alkenes. *Nat Catal* **2023**, *6*, 1087–1097.

(4) Cai, Y.; Zhang, J.-W.; Li, F.; Liu, J.-M.; Shi, S.-L. Nickel/N-Heterocyclic Carbene Complex-Catalyzed Enantioselective Redox-Neutral Coupling of Benzyl Alcohols and Alkynes to Allylic Alcohols. *ACS Catal.* **2019**, *9*, 1–6. (5) Stoll, C.; Besnard, C.; Mazet, C. *Organometallics* **2025**, *44*, 952–958.

(6) Kim, Y.; Kim, Y.; Hur, M. Y.; Lee, E. Efficient Synthesis of Bulky *N*-Heterocyclic Carbene Ligands for Coinage Metal Complexes. *J. Organomet. Chem.* **2016**, *820*, 1–7.

(7) Pedrazzani, R.; Pintus, A.; De Ventura, R.; Marchini, M.; Ceroni, P.; Silva López, C.; Monari, M.; Bandini, M. Boosting Gold(I) Catalysis via Weak Interactions: New Fine-Tunable Impy Ligands. *ACS Org. Inorg. Au* **2022**, *2*, 229–235.

(8) Makowska-Grzyska, M. M.; Szajna, E.; Shipley, C.; Arif, A. M.; Mitchell, M. H.; Halfen, J. A.; Berreau, L. M. First Row Divalent Transition Metal Complexes of Aryl-Appended Tris((Pyridyl)Methyl)Amine Ligands: Syntheses, Structures, Electrochemistry, and Hydroxamate Binding Properties. *Inorg. Chem.* **2003**, *42*, 7472–7488.

(9) Wang, C.-Y.; Liu, Y.-H.; Peng, S.-M.; Liu, S.-T. Rhodium(I) Complexes Containing a Bulky Pyridinyl N-Heterocyclic Carbene Ligand: Preparation and Reactivity. *J. Organomet. Chem.* **2006**, *691*, 4012–4020.

(10) Domski, G. J.; Lobkovsky, E. B.; Coates, G. W. Polymerization of α-Olefins with Pyridylamidohafnium Catalysts: Living Behavior and Unexpected Isoselectivity from a Cs-Symmetric Catalyst Precursor. *Macromolecules* **2007**, *40*, 3510–3513.

(11) Pedrazzani, R.; Pinosa, E.; Bertuzzi, G.; Monari, M.; Lauzon, S.; Ollevier, T.; Bandini, M. Convenient Synthesis of Tricyclic N(1)–C(2)-Fused Oxazino-Indolones *via* [Au(I)] Catalyzed Hydrocarboxylation of Allenes. *Chem. Commun.* **2022**, *58*, 8698–8701.

(12) López-Carrillo V.; Echavarren A.M. Gold(I)-catalyzed intermolecular [2+2] cycloaddition of alkynes with alkenes. *J. Am. Chem. Soc.* **2010**, *132*, 9292–9294.

(13) Prieto, A.; Fructos, M. R.; Mar Díaz-Requejo, M.; Pérez, P. J.; Pérez-Galán, P.; Delpont, N.; Echavarren, A. M. Gold-Catalyzed Olefin Cyclopropanation. *Tetrahedron* **2009**, *65*, 1790–1793.

13. NMR of new compounds

Figure S16. ^{1}H NMR spectrum (400 MHz, 298 K, $CDCl_3$) of **2e**

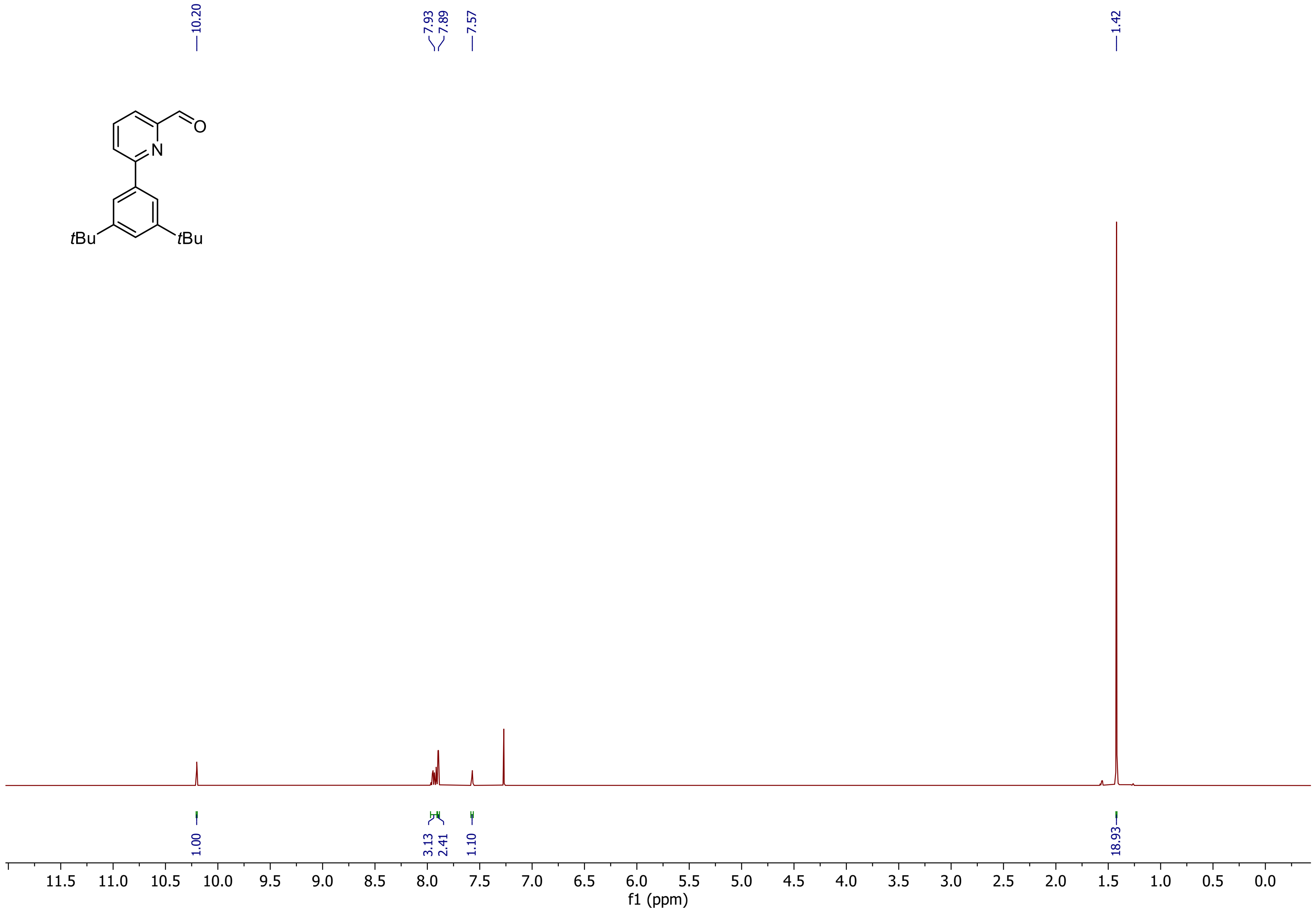

Figure S17. $^{13}C\{^1H\}$ NMR spectrum (101 MHz, 298 K, $CDCl_3$) of **2e**

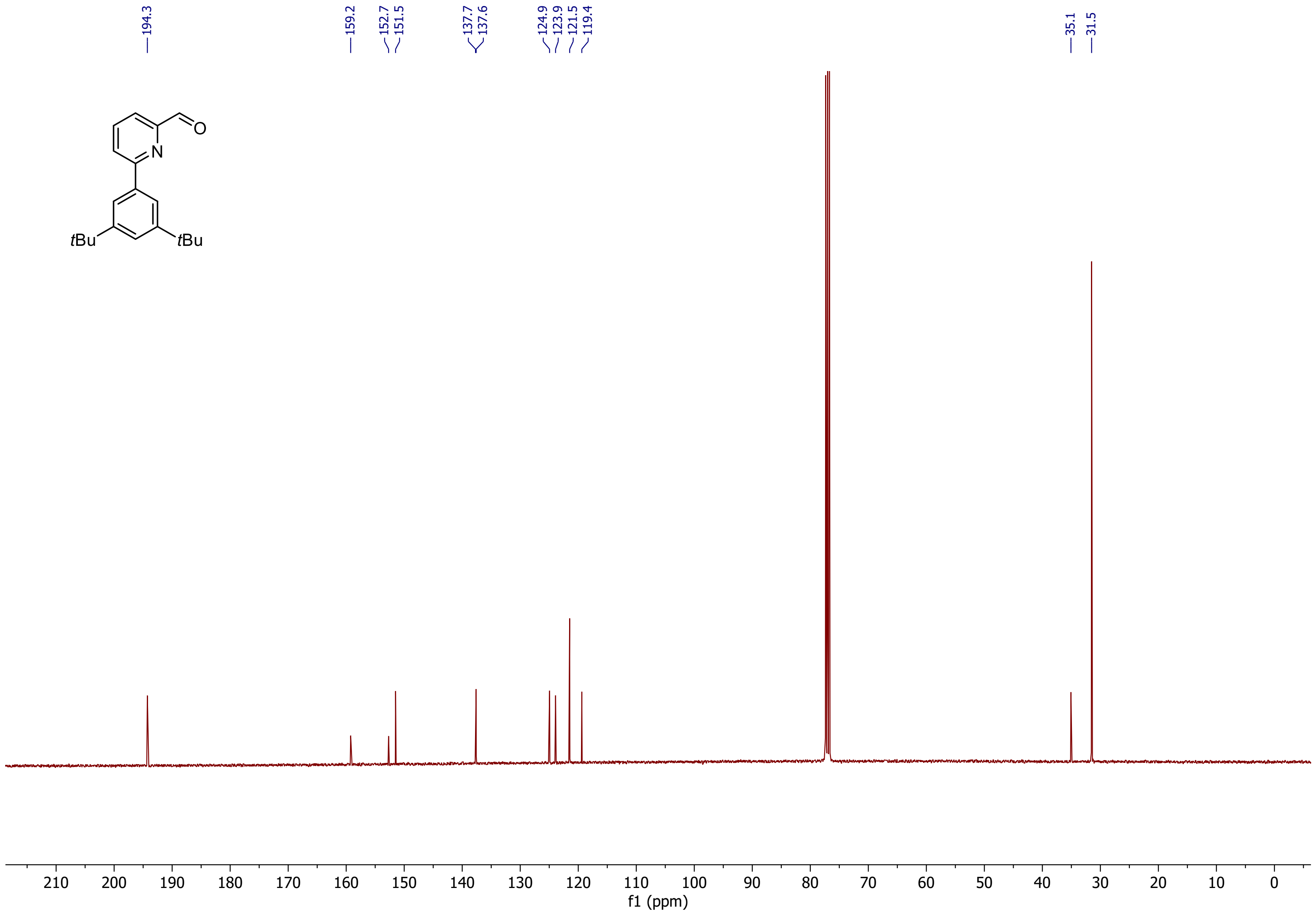

Figure S18. 2D ^{1}H-^{1}H COSY spectrum (298 K, $CDCl_3$) of **2e**

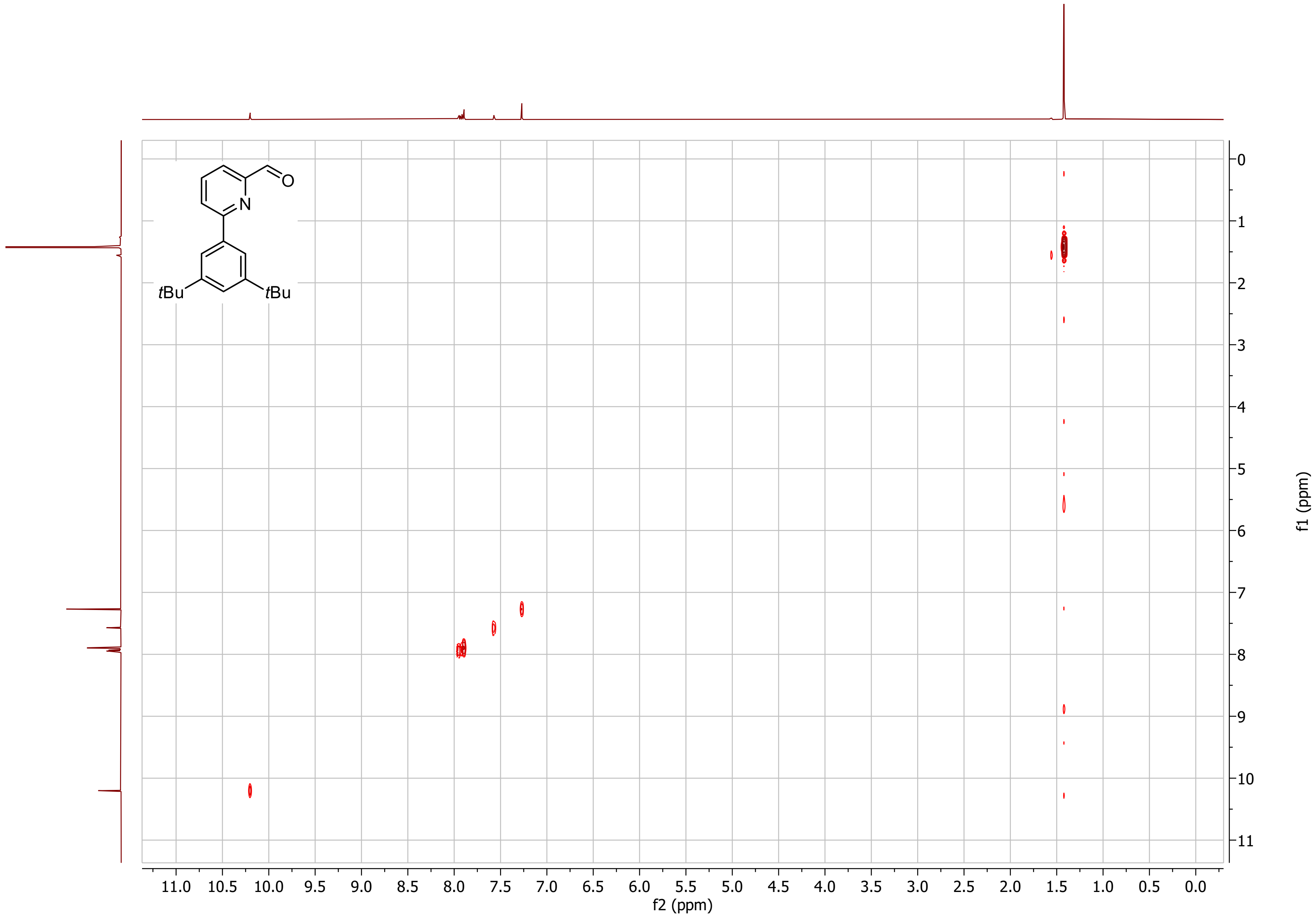

Figure S19. 2D ^{1}H-^{13}C HSQC spectrum (298 K, $CDCl_3$) of **2e**

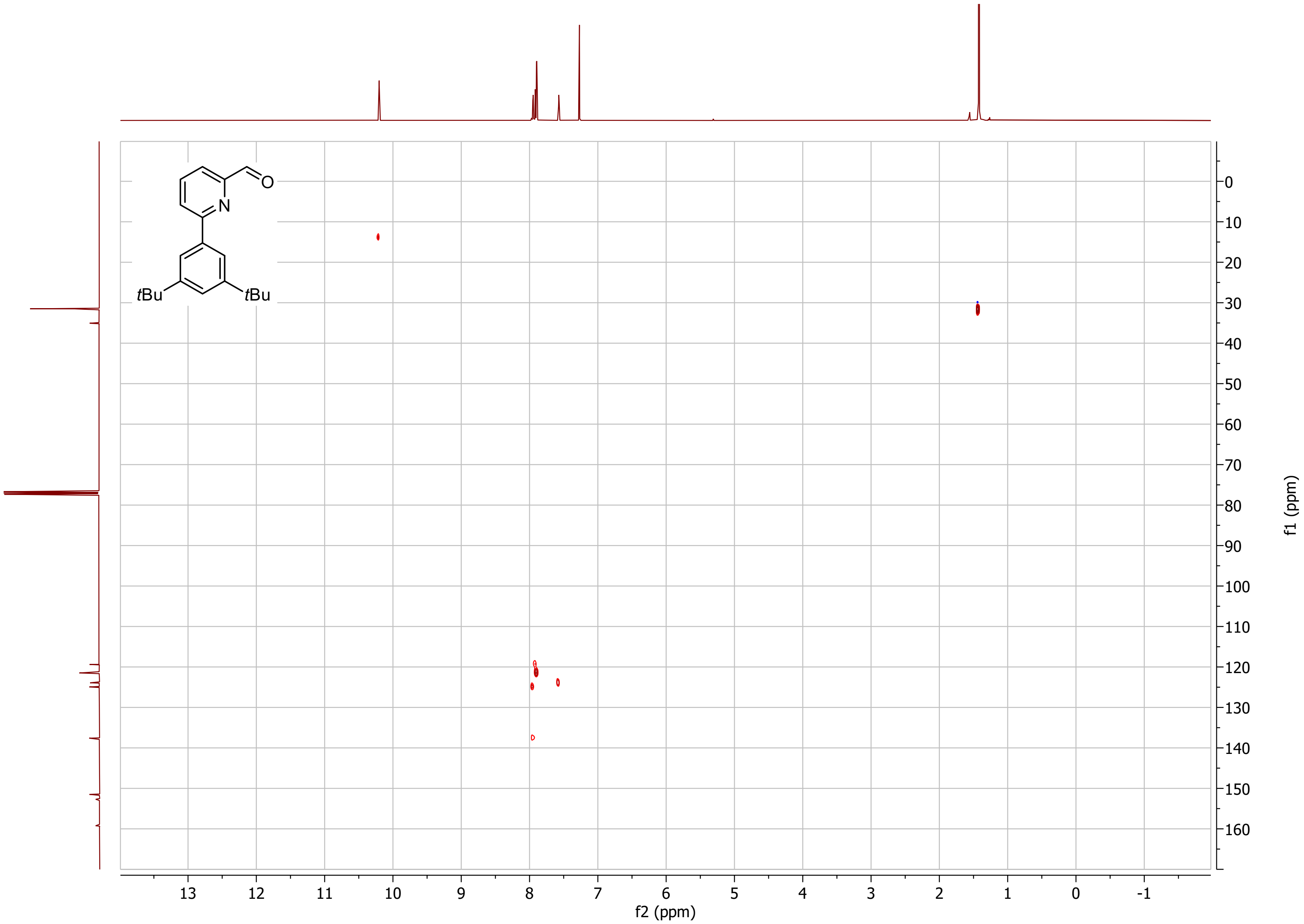

Figure S20. ^{1}H NMR spectrum (400 MHz, 298 K, $CDCl_3$) of **2h**

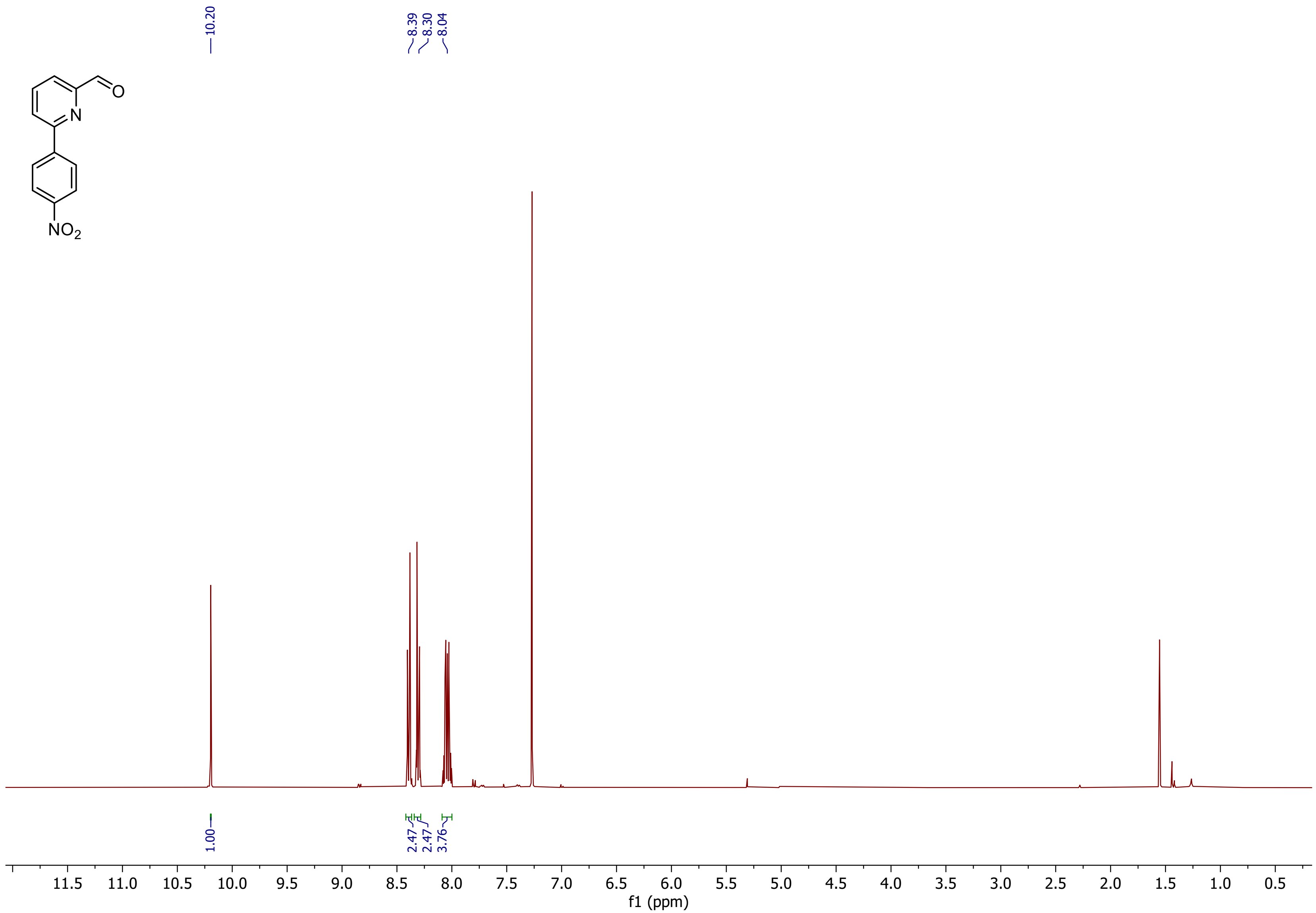

Figure S21. $^{13}C\{^1H\}$ NMR spectrum (101 MHz, 298 K, $CDCl_3$) of **2h**

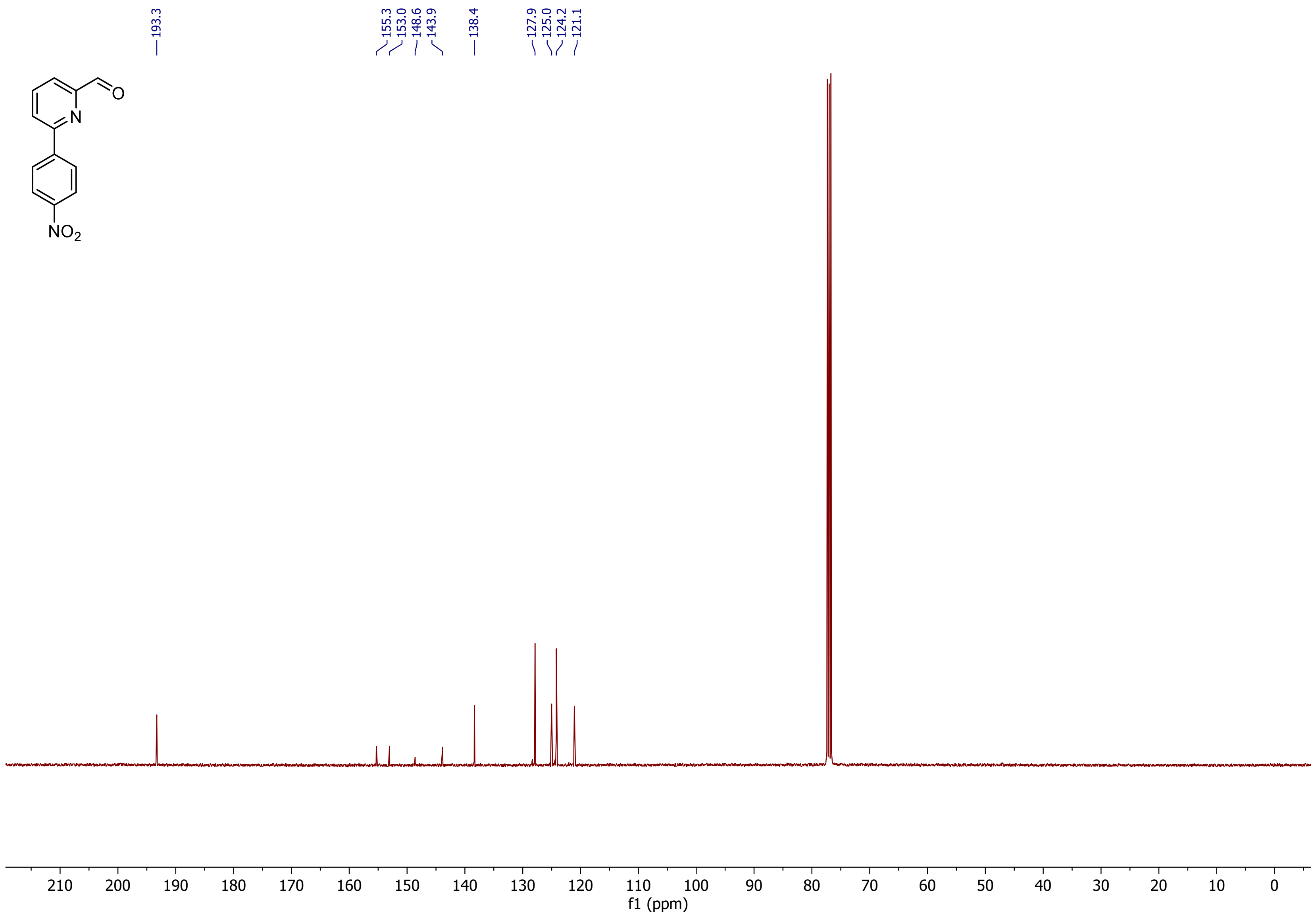

Figure S22. 2D ^{1}H-^{1}H COSY spectrum (298 K, $CDCl_3$) of **2h**

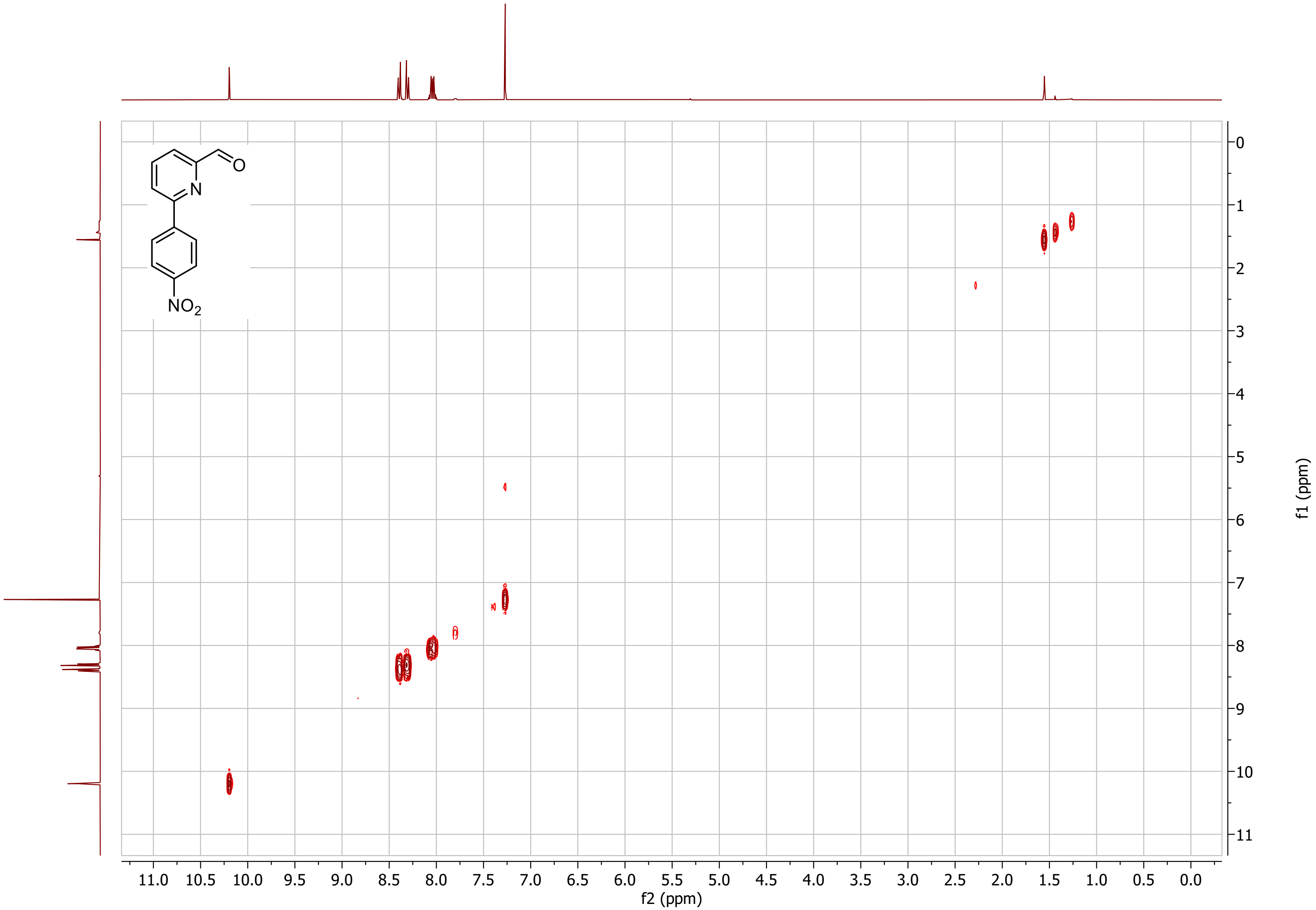

Figure S23. 2D ^{1}H-^{13}C HSQC spectrum (298 K, $CDCl_3$) of **2h**

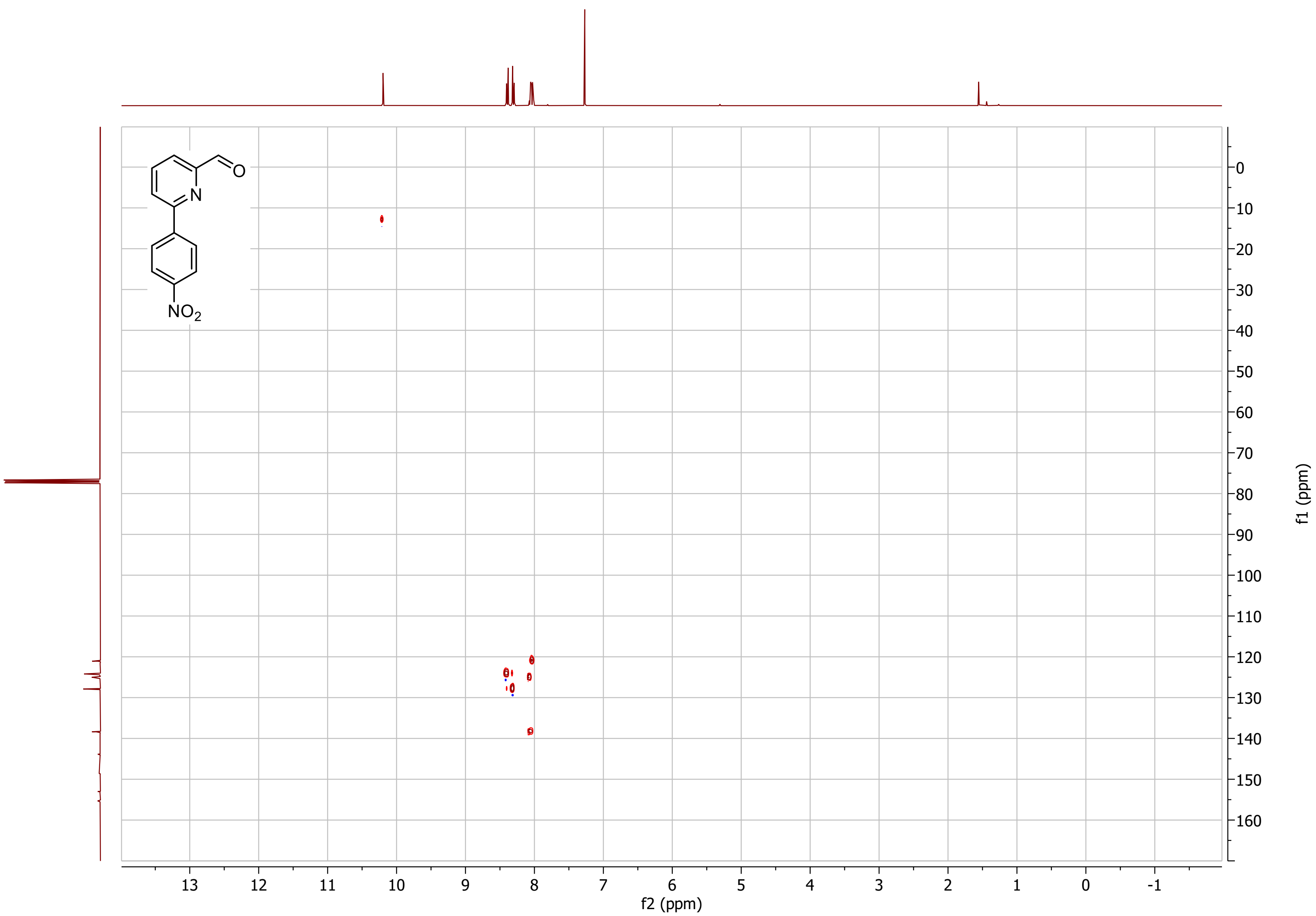

Figure S24. ^{1}H NMR spectrum (400 MHz, 298 K, $CDCl_3$) of (*R*,*R*)-**4a**

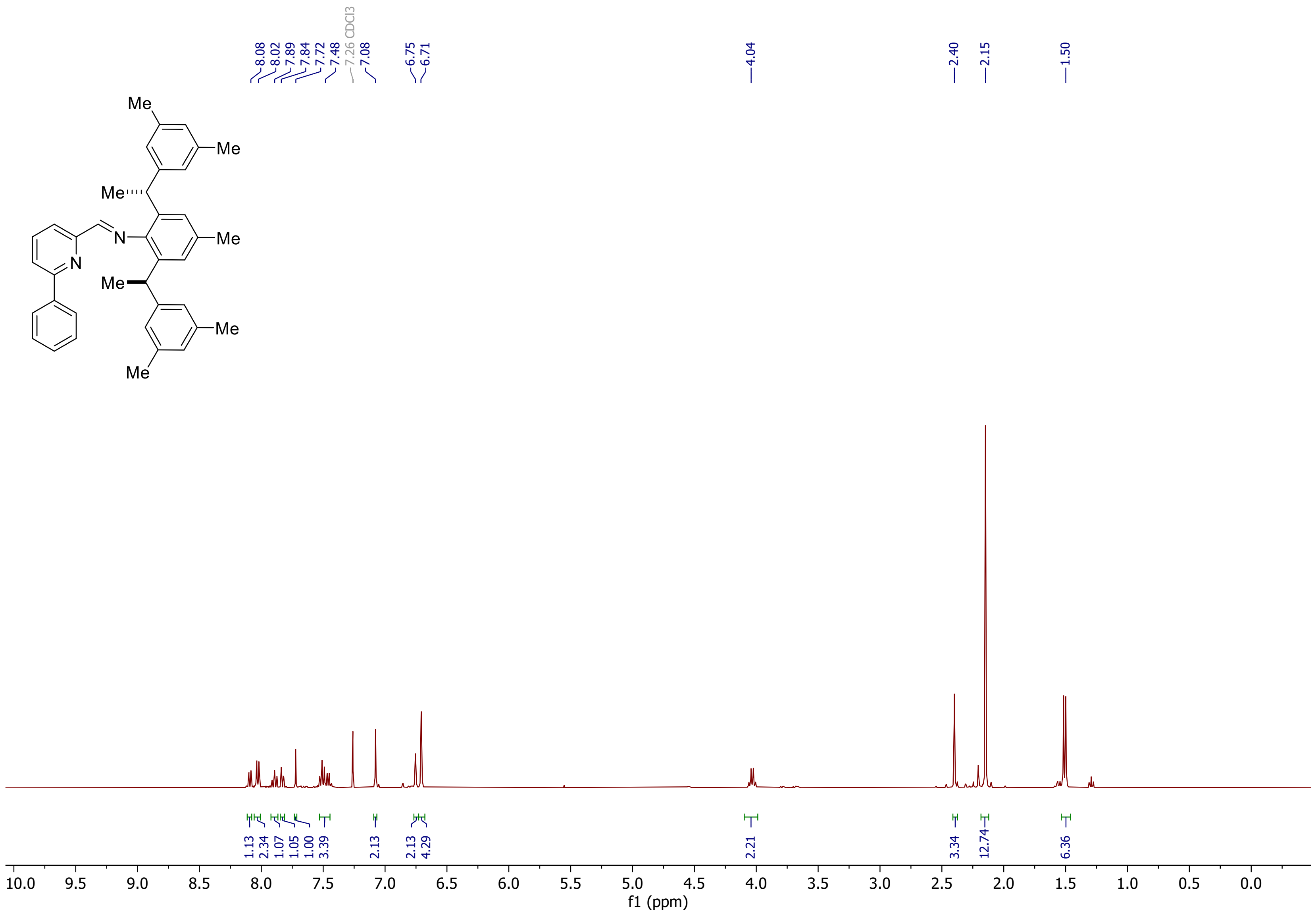

Figure S25. $^{13}C\{^{1}H\}$ NMR spectrum (101 MHz, 298 K, $CDCl_3$) of (*R*,*R*)-**4a**

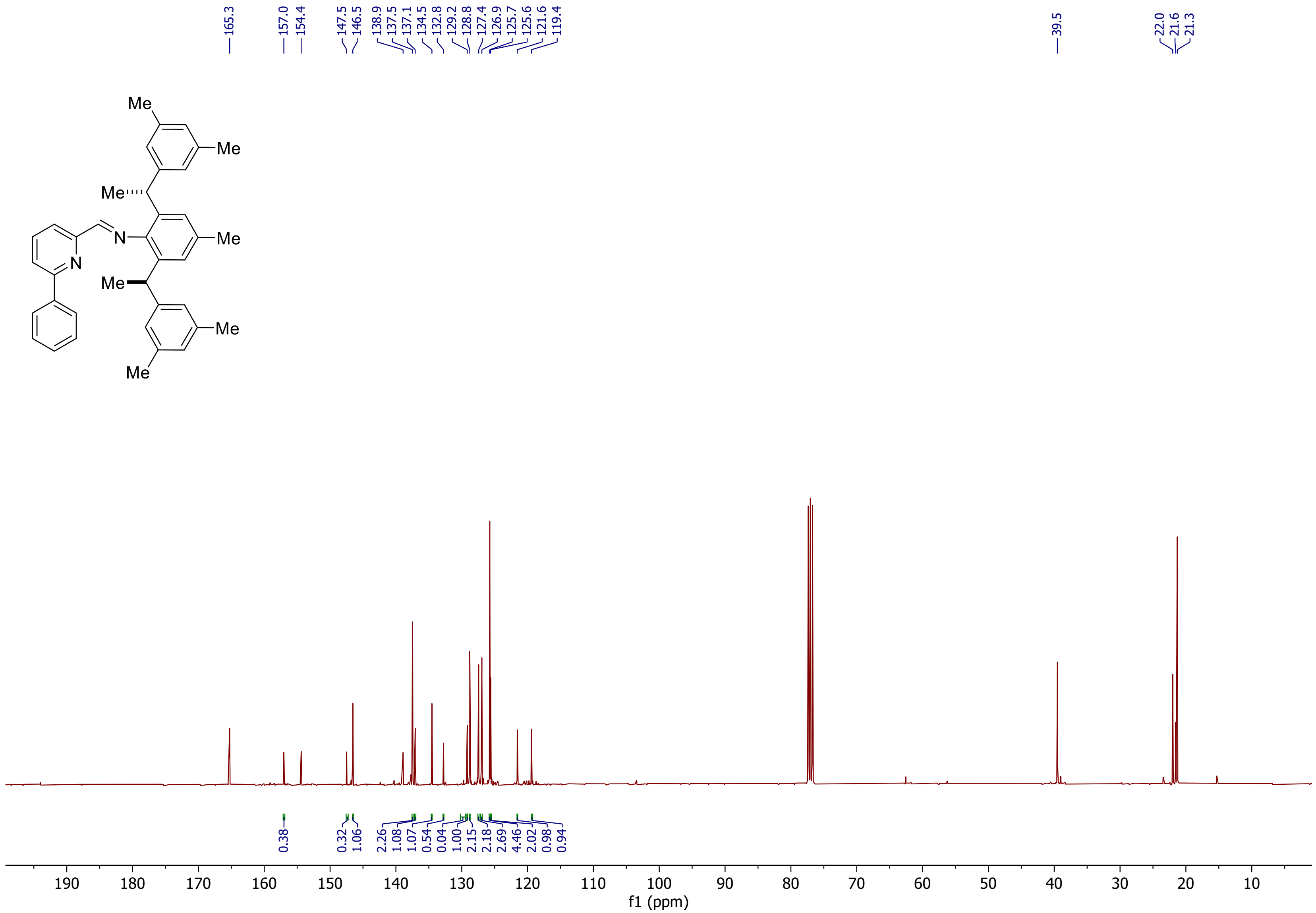

Figure S26. 2D ^{1}H-^{1}H COSY spectrum (298 K, $CDCl_3$) of (*R*,*R*)-**4a**

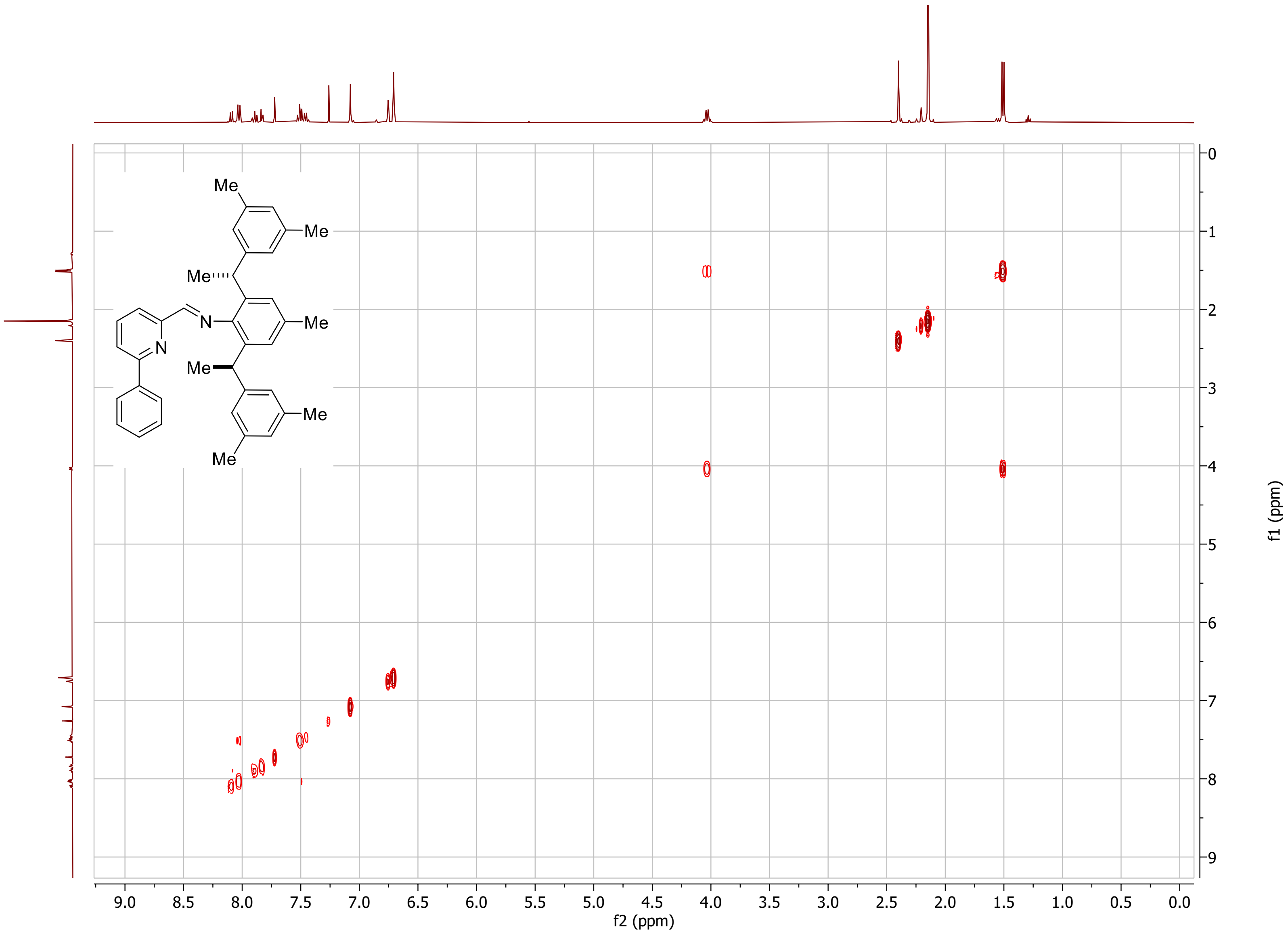

Figure S27. 2D ^1H-^{13}C HSQC spectrum (298 K, $CDCl_3$) of (*R*,*R*)-**4a**

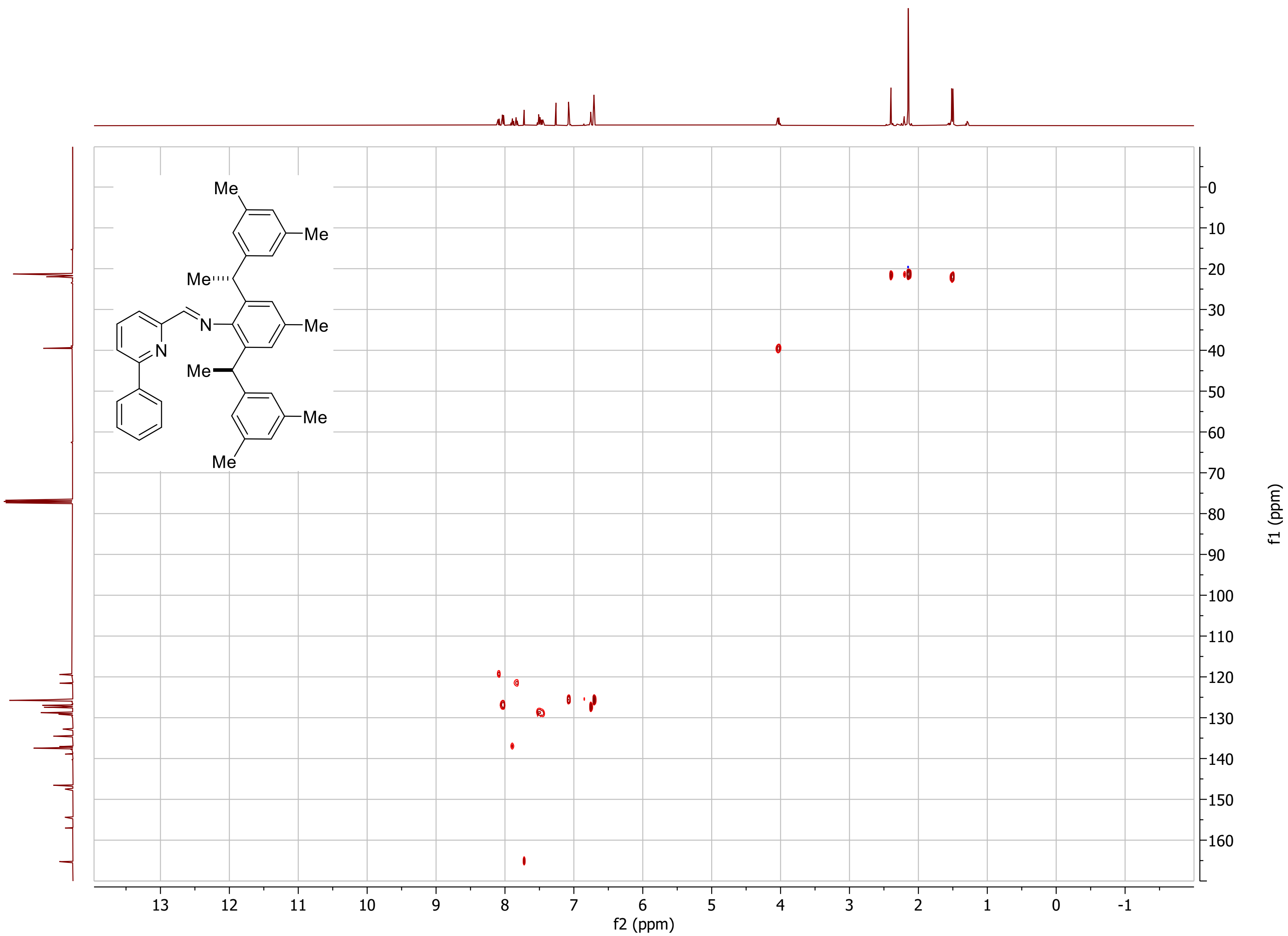

Figure S28. ^{1}H NMR spectrum (400 MHz, 298 K, $CDCl_3$) of (*R*,*R*)-**4b**

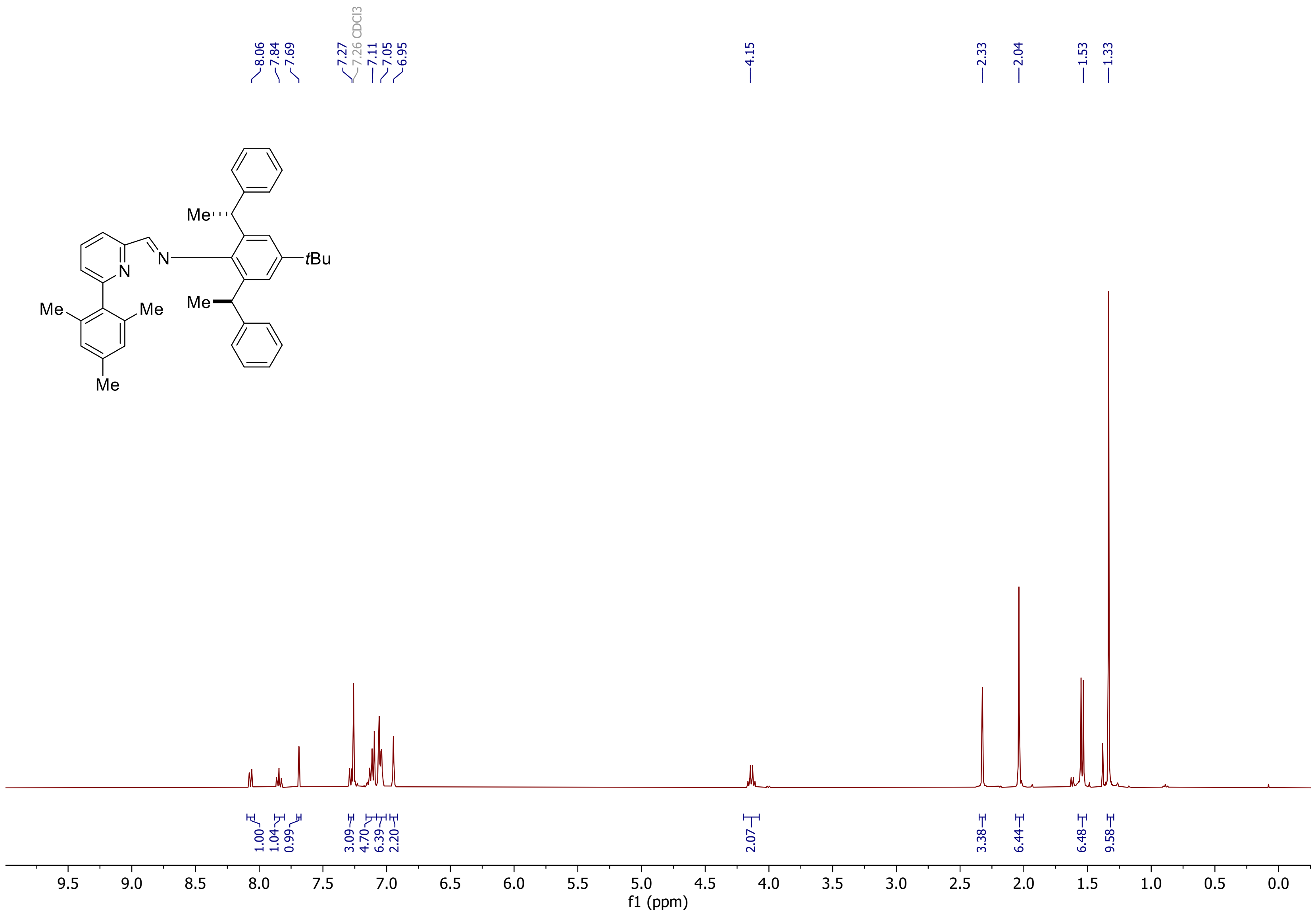

Figure S29. $^{13}C\{^1H\}$ NMR spectrum (101 MHz, 298 K, $CDCl_3$) of (*R*,*R*)-**4b**

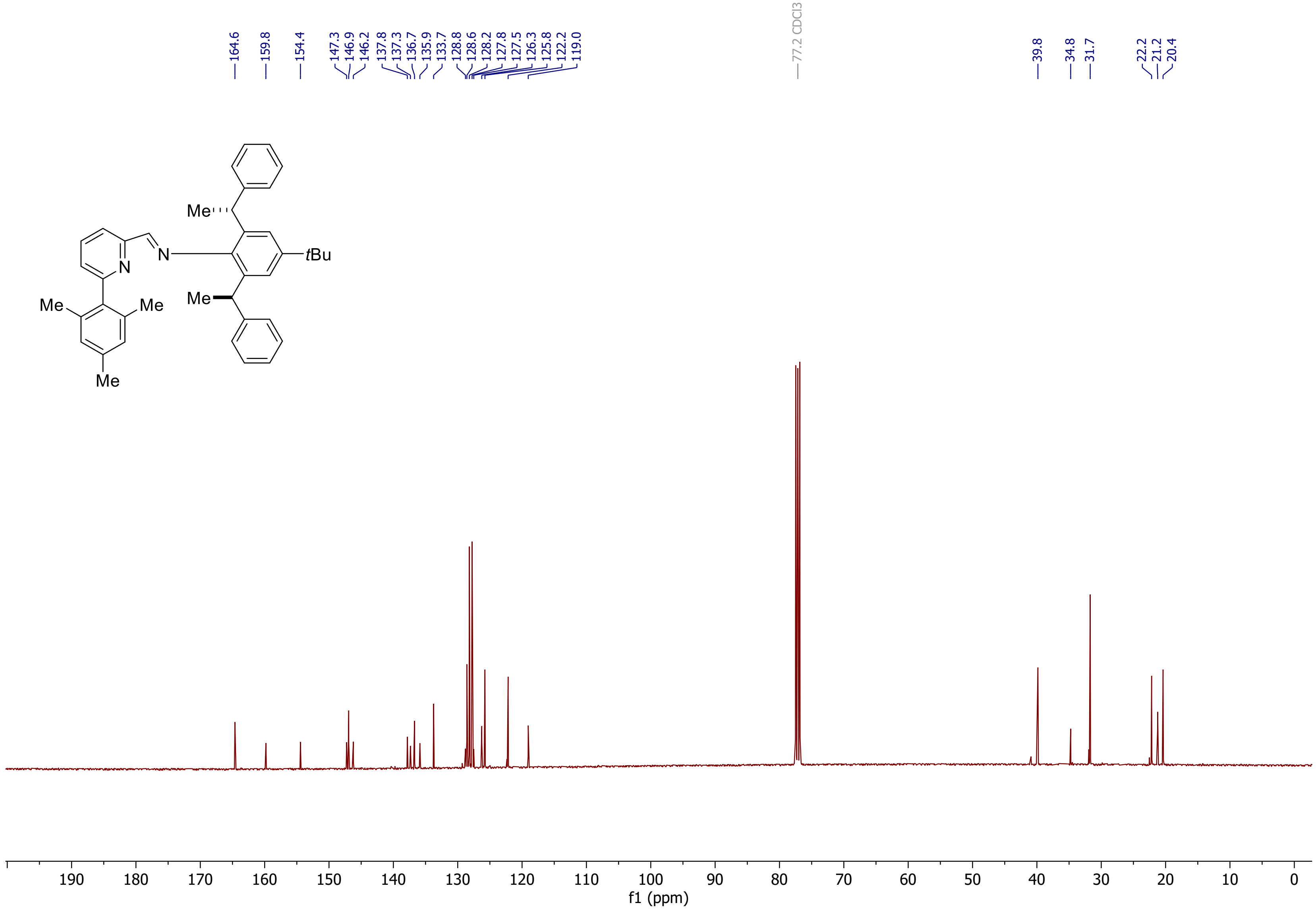

Figure S30. 2D ^{1}H-^{1}H COSY spectrum (298 K, $CDCl_3$) of (*R*,*R*)-**4b**

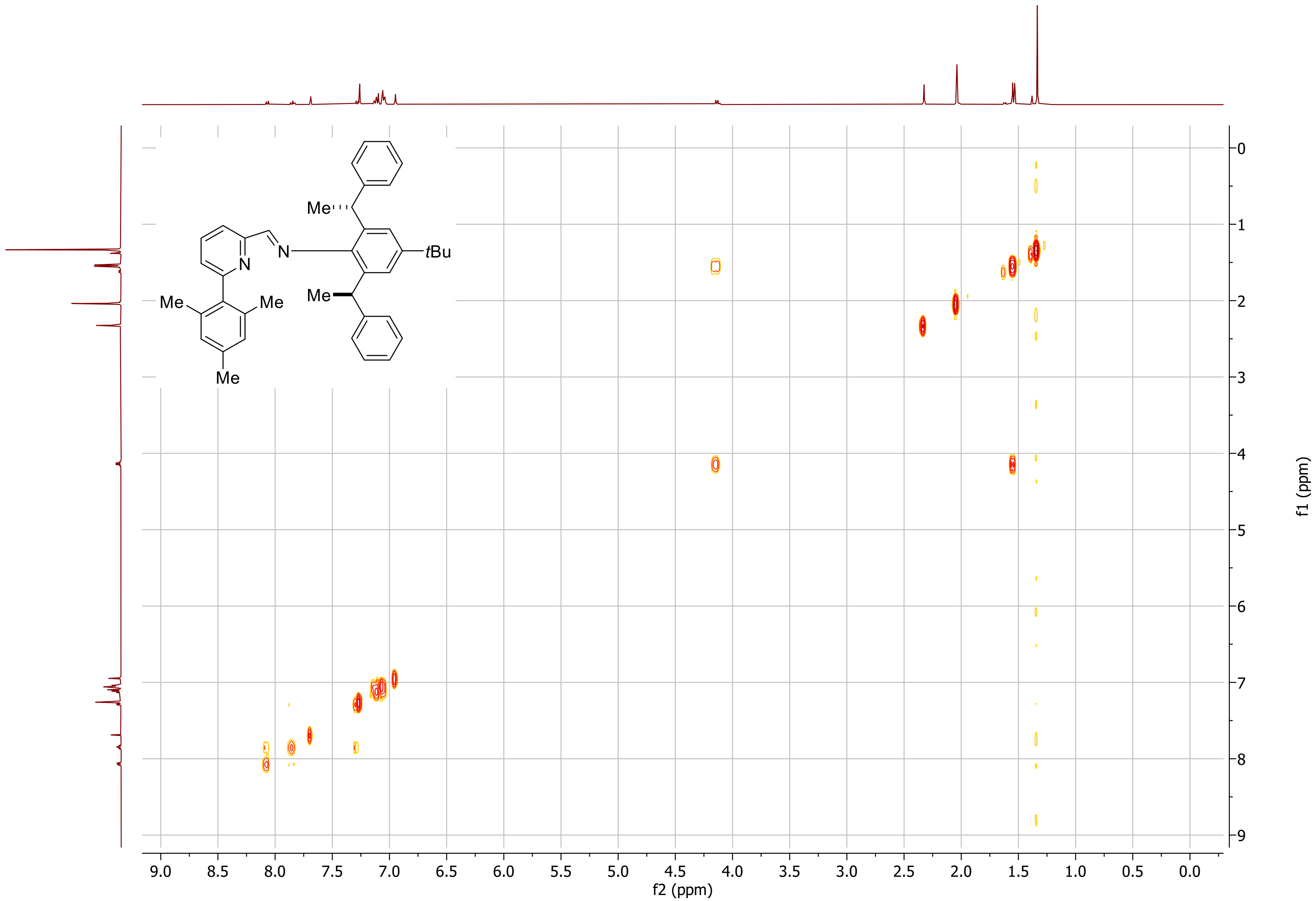

Figure S31. 2D ^1H-^{13}C HSQC spectrum (298 K, $CDCl_3$) of (*R*,*R*)-**4b**

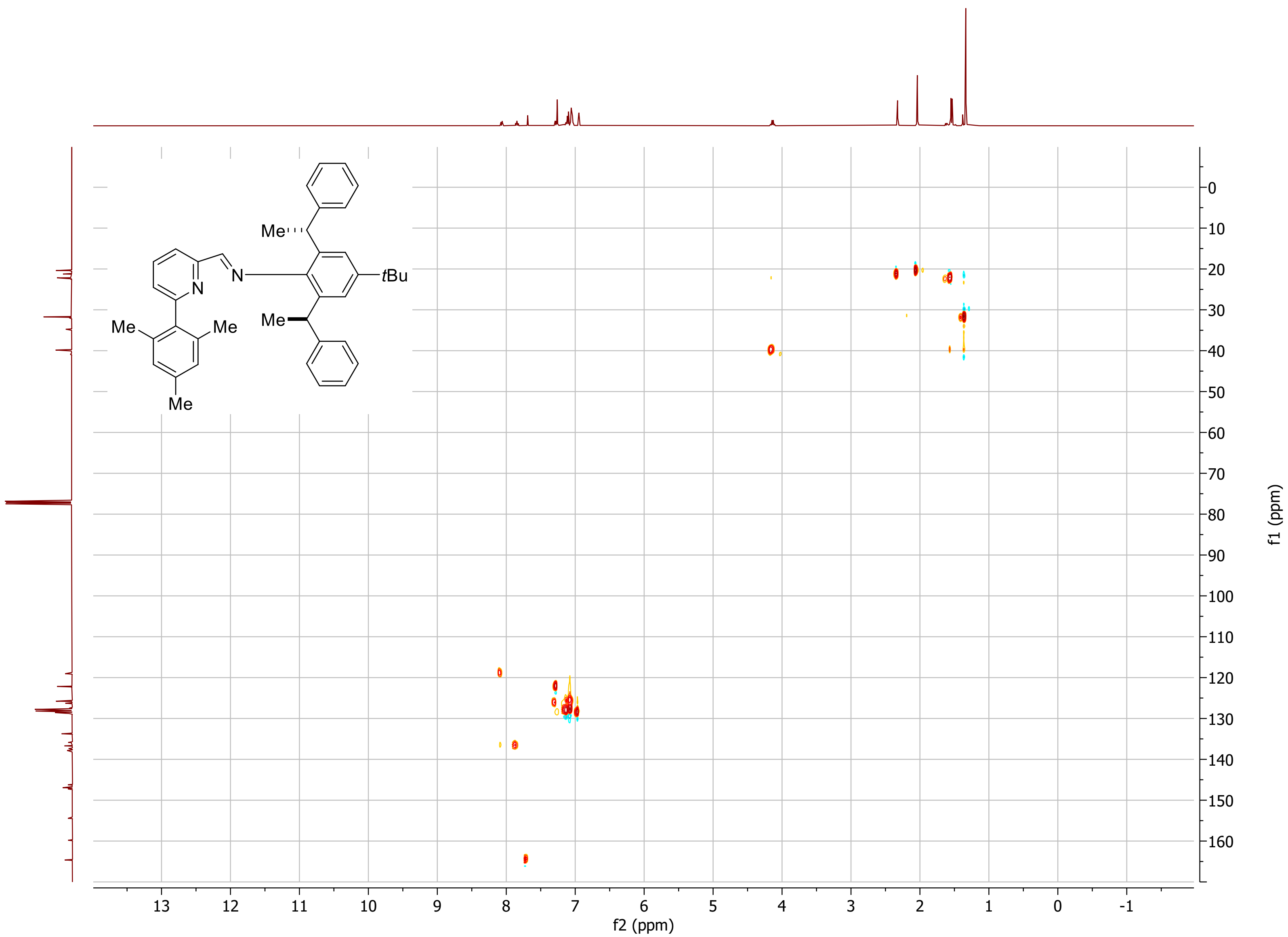

Figure S32. ^{1}H NMR spectrum (400 MHz, 298 K, $CDCl_3$) of (*R*,*R*)-**4c**

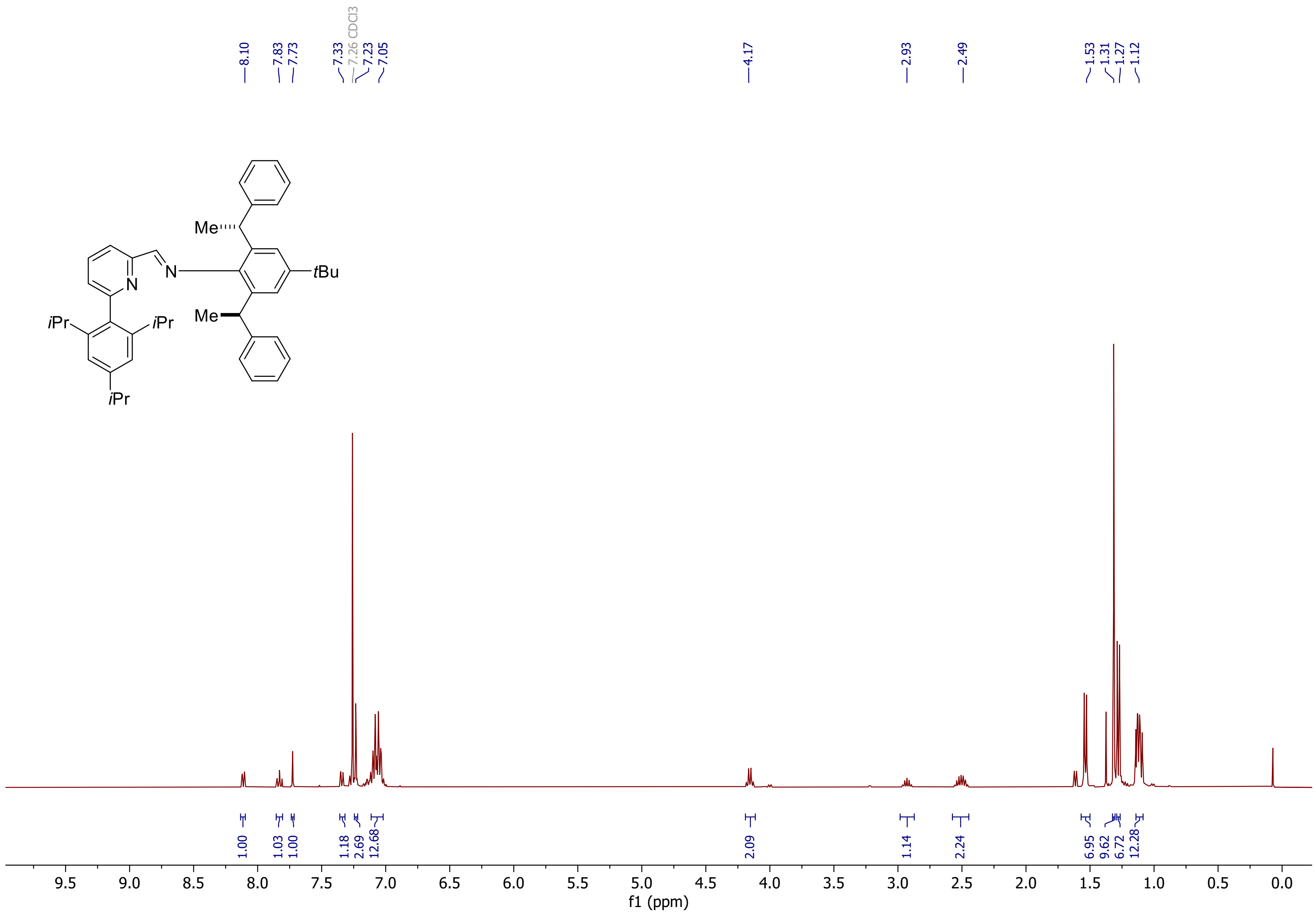

Figure S33. $^{13}C\{^{1}H\}$ NMR spectrum (101 MHz, 298 K, $CDCl_3$) of (*R*,*R*)-**4c**

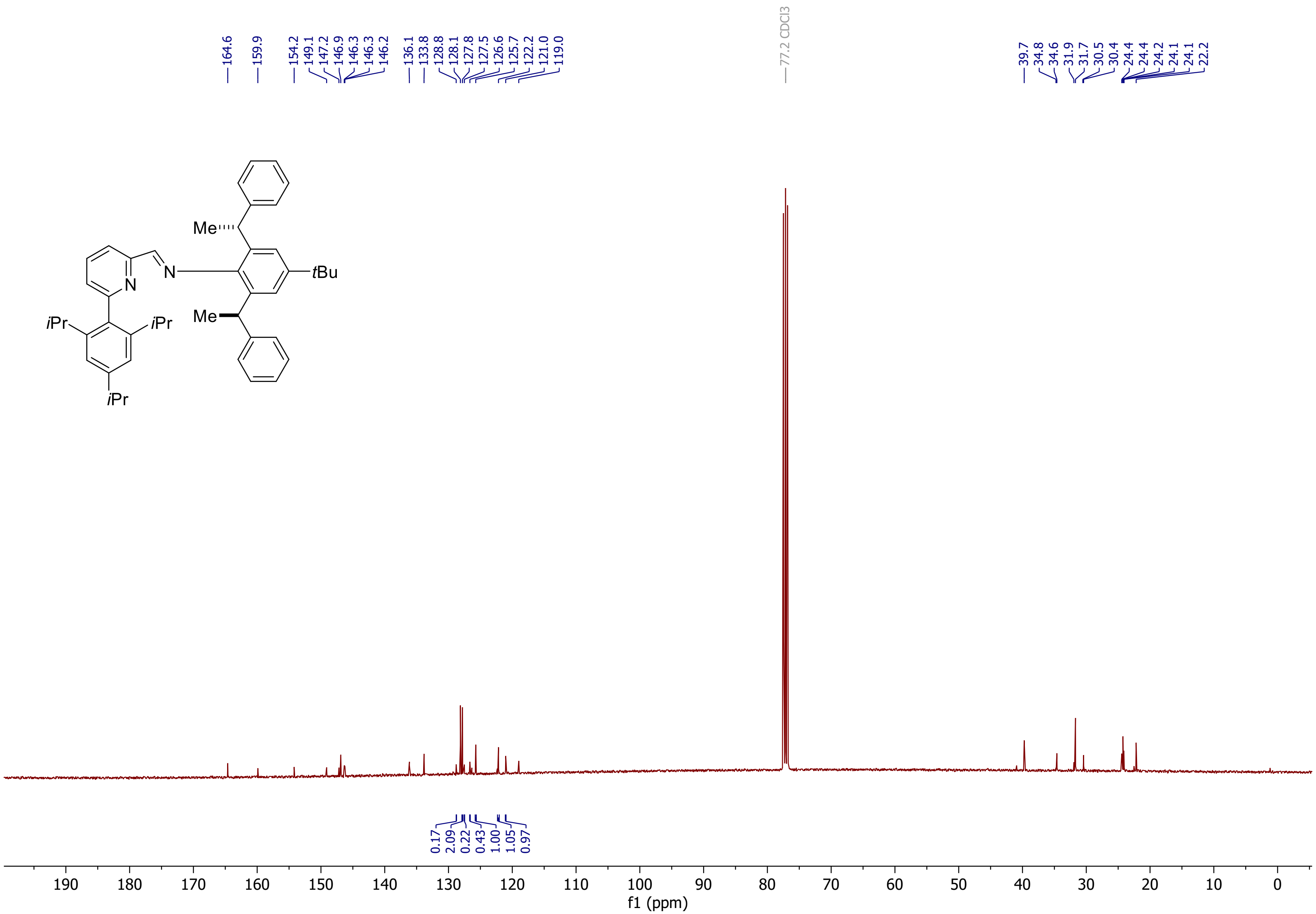

Figure S34. 2D ^1H-^1H COSY spectrum (298 K, $CDCl_3$) of (*R*,*R*)-**4c**

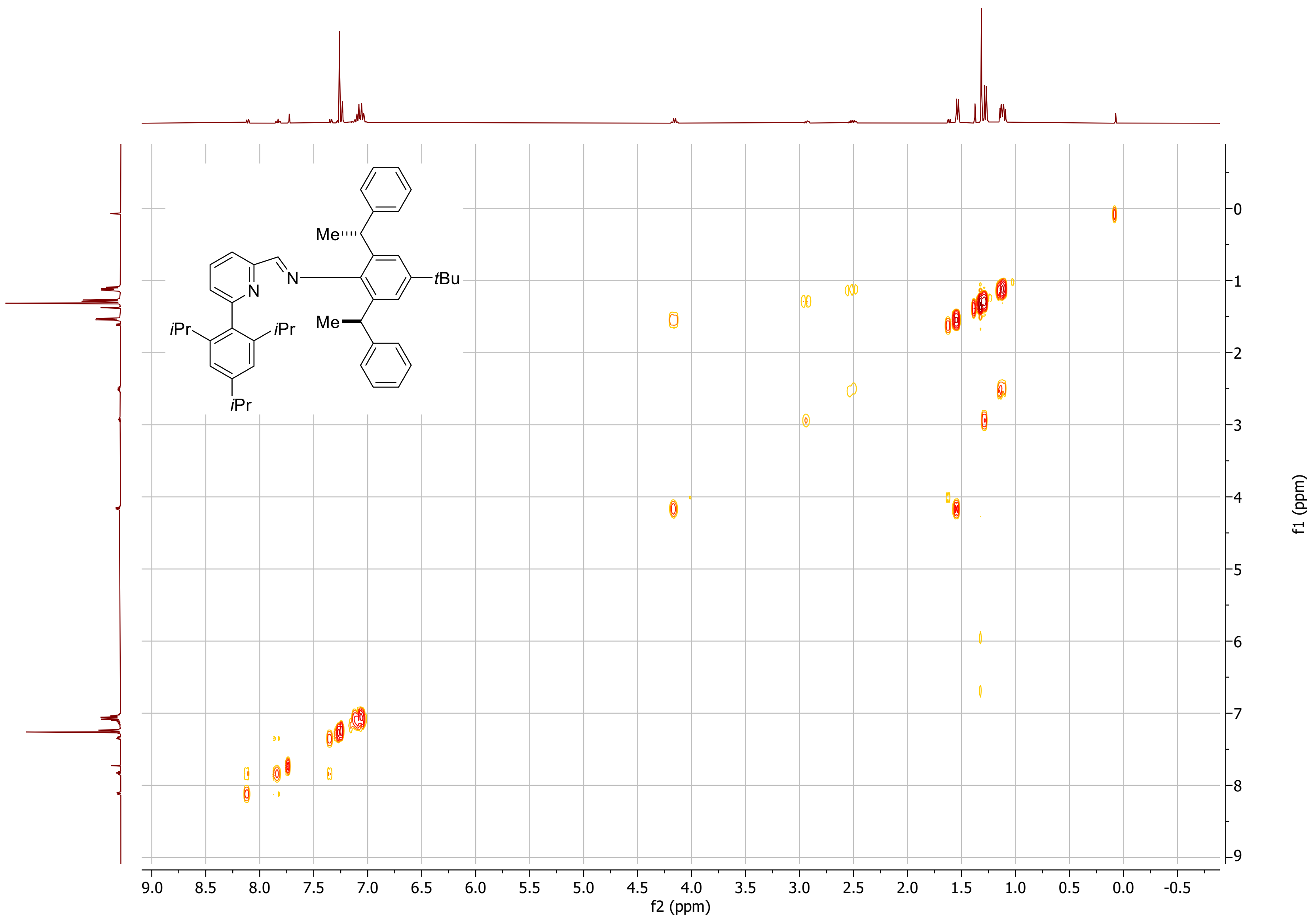

Figure S35. 2D ^{1}H-^{13}C HSQC spectrum (298 K, $CDCl_3$) of (*R*,*R*)-**4c**

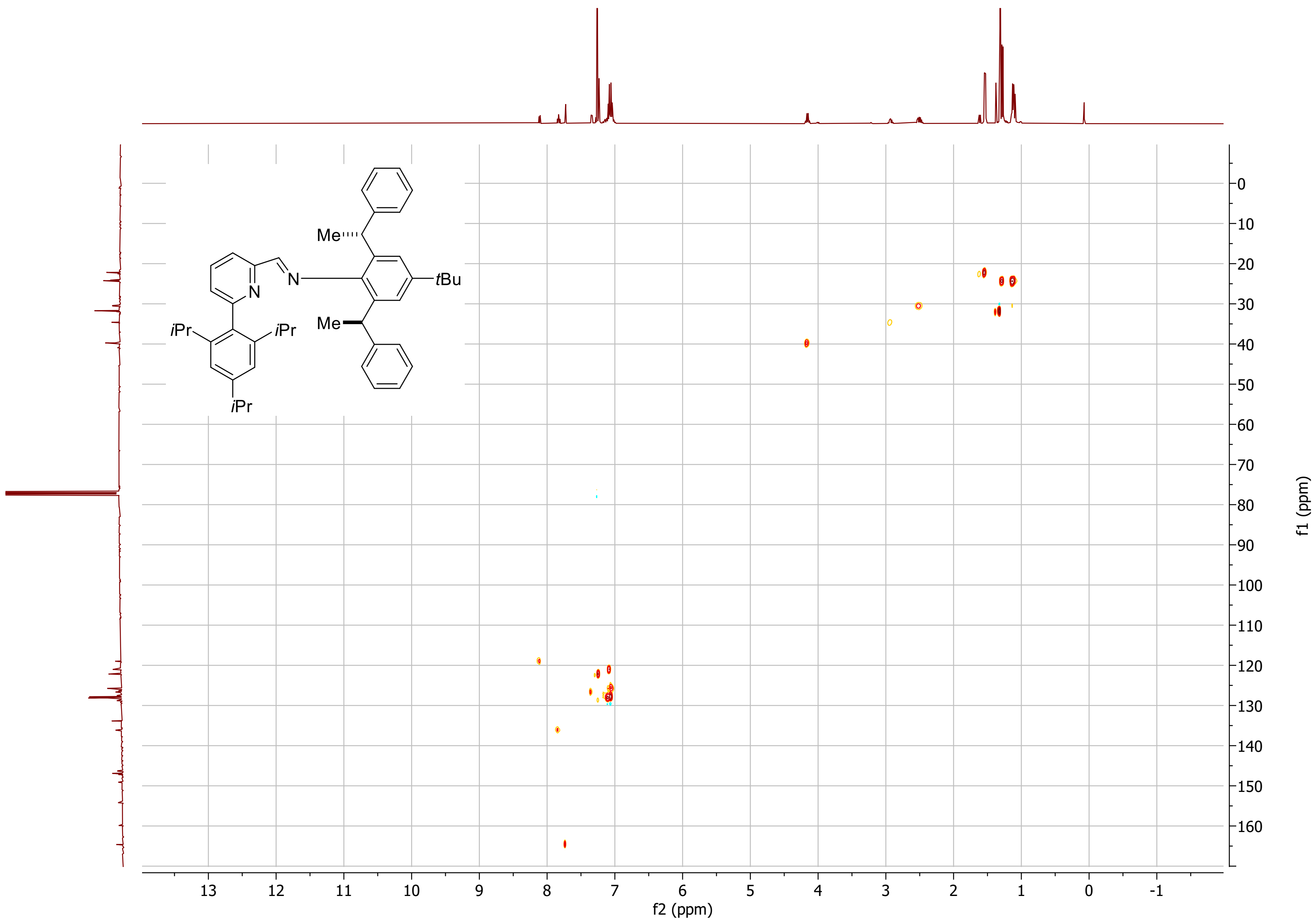

Figure S36. ^{1}H NMR spectrum (400 MHz, 298 K, $CDCl_3$) of (*R*,*R*)-**4d**

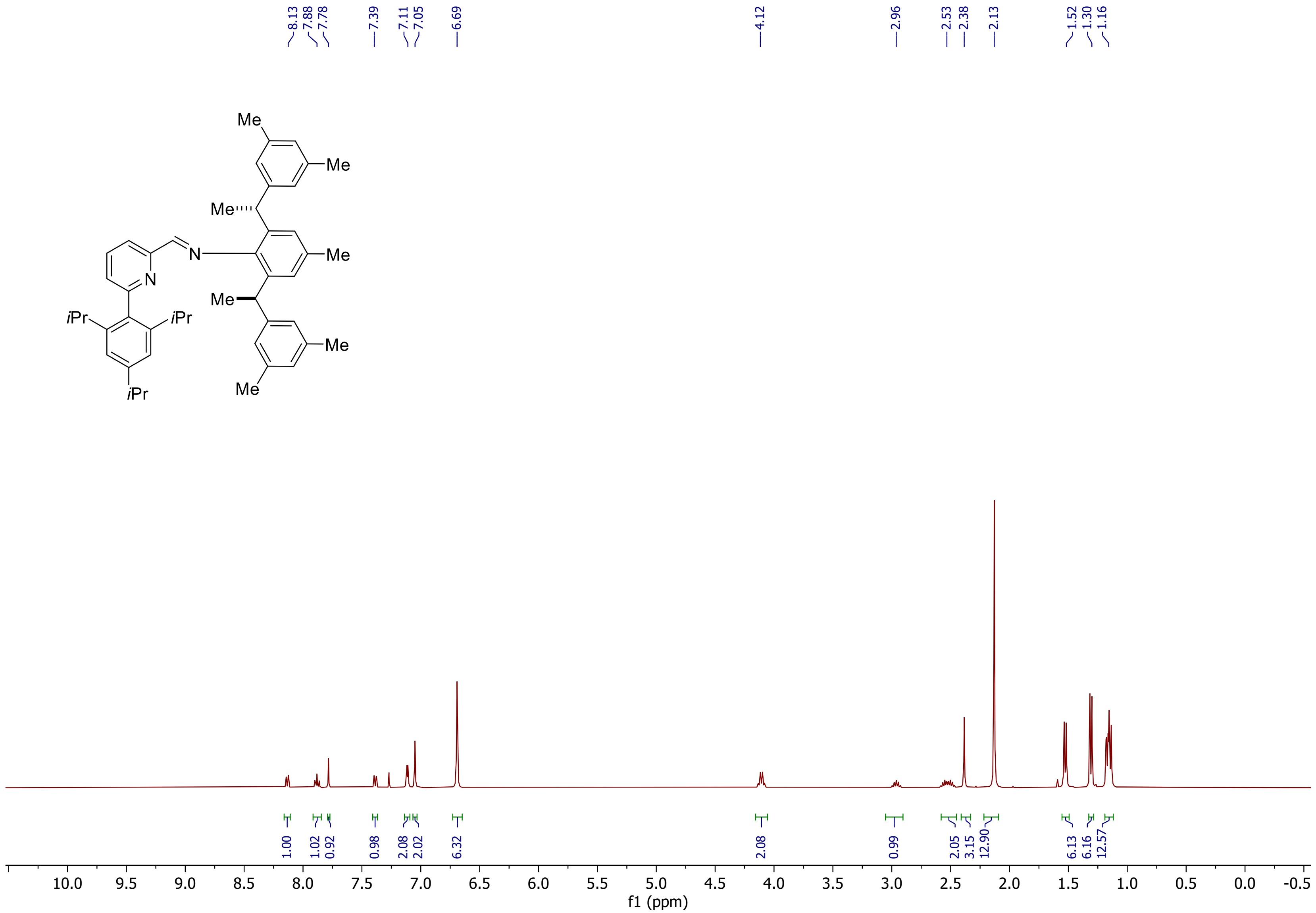

Figure S37. $^{13}C\{^{1}H\}$ NMR spectrum (101 MHz, 298 K, $CDCl_3$) of (*R*,*R*)-**4d**

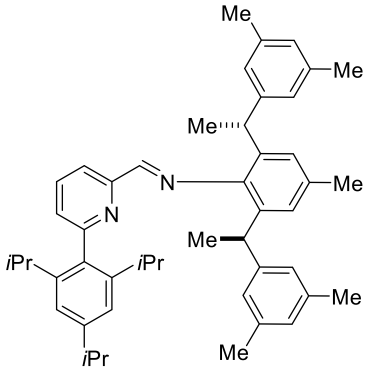

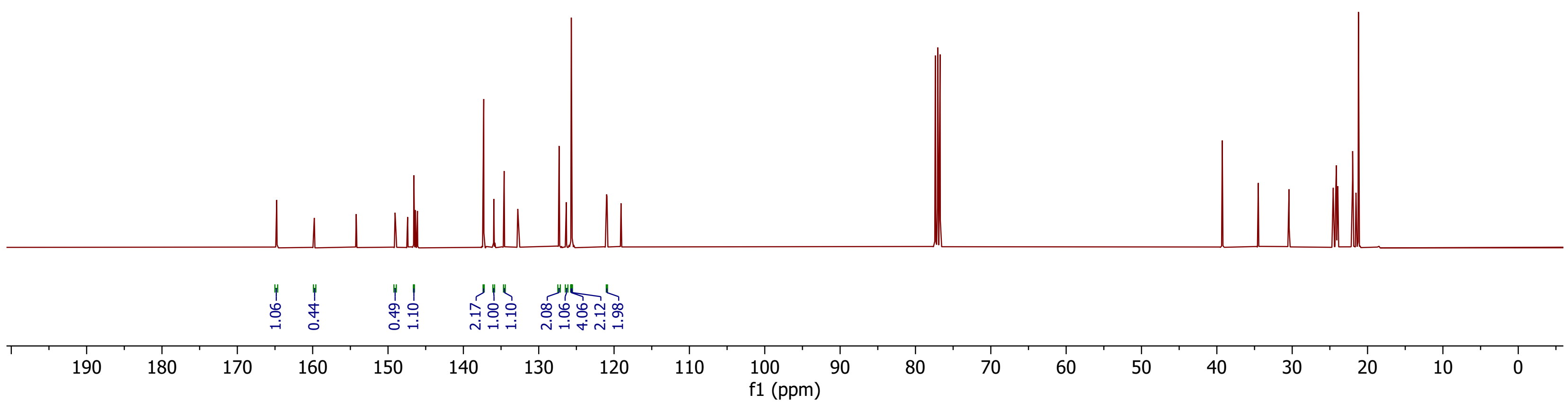

Figure S38. 2D ^1H-^1H COSY spectrum (298 K, $CDCl_3$) of (*R*,*R*)-**4d**

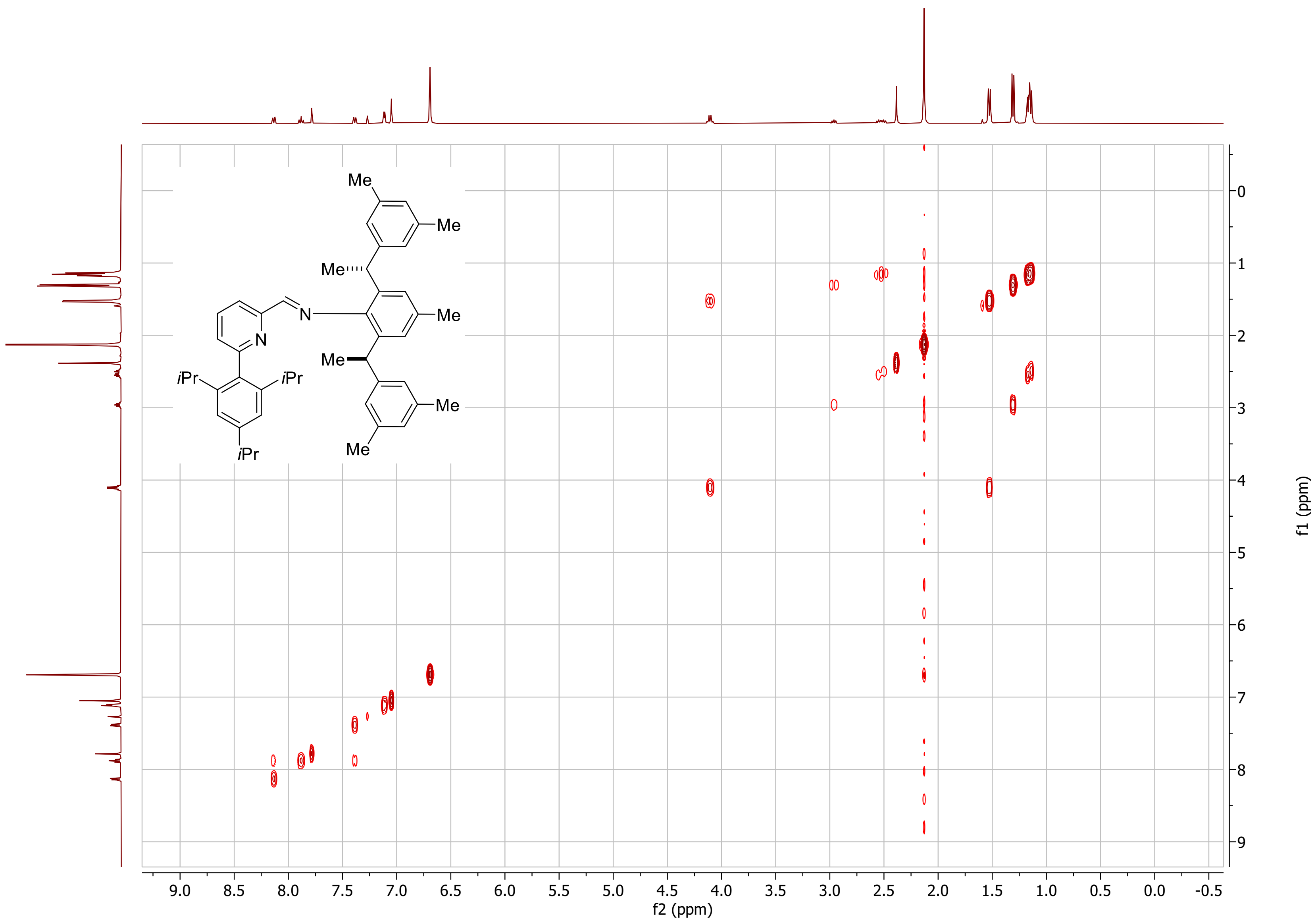

Figure S39. 2D ^{1}H-^{13}C HSQC spectrum (298 K, $CDCl_3$) of (*R*,*R*)-**4d**

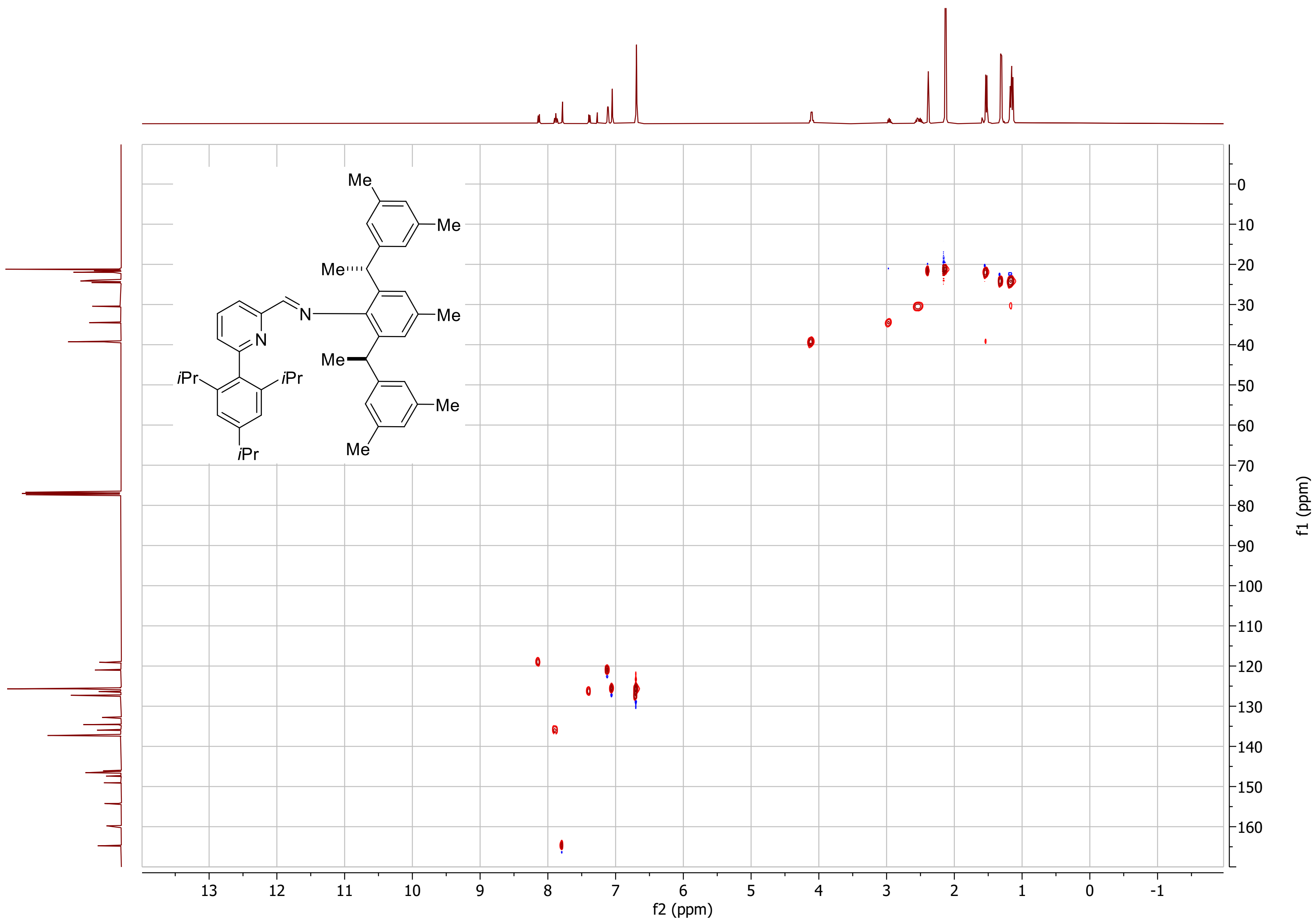

Figure S40. ^{1}H NMR spectrum (400 MHz, 298 K, $CDCl_3$) of (*R*,*R*)-**4e**

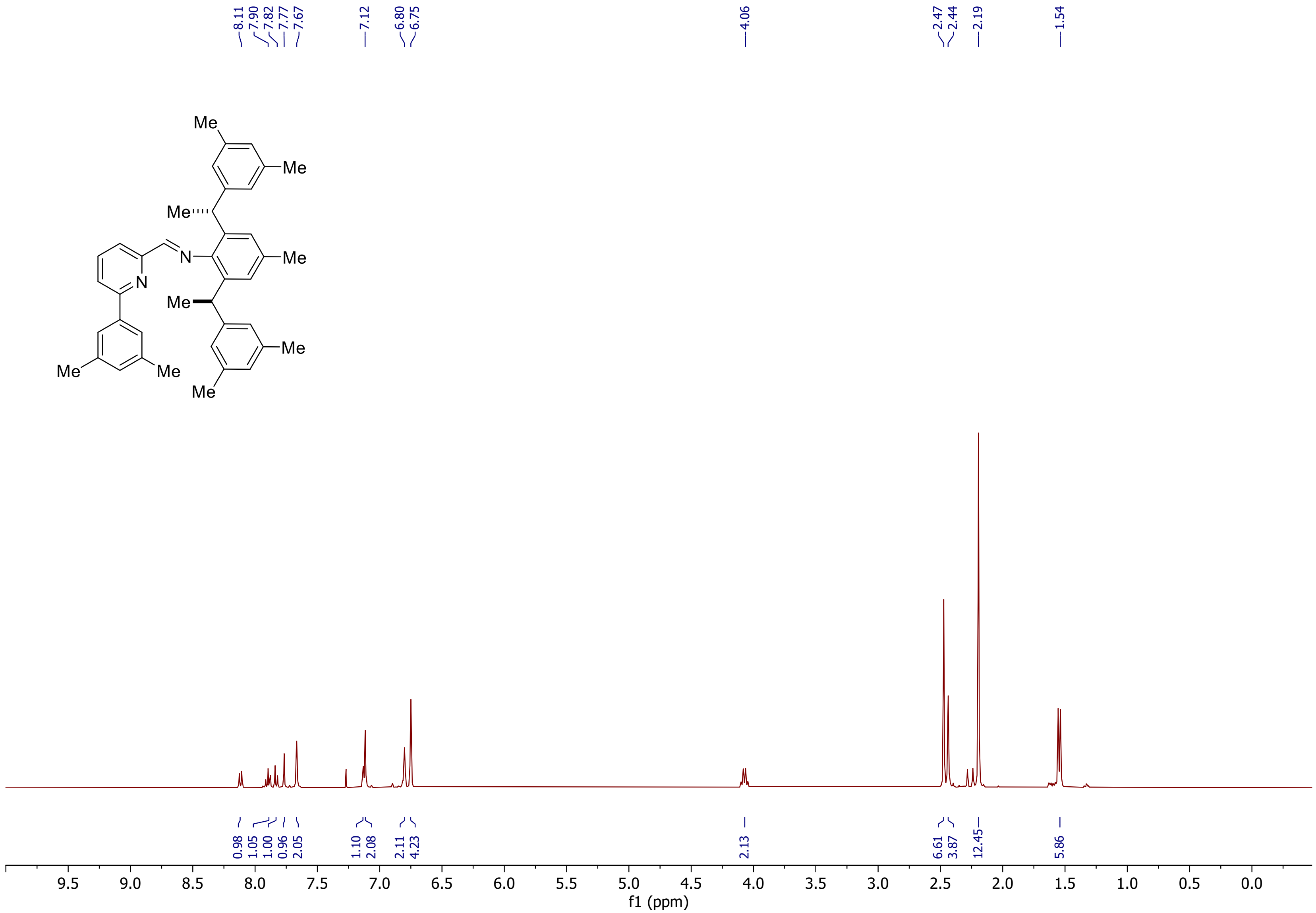

Figure S41. $^{13}C\{^{1}H\}$ NMR spectrum (101 MHz, 298 K, $CDCl_3$) of (*R*,*R*)-**4e**

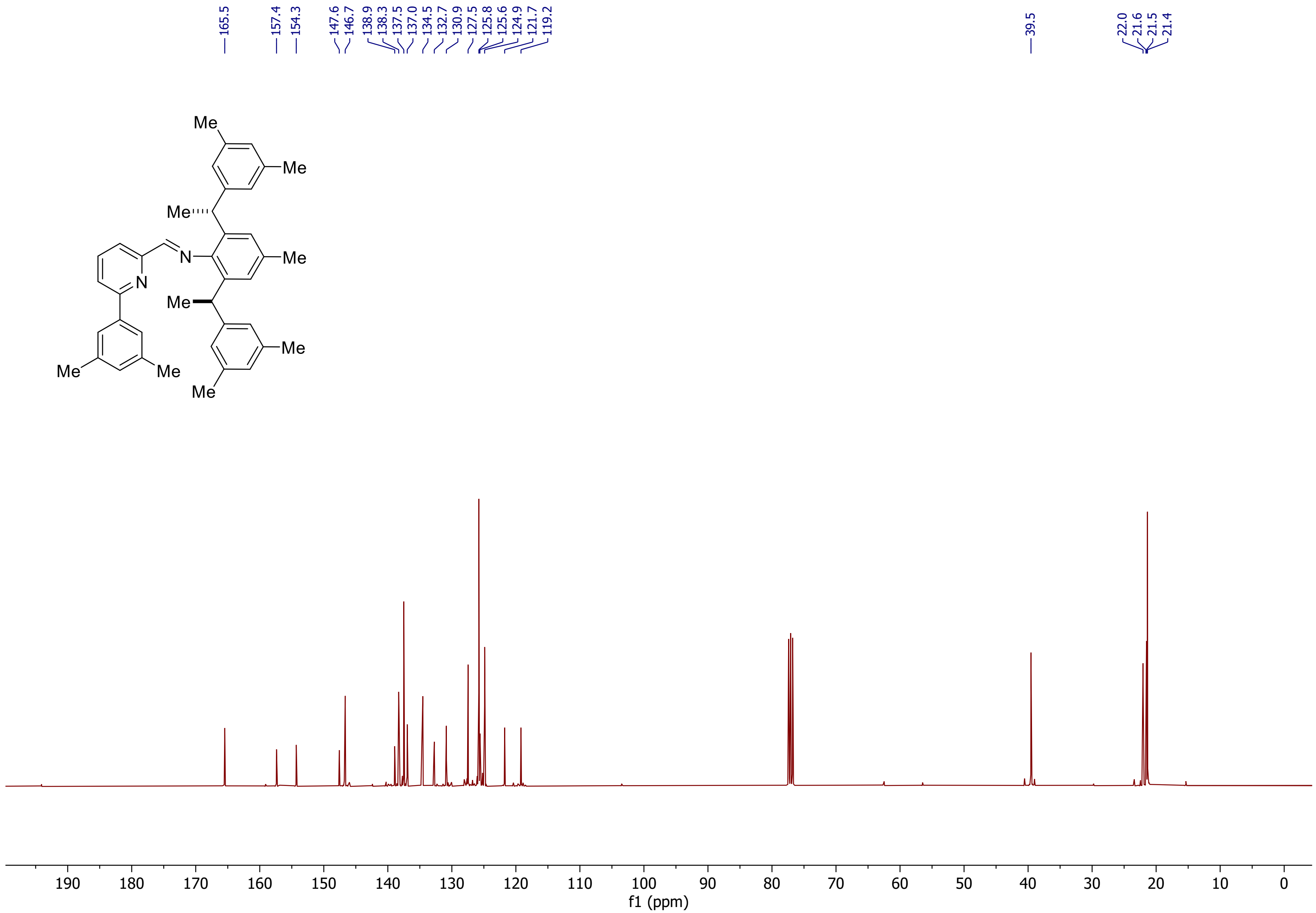

Figure S42. 2D ^{1}H-^{1}H COSY spectrum (298 K, $CDCl_3$) of (*R*,*R*)-**4e**

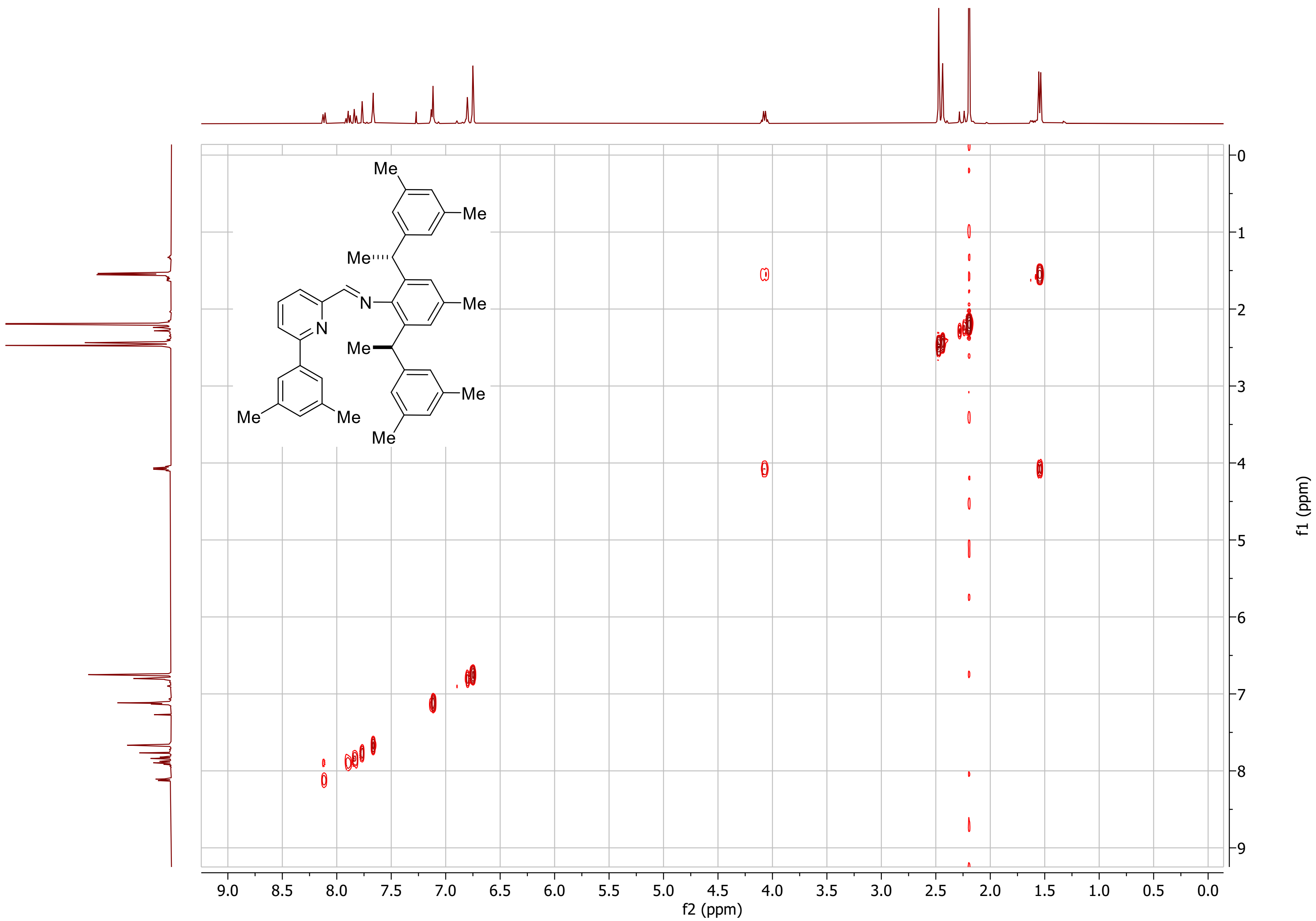

Figure S43. 2D ^{1}H-^{13}C HSQC spectrum (298 K, $CDCl_3$) of (*R*,*R*)-**4e**

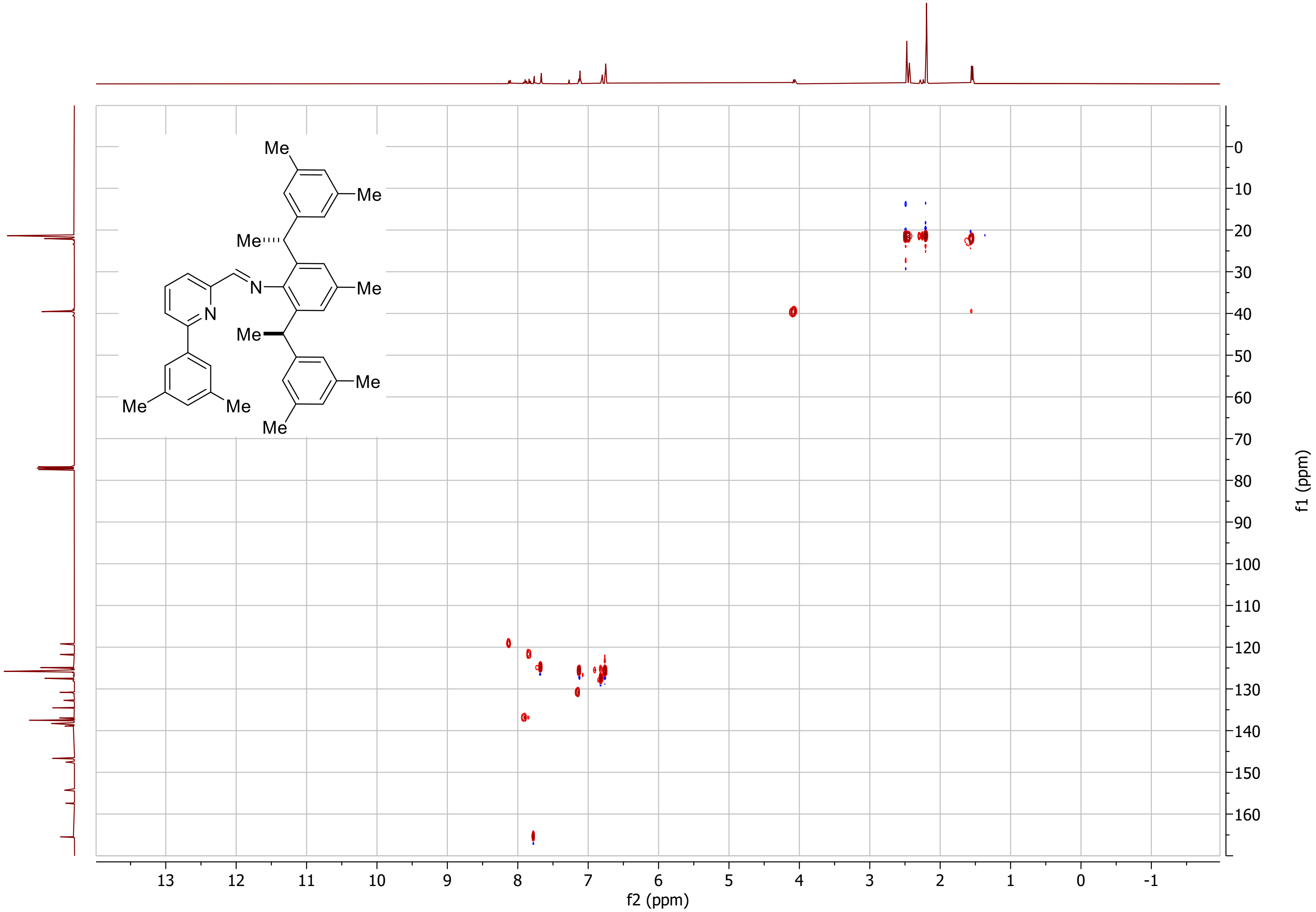

Figure S44. ^{1}H NMR spectrum (400 MHz, 298 K, $CDCl_3$) of (*R*,*R*)-**4f**

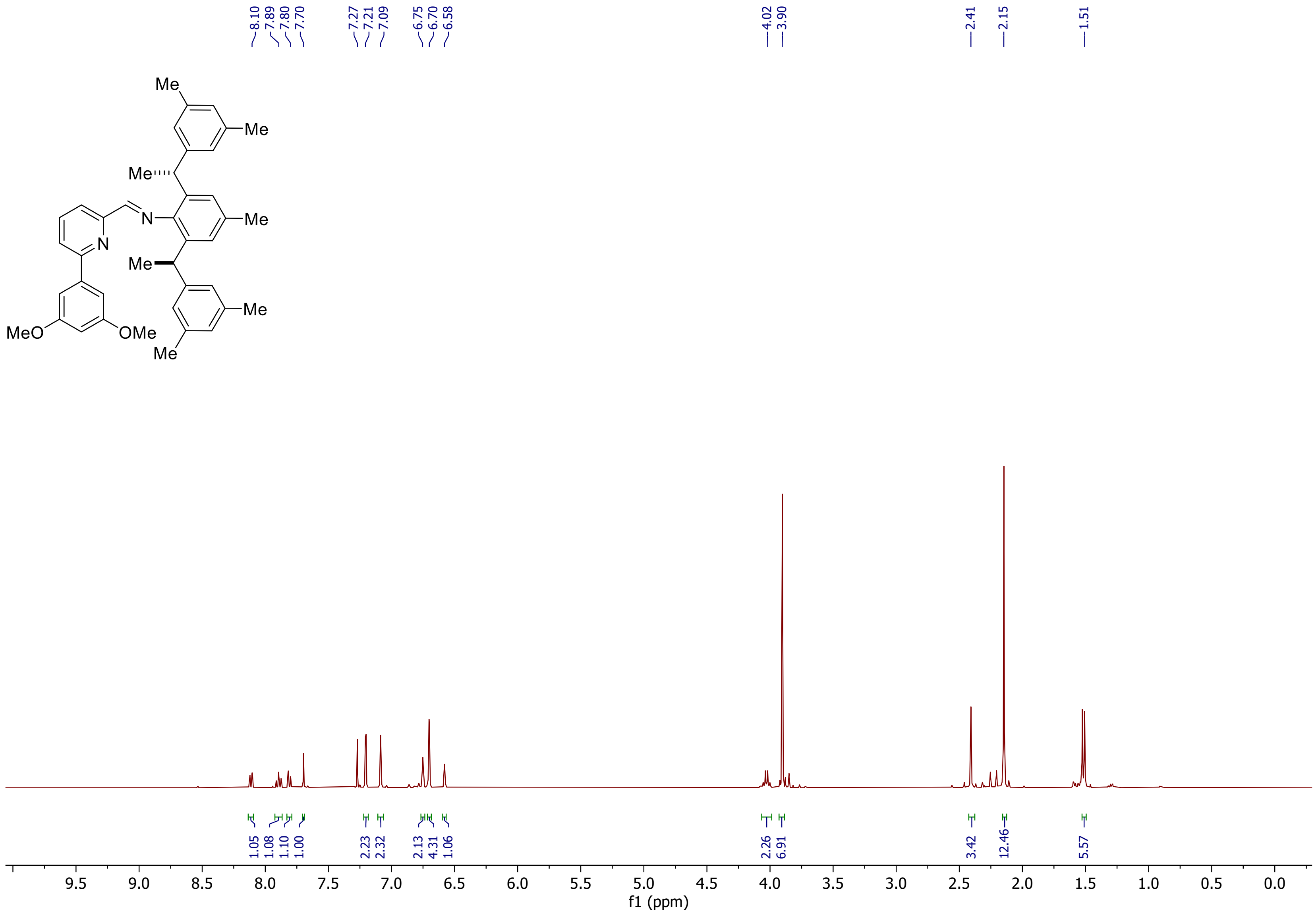

Figure S45. $^{13}C\{^{1}H\}$ NMR spectrum (101 MHz, 298 K, $CDCl_3$) of (*R*,*R*)-**4f**

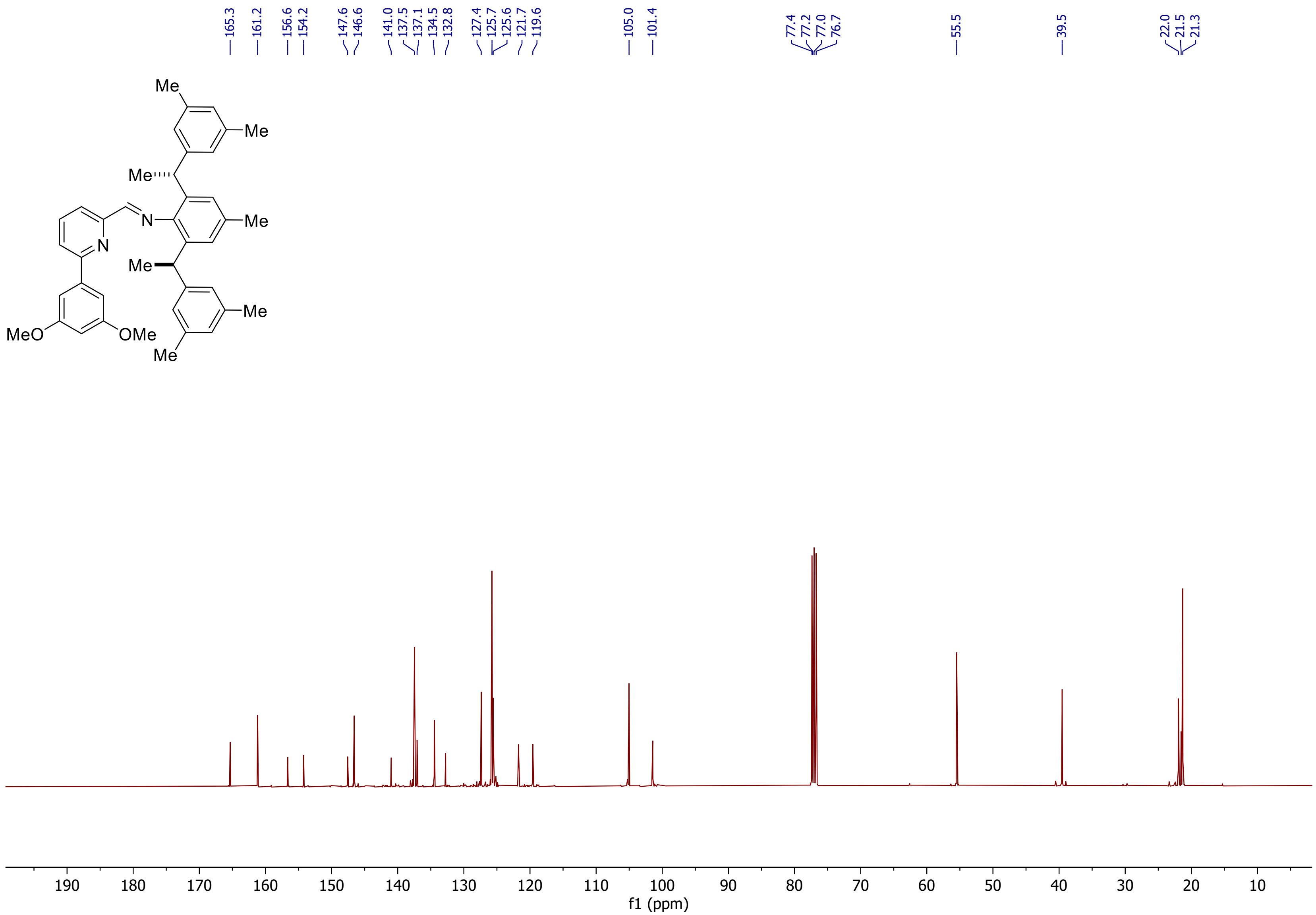

Figure S46. 2D ^{1}H-^{1}H COSY spectrum (298 K, $CDCl_3$) of (*R*,*R*)-**4f**

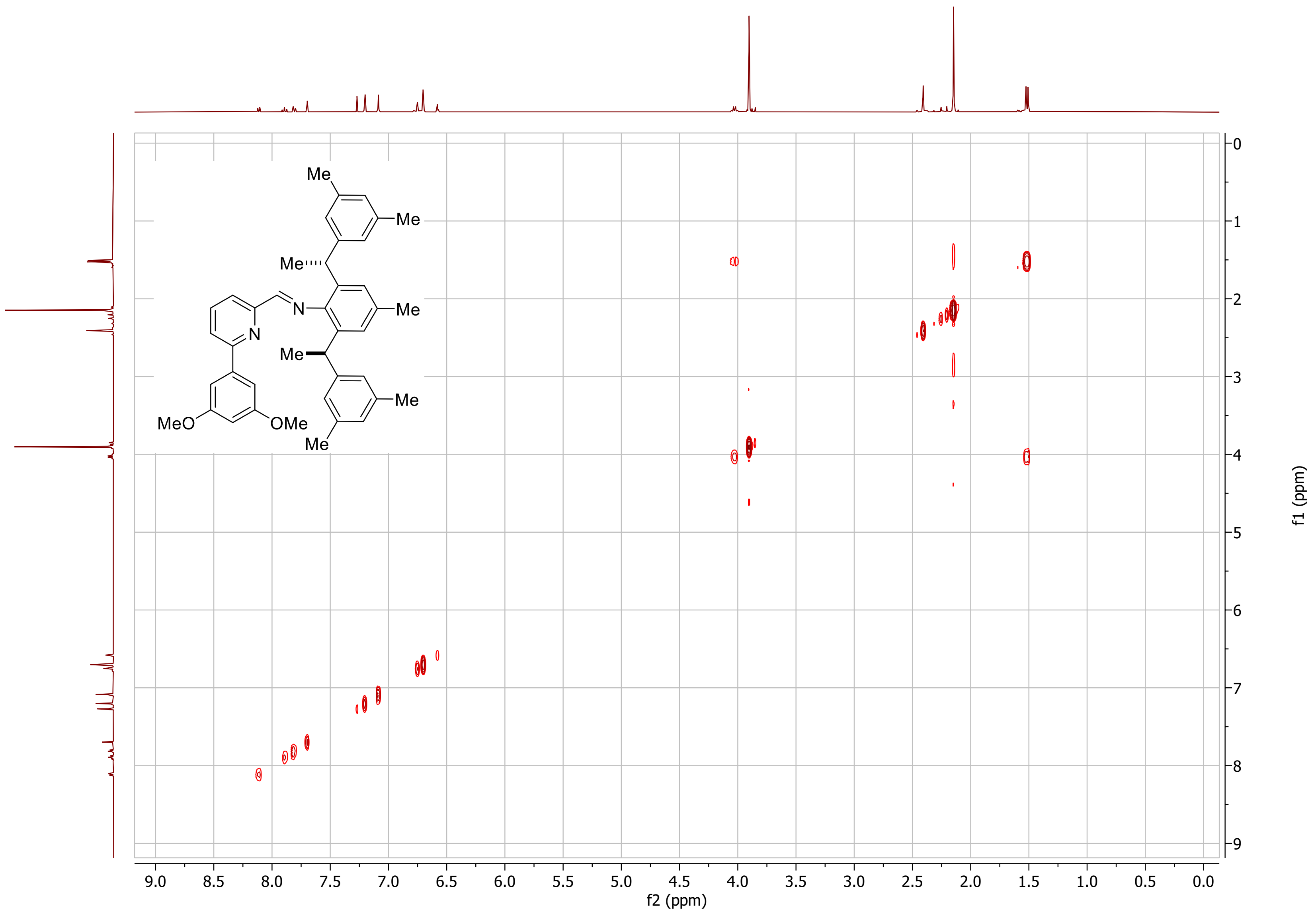

Figure S47. 2D ^{1}H-^{13}C HSQC spectrum (298 K, $CDCl_3$) of (*R*,*R*)-**4f**

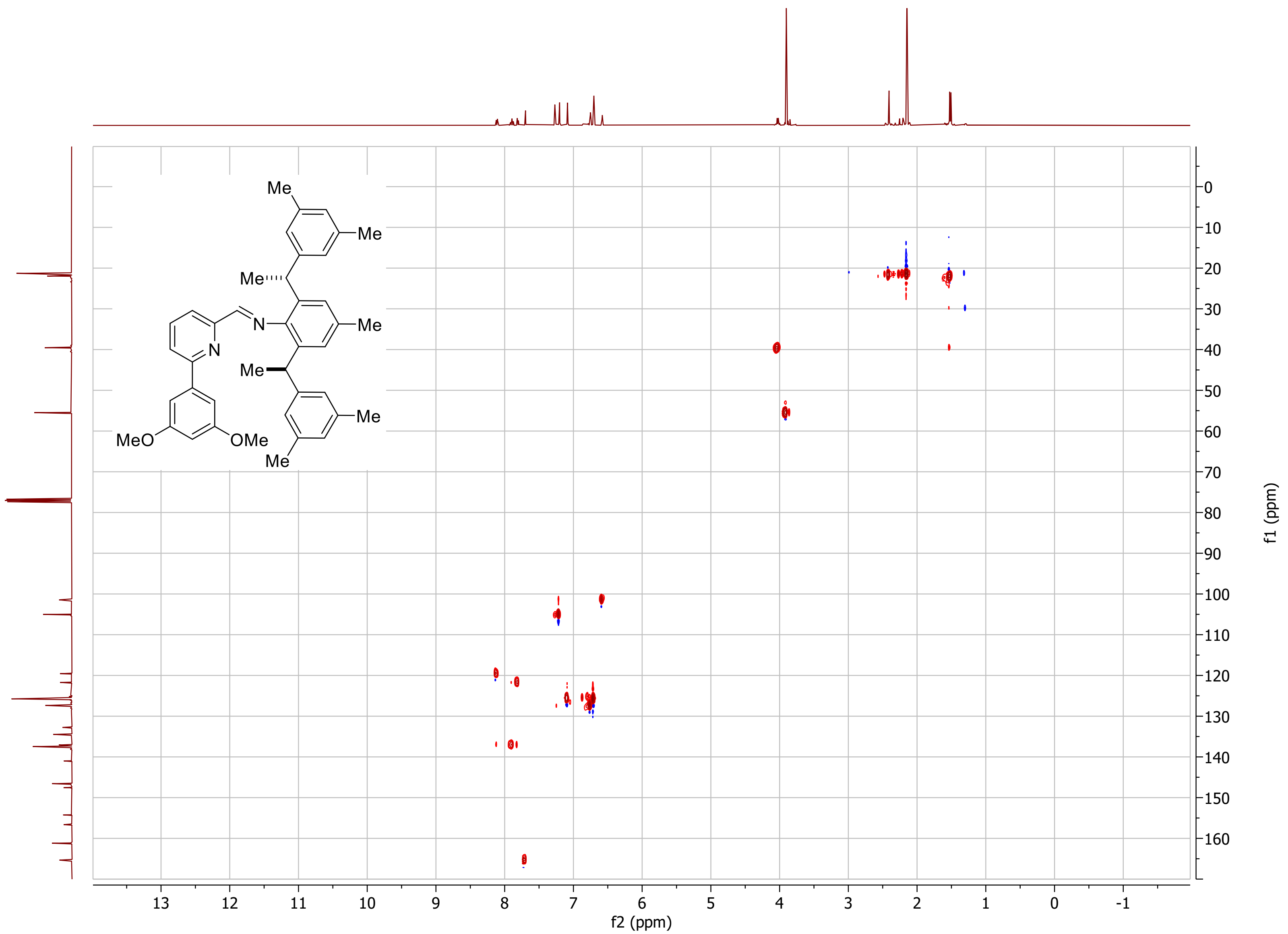

Figure S48. ^{1}H NMR spectrum (400 MHz, 298 K, $CDCl_3$) of (*R*,*R*)-**4h**

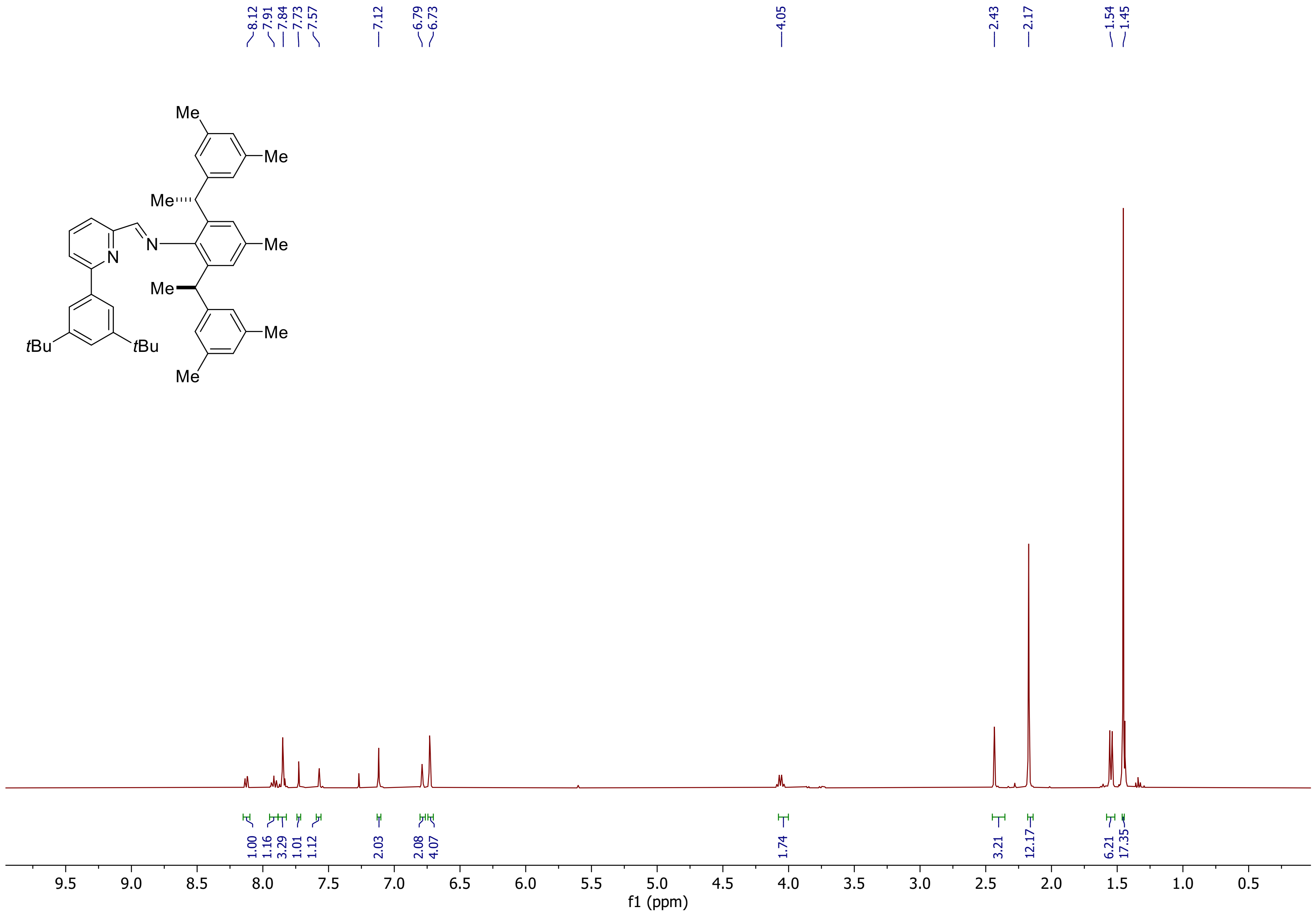

Figure S49. $^{13}C\{^1H\}$ NMR spectrum (101 MHz, 298 K, $CDCl_3$) of (*R*,*R*)-**4h**

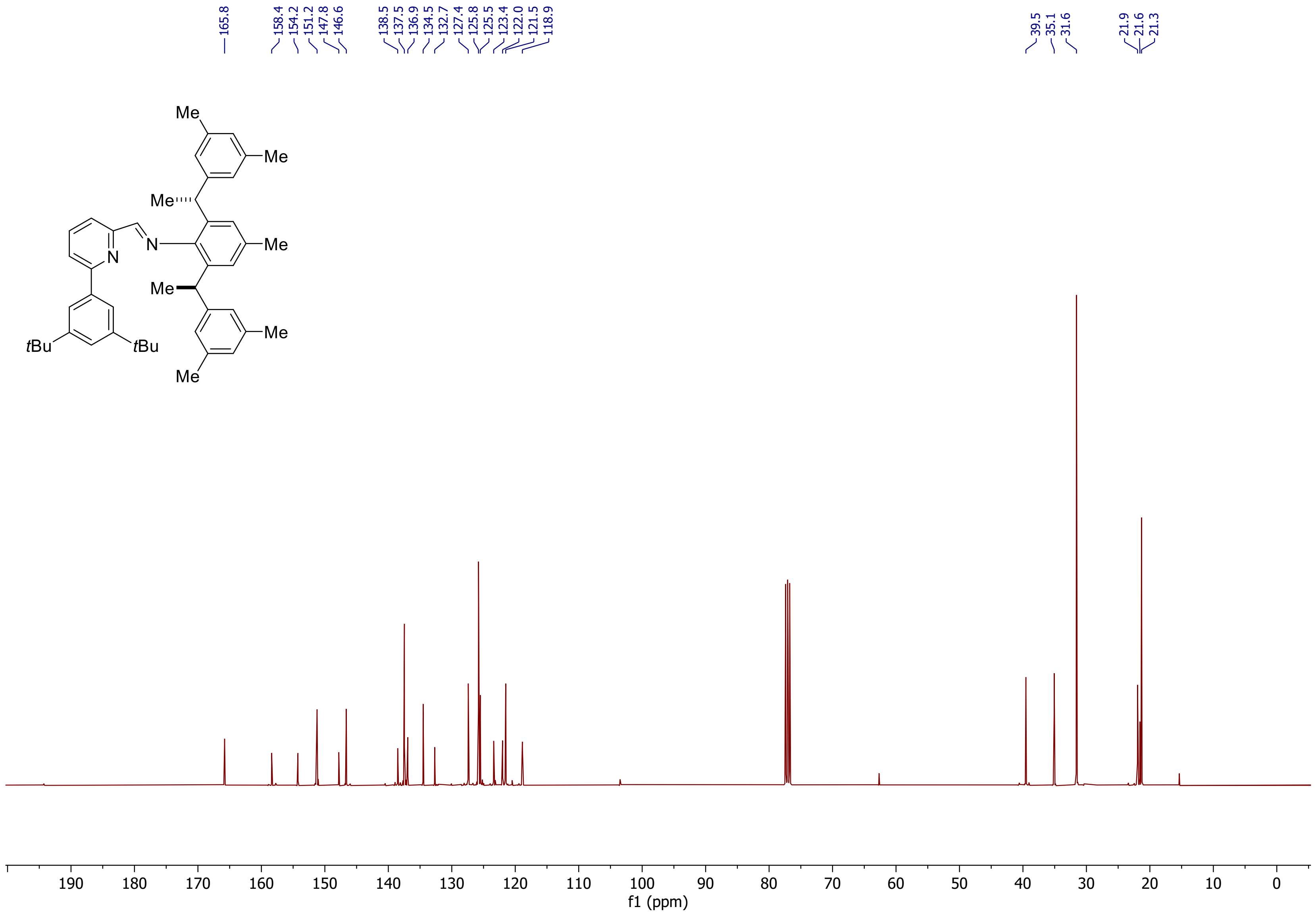

Figure S50. 2D ^{1}H-^{1}H COSY spectrum (298 K, $CDCl_3$) of (*R*,*R*)-**4h**

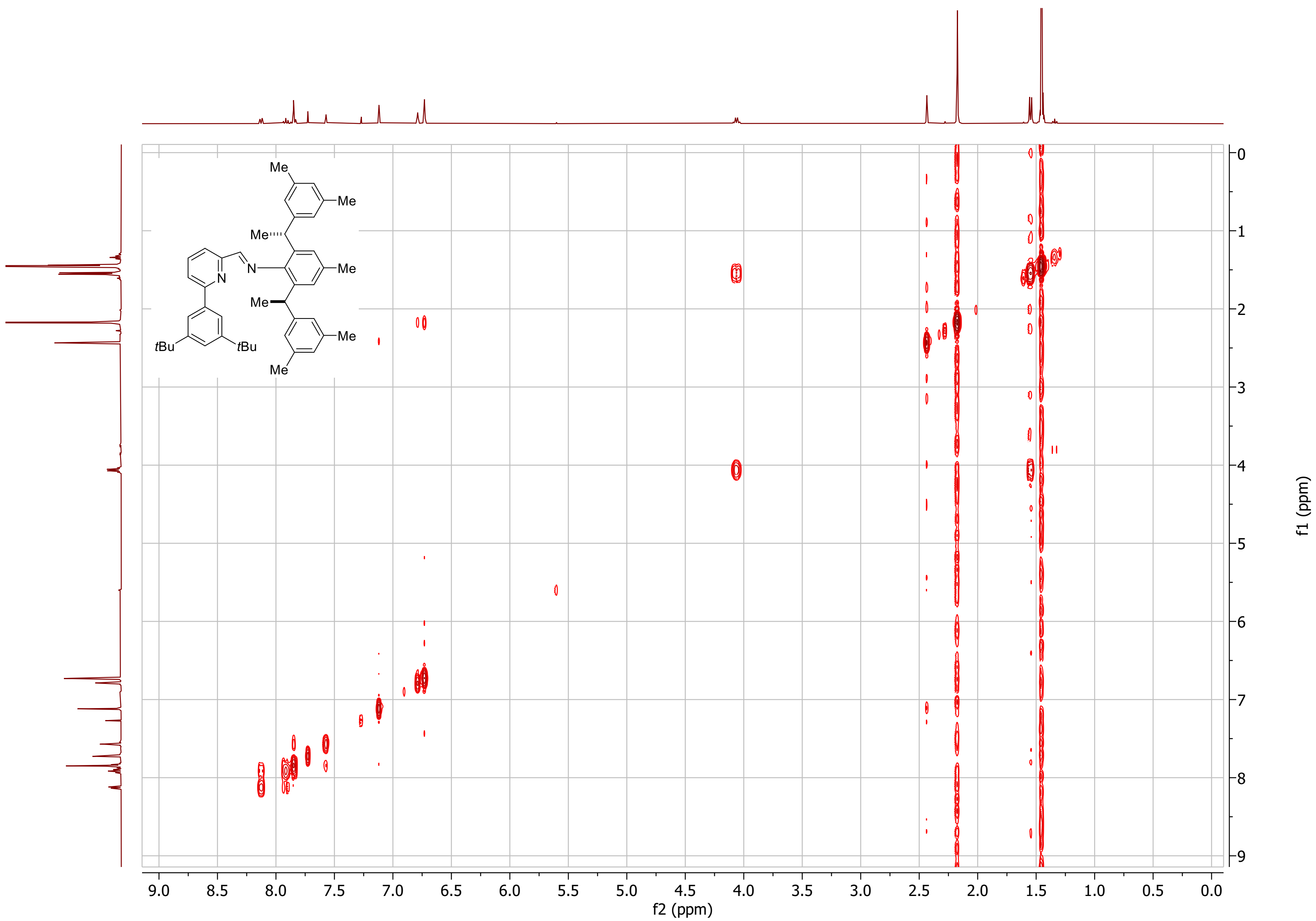

Figure S51. 2D ^{1}H-^{13}C HSQC spectrum (298 K, $CDCl_3$) of (*R*,*R*)-**4h**

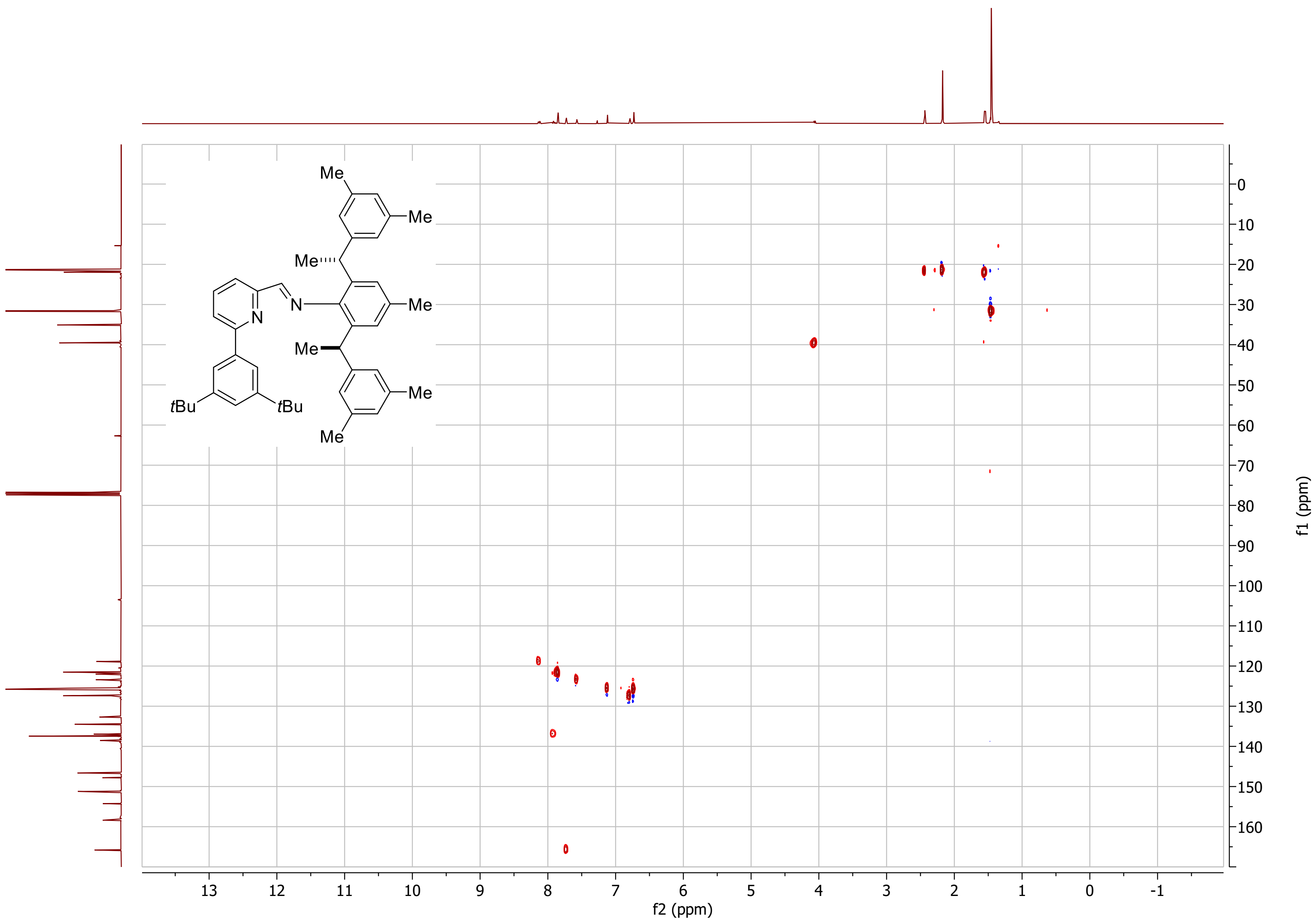

Figure S52. ^{1}H NMR spectrum (400 MHz, 298 K, $CDCl_3$) of (*R*,*R*)-**4i**

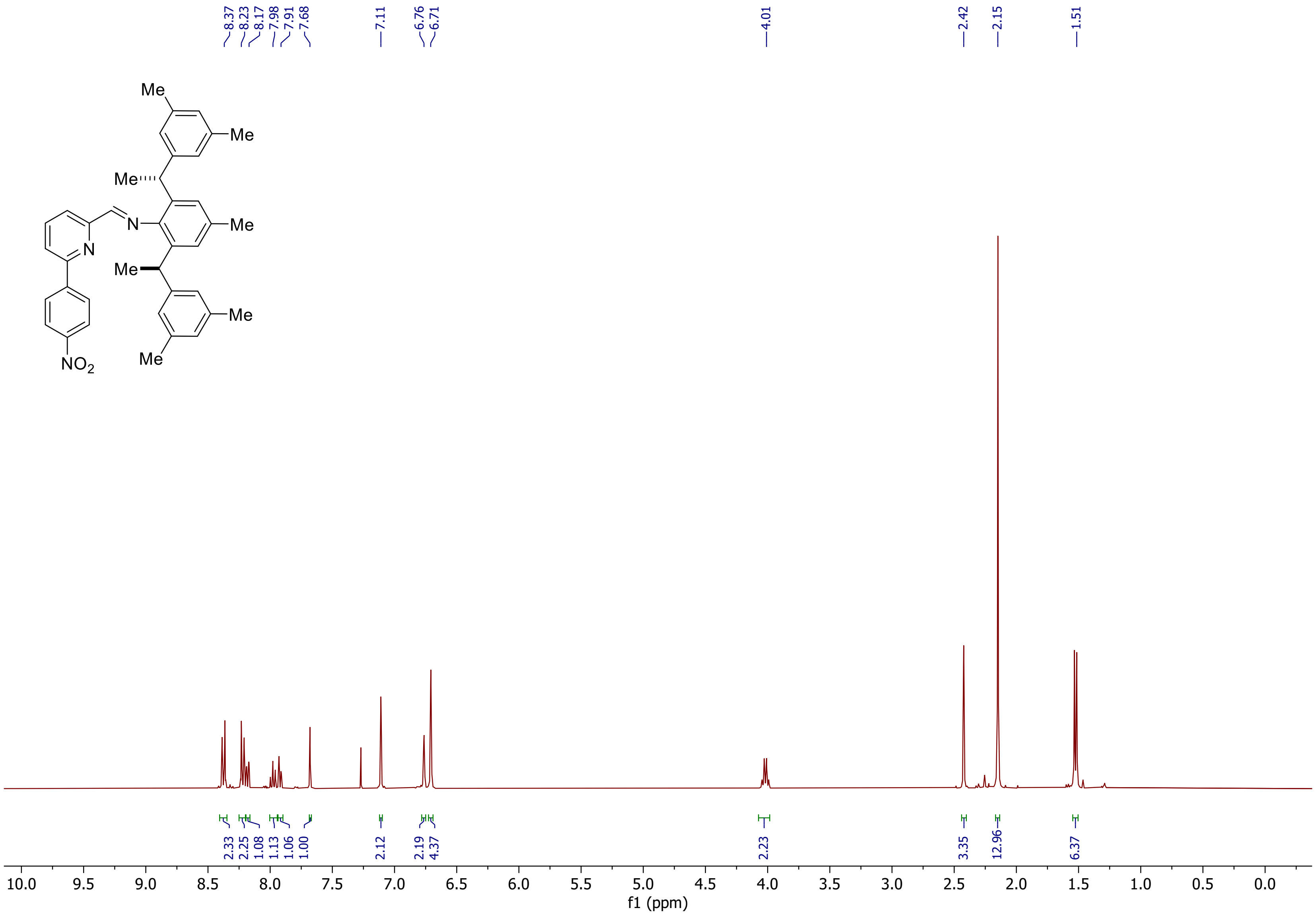

Figure S53. $^{13}C\{^{1}H\}$ NMR spectrum (101 MHz, 298 K, $CDCl_3$) of (*R*,*R*)-**4i**

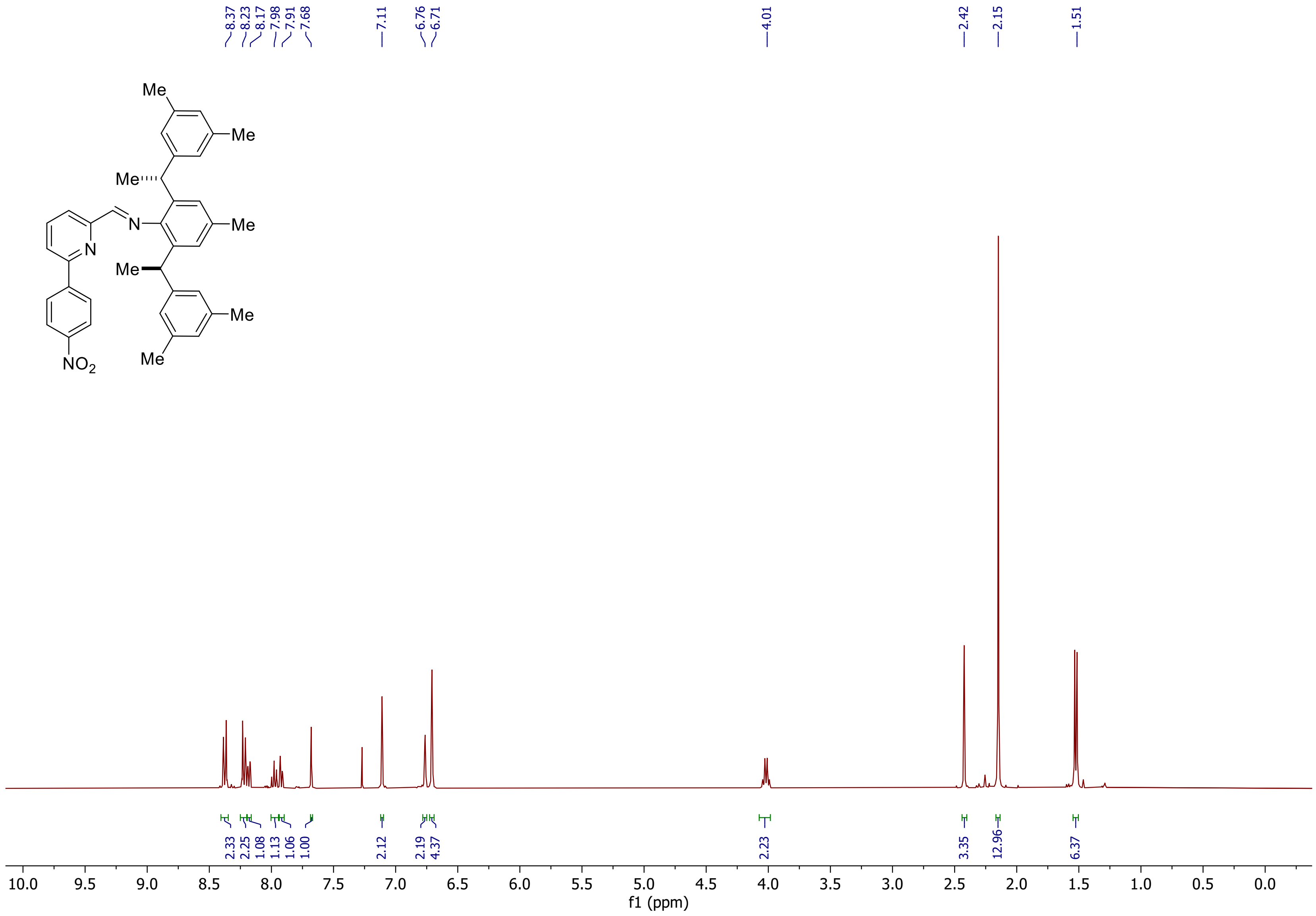

Figure S54. 2D ^{1}H-^{1}H COSY spectrum (298 K, $CDCl_3$) of (*R*,*R*)-**4i**

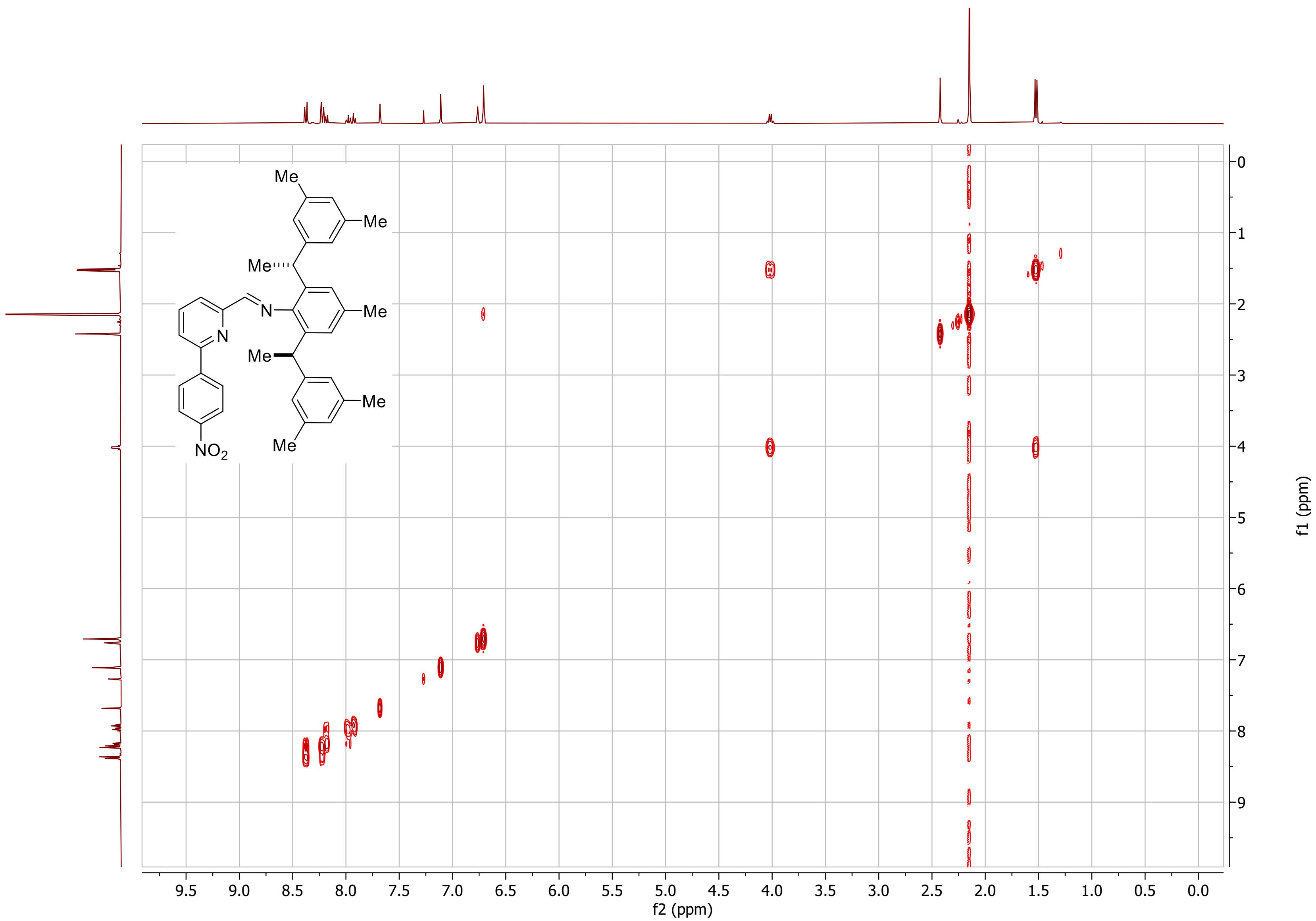

Figure S55. 2D ^{1}H-^{13}C HSQC spectrum (298 K, $CDCl_3$) of (*R*,*R*)-**4i**

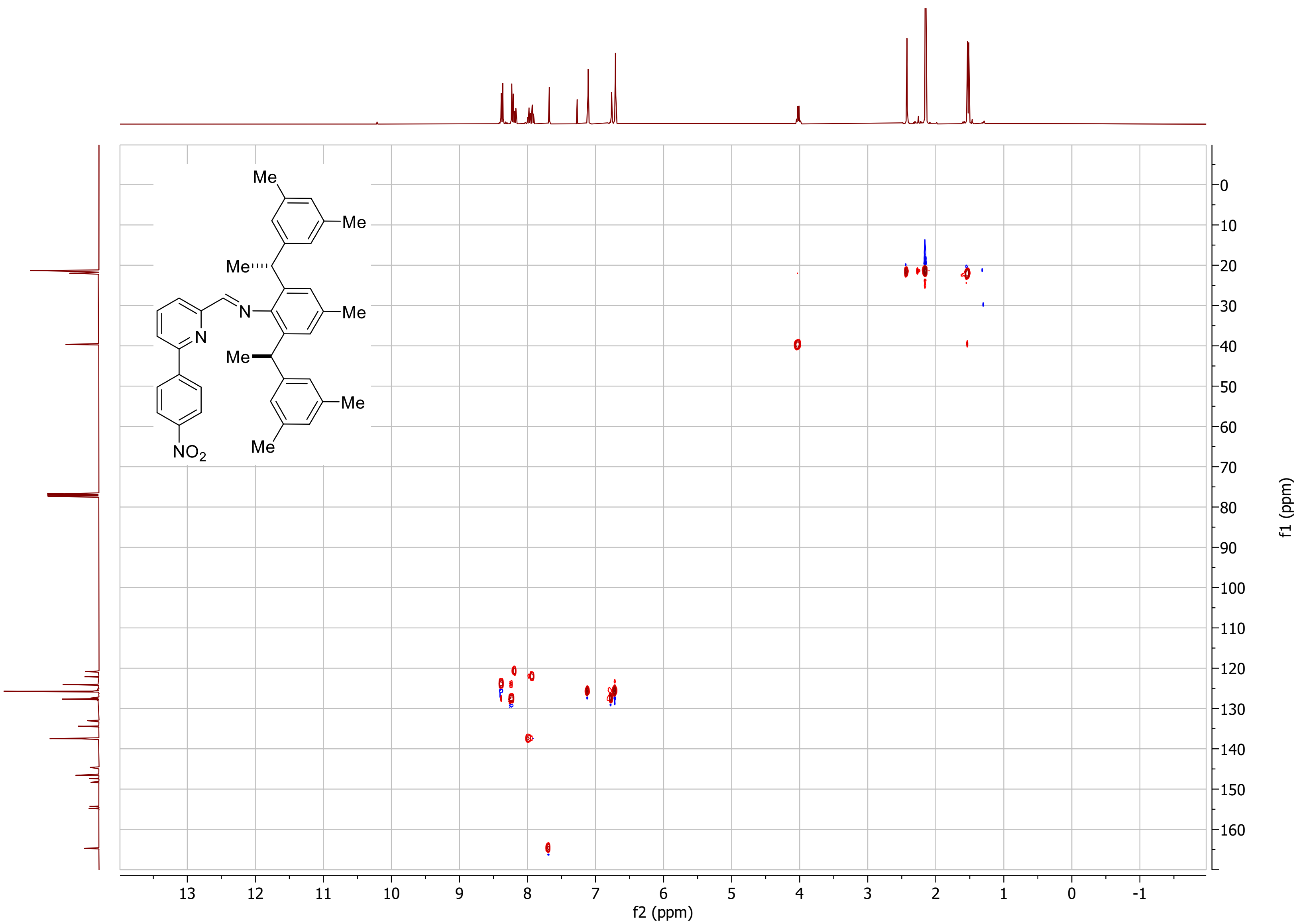

Figure S56. ^{1}H NMR spectrum (400 MHz, 298 K, $CDCl_3$) of (*R*,*R*)-**4j**

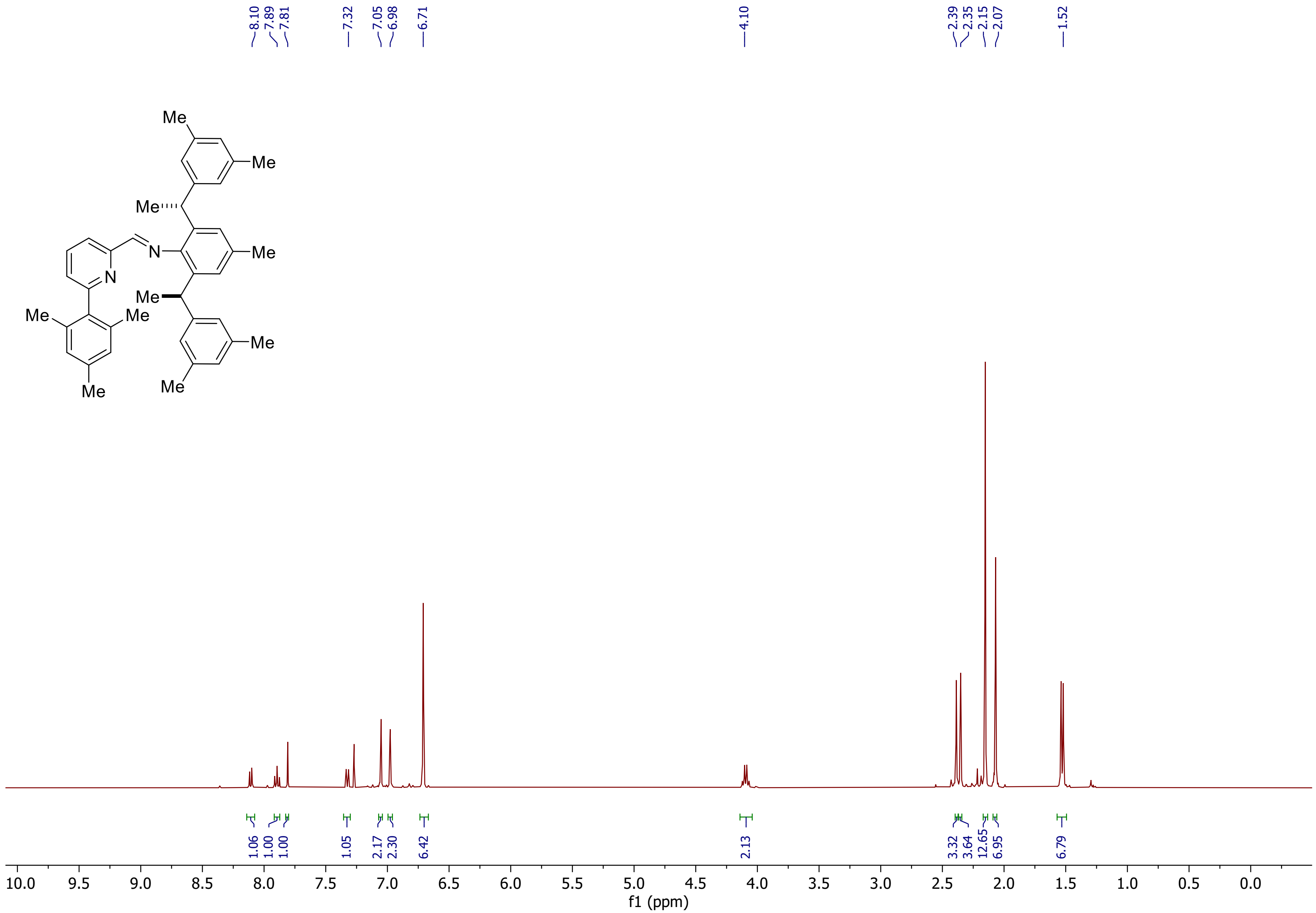

Figure S57. $^{13}C\{^{1}H\}$ NMR spectrum (101 MHz, 298 K, $CDCl_3$) of (*R*,*R*)-**4j**

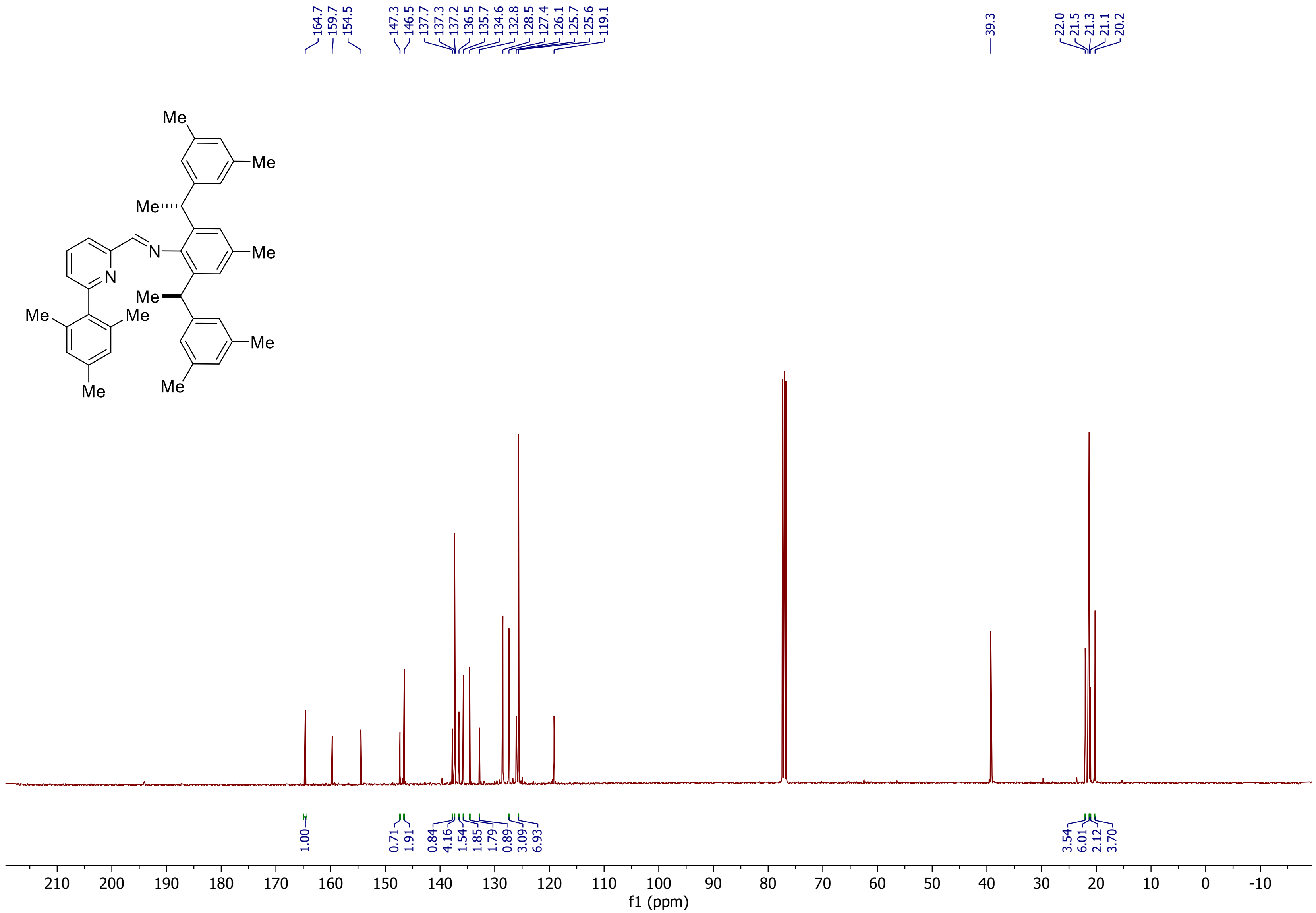

Figure S58. 2D ^1H-^1H COSY spectrum (298 K, $CDCl_3$) of (*R*,*R*)-**4j**

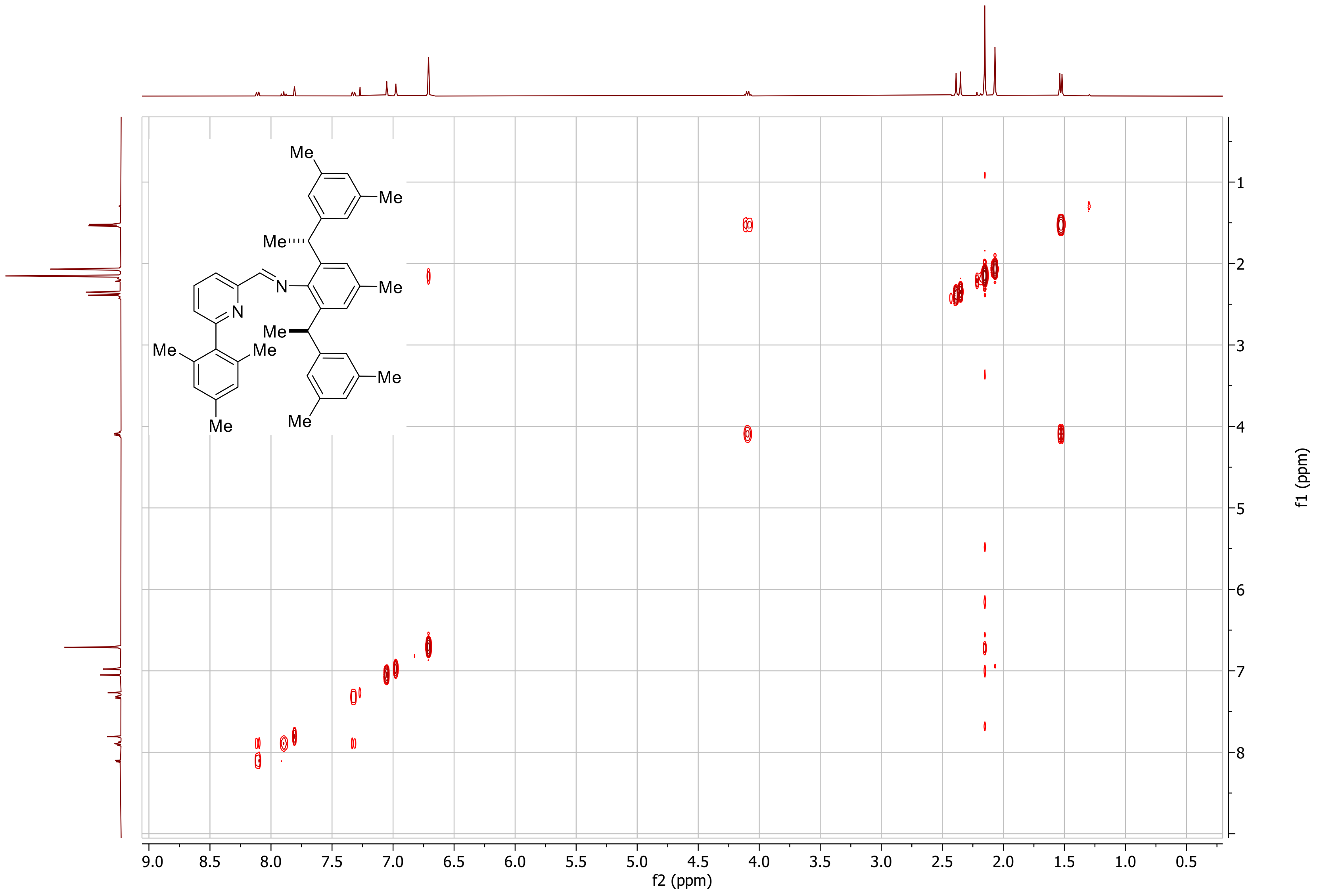

Figure S59. 2D ^{1}H-^{13}C HSQC spectrum (298 K, $CDCl_3$) of (*R*,*R*)-**4j**

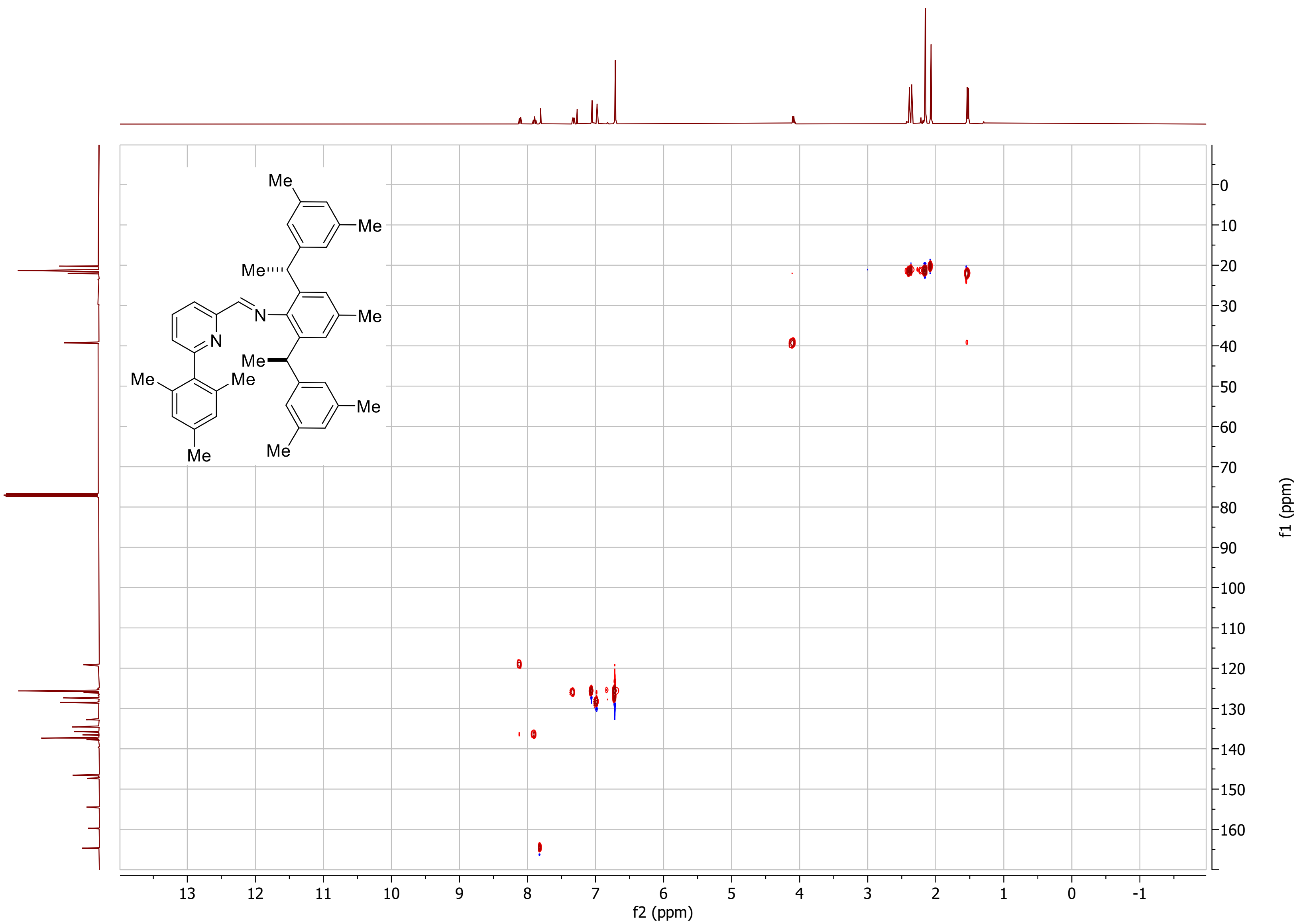

Figure S60. ^{1}H NMR spectrum (400 MHz, 298 K, $CDCl_3$) of (*R*,*R*)-**5a**

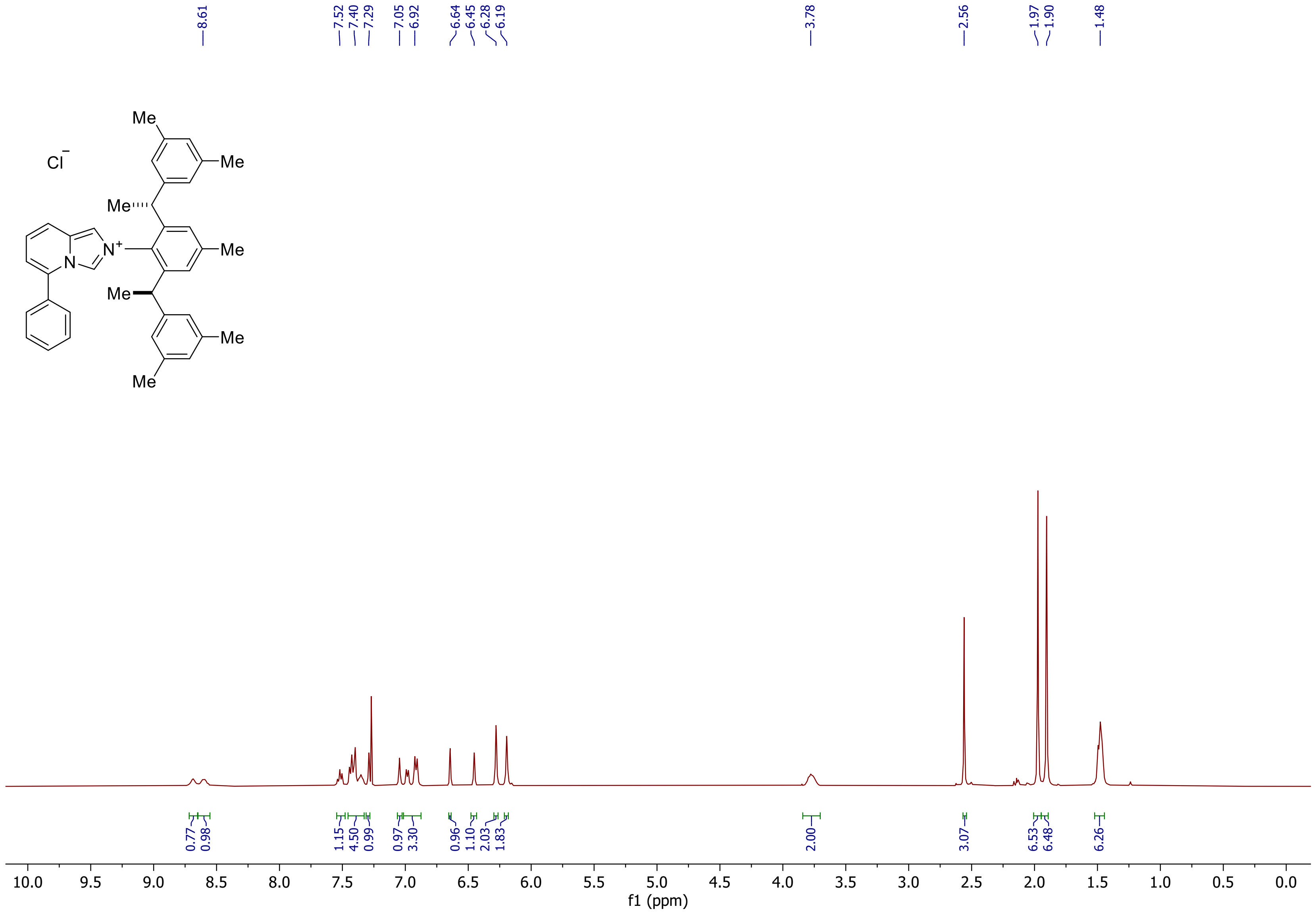

Figure S61. $^{13}C\{^1H\}$ NMR spectrum (101 MHz, 298 K, $CDCl_3$) of (*R*,*R*)-**5a**

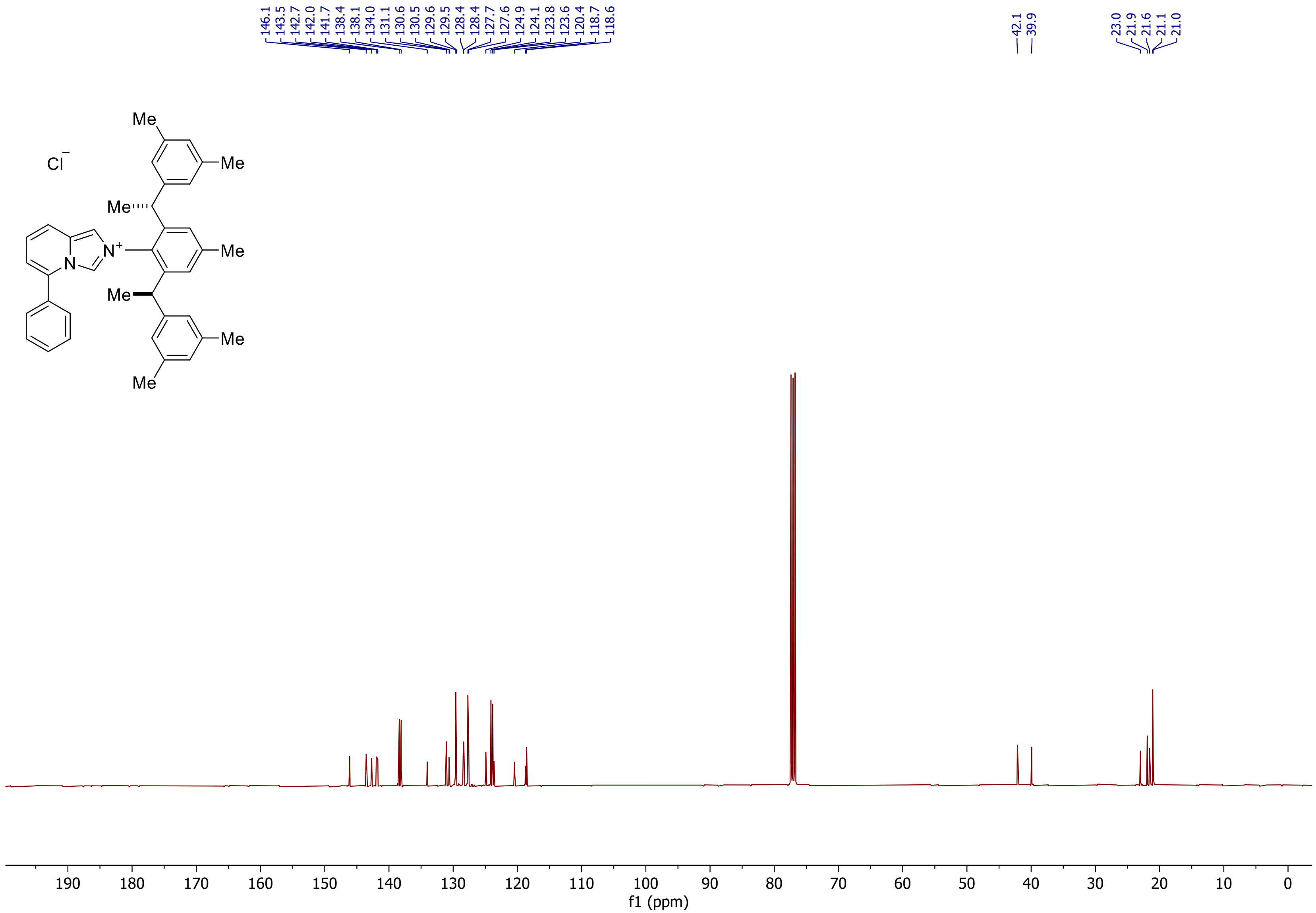

Figure S62. 2D ^{1}H-^{1}H COSY spectrum (298 K, $CDCl_3$) of (*R*,*R*)-**5a**

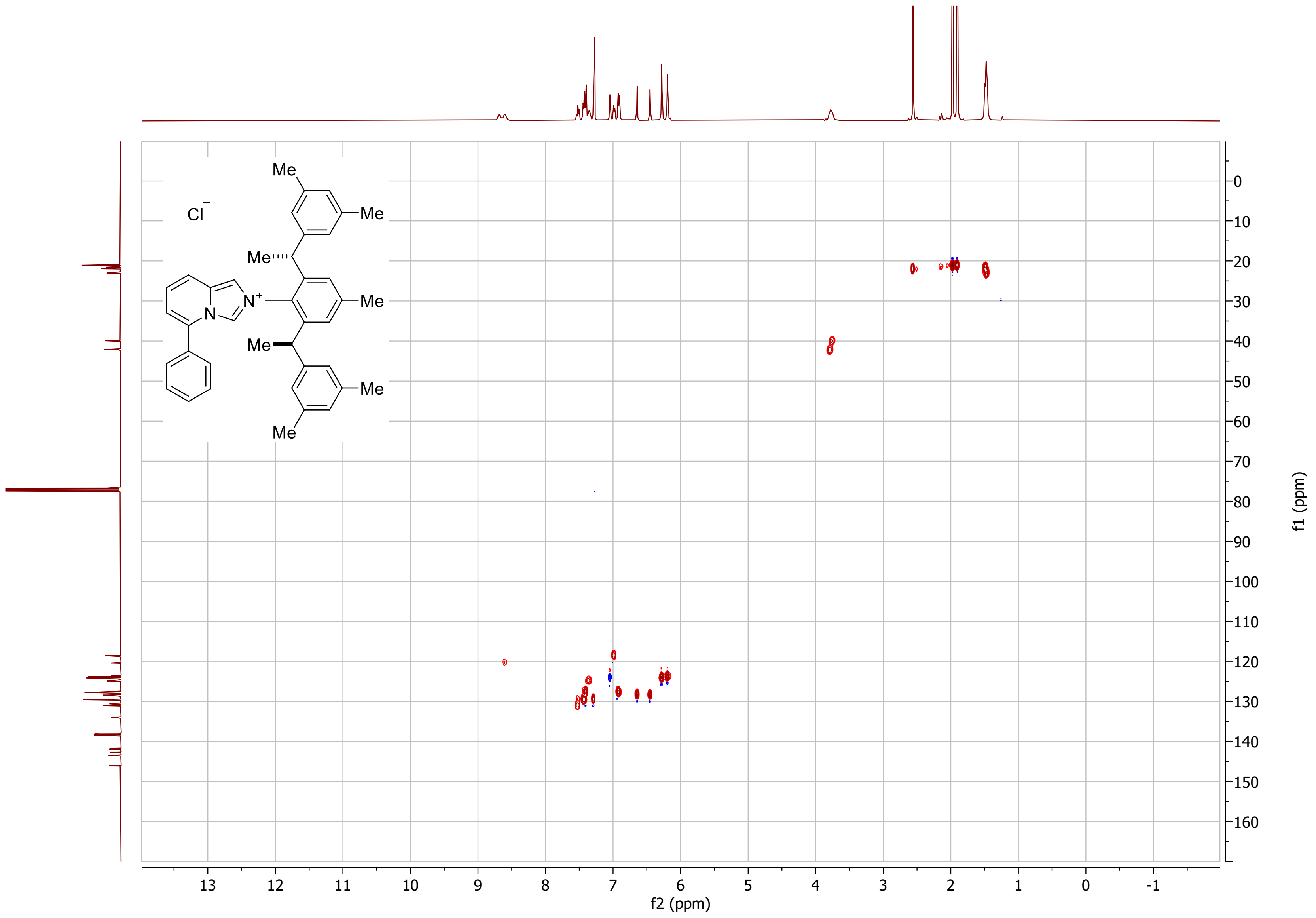

Figure S63. 2D ^{1}H-^{13}C HSQC spectrum (298 K, $CDCl_3$) of (*R*,*R*)-**5a**

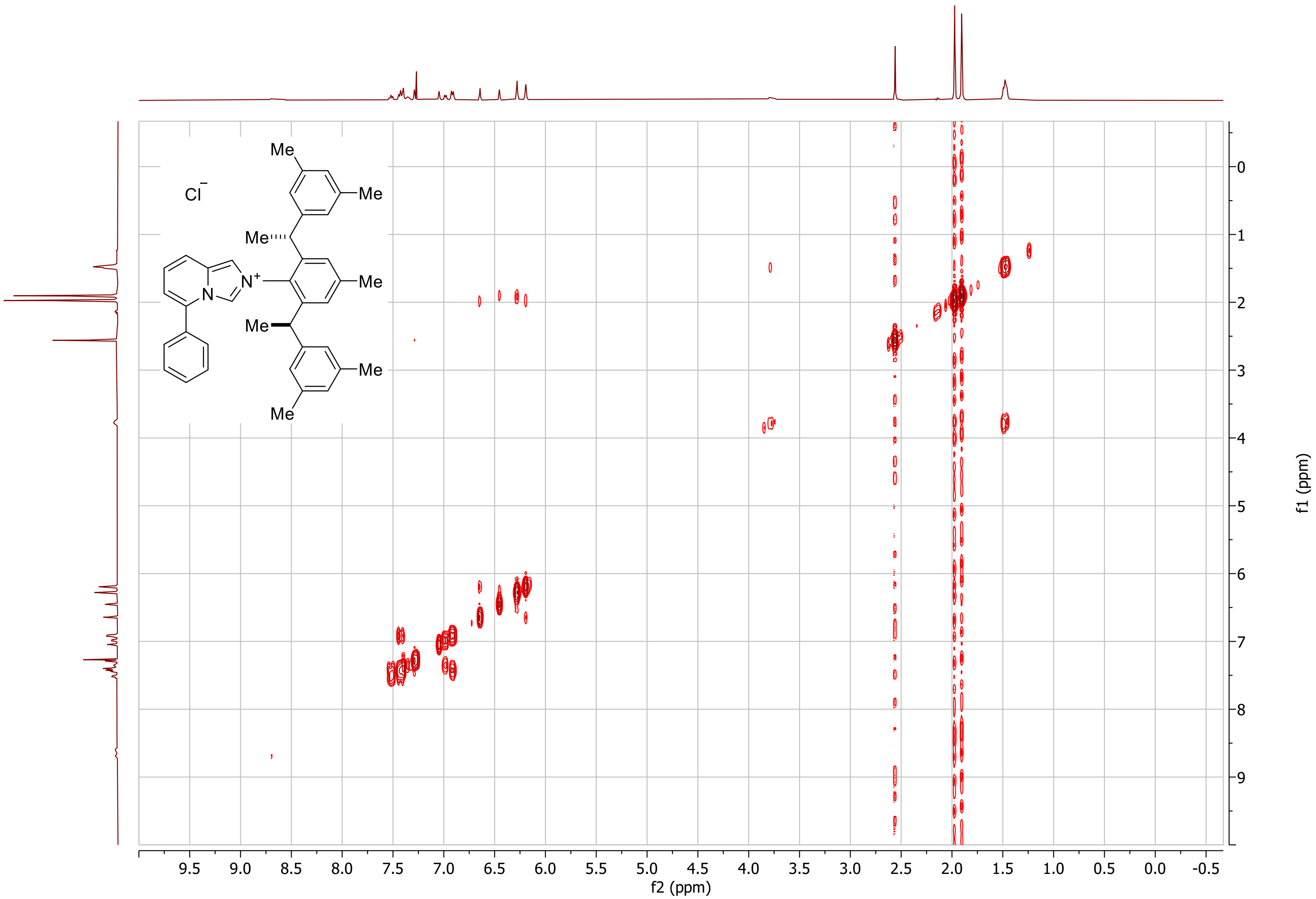

Figure S64. ^{1}H NMR spectrum (400 MHz, 298 K, $CDCl_3$) of (*R*,*R*)-**5b**

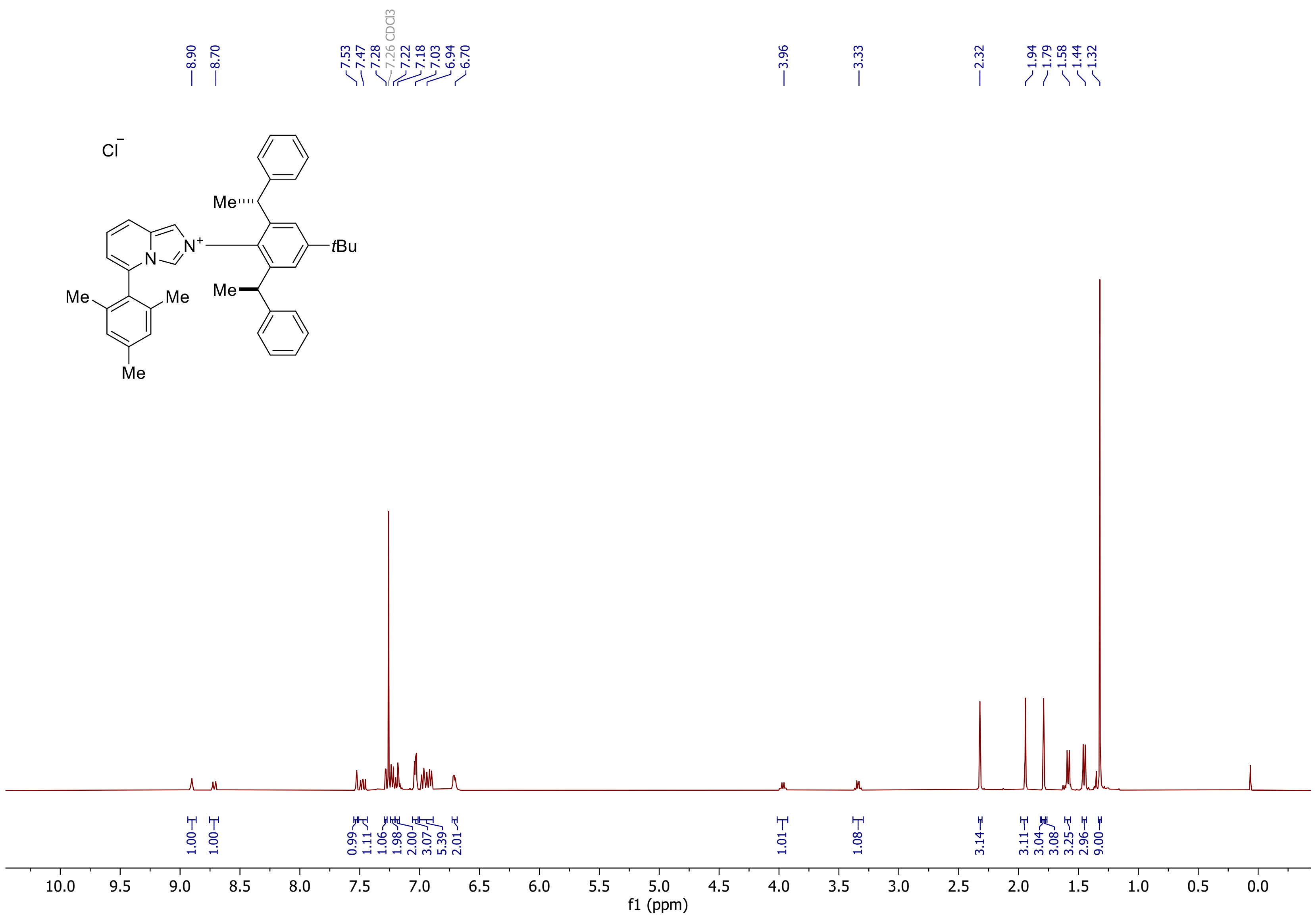

Figure S65. $^{13}C\{^{1}H\}$ NMR spectrum (101 MHz, 298 K, $CDCl_3$) of (*R*,*R*)-**5b**

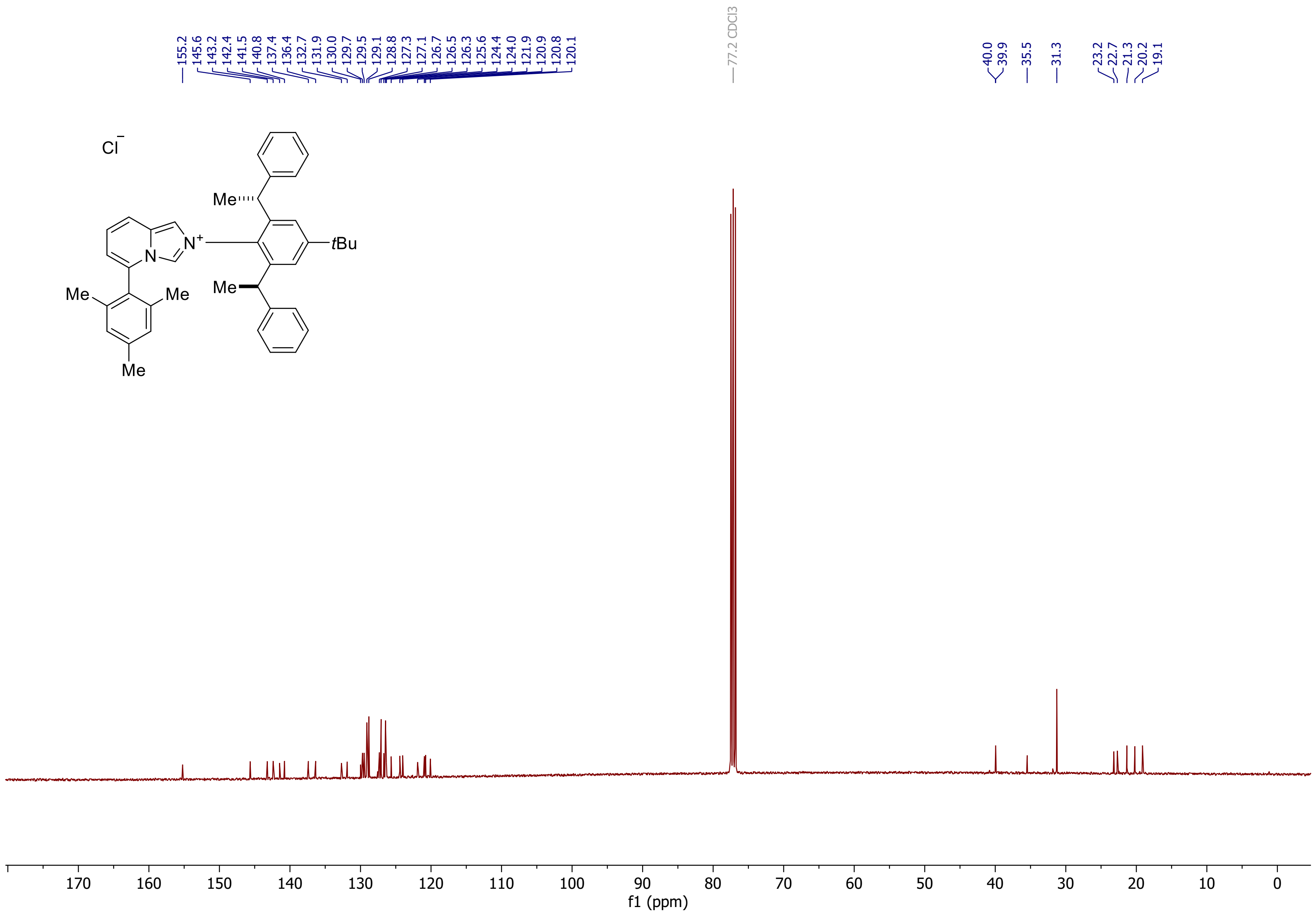

Figure S66. 2D ^{1}H-^{1}H COSY spectrum (298 K, $CDCl_3$) of (*R*,*R*)-**5b**

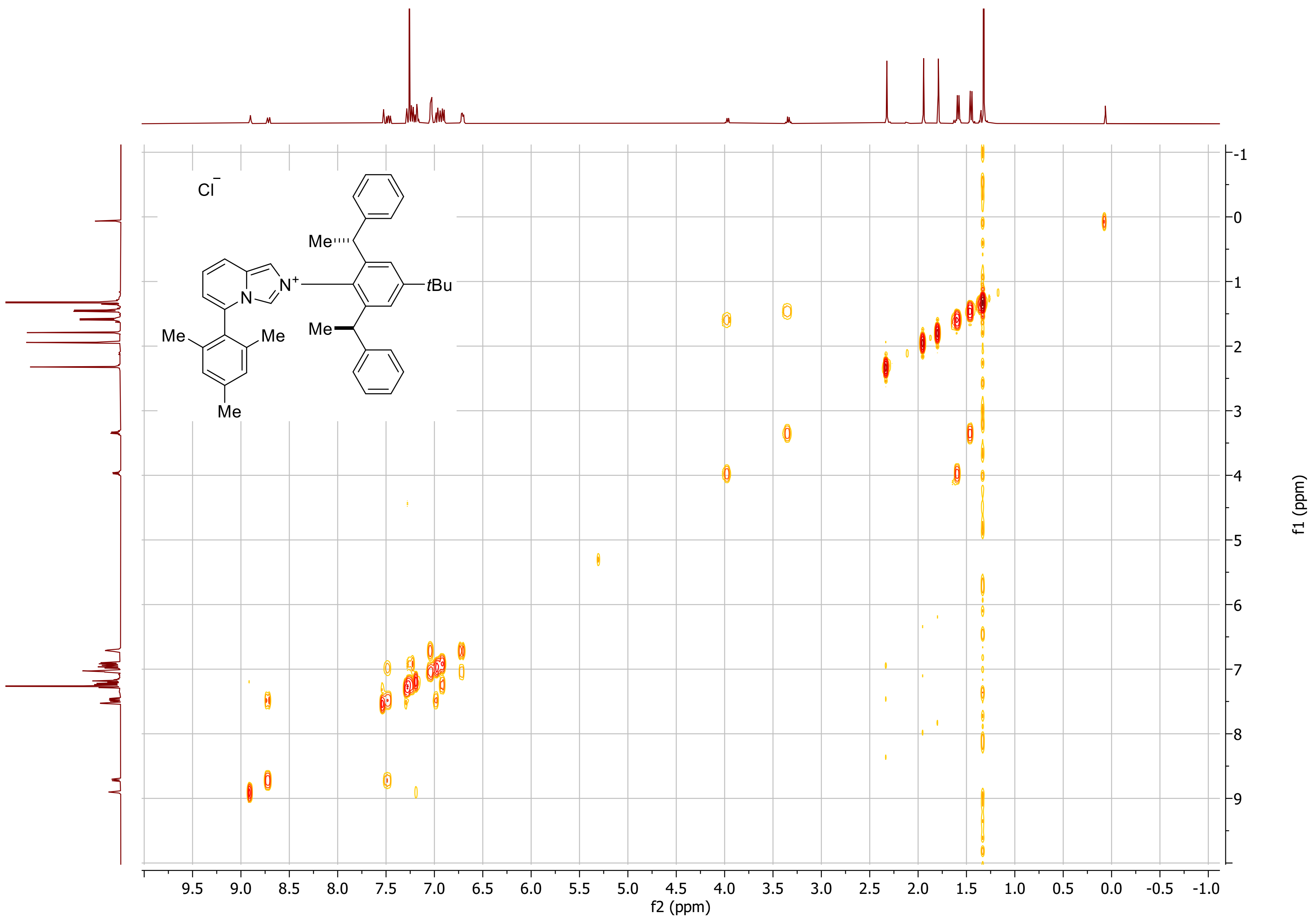

Figure S67. 2D ^{1}H-^{13}C HSQC spectrum (298 K, $CDCl_3$) of (*R*,*R*)-**5b**

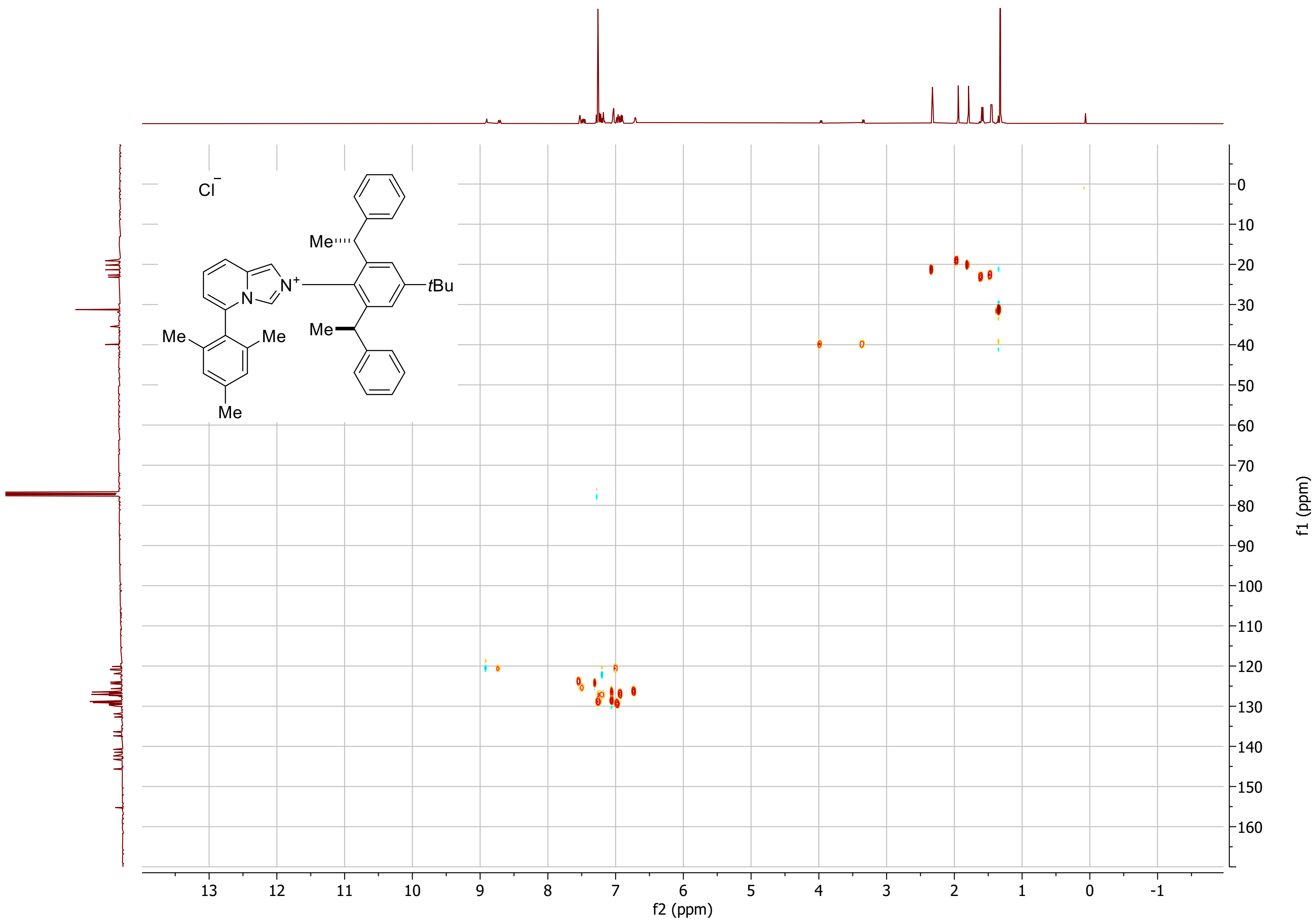

Figure S68. ^{1}H NMR spectrum (400 MHz, 298 K, $CDCl_3$) of (*R*,*R*)-**5c**

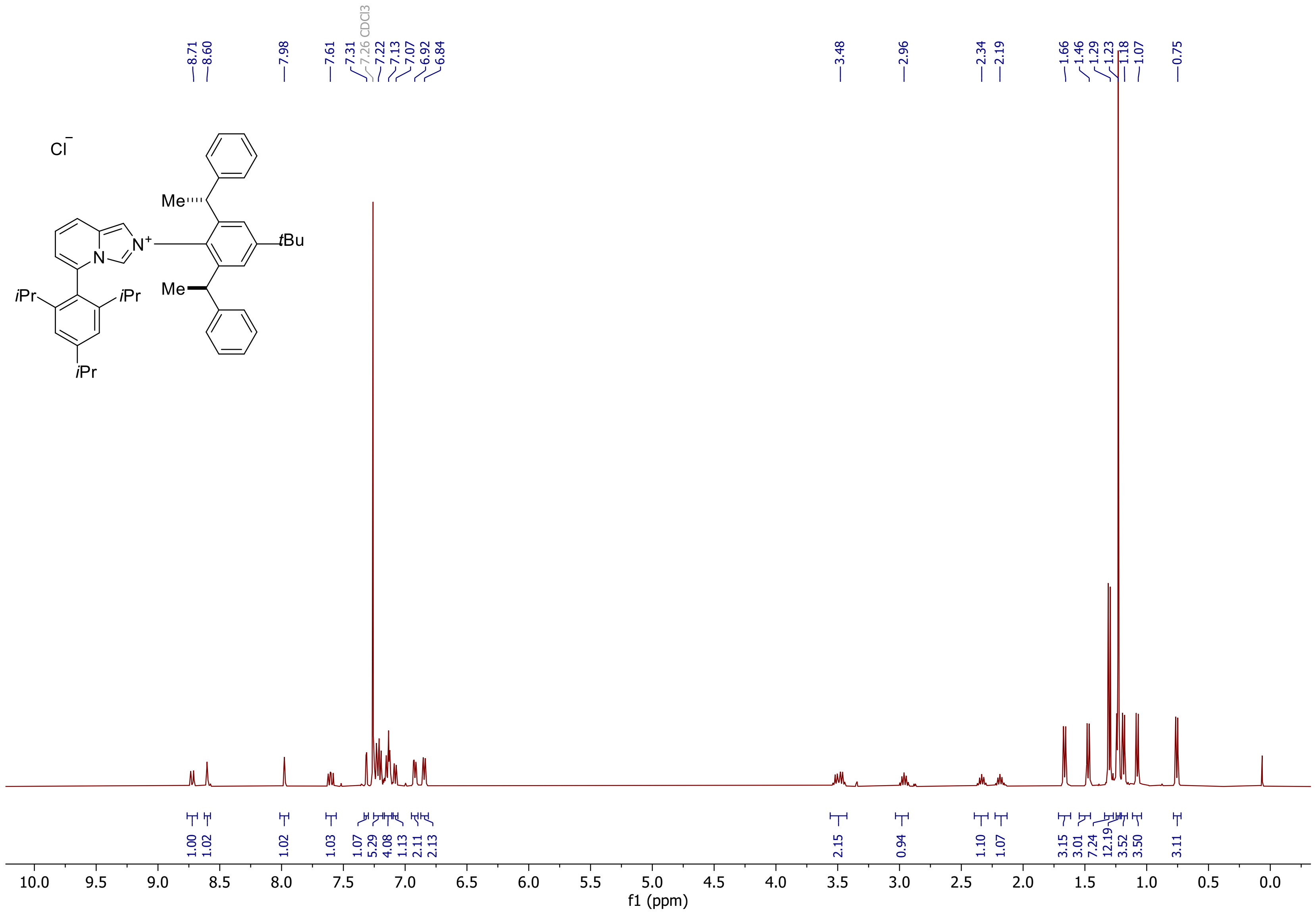

Figure S69. $^{13}C\{^{1}H\}$ NMR spectrum (101 MHz, 298 K, $CDCl_3$) of (*R*,*R*)-**5c**

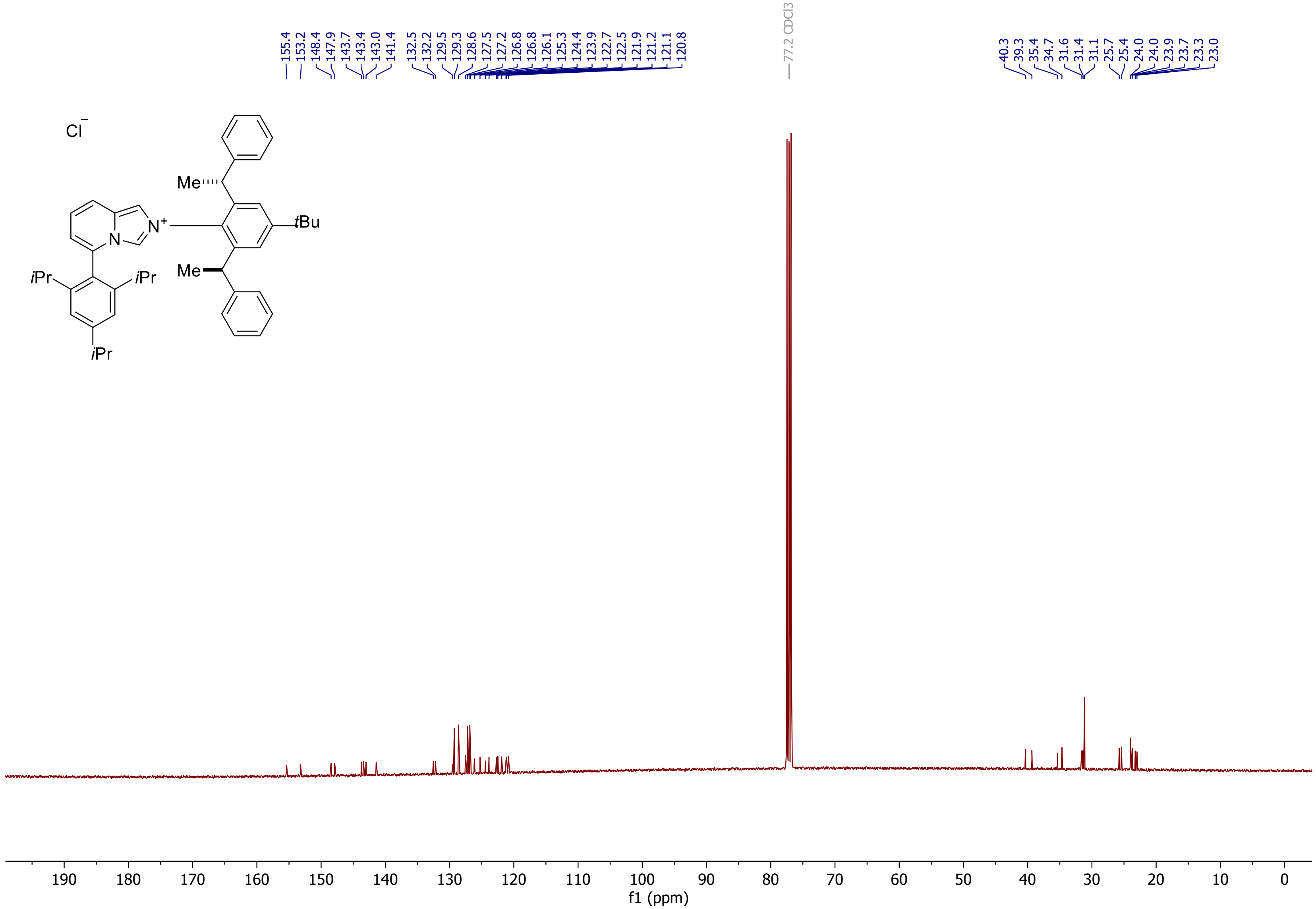

Figure S70. 2D ^{1}H-^{1}H COSY spectrum (298 K, $CDCl_3$) of (*R*,*R*)-**5c**

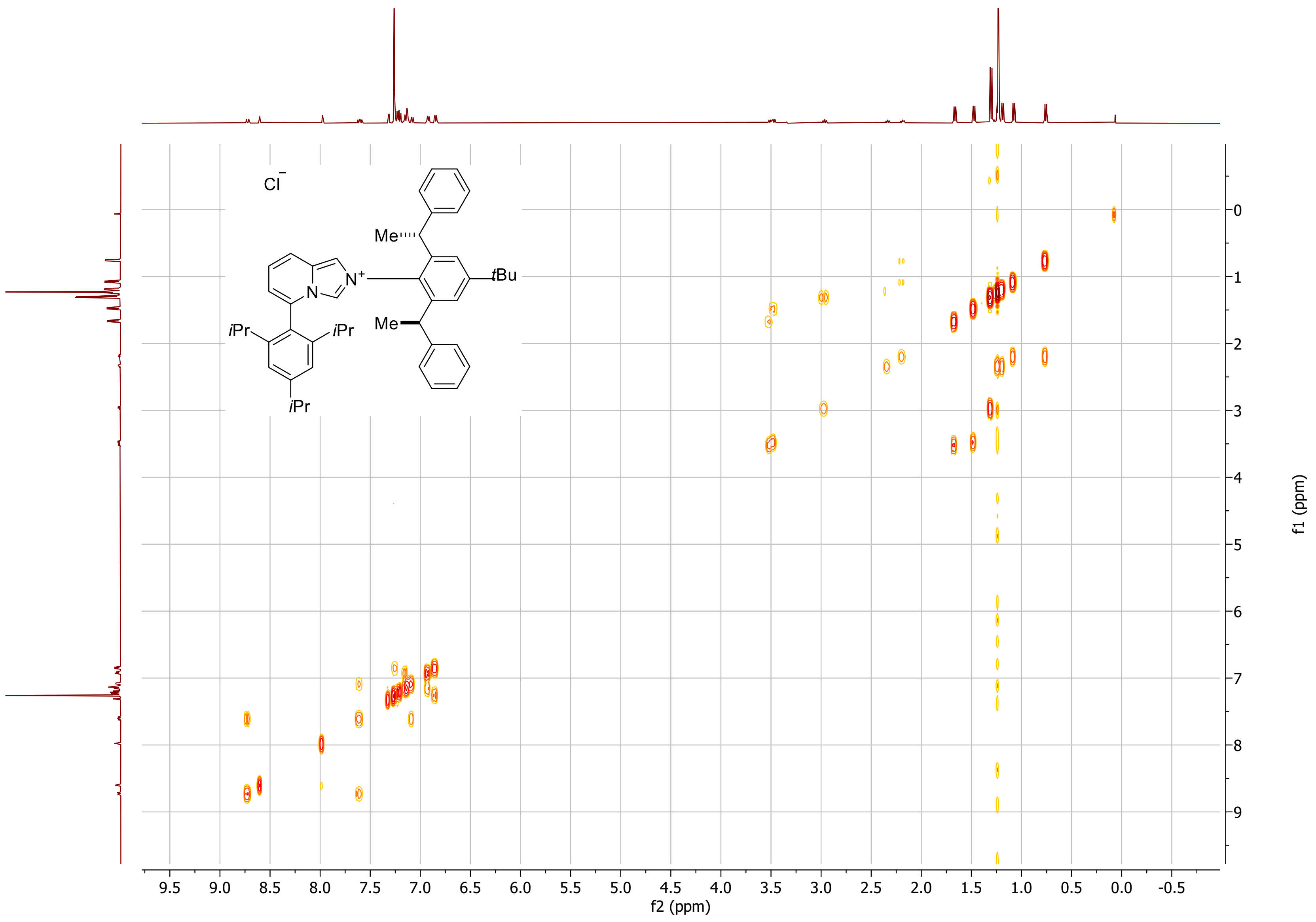

Figure S71. 2D ^{1}H-^{13}C HSQC spectrum (298 K, $CDCl_3$) of (*R*,*R*)-**5c**

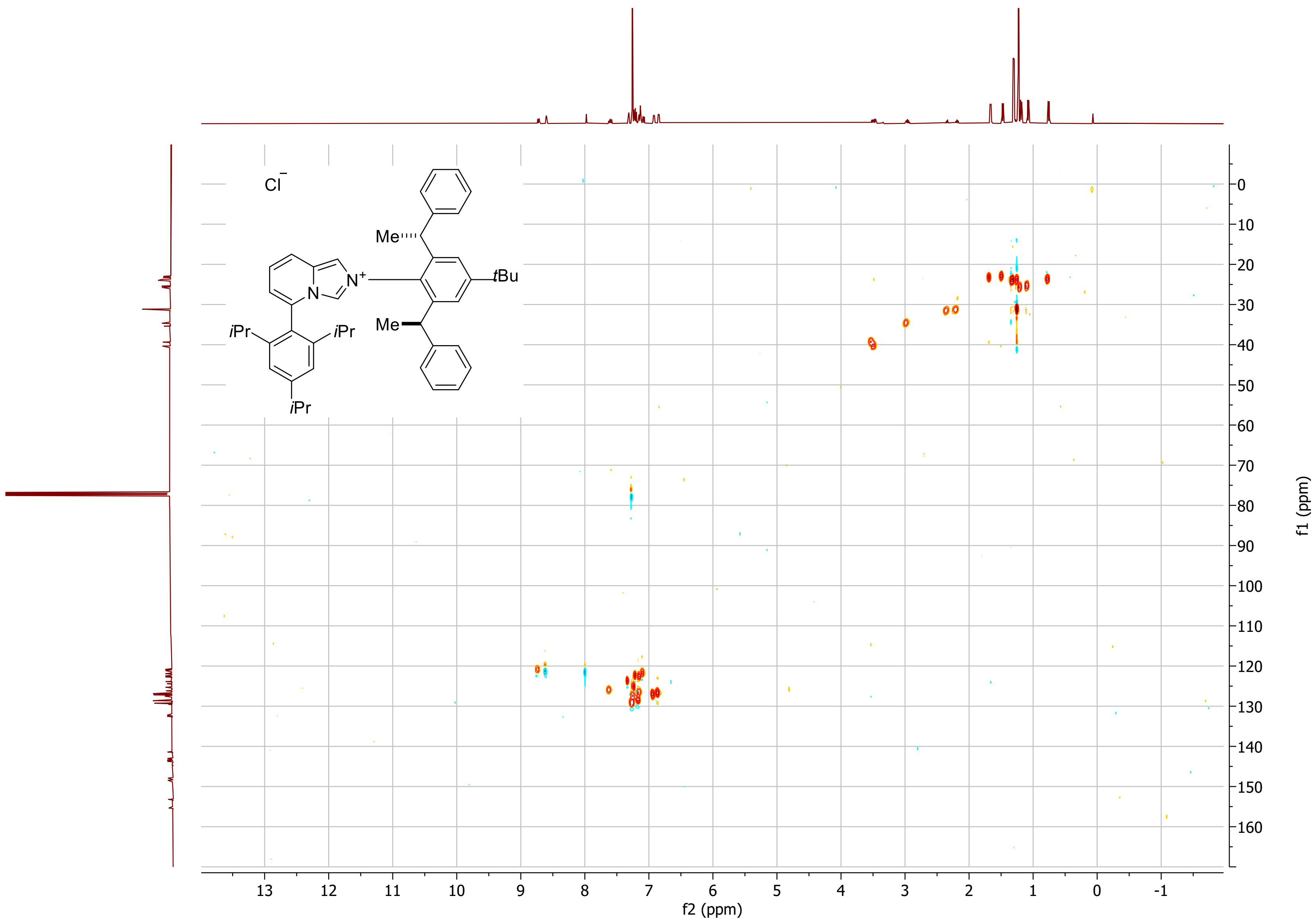

Figure S72. ^{1}H NMR spectrum (400 MHz, 298 K, $CDCl_3$) of (*R*,*R*)-**5d**

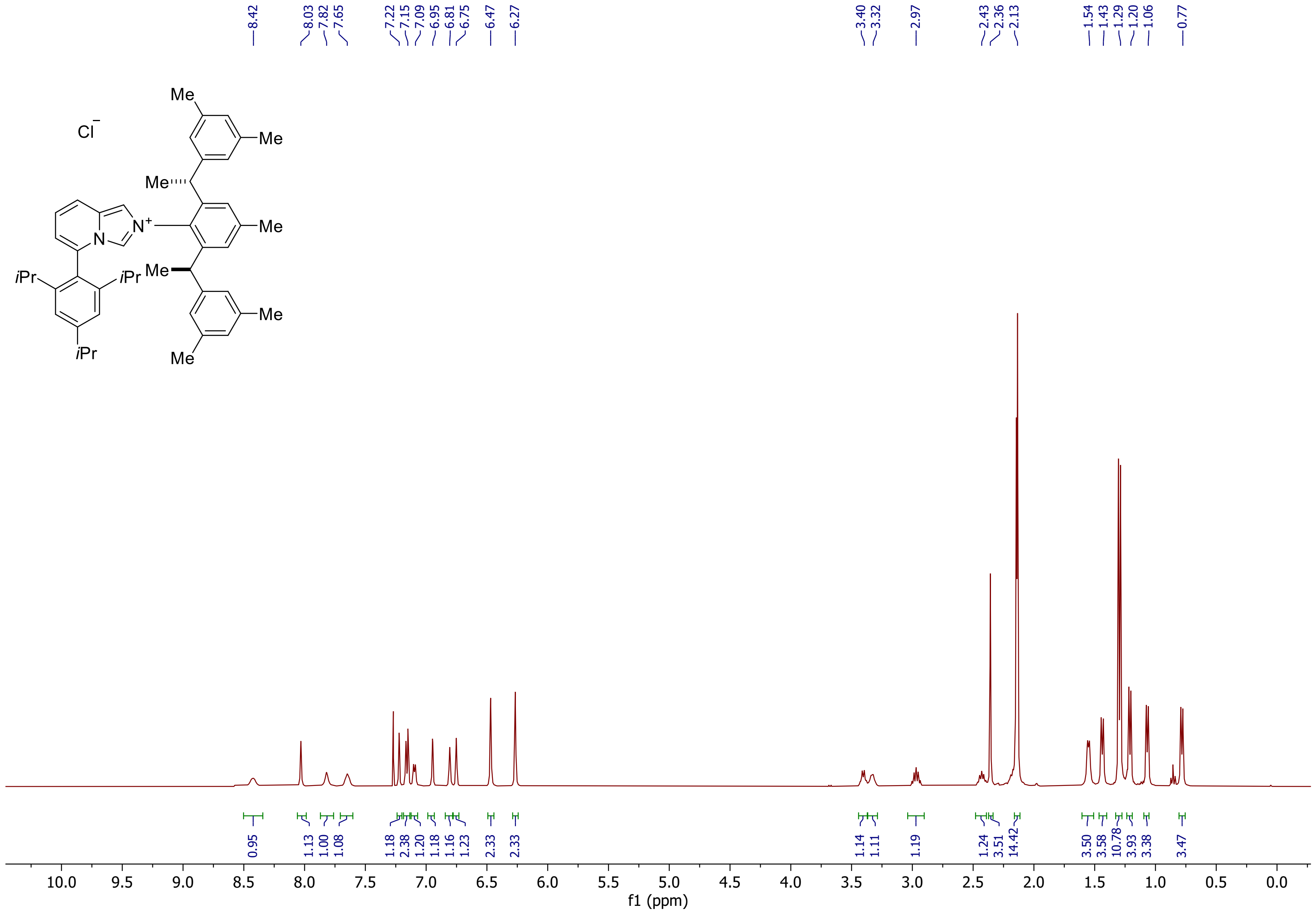

Figure S73. $^{13}C\{^{1}H\}$ NMR spectrum (101 MHz, 298 K, $CDCl_3$) of (*R*,*R*)-**5d**

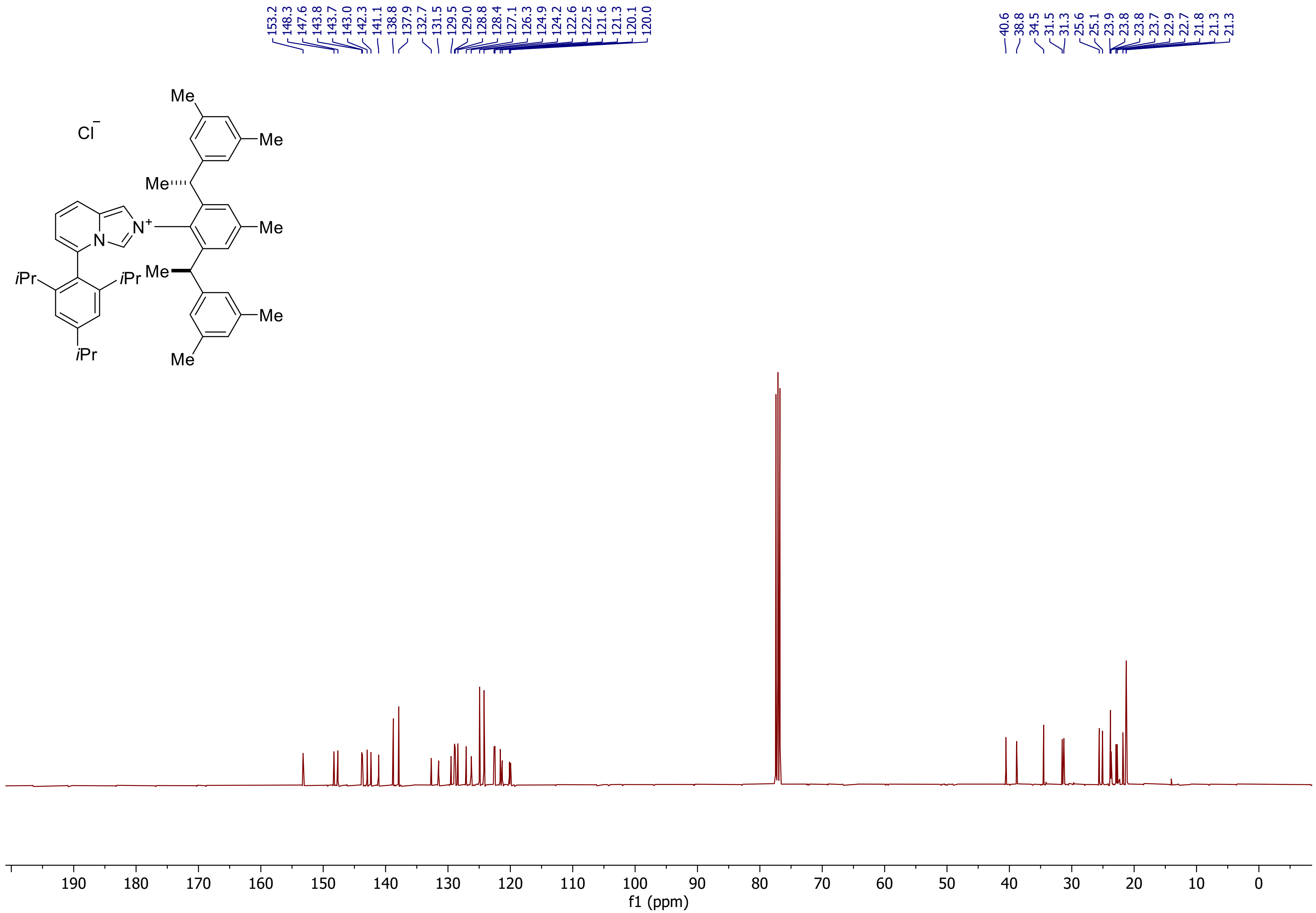

Figure S74. 2D ^{1}H-^{1}H COSY spectrum (298 K, $CDCl_3$) of (*R*,*R*)-**5d**

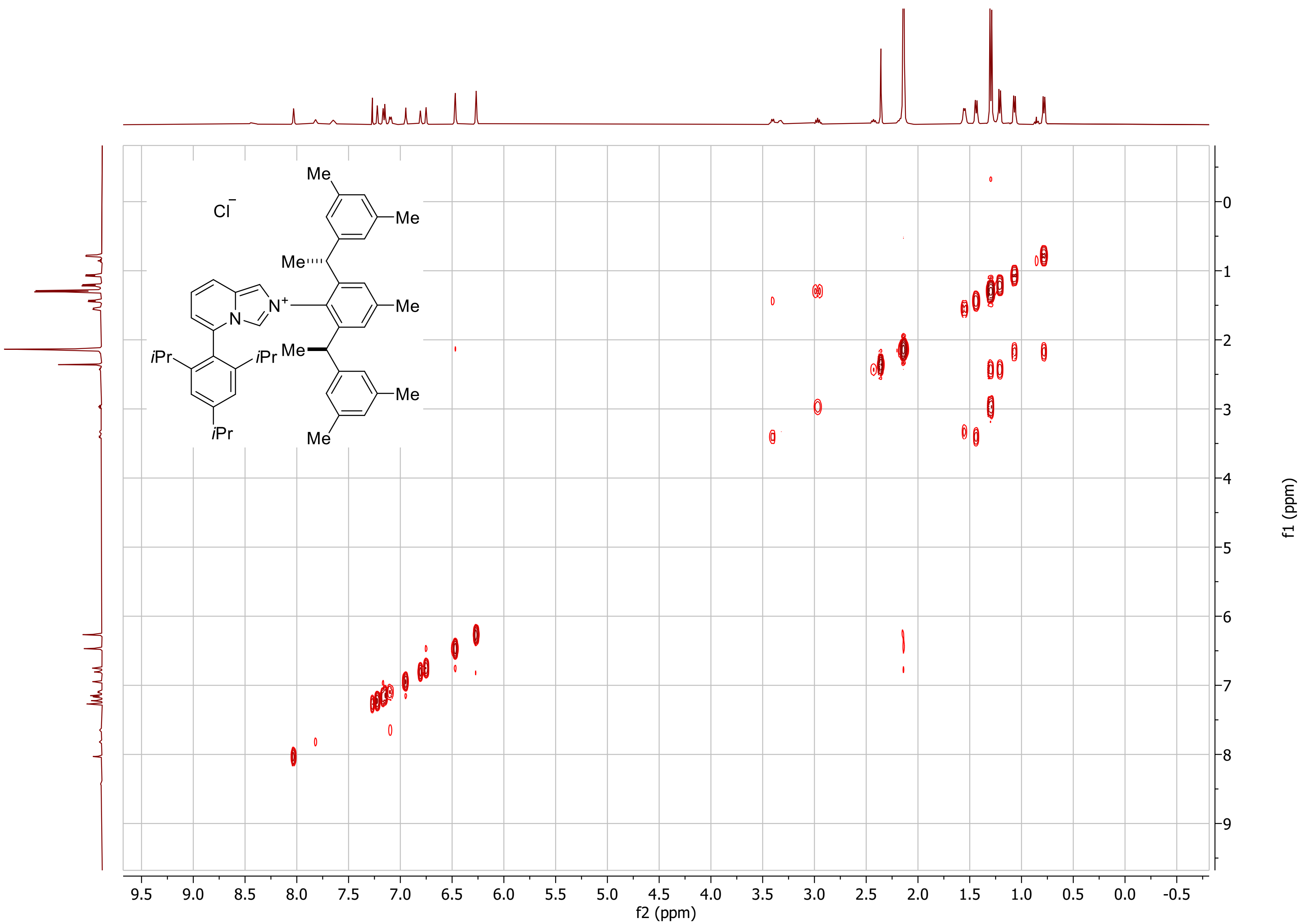

Figure S75. 2D ^1H-^{13}C HSQC spectrum (298 K, $CDCl_3$) of (*R*,*R*)-**5d**

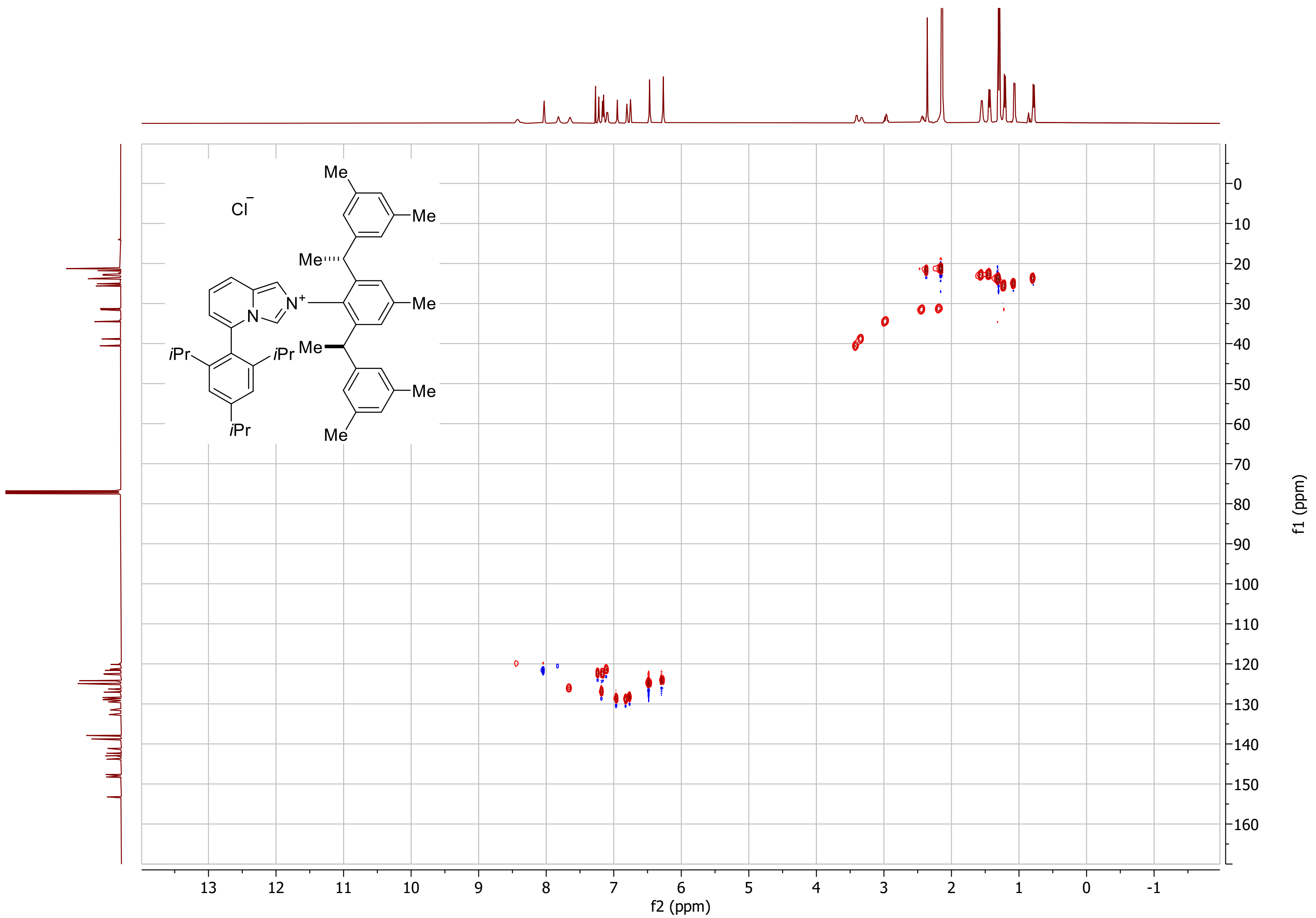

Figure S76. ^{1}H NMR spectrum (400 MHz, 298 K, $CDCl_3$) of (*R*,*R*)-**5e**

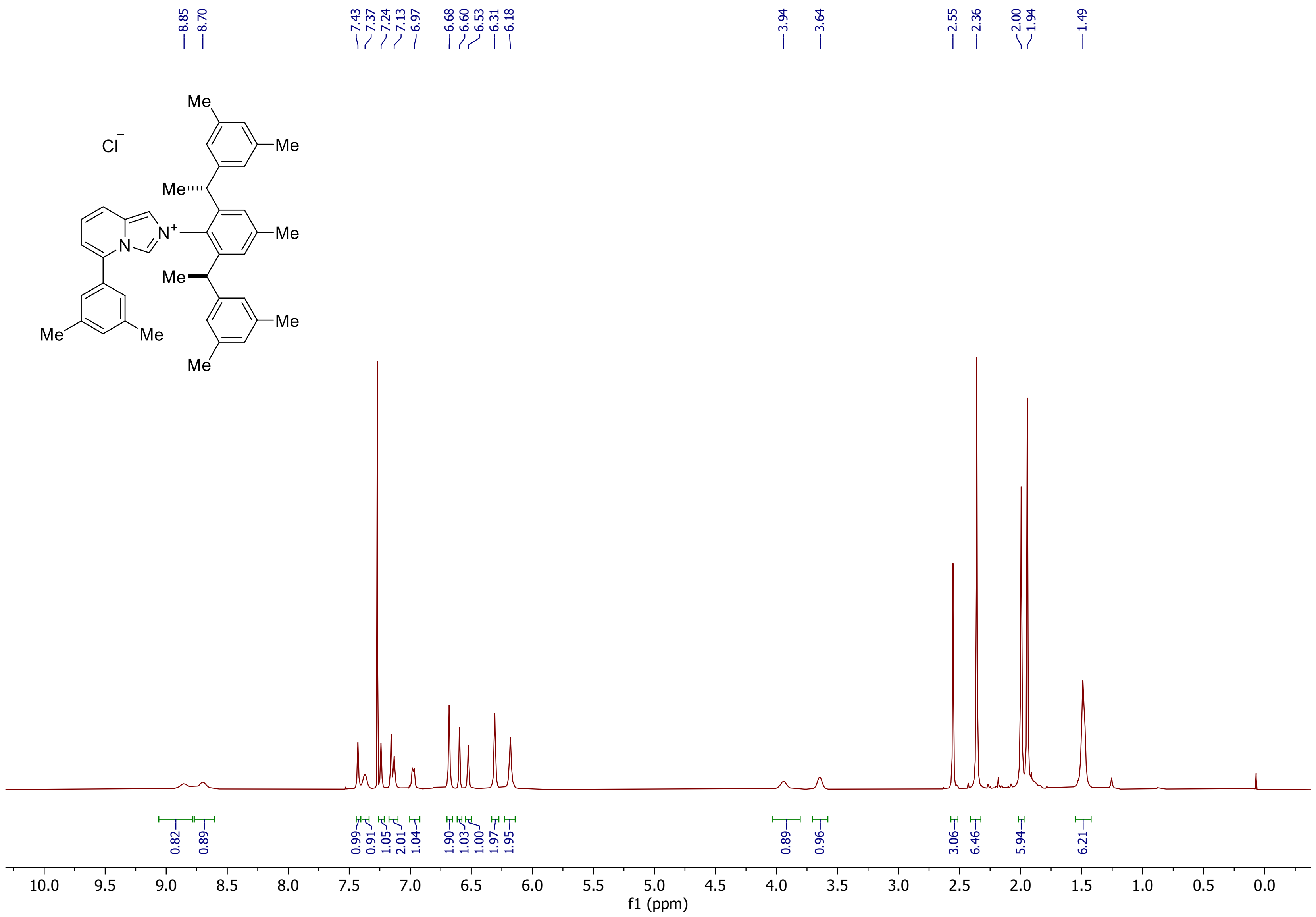

Figure S77. $^{13}C\{^{1}H\}$ NMR spectrum (101 MHz, 298 K, $CDCl_3$) of (*R*,*R*)-**5e**

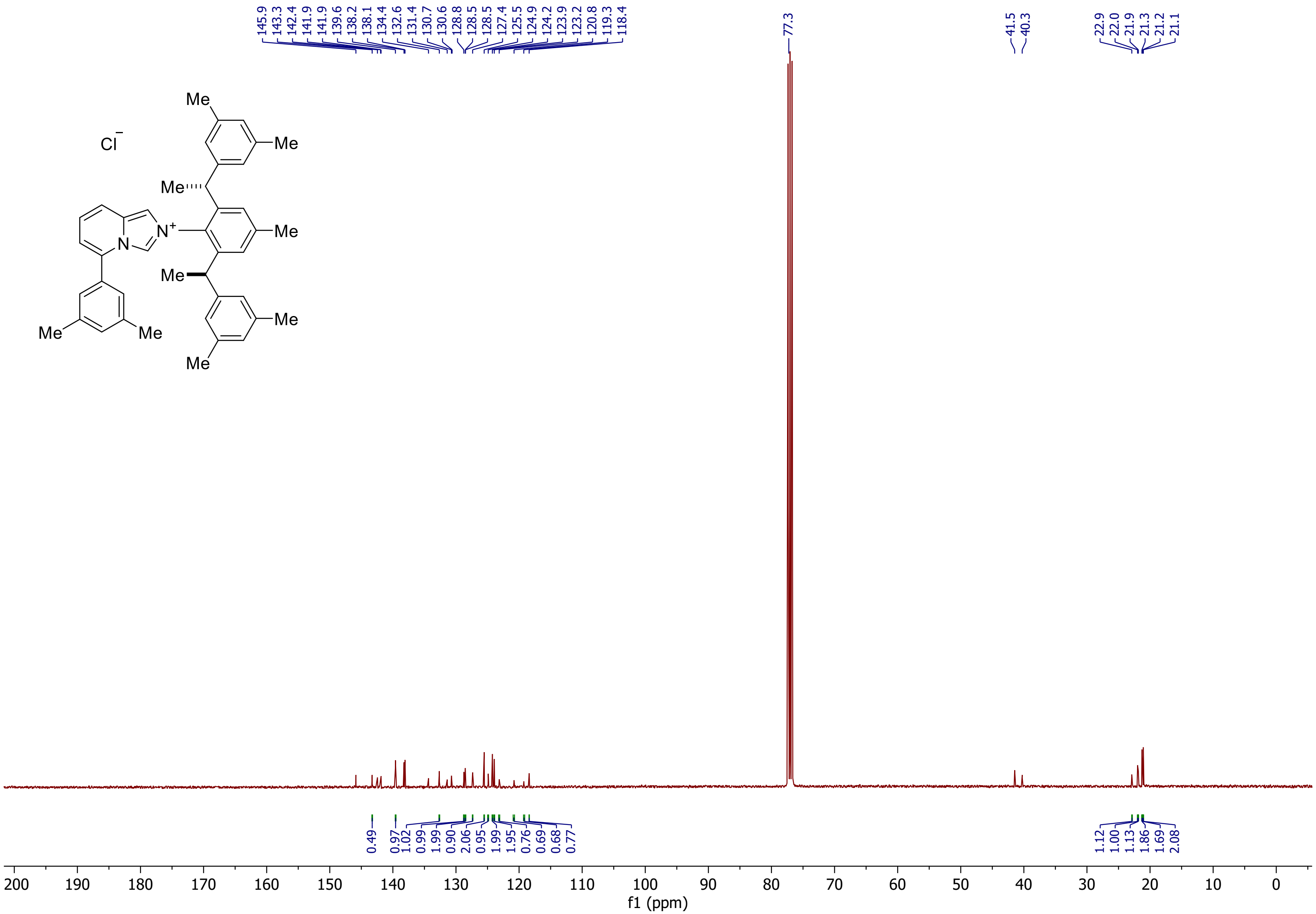

Figure S78. 2D ^{1}H-^{1}H COSY spectrum (298 K, $CDCl_3$) of (*R*,*R*)-**5e**

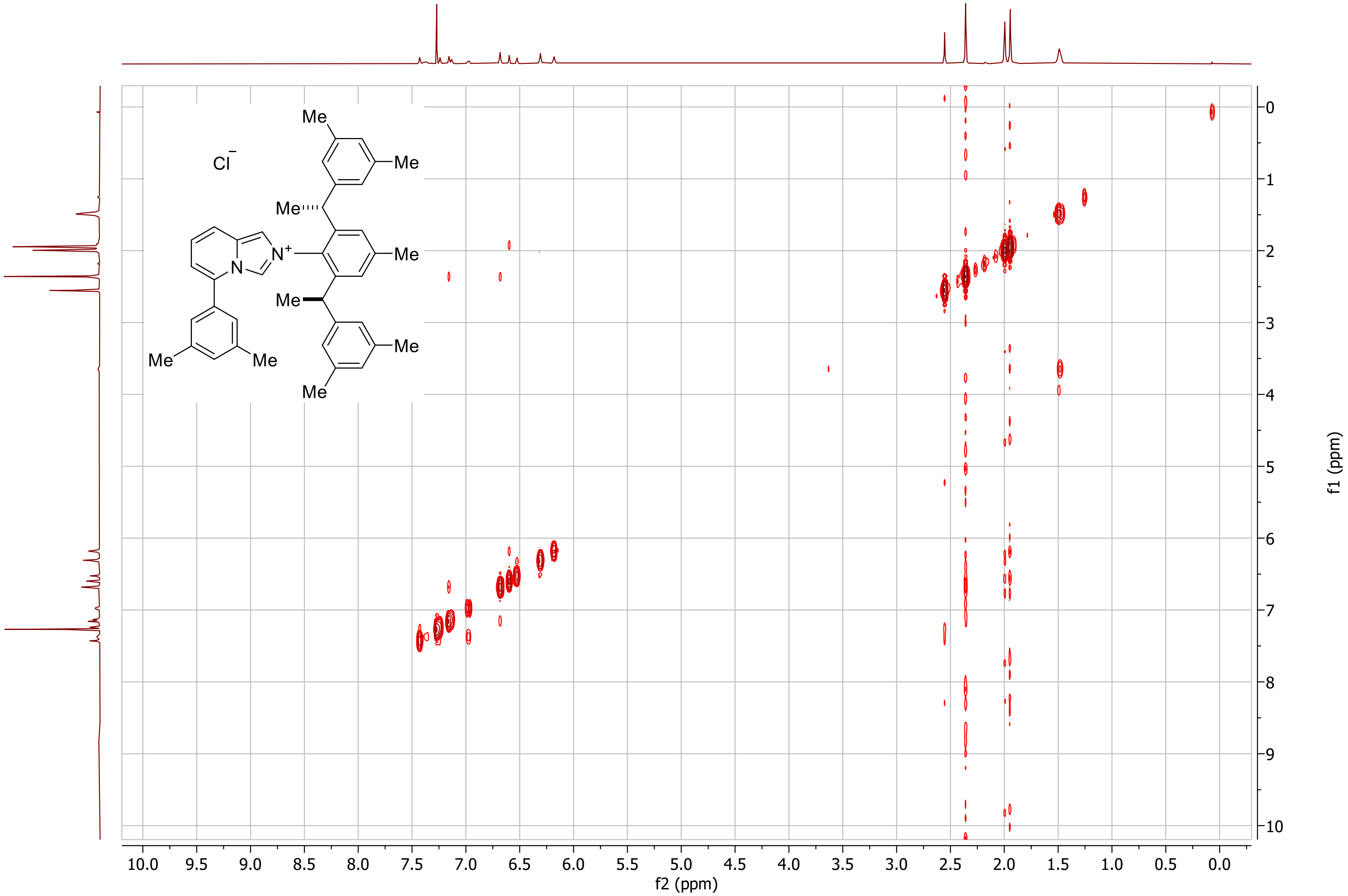

Figure S79. 2D ^1H-^{13}C HSQC spectrum (298 K, $CDCl_3$) of (*R*,*R*)-**5e**

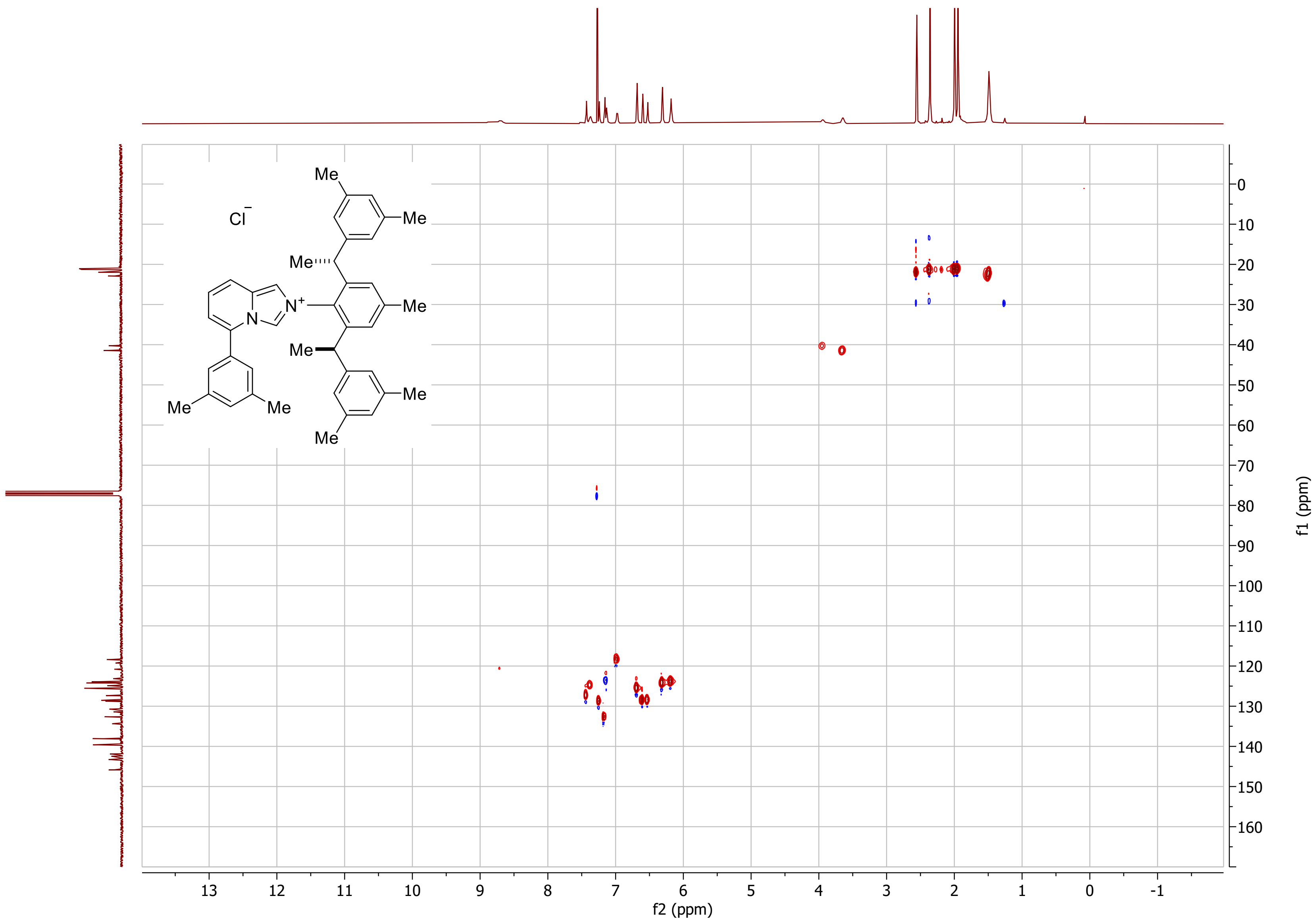

Figure S80. ^{1}H NMR spectrum (400 MHz, 298 K, $CDCl_3$) of (*R*,*R*)-**5f**

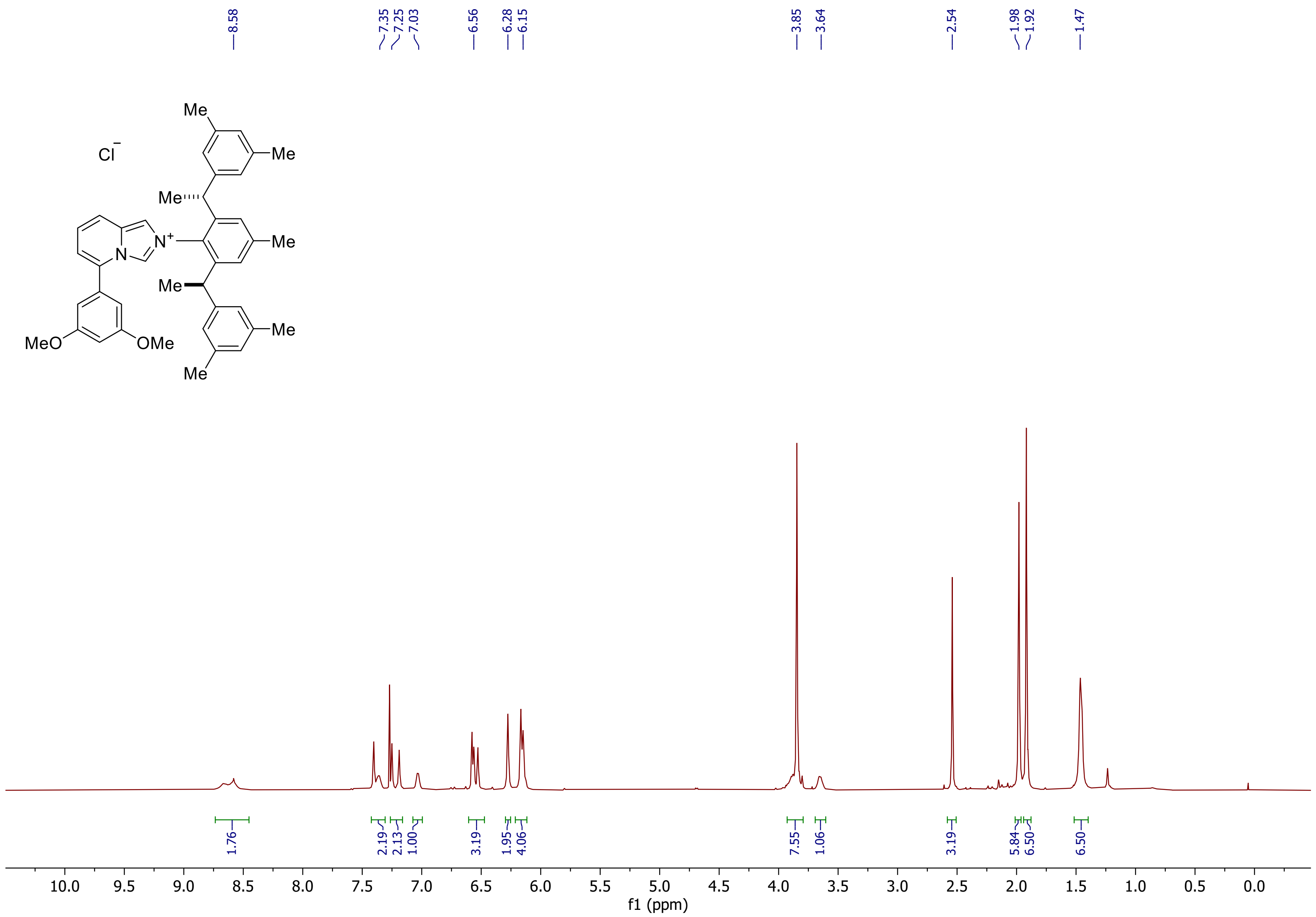

Figure S81. $^{13}C\{^{1}H\}$ NMR spectrum (101 MHz, 298 K, $CDCl_3$) of (*R*,*R*)-**5f**

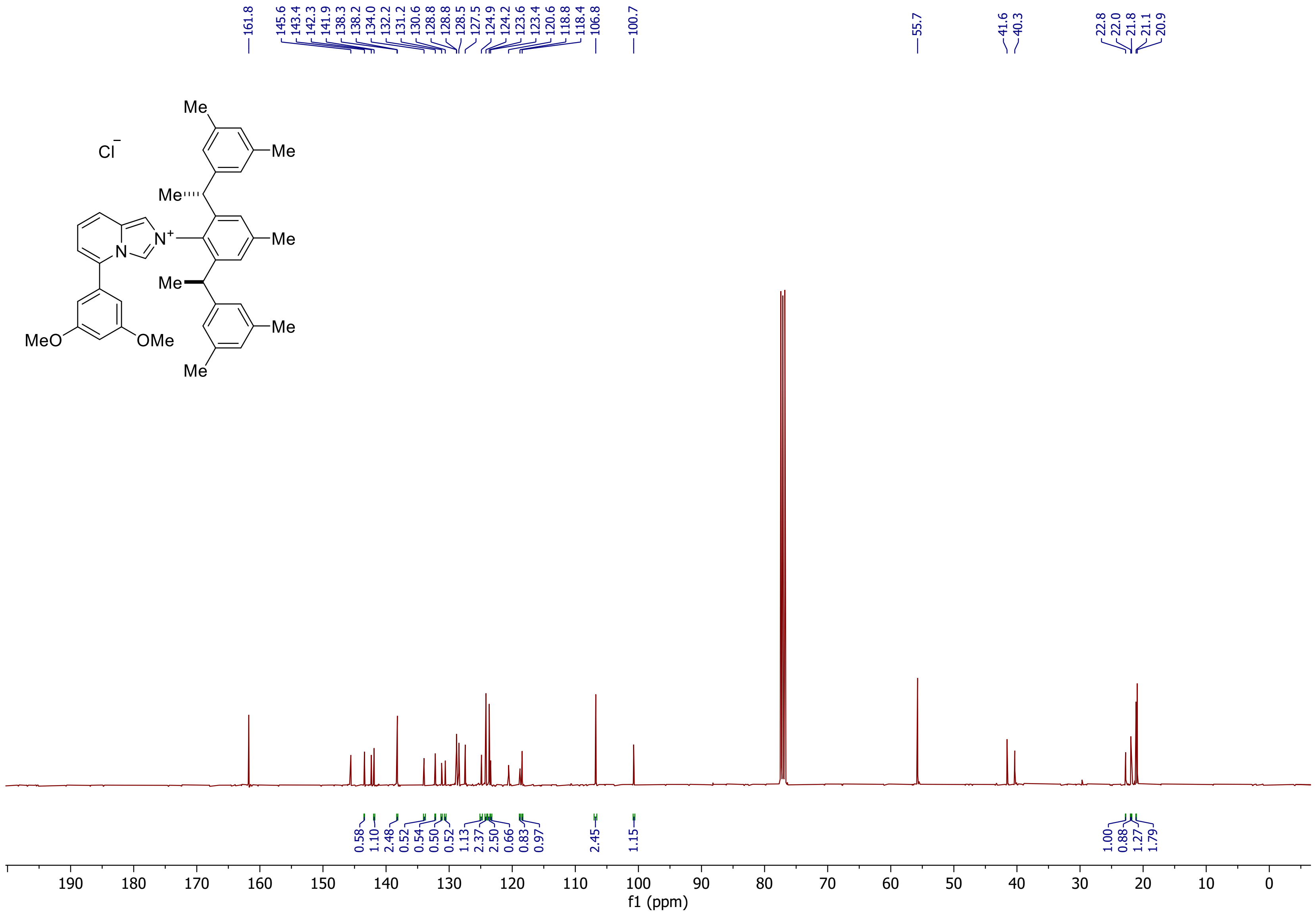

Figure S82. 2D ^{1}H-^{1}H COSY spectrum (298 K, $CDCl_3$) of (*R*,*R*)-**5f**

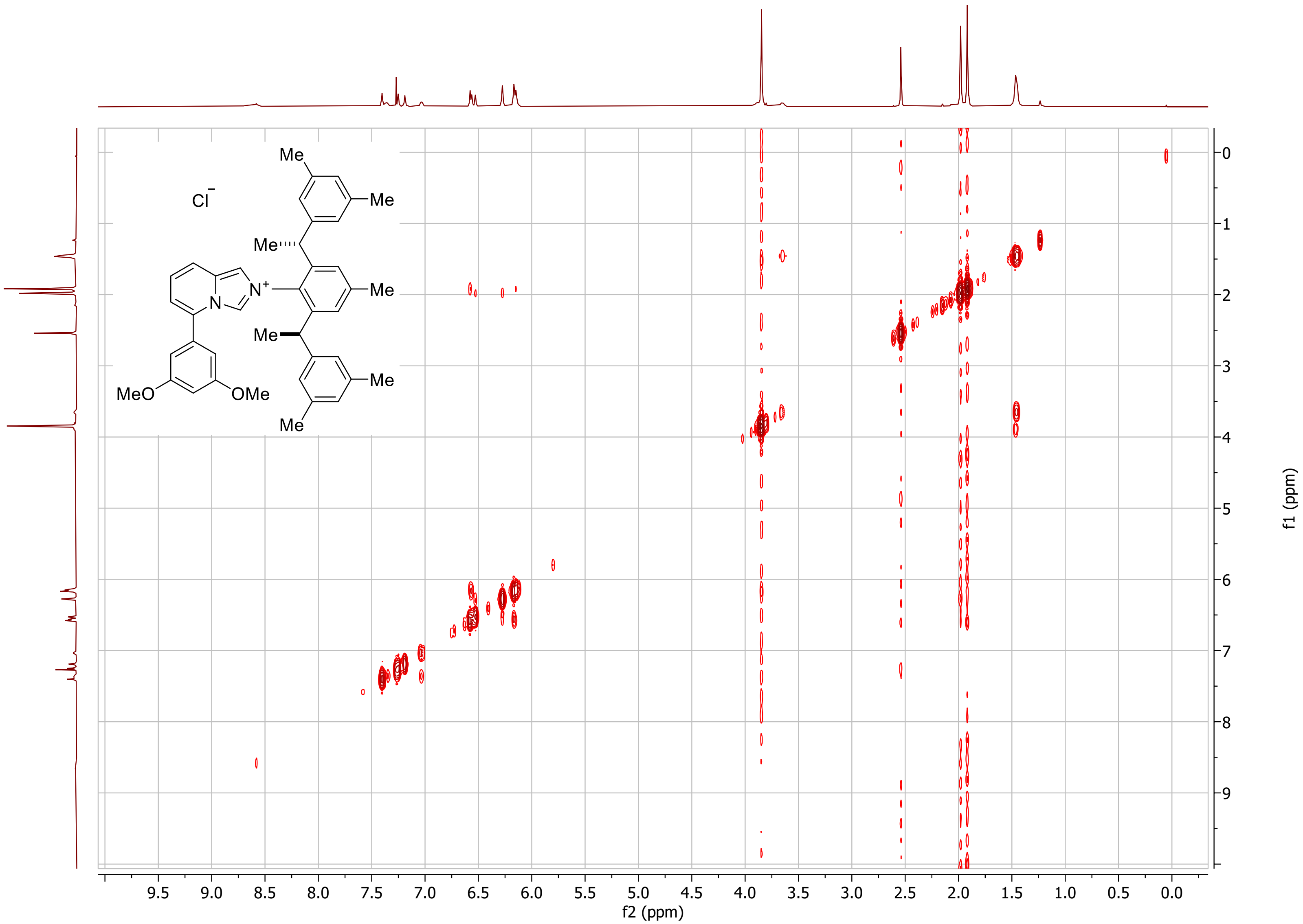

Figure S83. 2D ^{1}H-^{13}C HSQC spectrum (298 K, $CDCl_3$) of (*R*,*R*)-**5f**

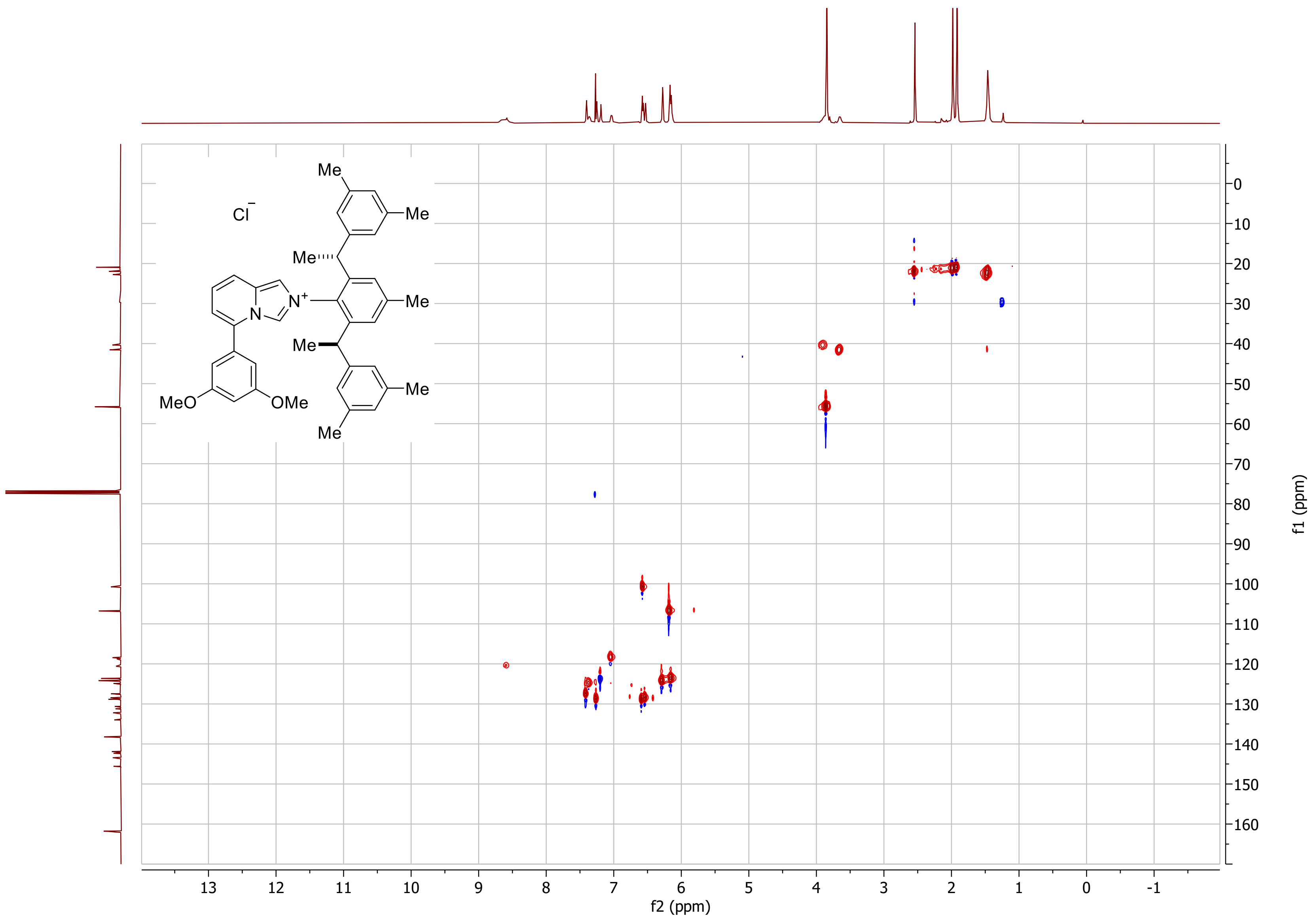

Figure S84. ^{1}H NMR spectrum (400 MHz, 298 K, $CDCl_3$) of (*R*,*R*)-**5h**

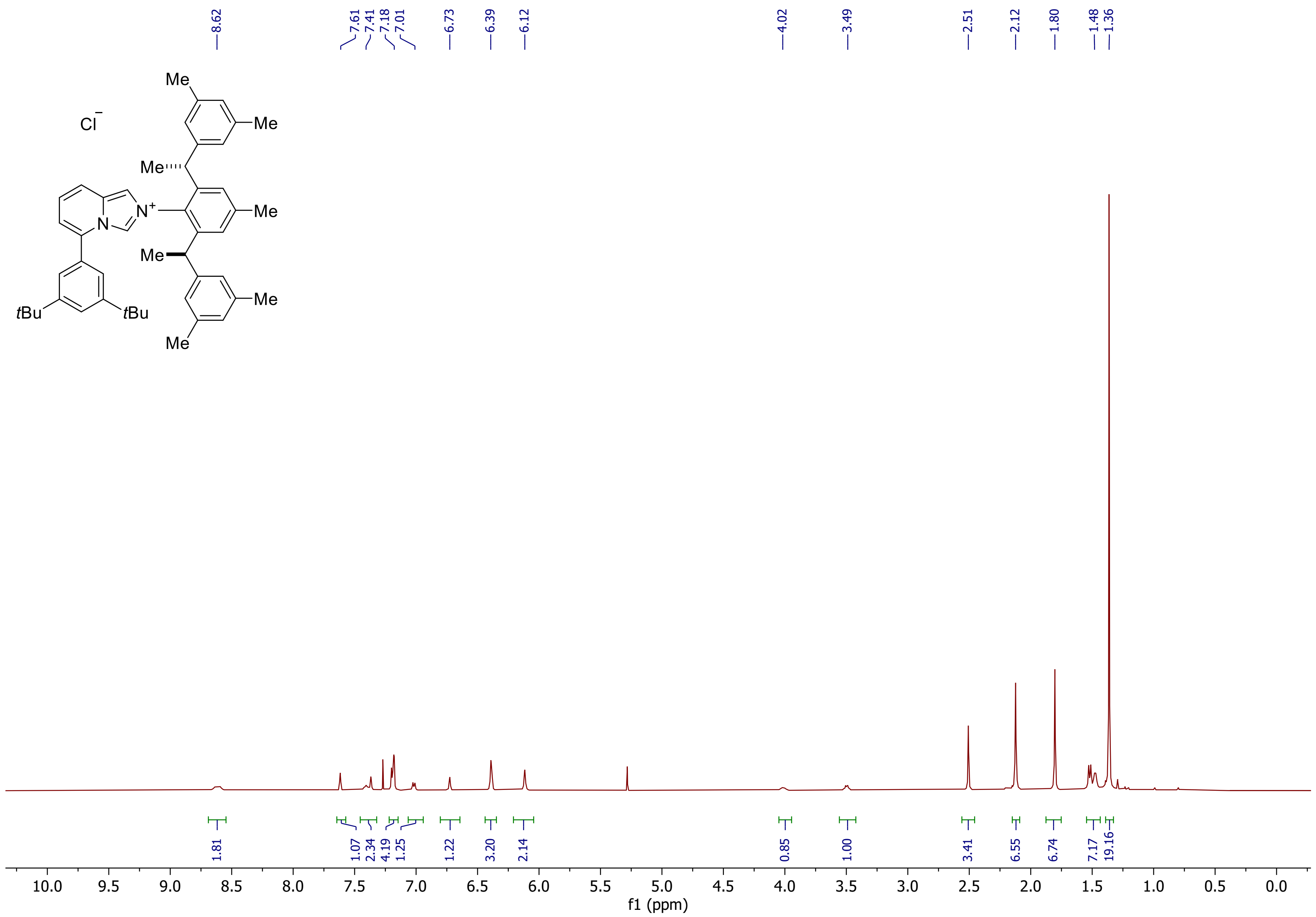

Figure S85. $^{13}C\{^{1}H\}$ NMR spectrum (101 MHz, 298 K, $CDCl_3$) of (*R*,*R*)-**5h**

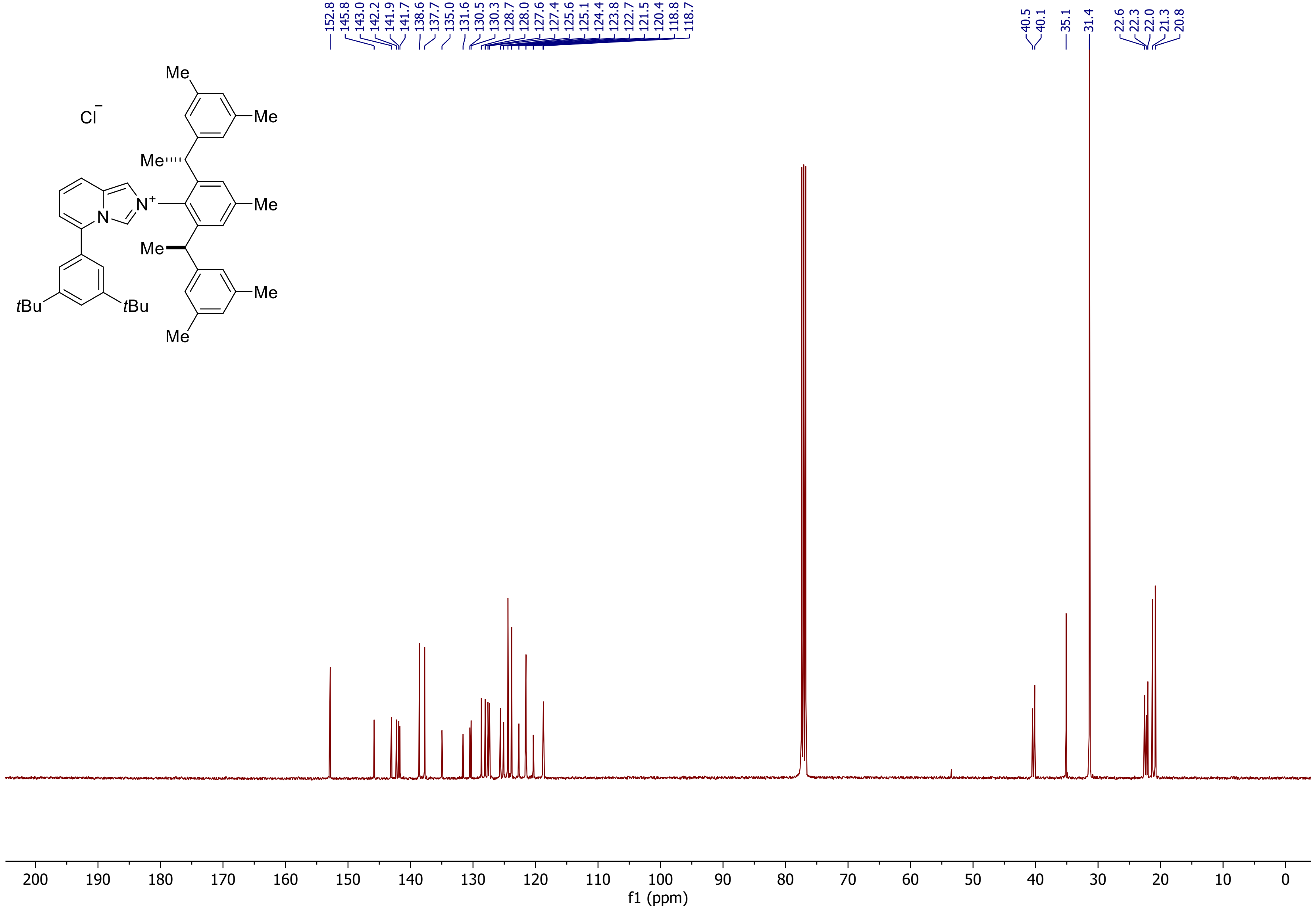

Figure S86. 2D ^{1}H-^{1}H COSY spectrum (298 K, $CDCl_3$) of (*R*,*R*)-**5h**

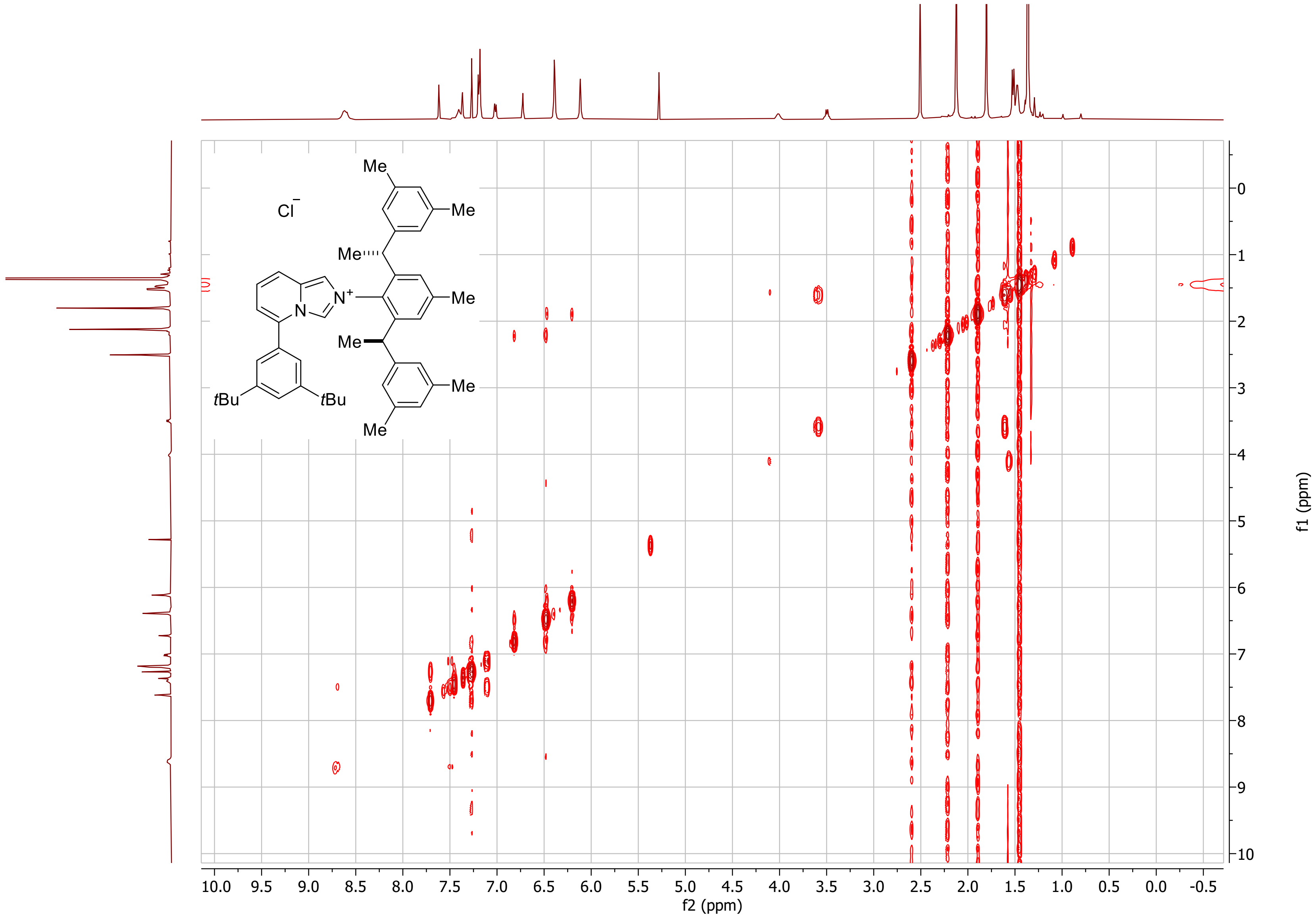

Figure S87. 2D ^{1}H-^{13}C HSQC spectrum (298 K, $CDCl_3$) of (*R*,*R*)-**5h**

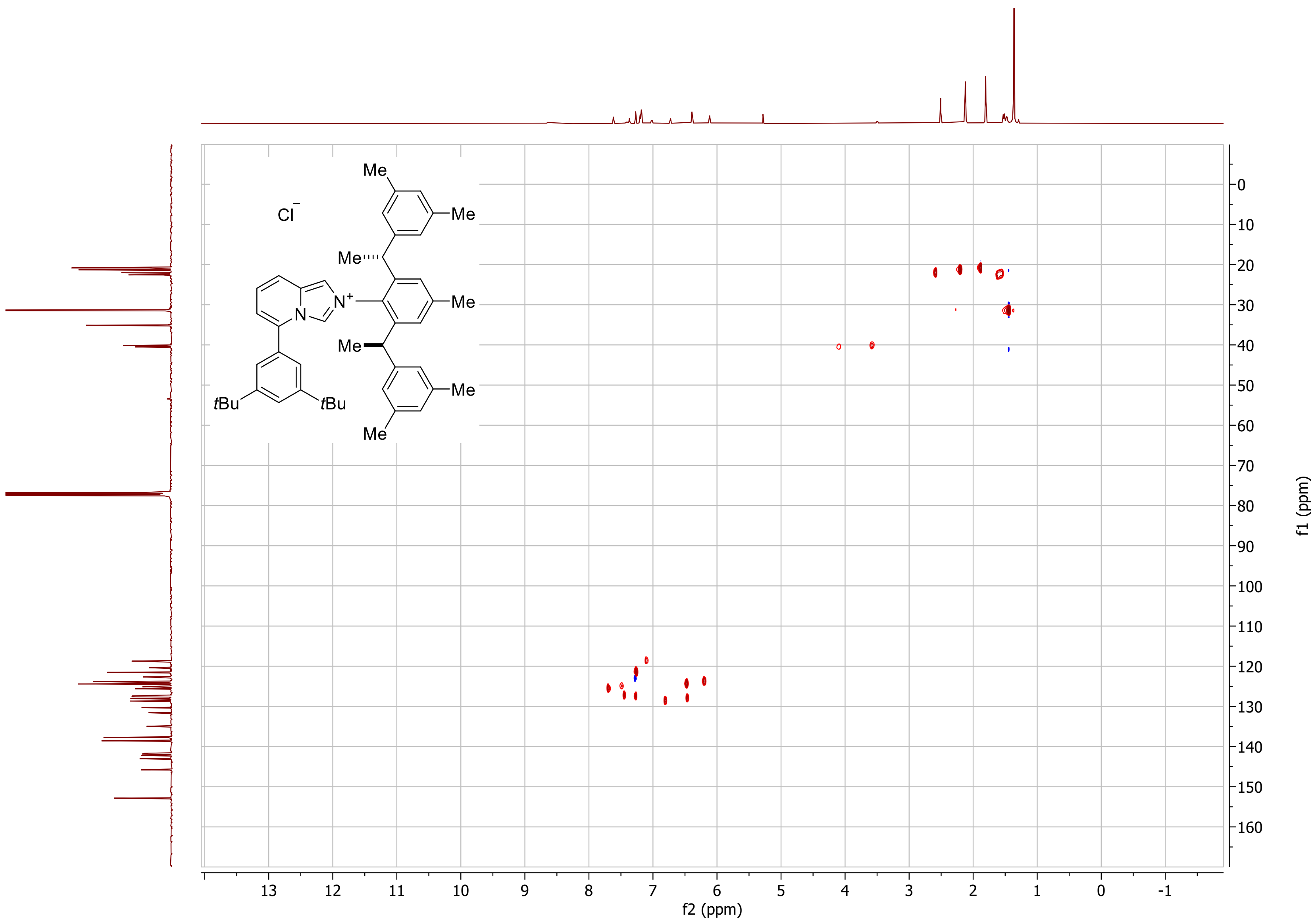

Figure S88. ^{1}H NMR spectrum (400 MHz, 298 K, $CDCl_3$) of (*R*,*R*)-**5i**

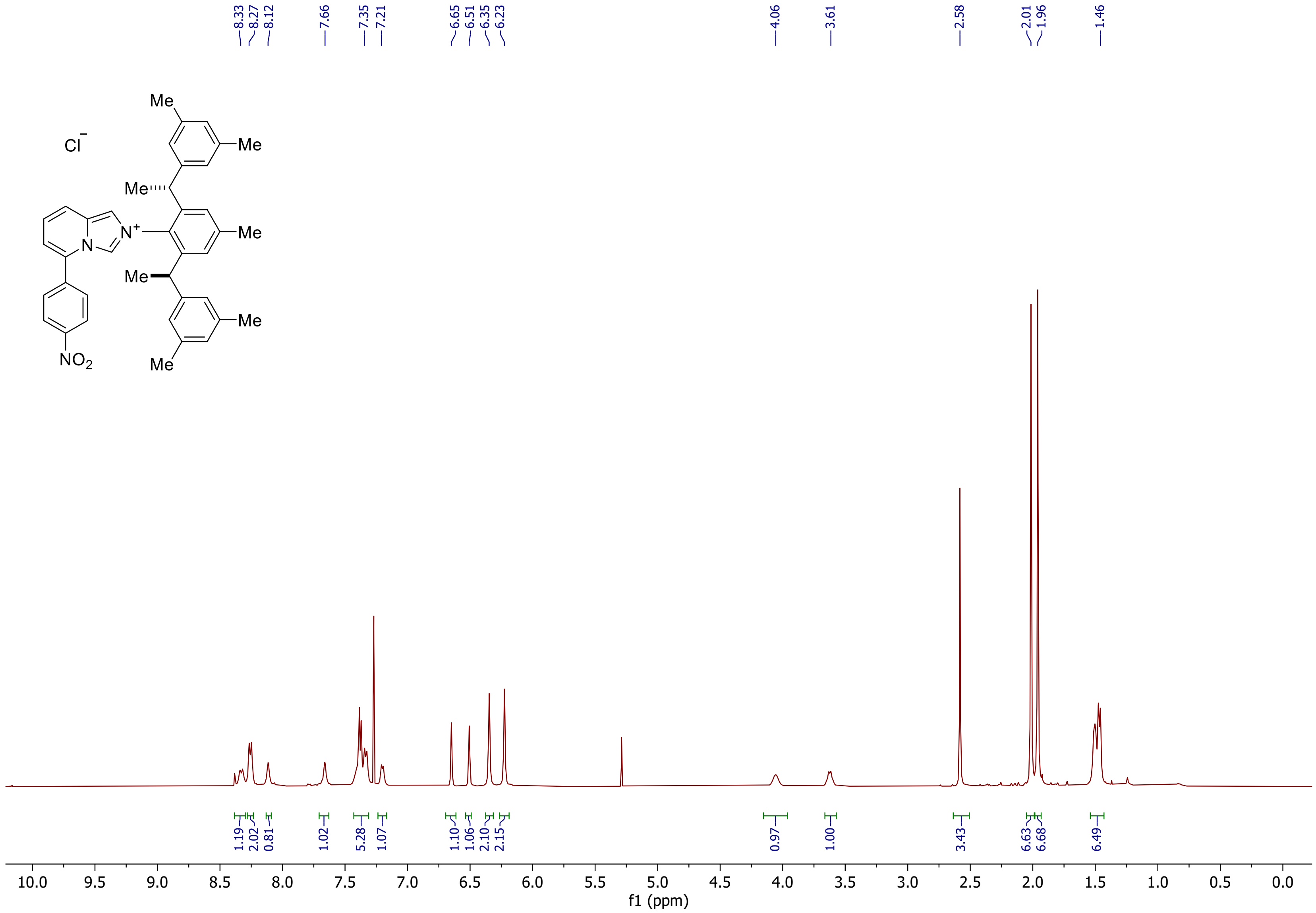

Figure S89. $^{13}C\{^1H\}$ NMR spectrum (101 MHz, 298 K, $CDCl_3$) of (*R*,*R*)-**5i**

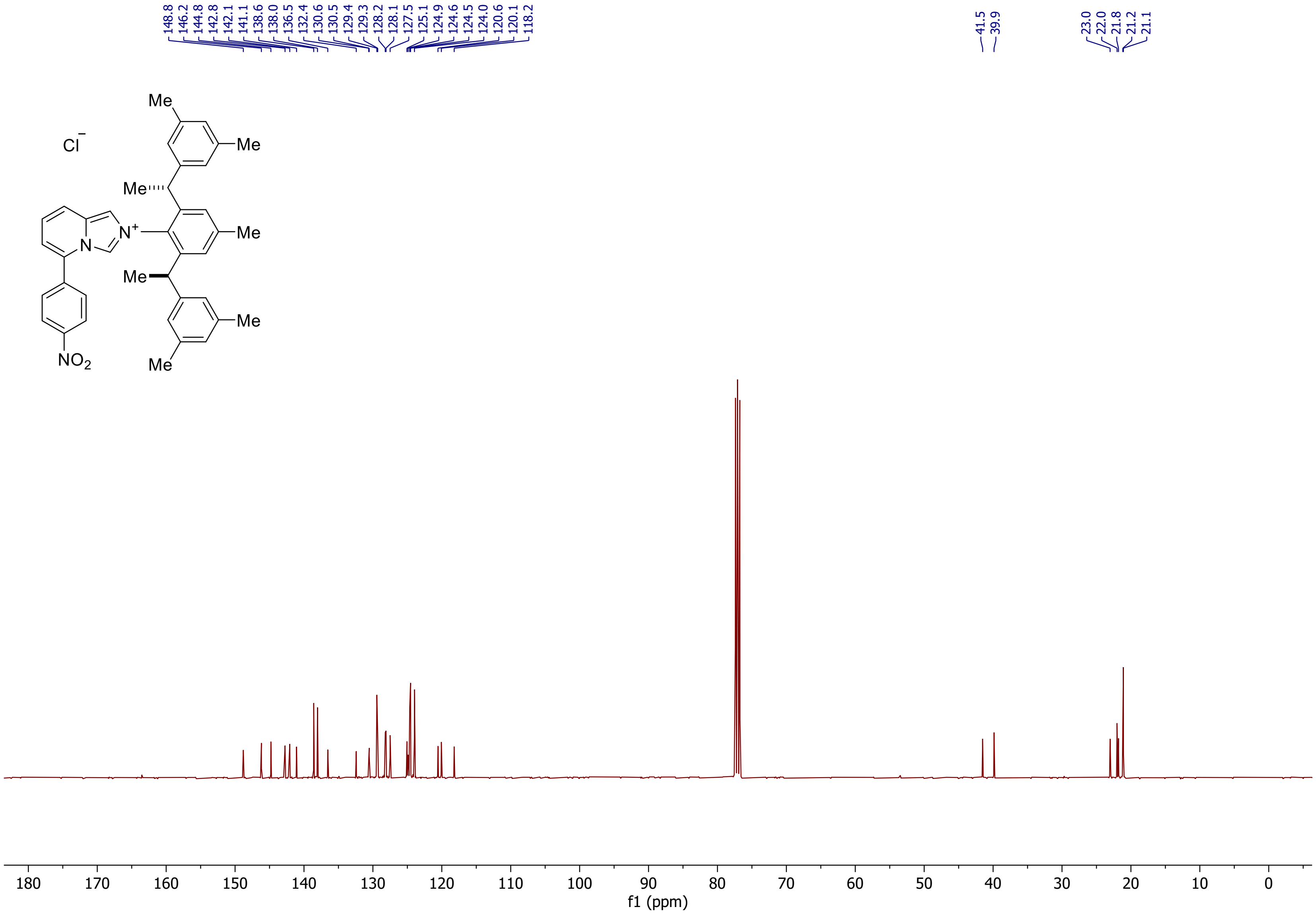

Figure S90. 2D ^{1}H-^{1}H COSY spectrum (298 K, $CDCl_3$) of (*R*,*R*)-**5i**

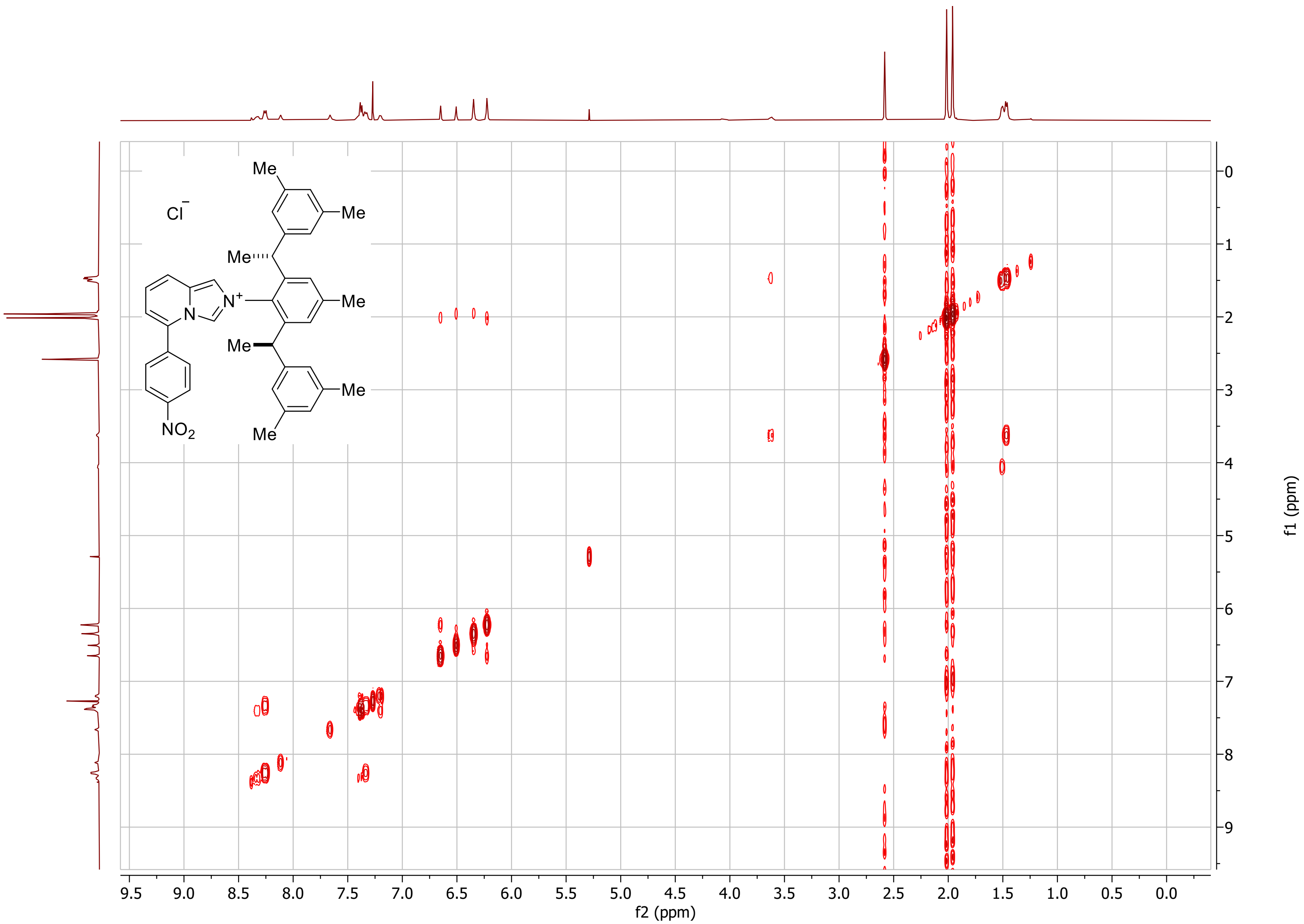

Figure S91. 2D ^{1}H-^{13}C HSQC spectrum (298 K, $CDCl_3$) of (*R*,*R*)-**5i**

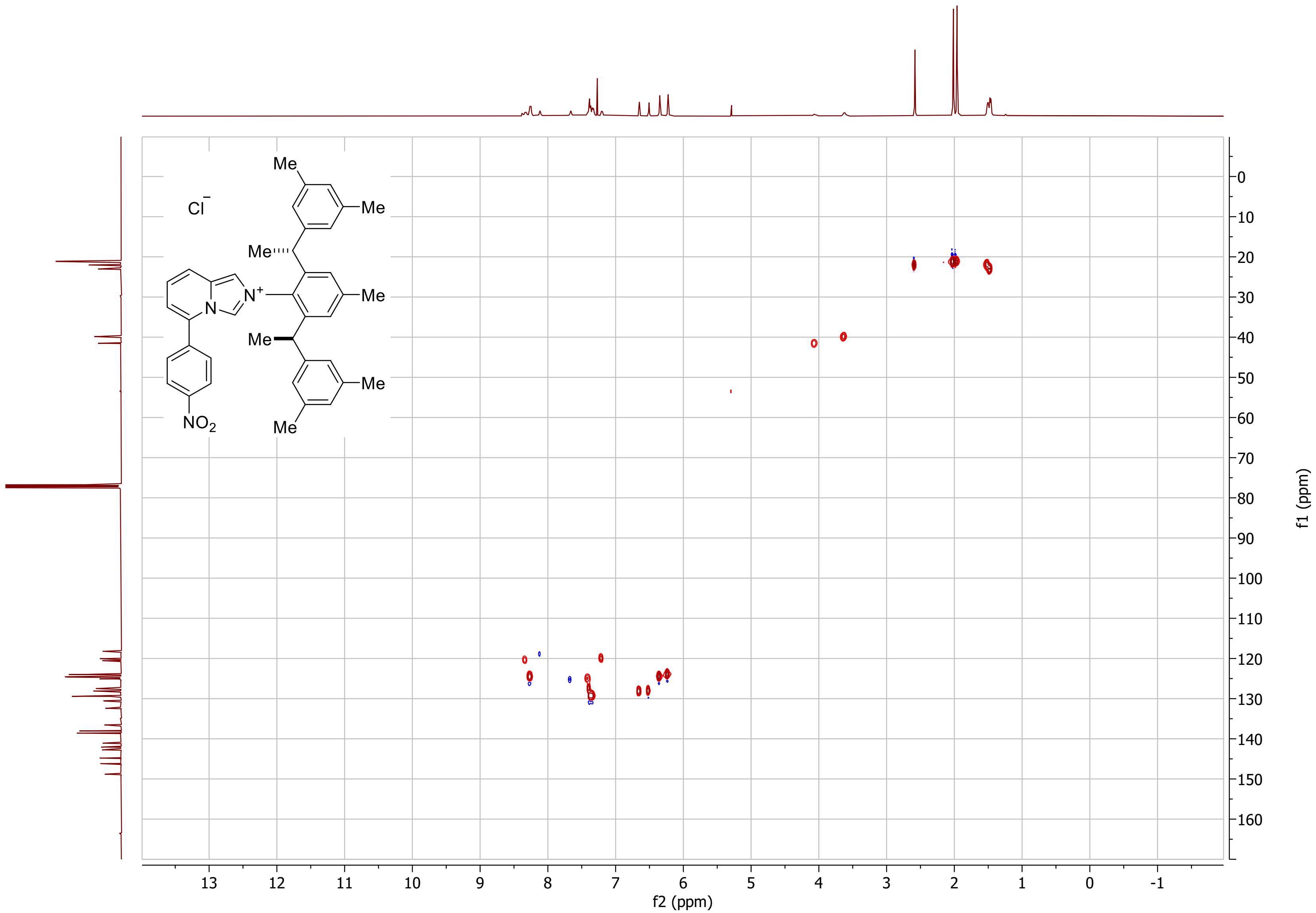

Figure S92. ^{1}H NMR spectrum (400 MHz, 298 K, $CDCl_3$) of (*R*,*R*)-**5j**

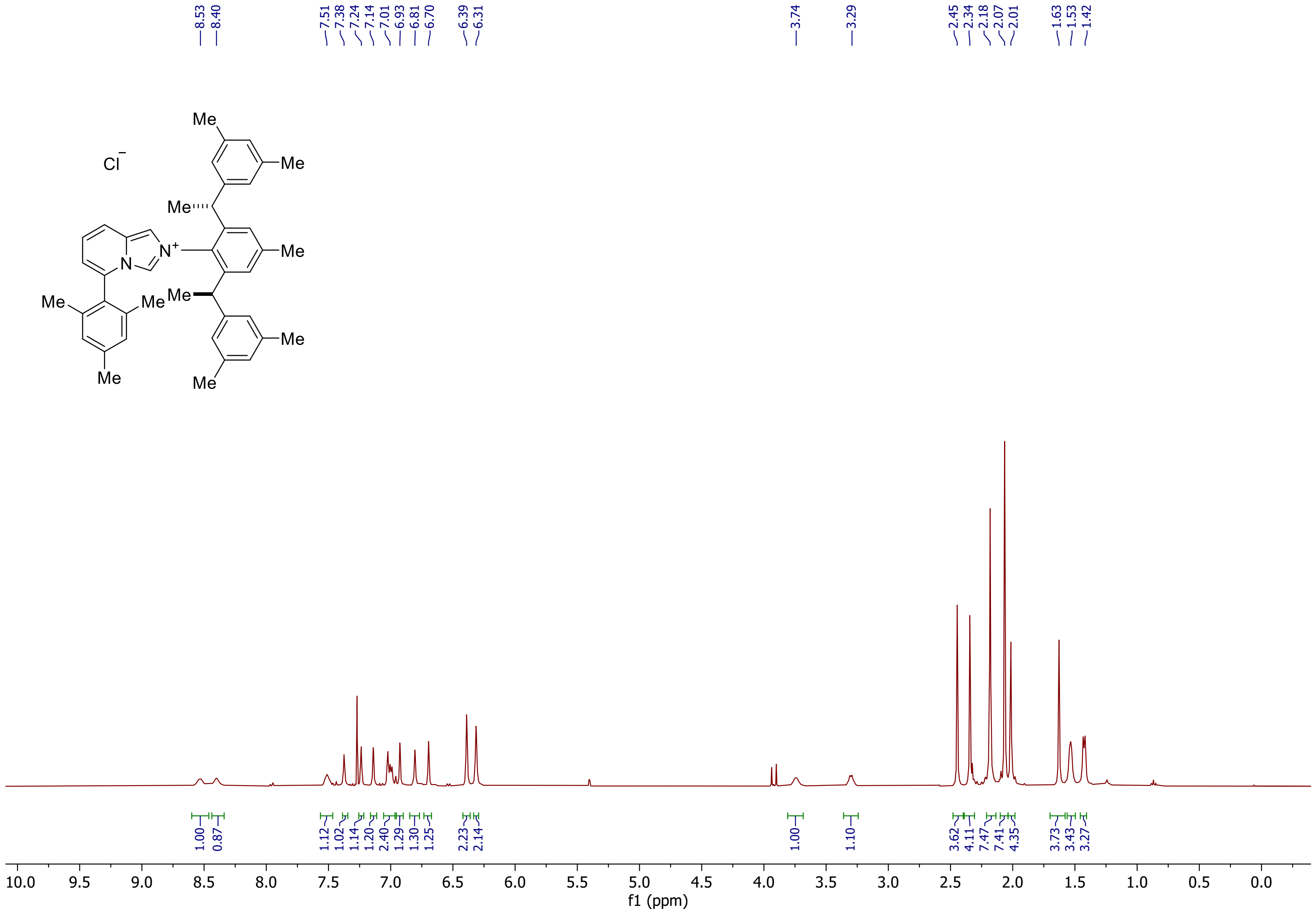

Figure S93. $^{13}C\{^1H\}$ NMR spectrum (101 MHz, 298 K, $CDCl_3$) of (*R*,*R*)-**5j**

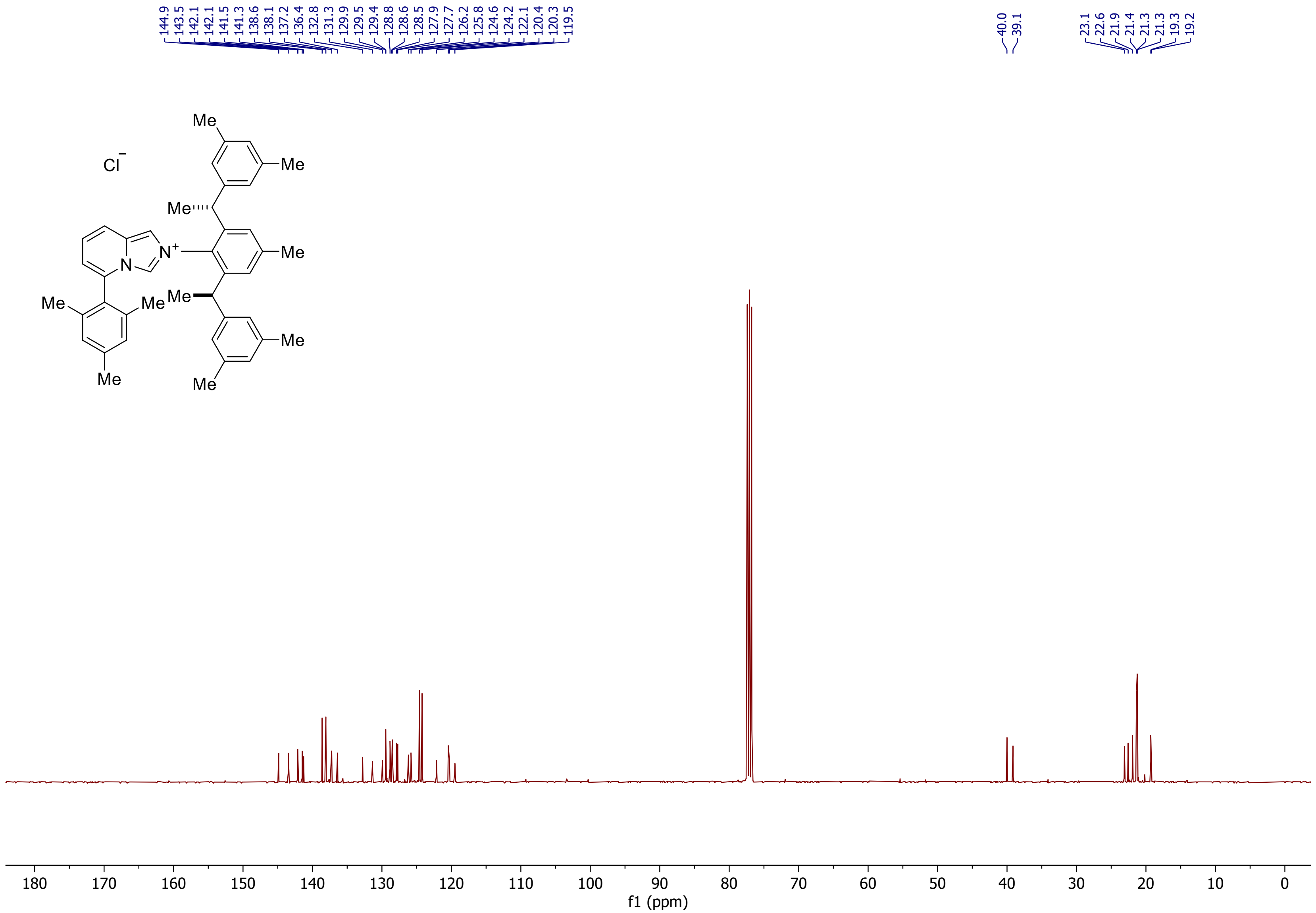

Figure S94. 2D ^1H-^1H COSY spectrum (298 K, $CDCl_3$) of (*R*,*R*)-**5j**

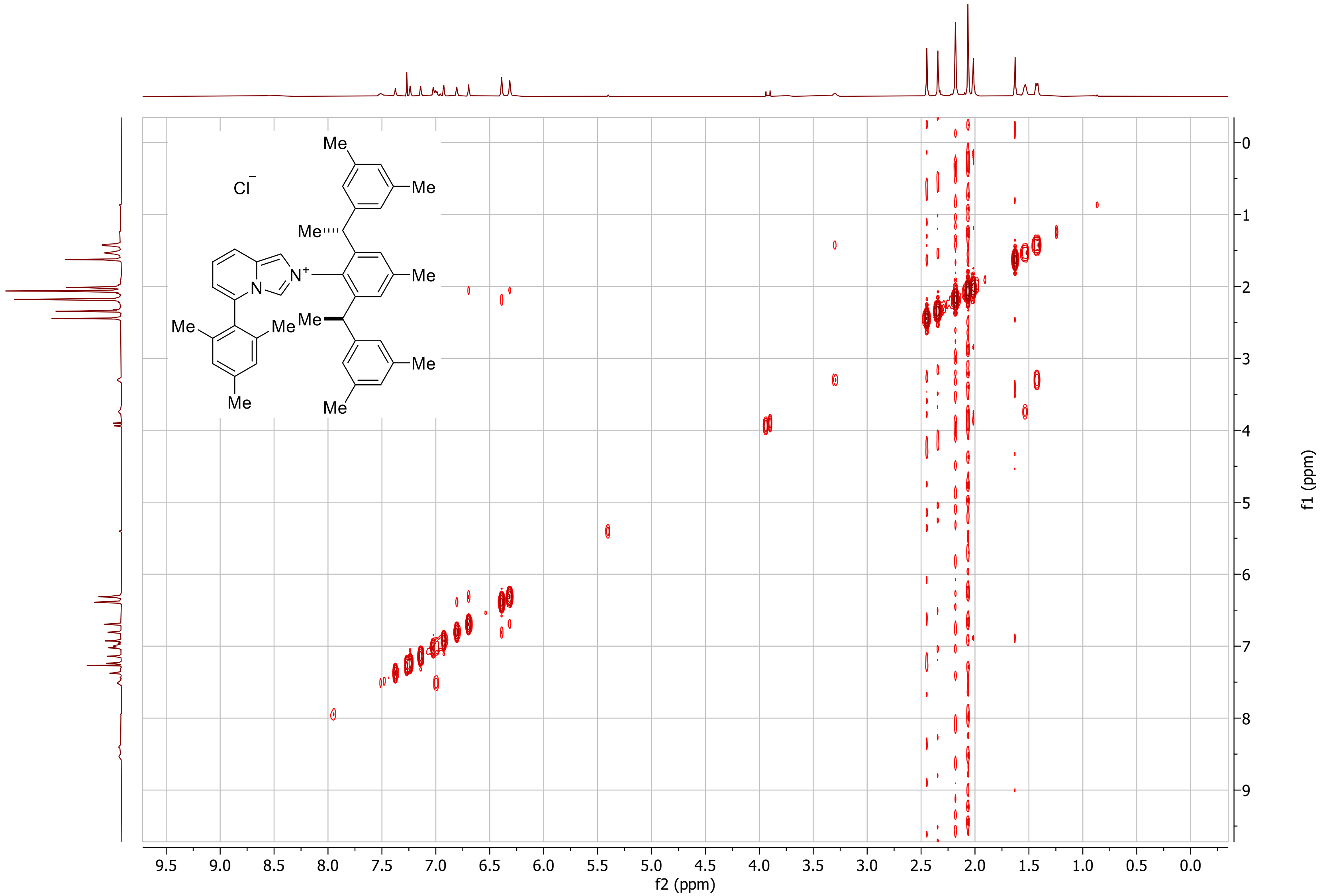

Figure S95. 2D ^{1}H-^{13}C HSQC spectrum (298 K, $CDCl_3$) of (*R*,*R*)-**5j**

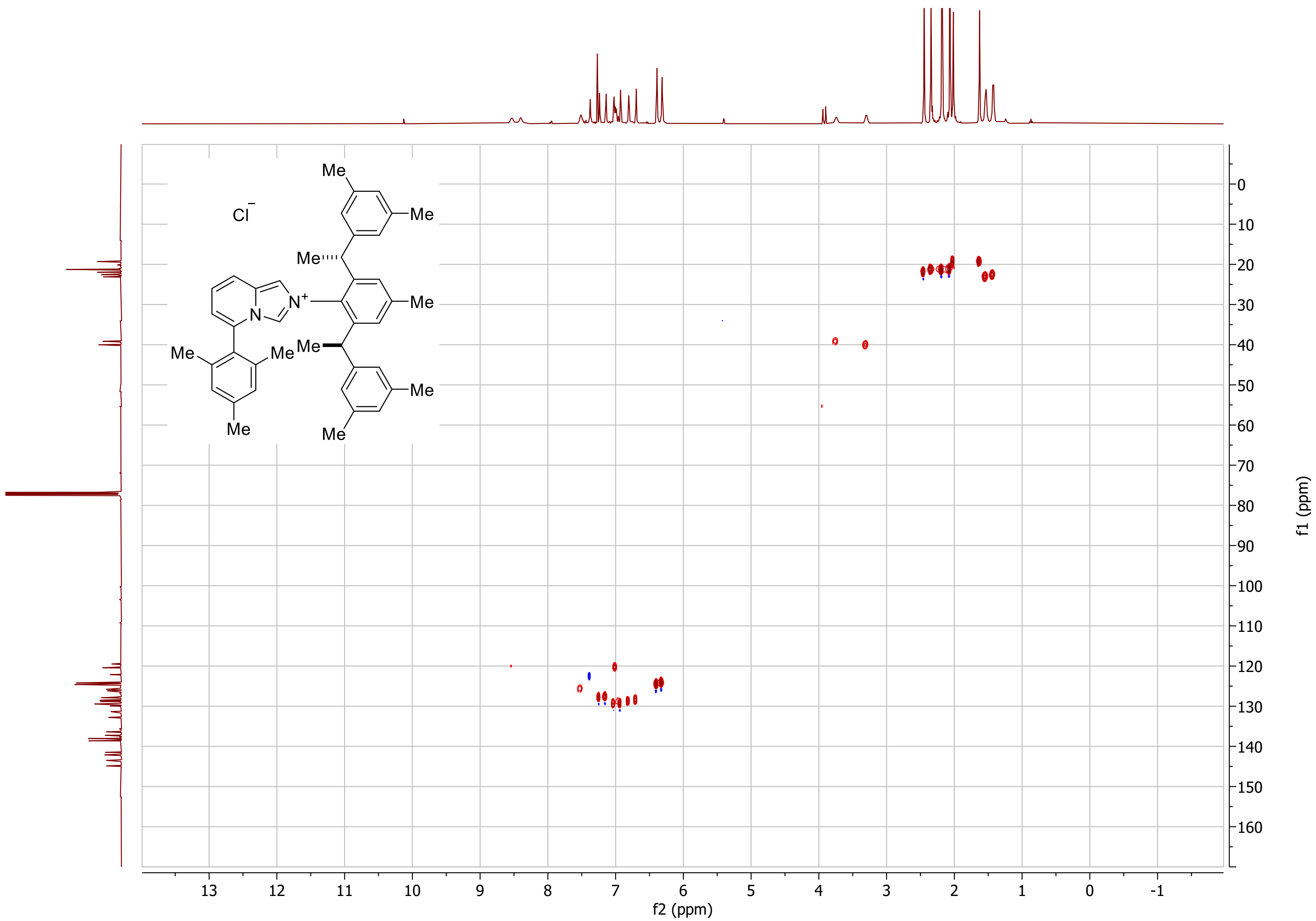

Figure S96. ^{1}H NMR spectrum (400 MHz, 298 K, $CDCl_3$) of (*R,R*)-**6a**

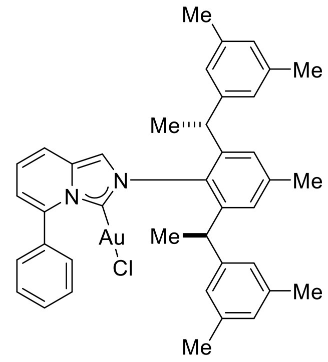

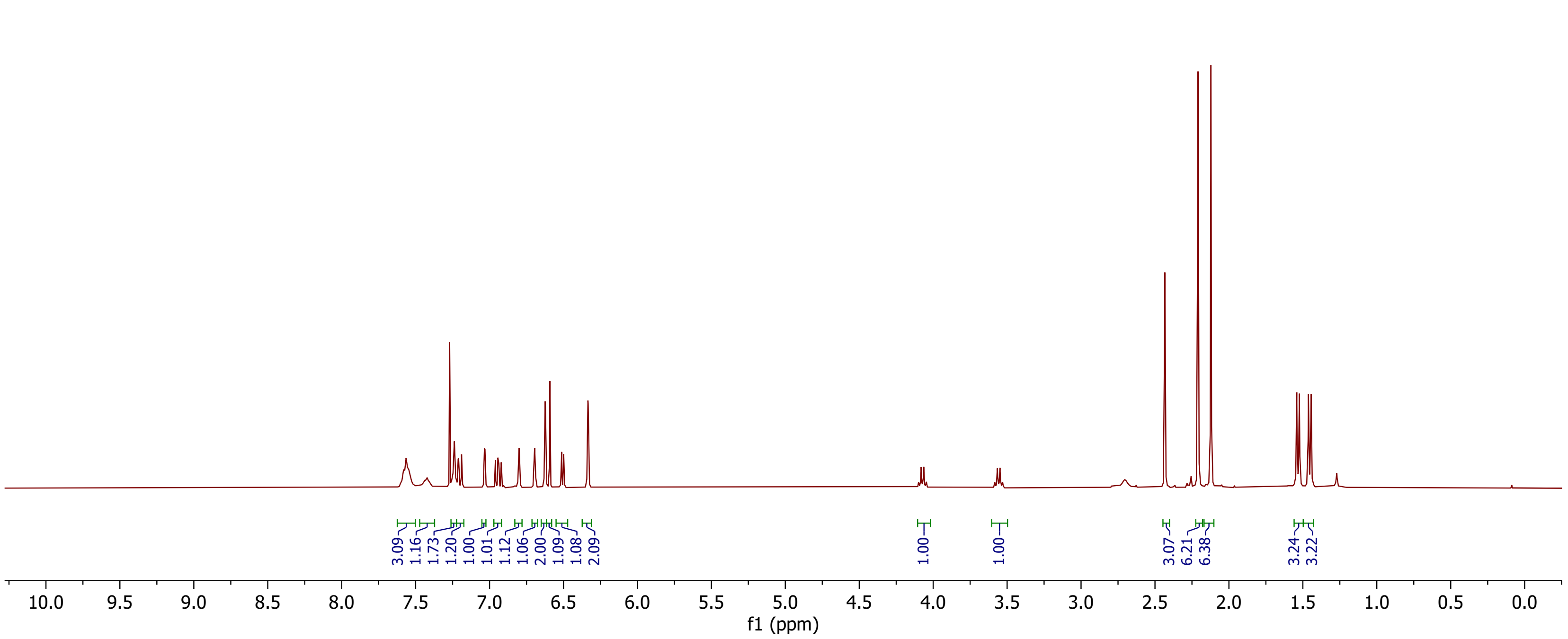

Figure S97. $^{13}C\{^{1}H\}$ NMR spectrum (101 MHz, 298 K, $CDCl_3$) of (*R,R*)-**6a**

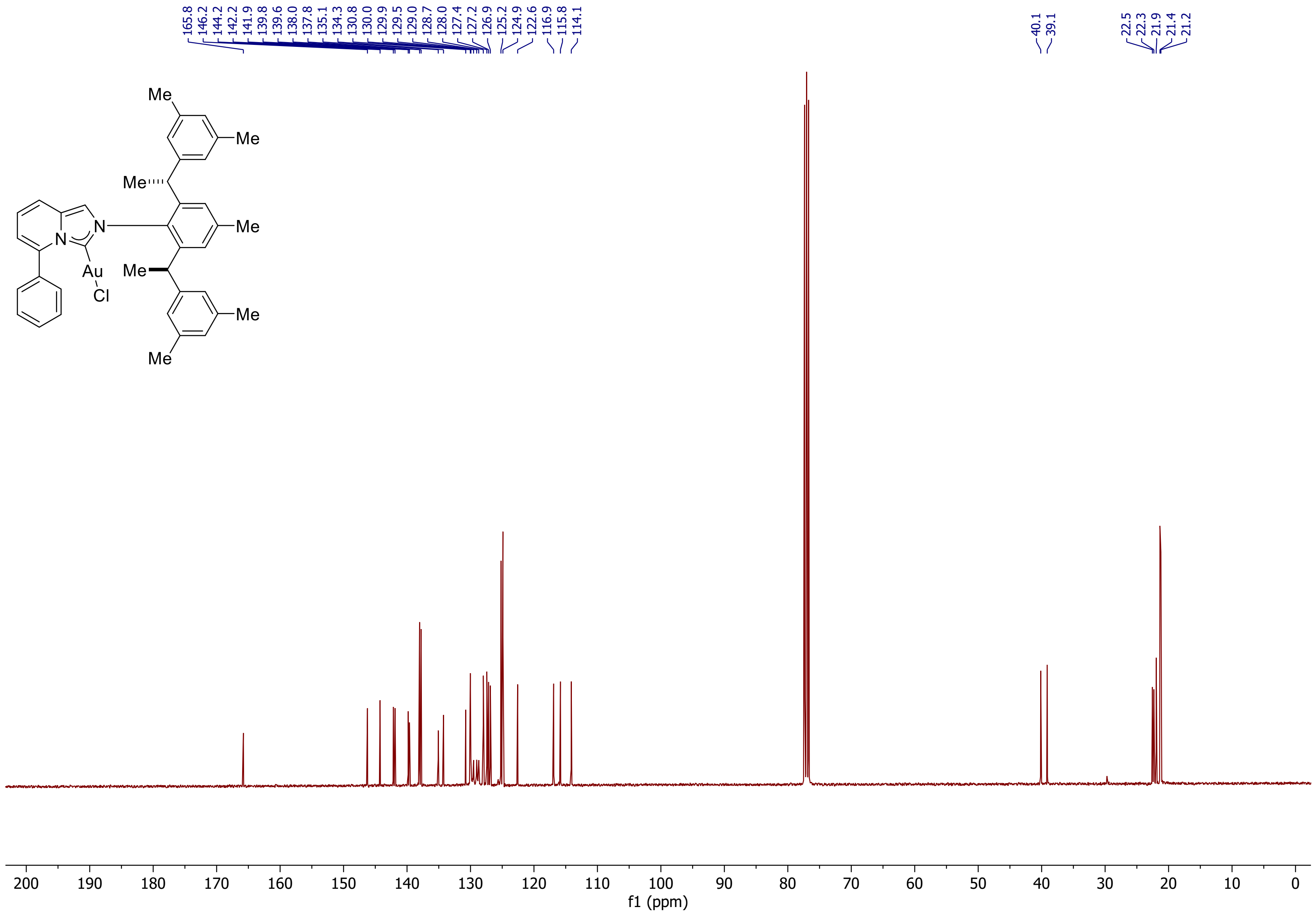

Figure S98. 2D ^{1}H-^{1}H COSY spectrum (298 K, $CDCl_3$) of (*R*,*R*)-**6a**

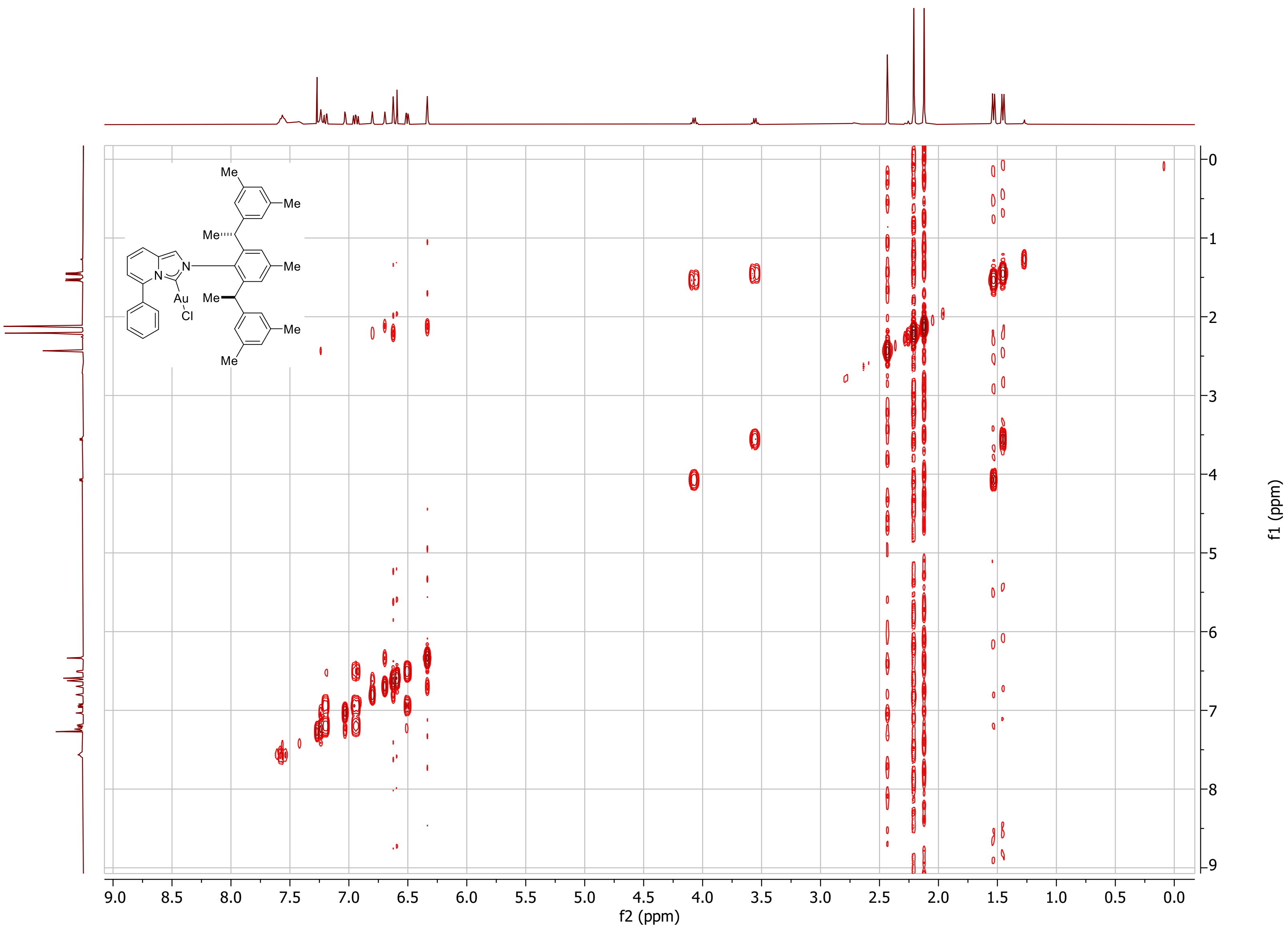

Figure S99. 2D ^{1}H-^{13}C HSQC spectrum (298 K, $CDCl_3$) of (*R*,*R*)-**6a**

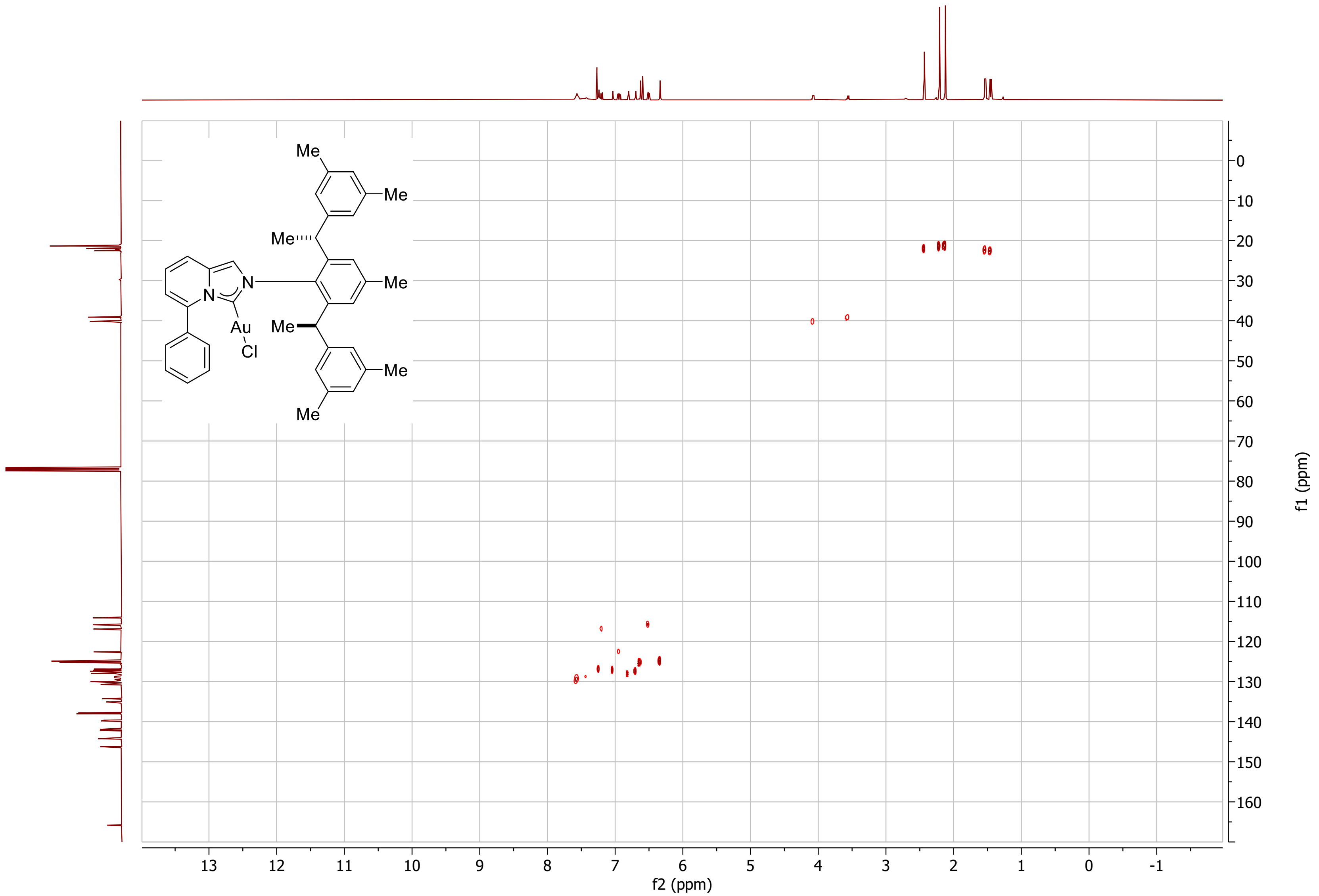

Figure S100. ^{1}H NMR spectrum (400 MHz, 298 K, $CDCl_3$) of (*R*,*R*)-**6b**

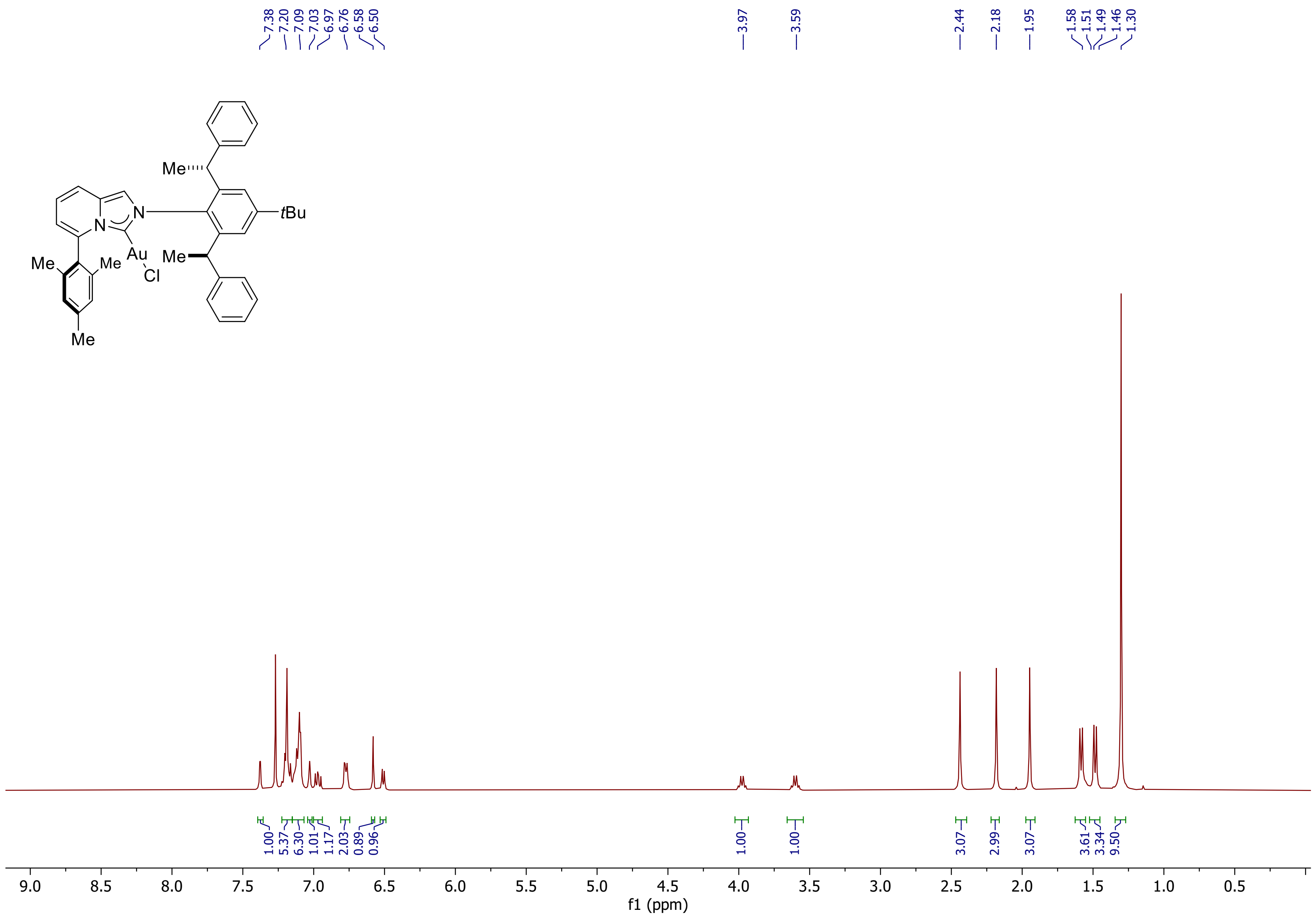

Figure S101. $^{13}C\{^{1}H\}$ NMR spectrum (101 MHz, 298 K, $CDCl_3$) of (*R,R*)-**6b**

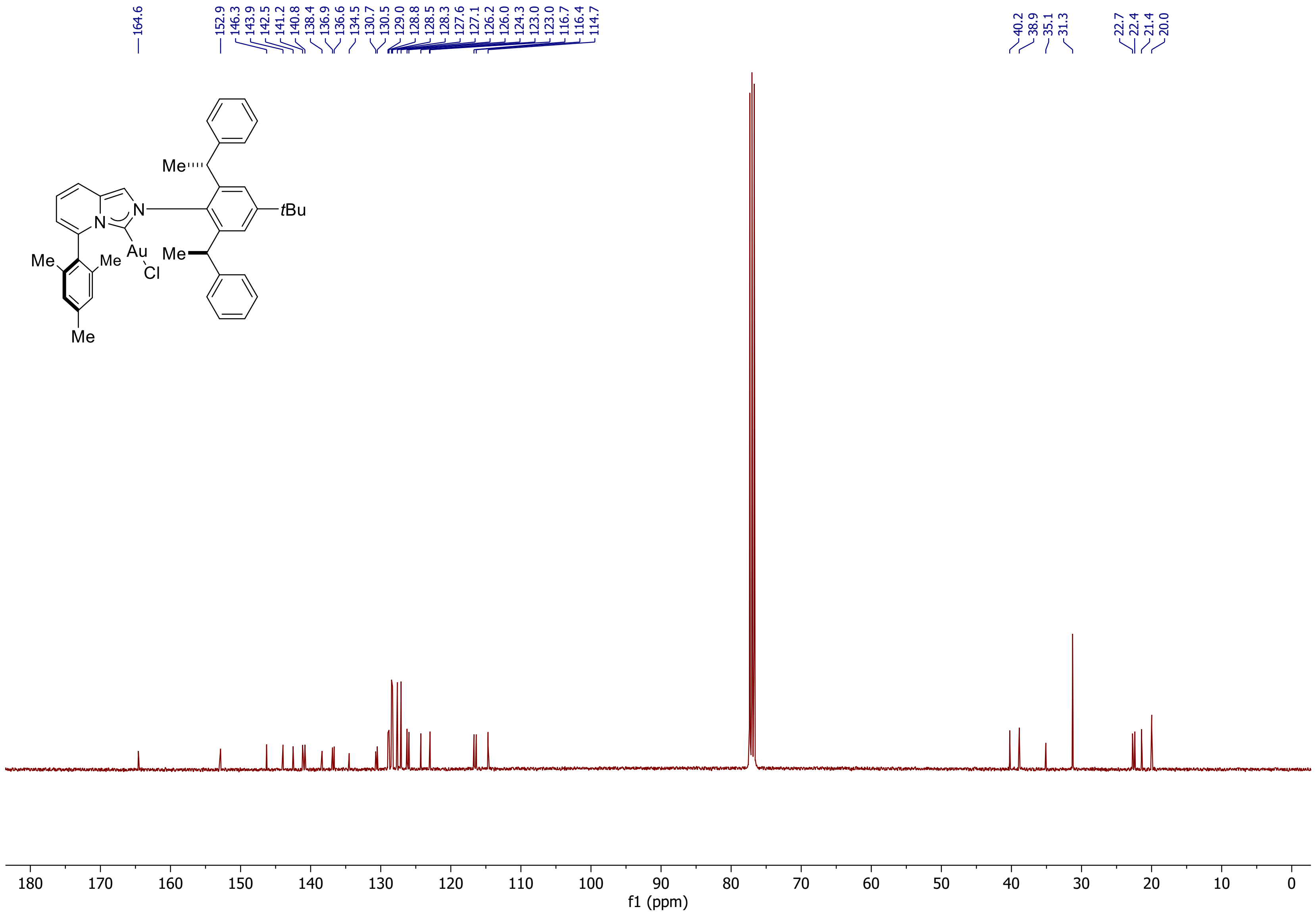

Figure S102. 2D ^{1}H-^{1}H COSY spectrum (298 K, $CDCl_3$) of (*R,R*)-**6b**

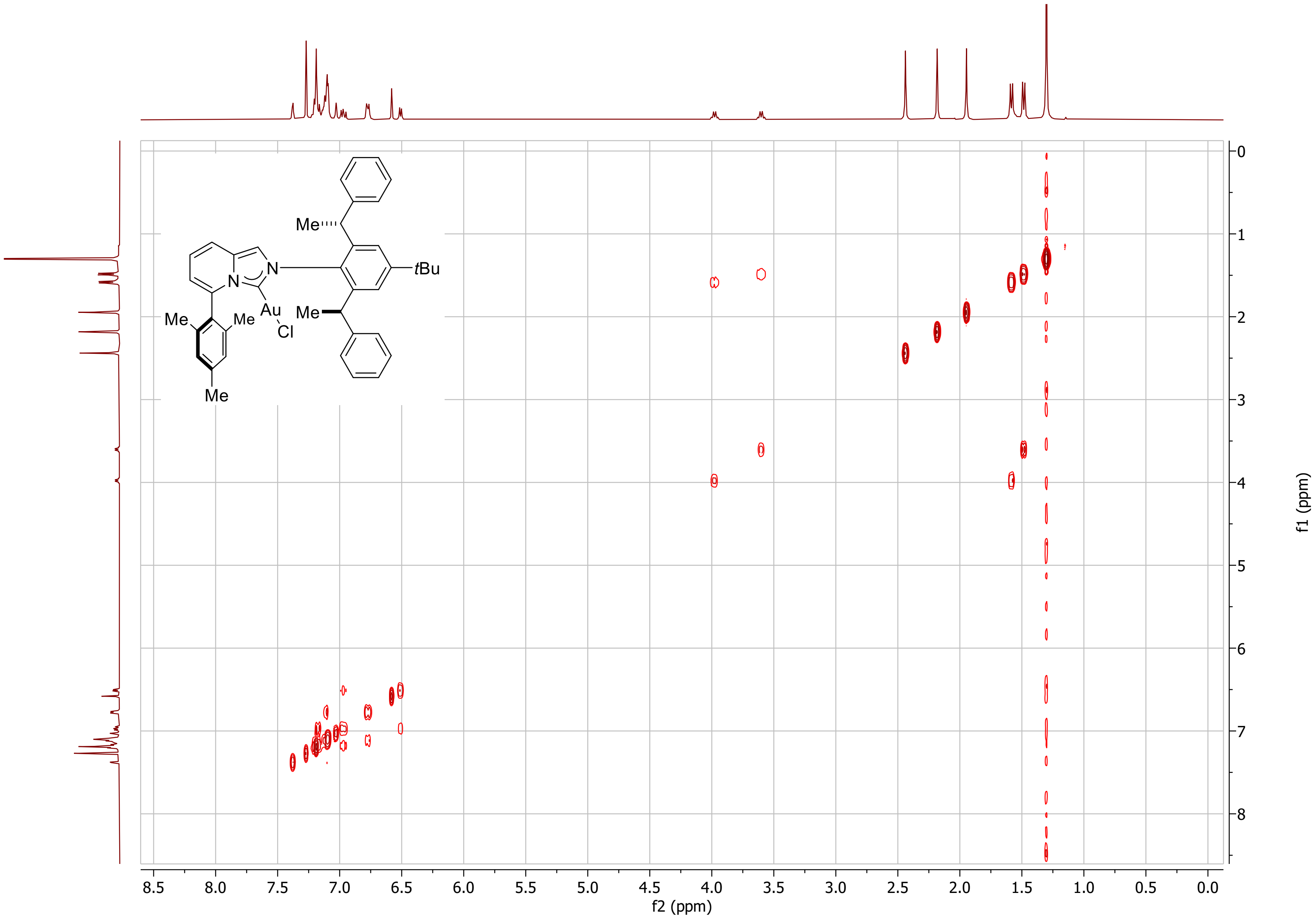

Figure S103. 2D ^{1}H-^{13}C HSQC spectrum (298 K, $CDCl_3$) of (*R,R*)-**6b**

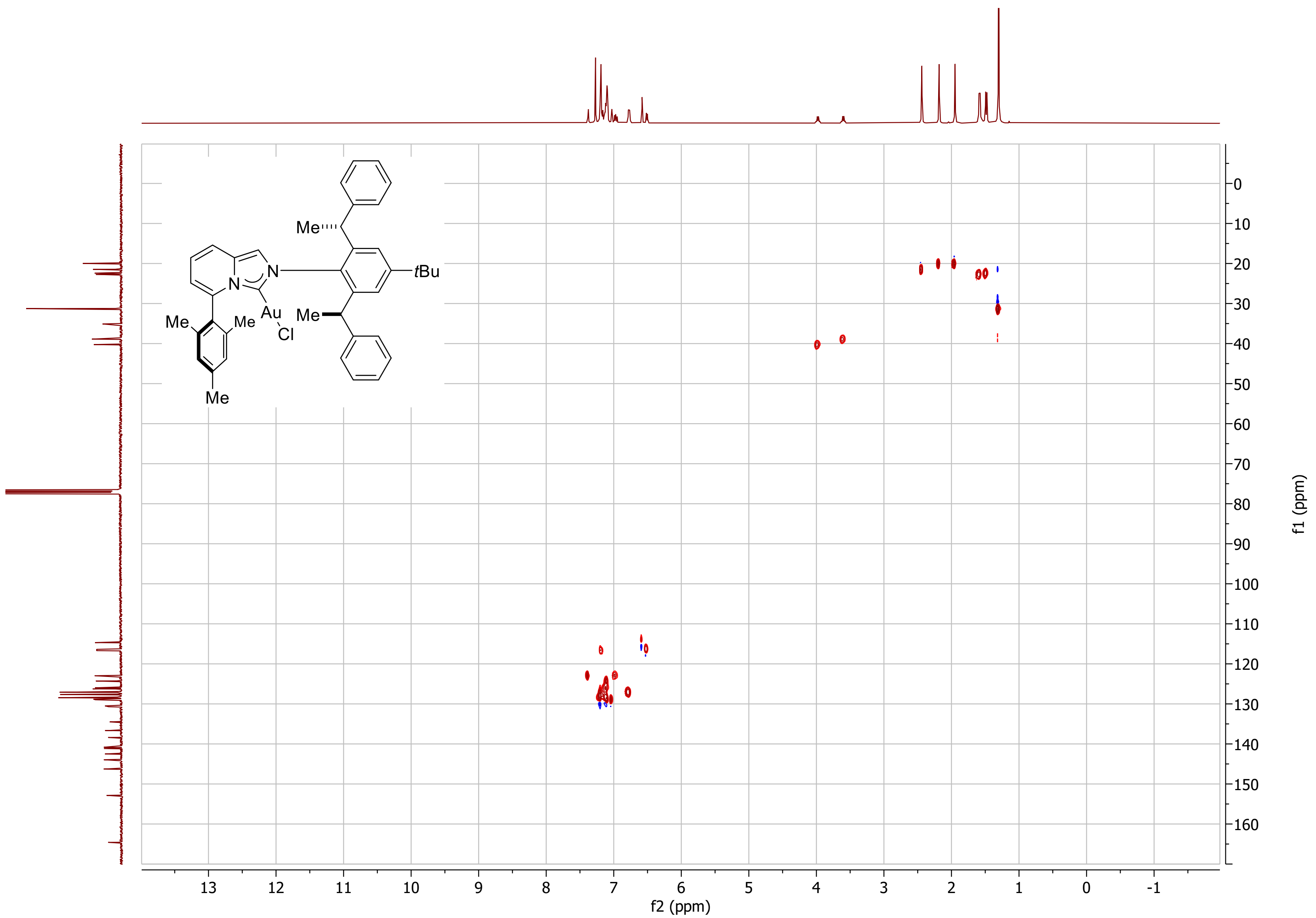

Figure S104. ^{1}H NMR spectrum (400 MHz, 298 K, $CDCl_3$) of (*R,R*)-**6c**

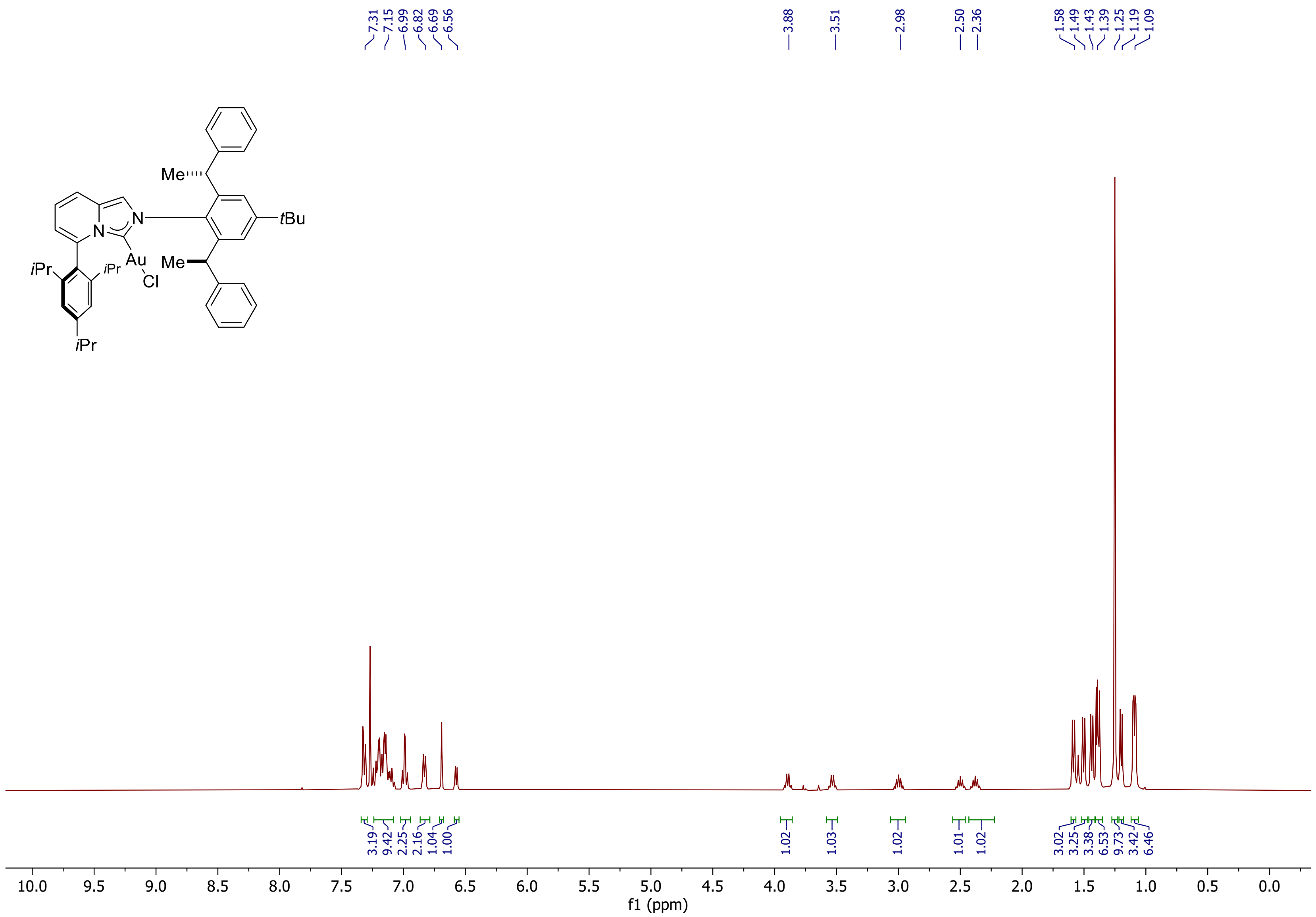

Figure S105. $^{13}C\{^1H\}$ NMR spectrum (101 MHz, 298 K, $CDCl_3$) of (*R,R*)-**6c**

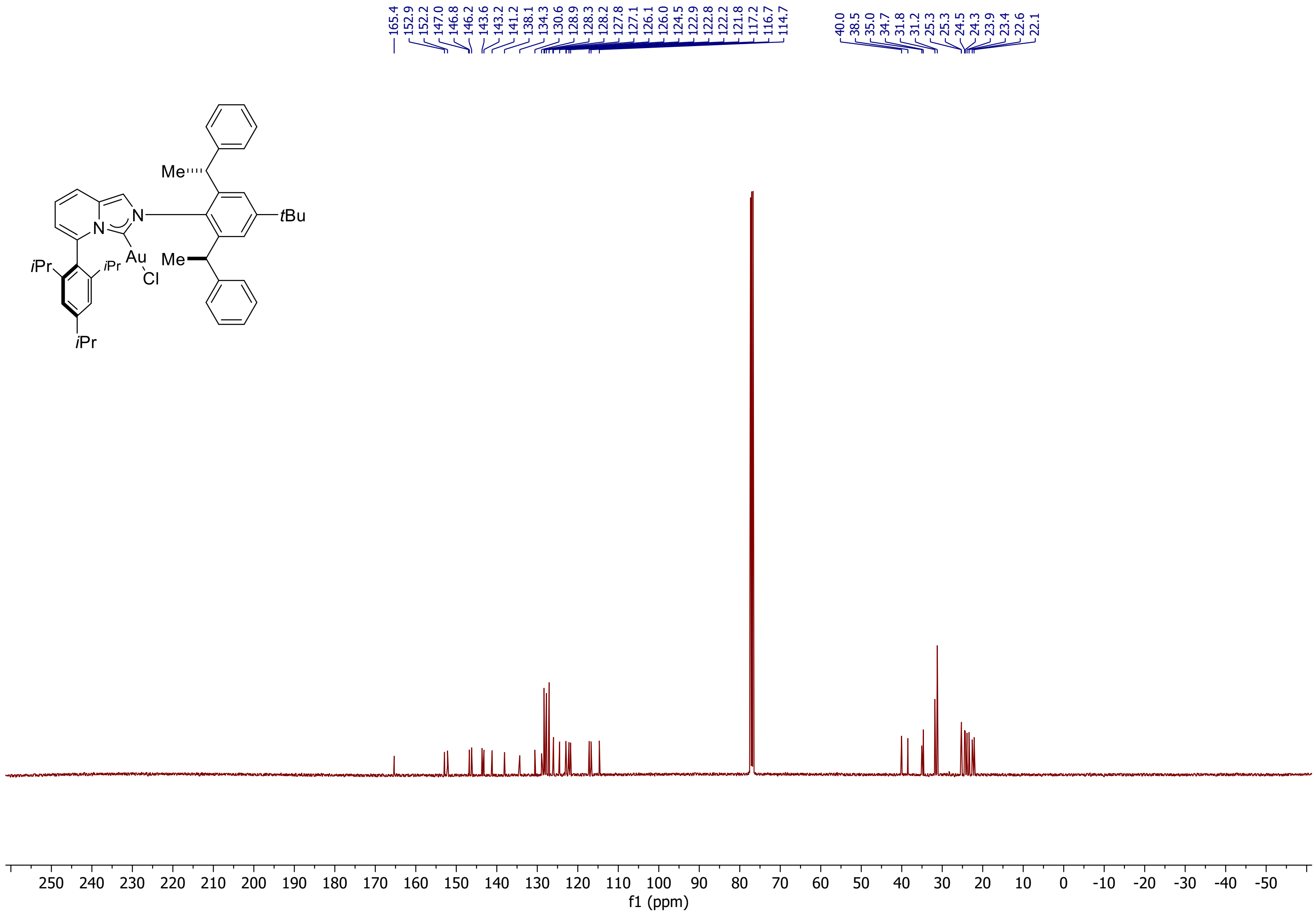

Figure S106. 2D ^{1}H-^{1}H COSY spectrum (298 K, $CDCl_3$) of (*R,R*)-**6c**

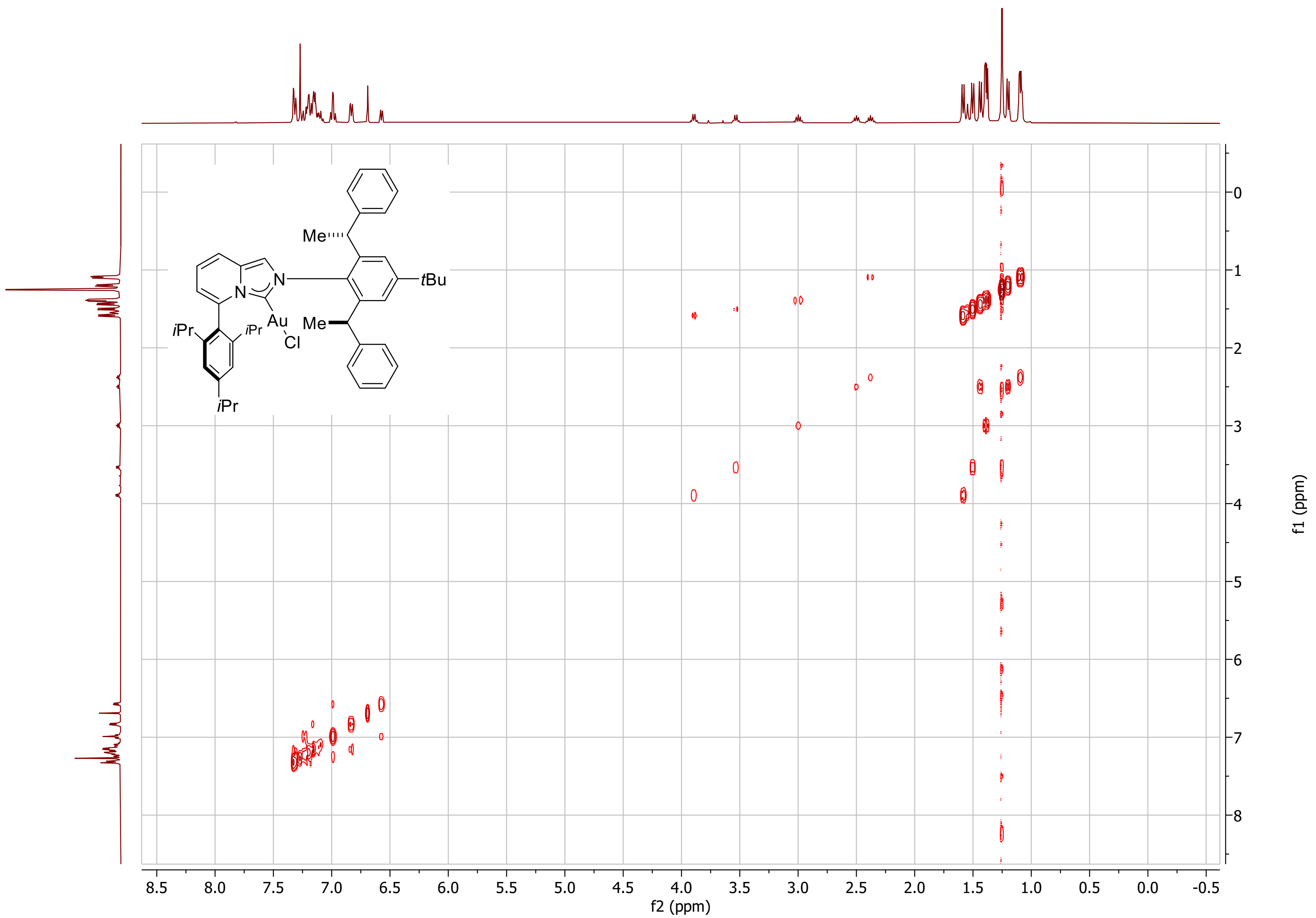

Figure S107. 2D ^{1}H-^{13}C HSQC spectrum (298 K, $CDCl_3$) of (*R*,*R*)-**6c**

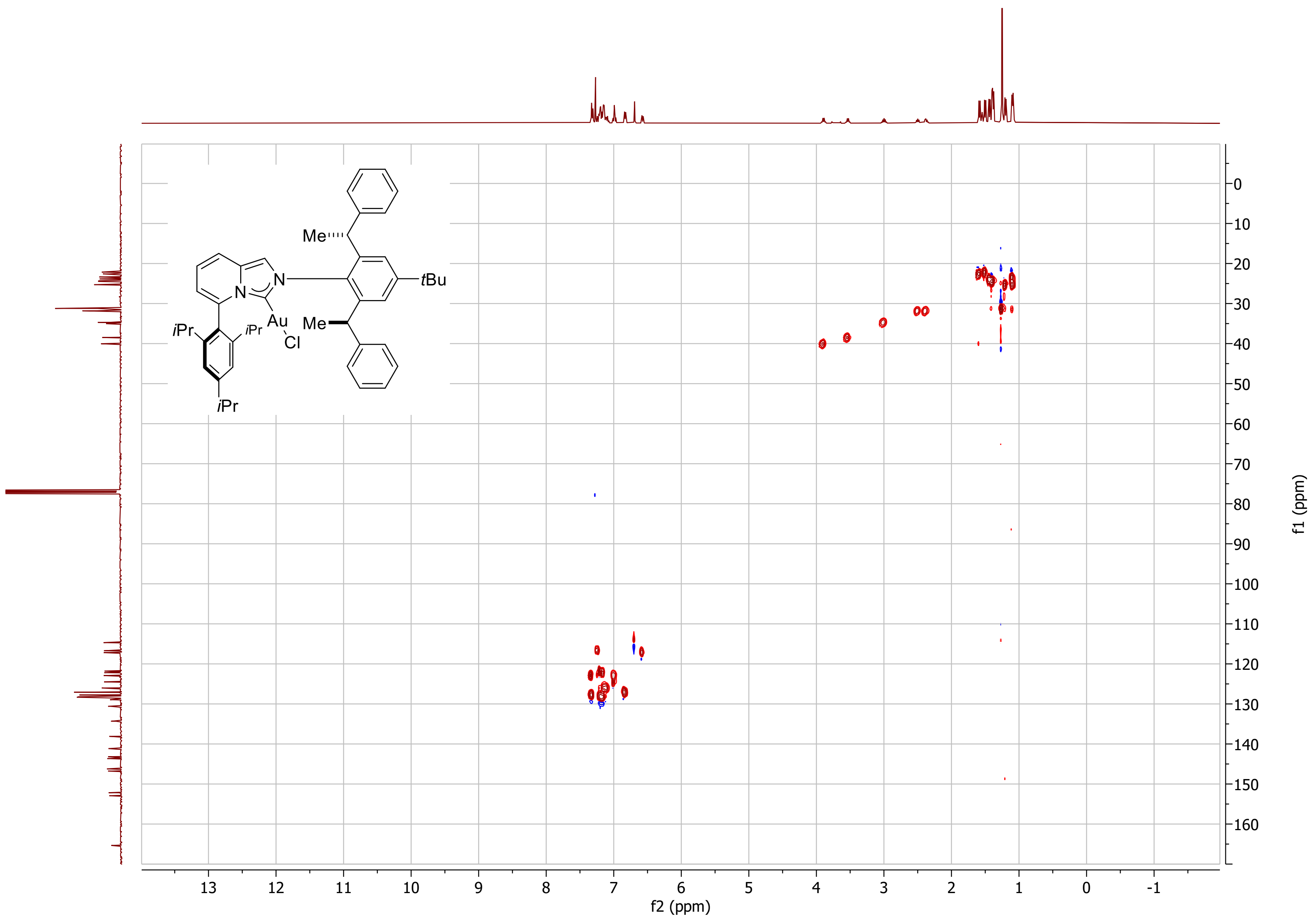

Figure S108. ^{1}H NMR spectrum (400 MHz, 298 K, $CDCl_3$) of (*R*,*R*)-**6d**

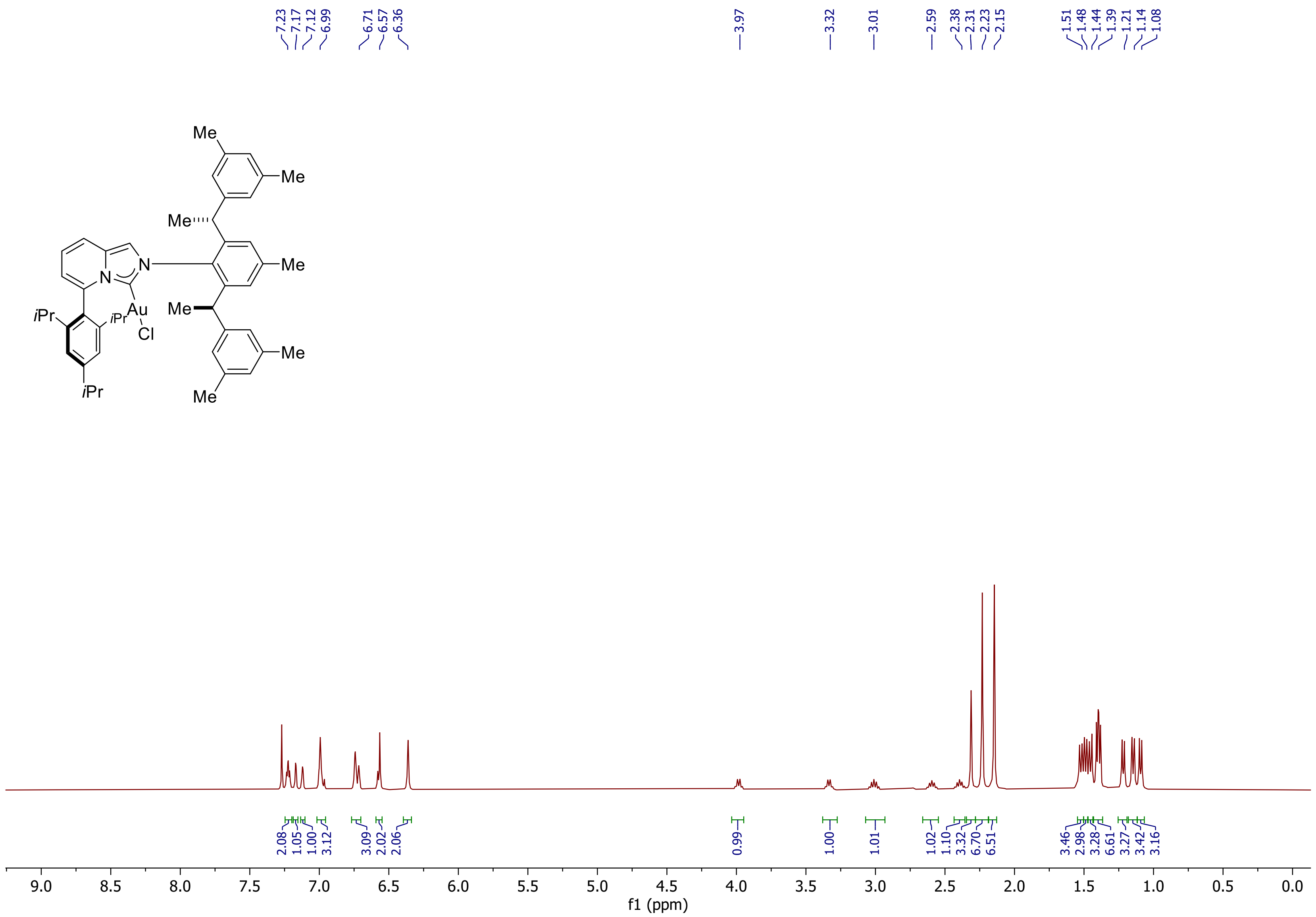

Figure S109. $^{13}C\{^{1}H\}$ NMR spectrum (101 MHz, 298 K, $CDCl_3$) of (*R,R*)-**6d**

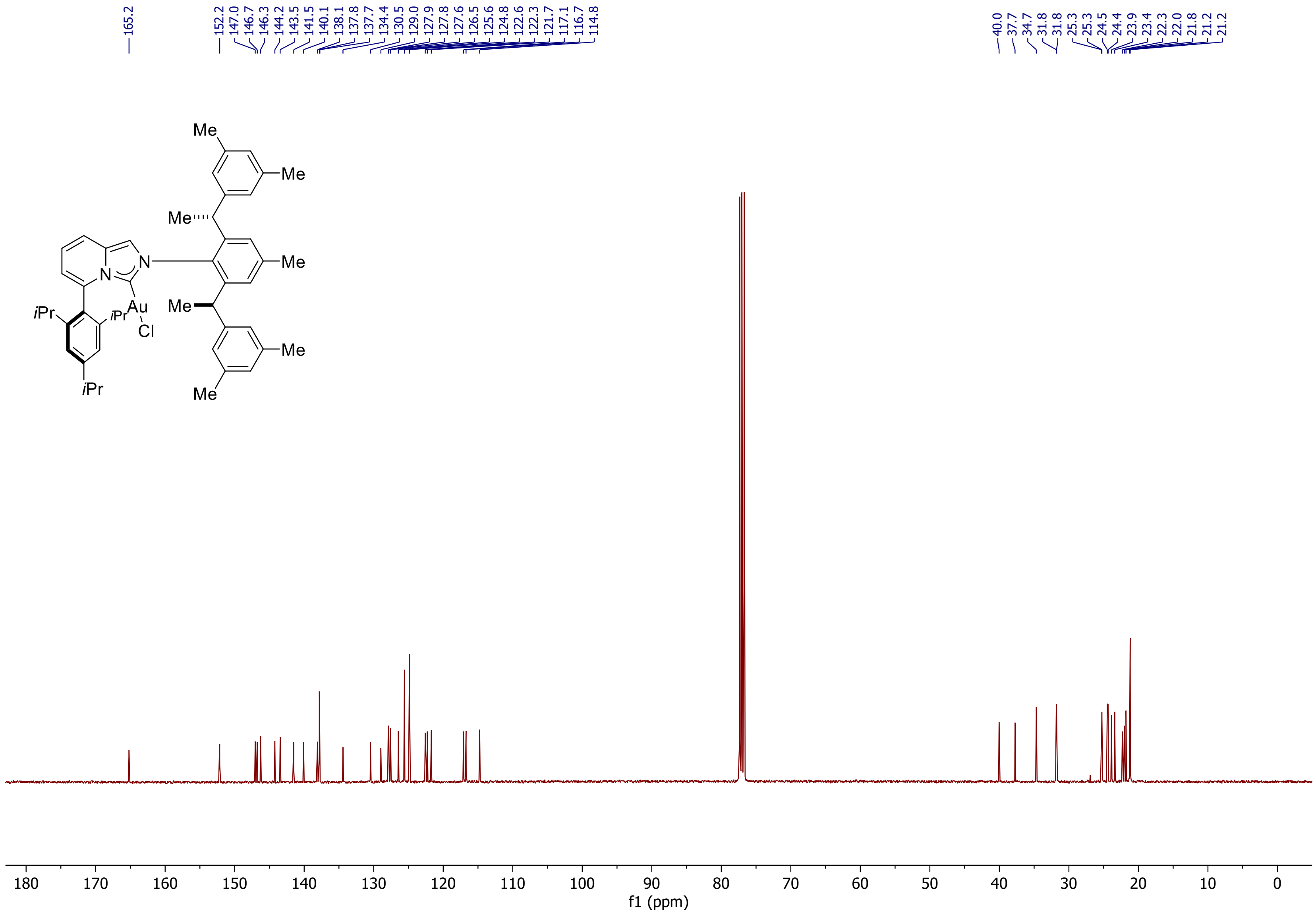

Figure S110. 2D ^{1}H-^{1}H COSY spectrum (298 K, $CDCl_3$) of (*R,R*)-**6d**

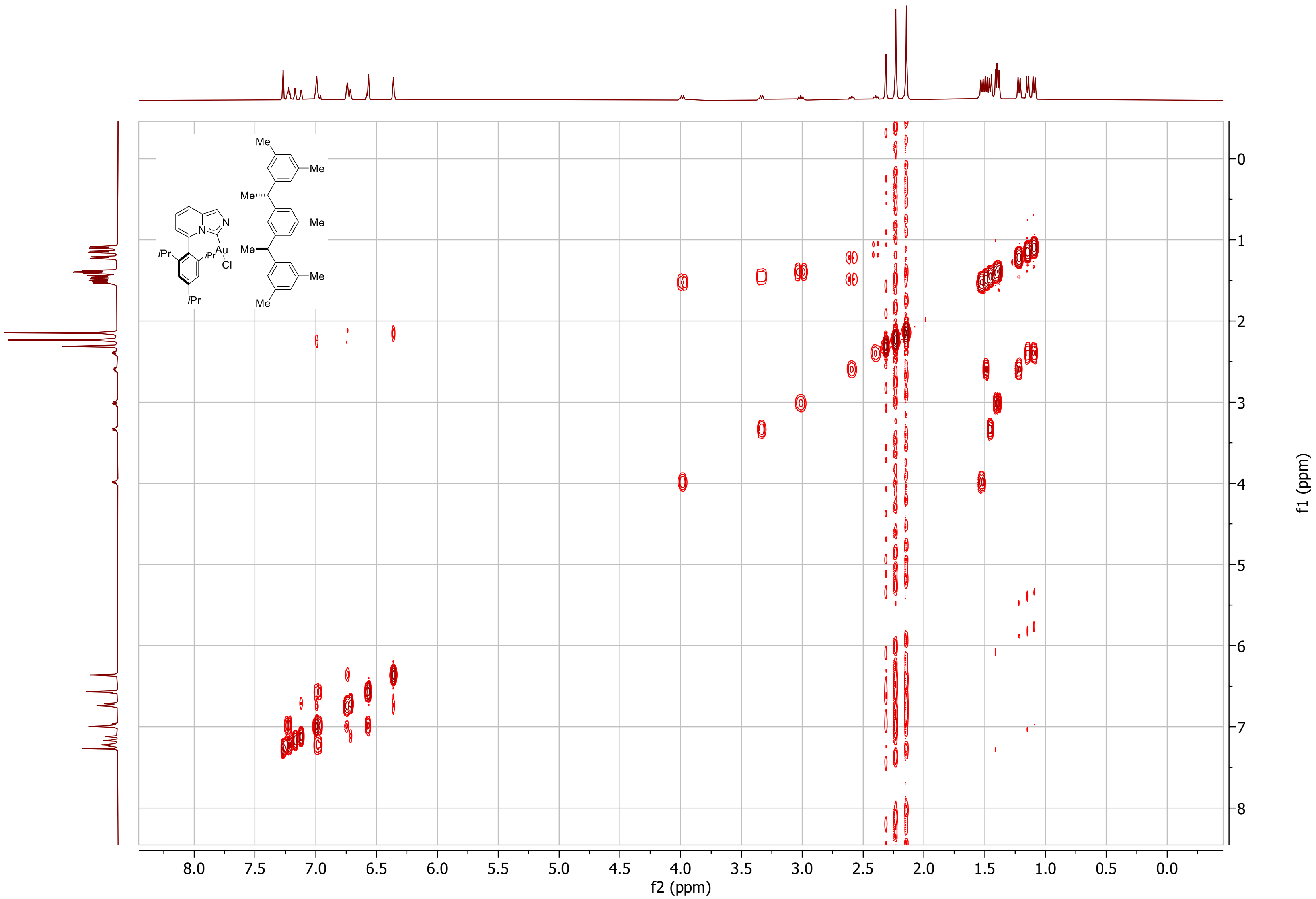

Figure S111. 2D ^{1}H-^{13}C HSQC spectrum (298 K, $CDCl_3$) of (*R,R*)-**6d**

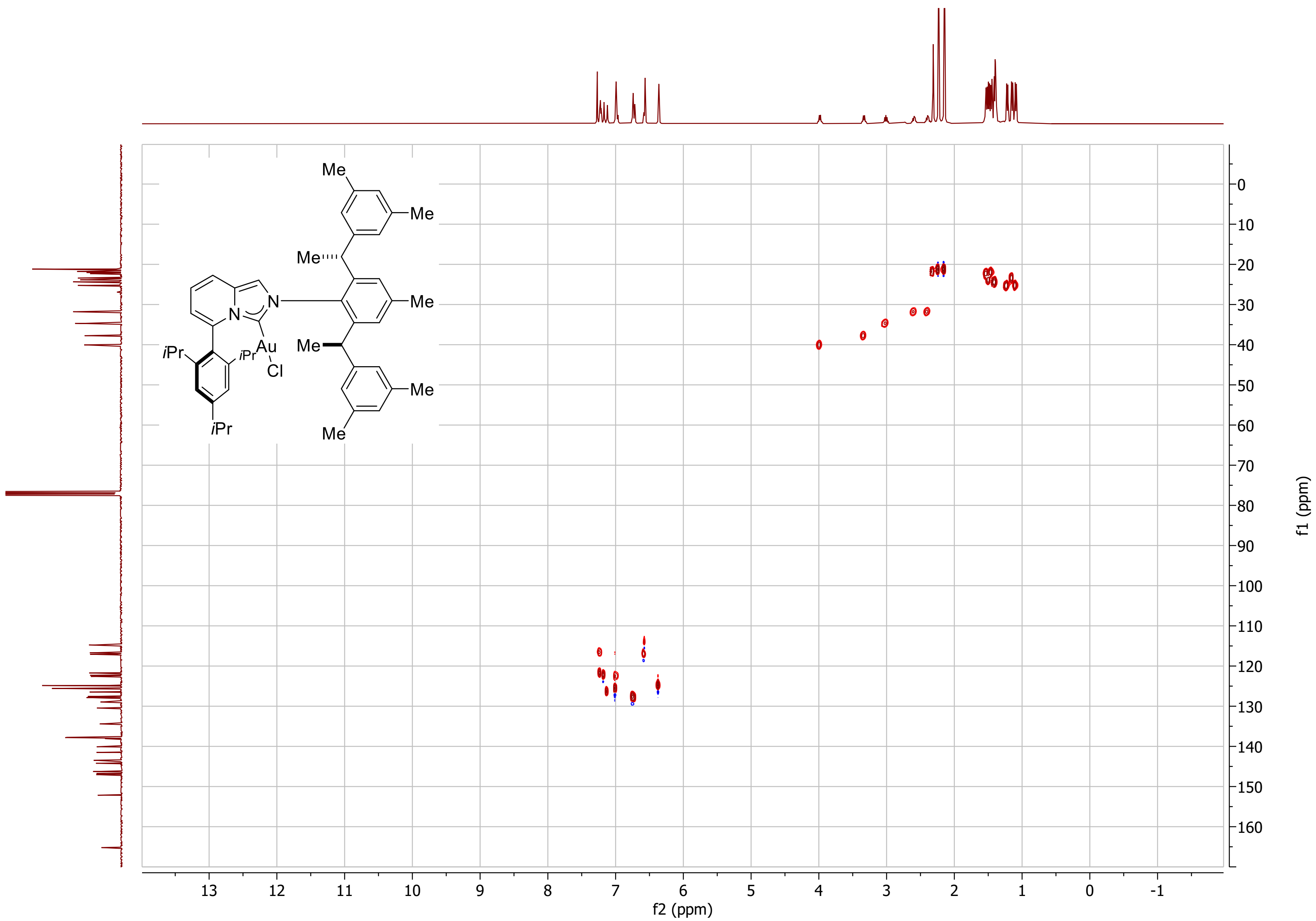

Figure S112. ^{1}H NMR spectrum (400 MHz, 298 K, $CDCl_3$) of (*R,R*)-**6e**

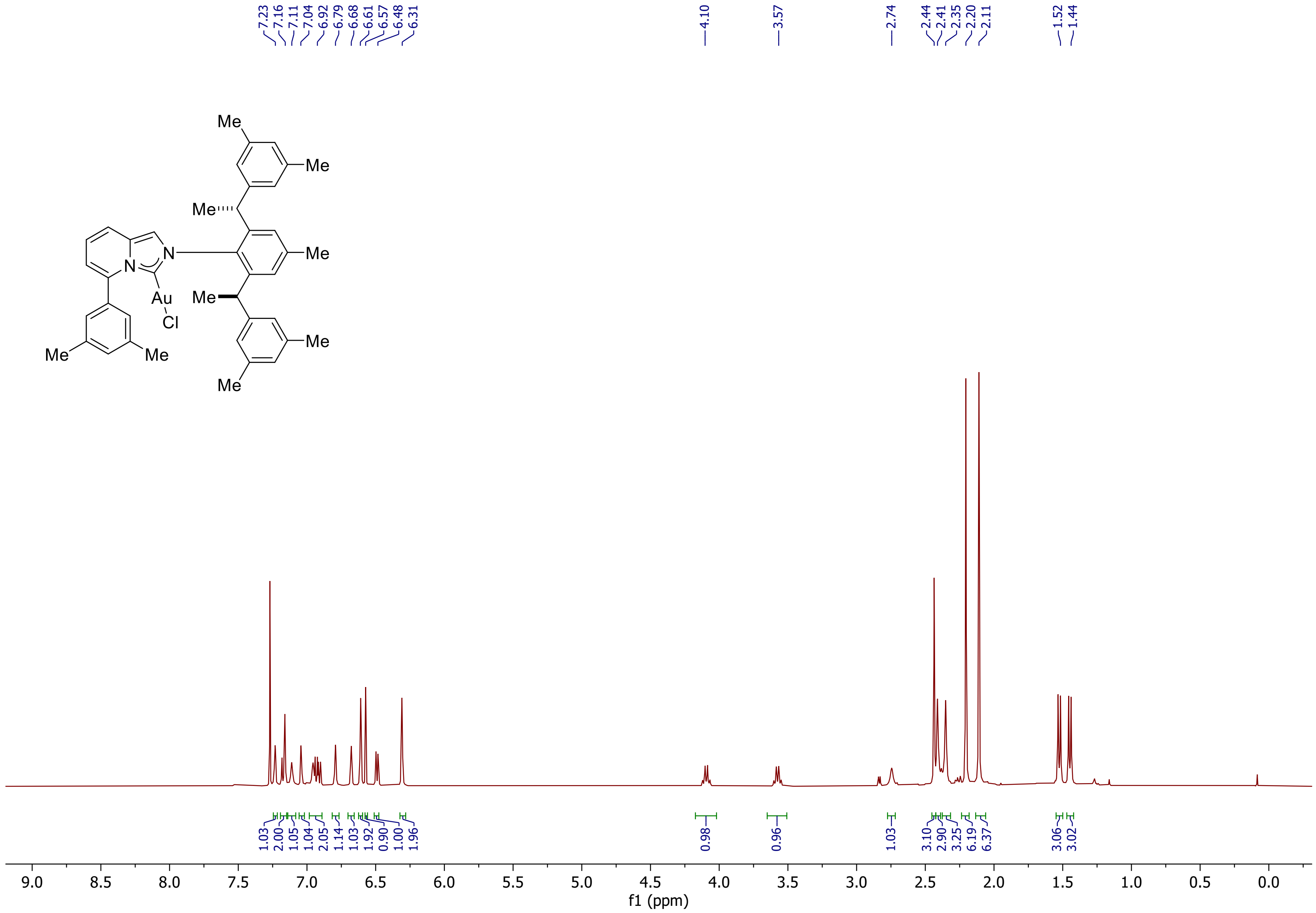

Figure S113. $^{13}C\{^{1}H\}$ NMR spectrum (101 MHz, 298 K, $CDCl_3$) of (*R,R*)-**6e**

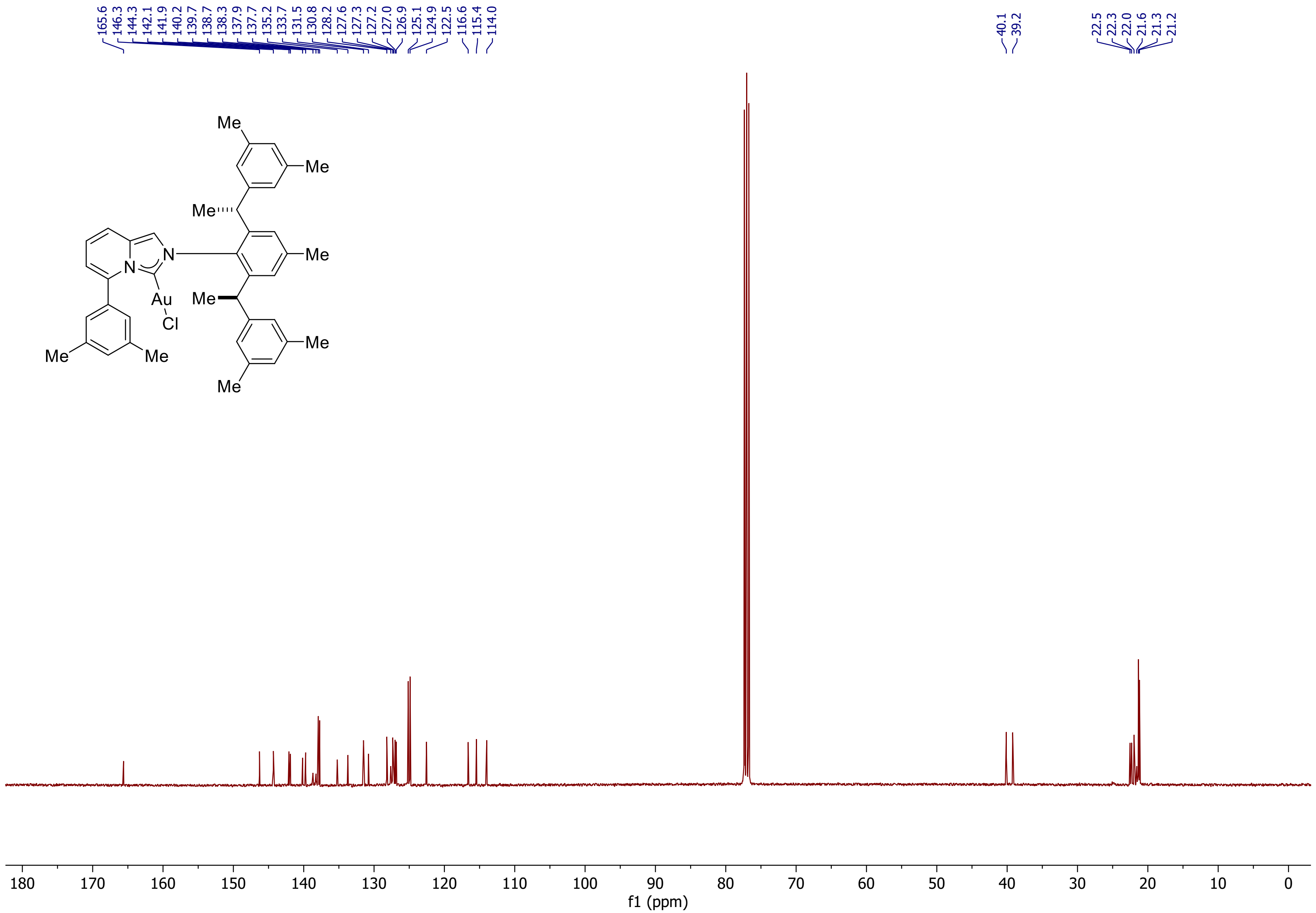

Figure S114. 2D ^{1}H-^{1}H COSY spectrum (298 K, $CDCl_3$) of (*R,R*)-**6e**

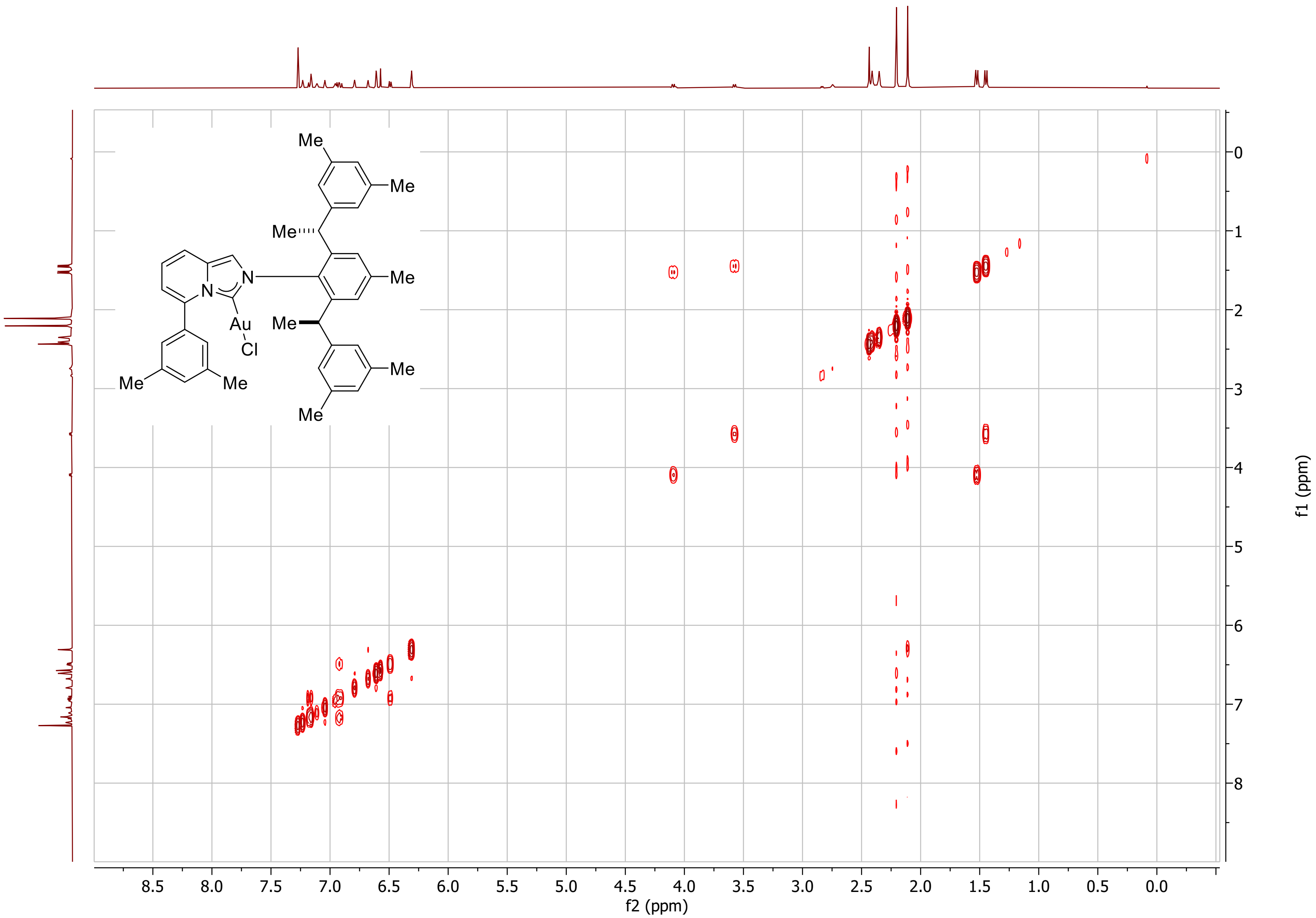

Figure S115. 2D ^1H-^{13}C HSQC spectrum (298 K, $CDCl_3$) of (*R,R*)-**6e**

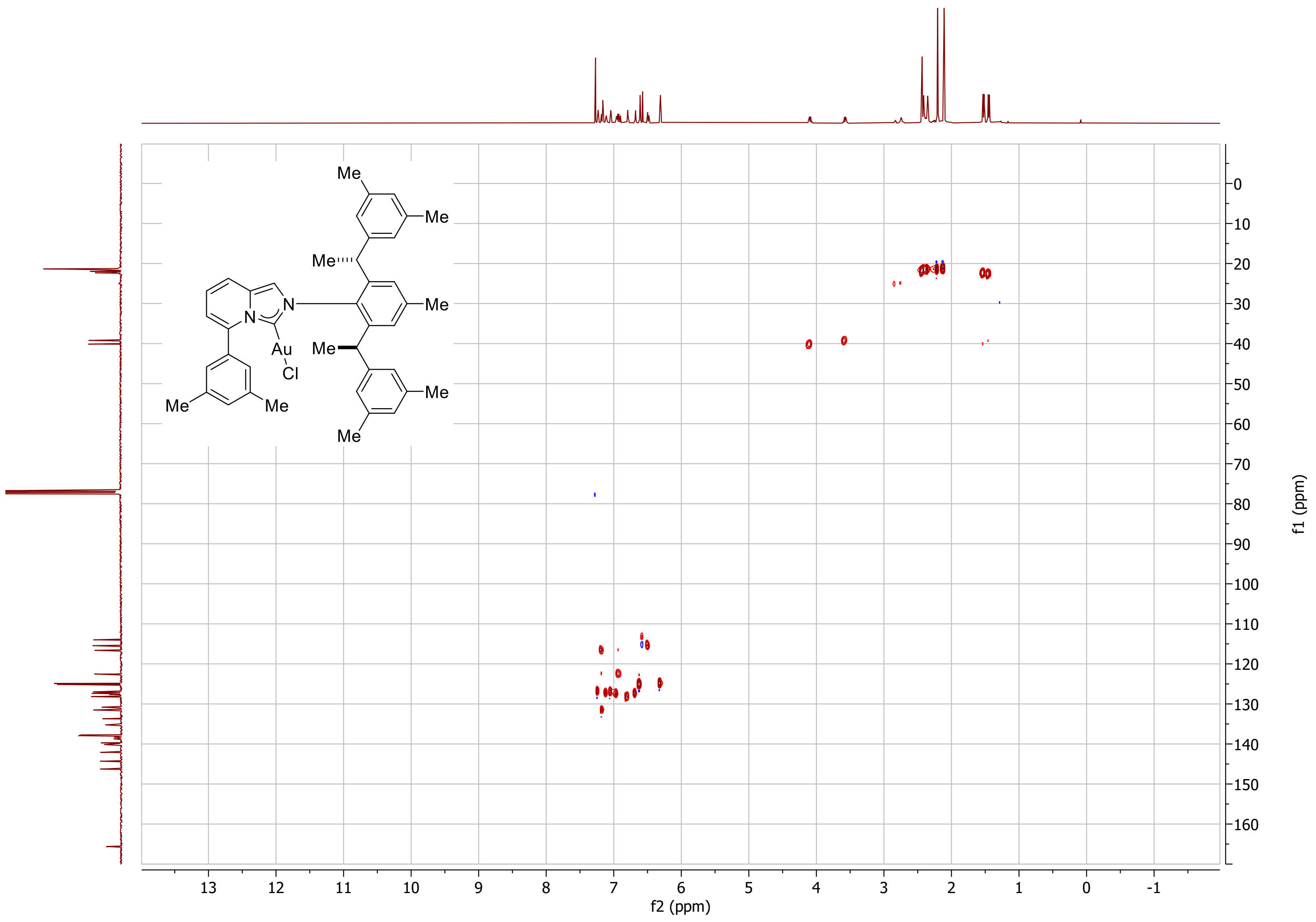

Figure S116. ^{1}H NMR spectrum (400 MHz, 298 K, $CDCl_3$) of (*R,R*)-**6f**

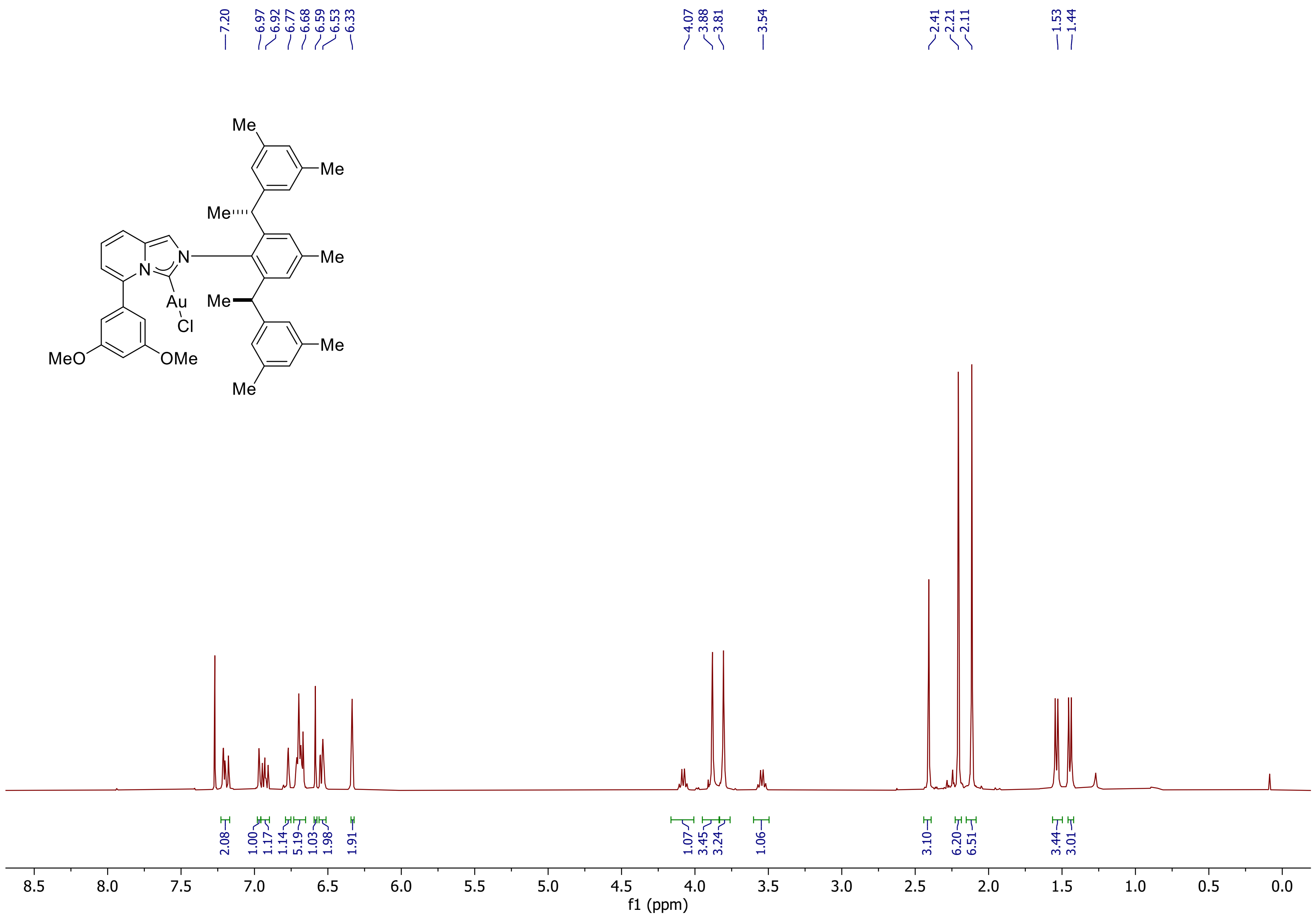

Figure S117. $^{13}C\{^{1}H\}$ NMR spectrum (101 MHz, 298 K, $CDCl_3$) of (*R,R*)-**6f**

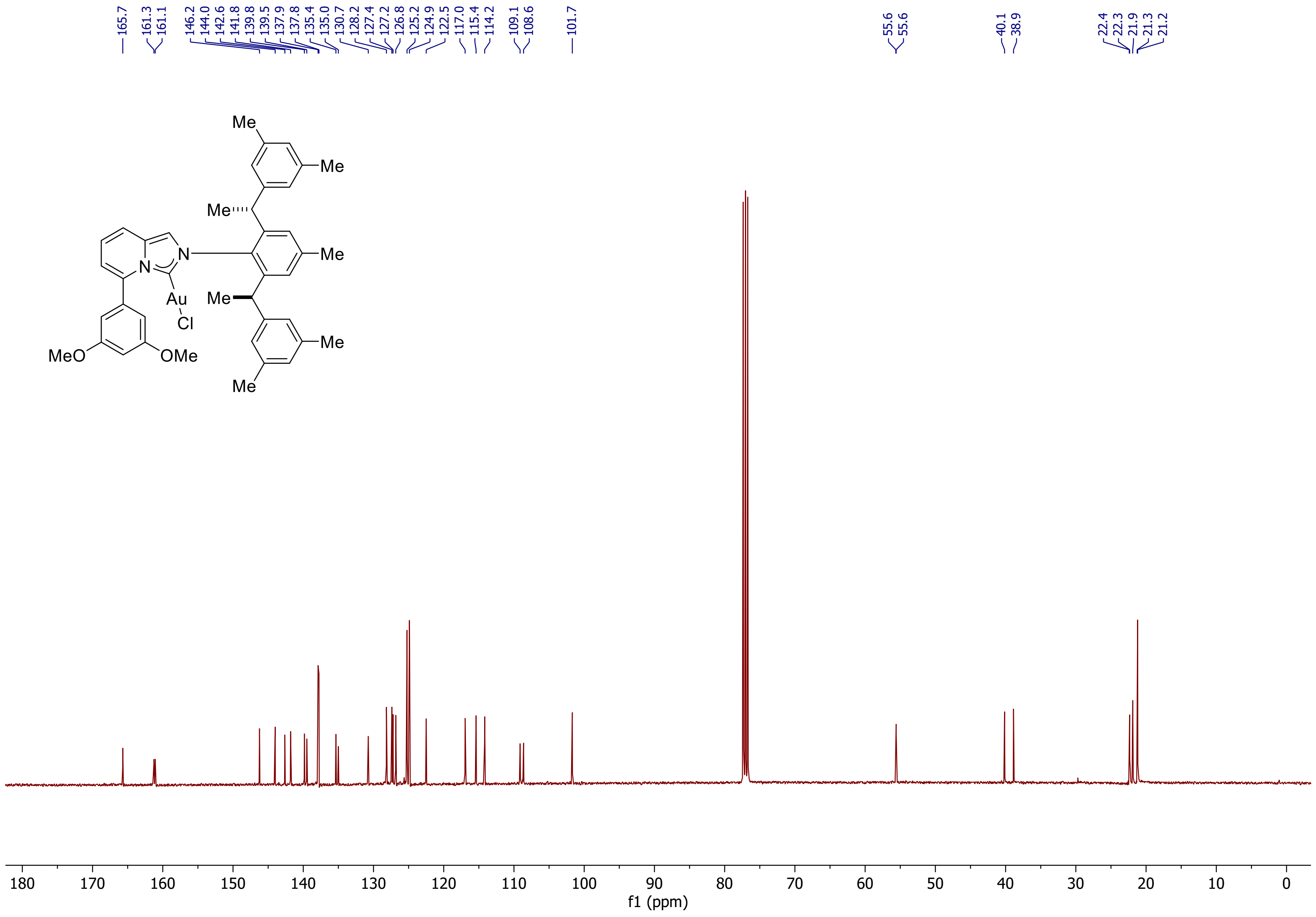

Figure S118. 2D ^1H-^1H COSY spectrum (298 K, $CDCl_3$) of (*R*,*R*)-**6f**

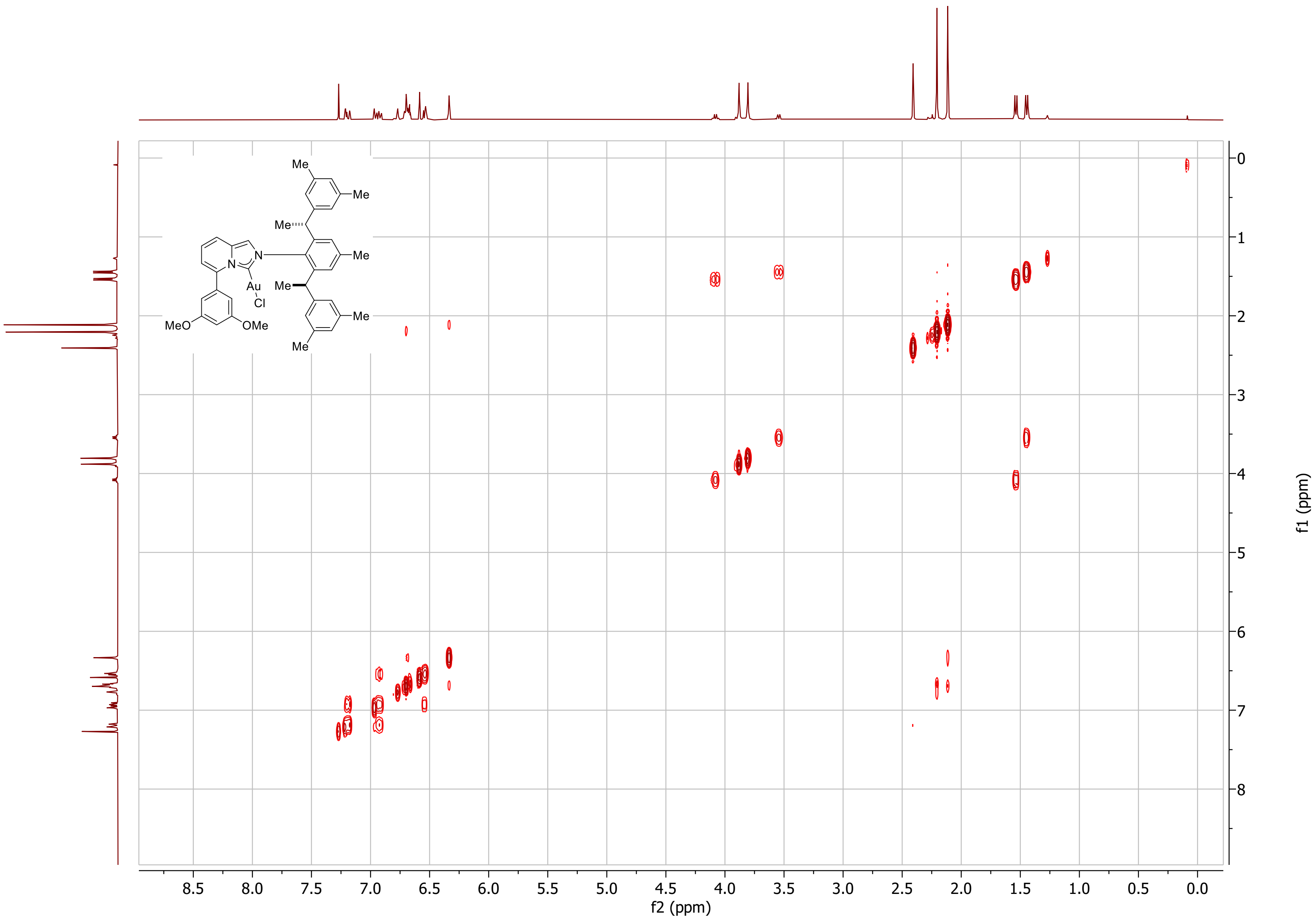

Figure S119. 2D ^{1}H-^{13}C HSQC spectrum (298 K, $CDCl_3$) of (*R*,*R*)-**6f**

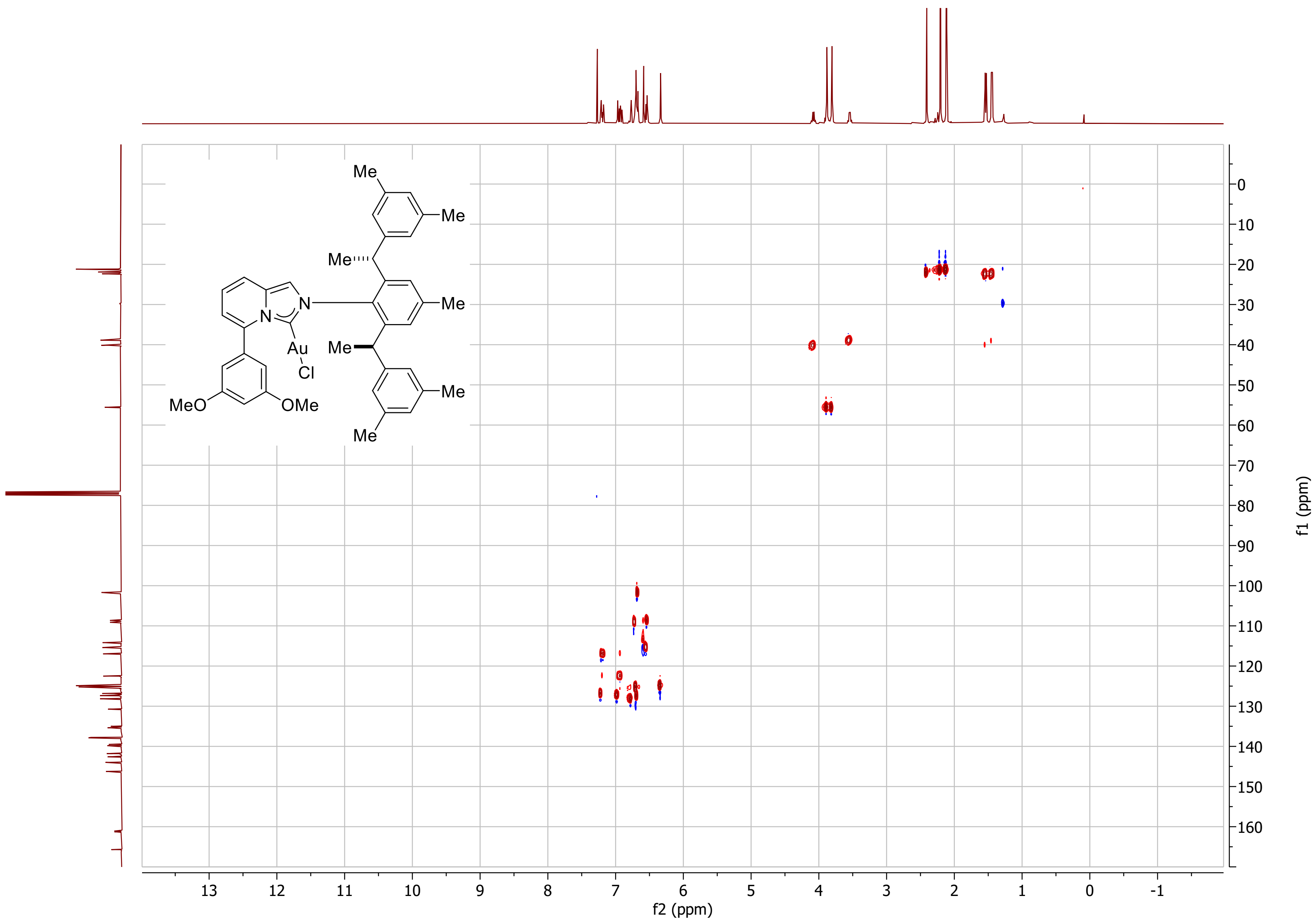

Figure S120. ^{1}H NMR spectrum (400 MHz, 298 K, $CDCl_3$) of (*R*,*R*)-**6g**

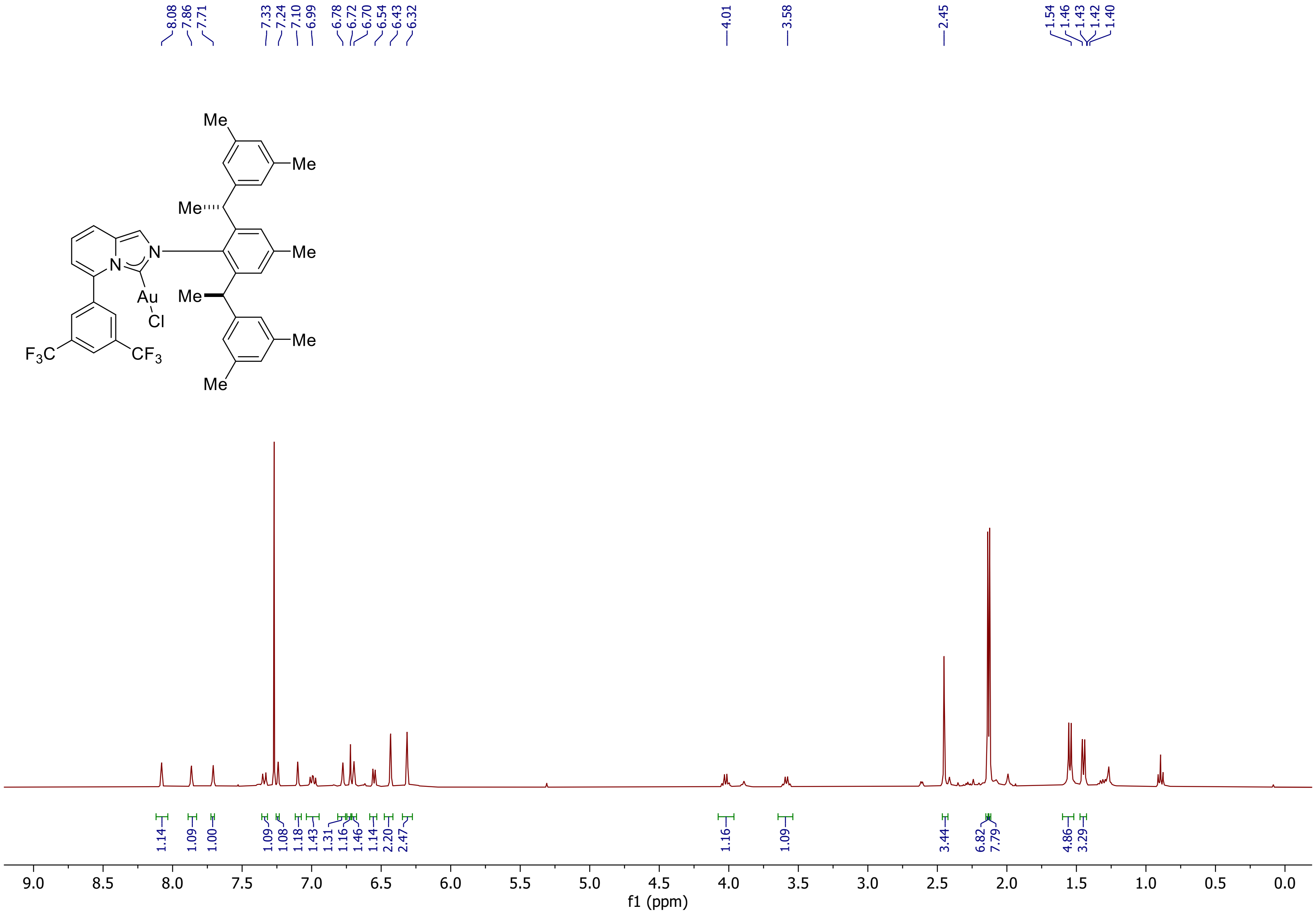

Figure S121. $^{13}C\{^{1}H\}$ NMR spectrum (101 MHz, 298 K, $CDCl_3$) of (*R,R*)-**6g**

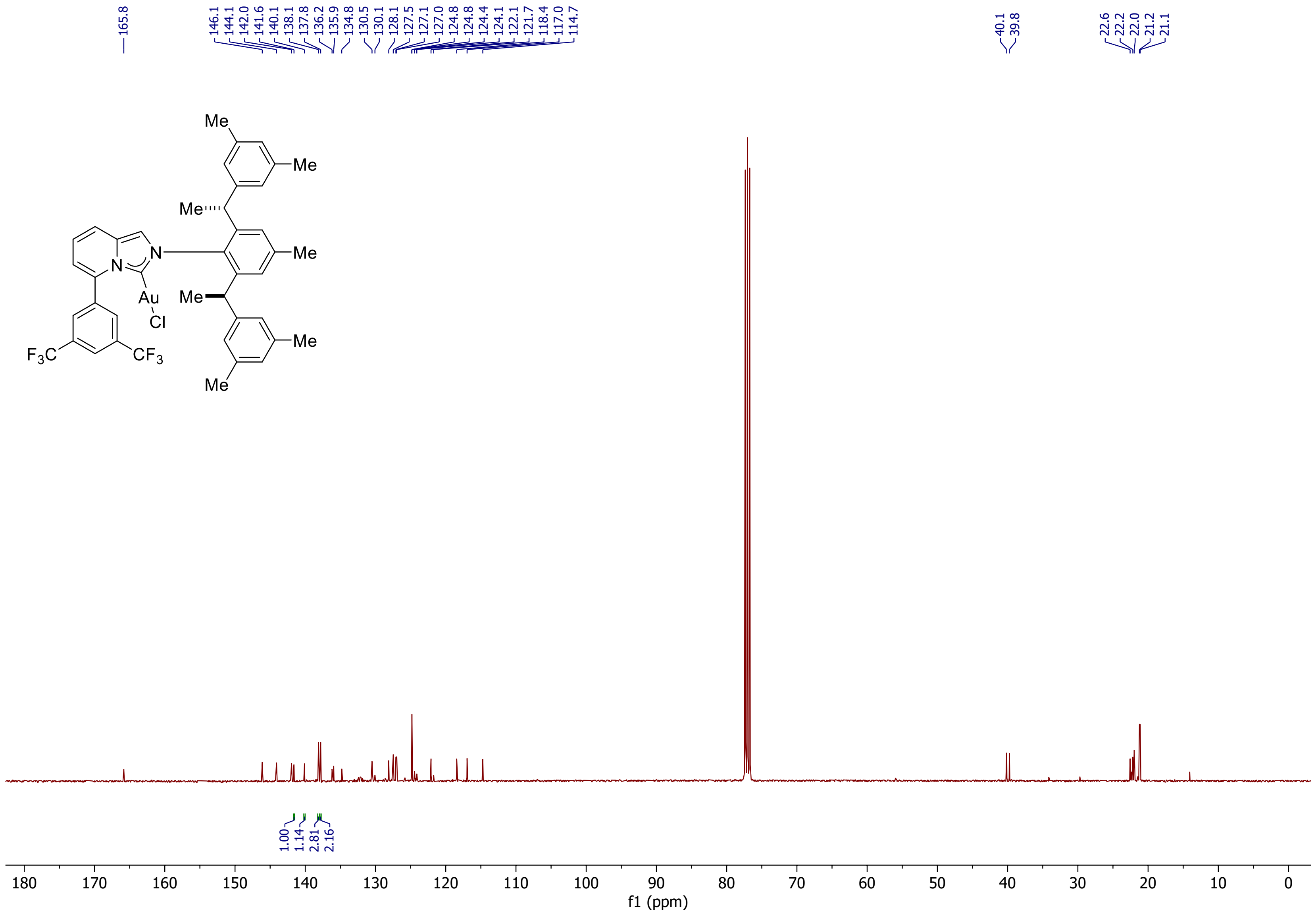

Figure S122. 2D ^1H-^1H COSY spectrum (298 K, $CDCl_3$) of (*R,R*)-**6g**

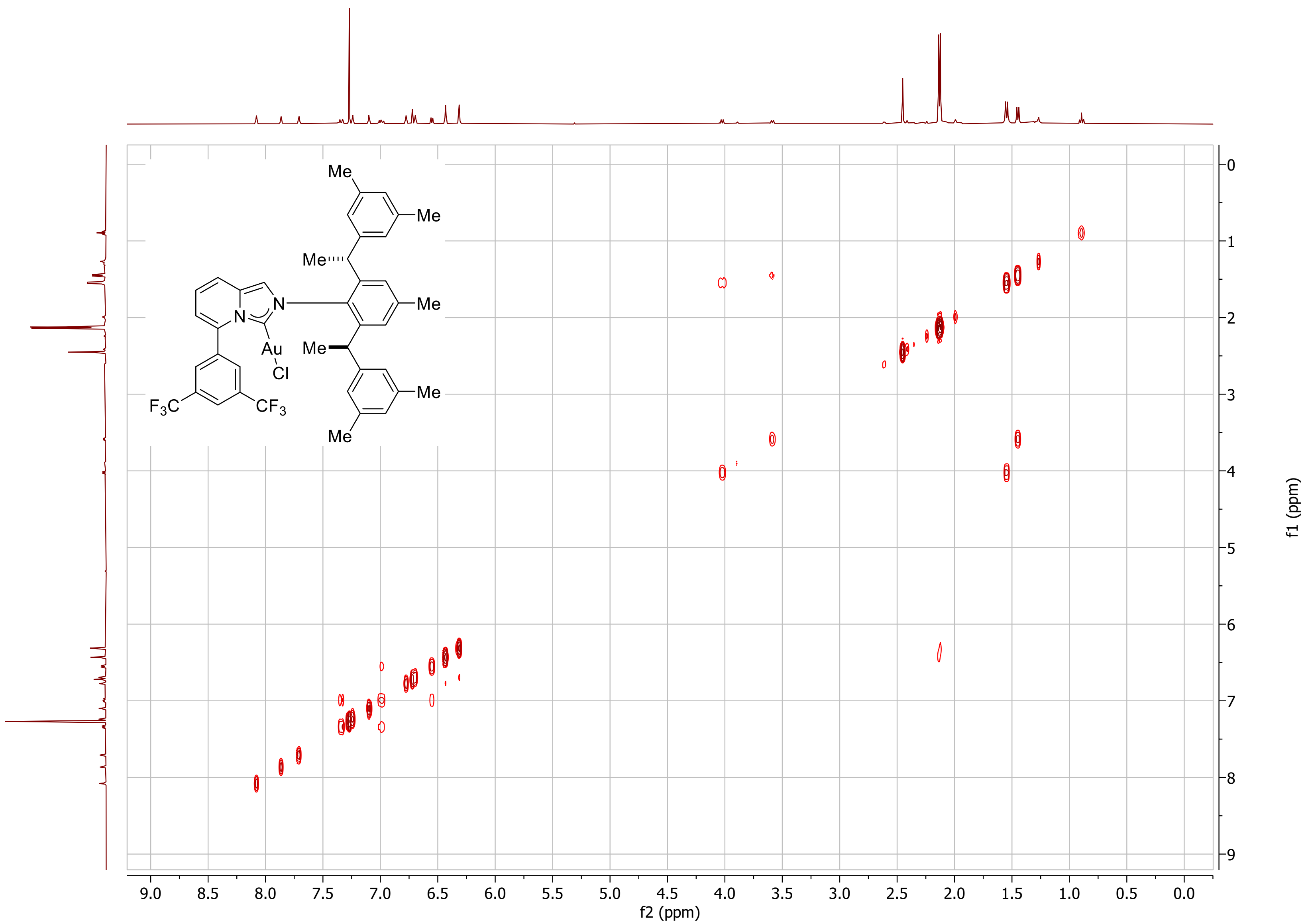

Figure S123. 2D ^{1}H-^{13}C HSQC spectrum (298 K, $CDCl_3$) of (*R,R*)-**6g**

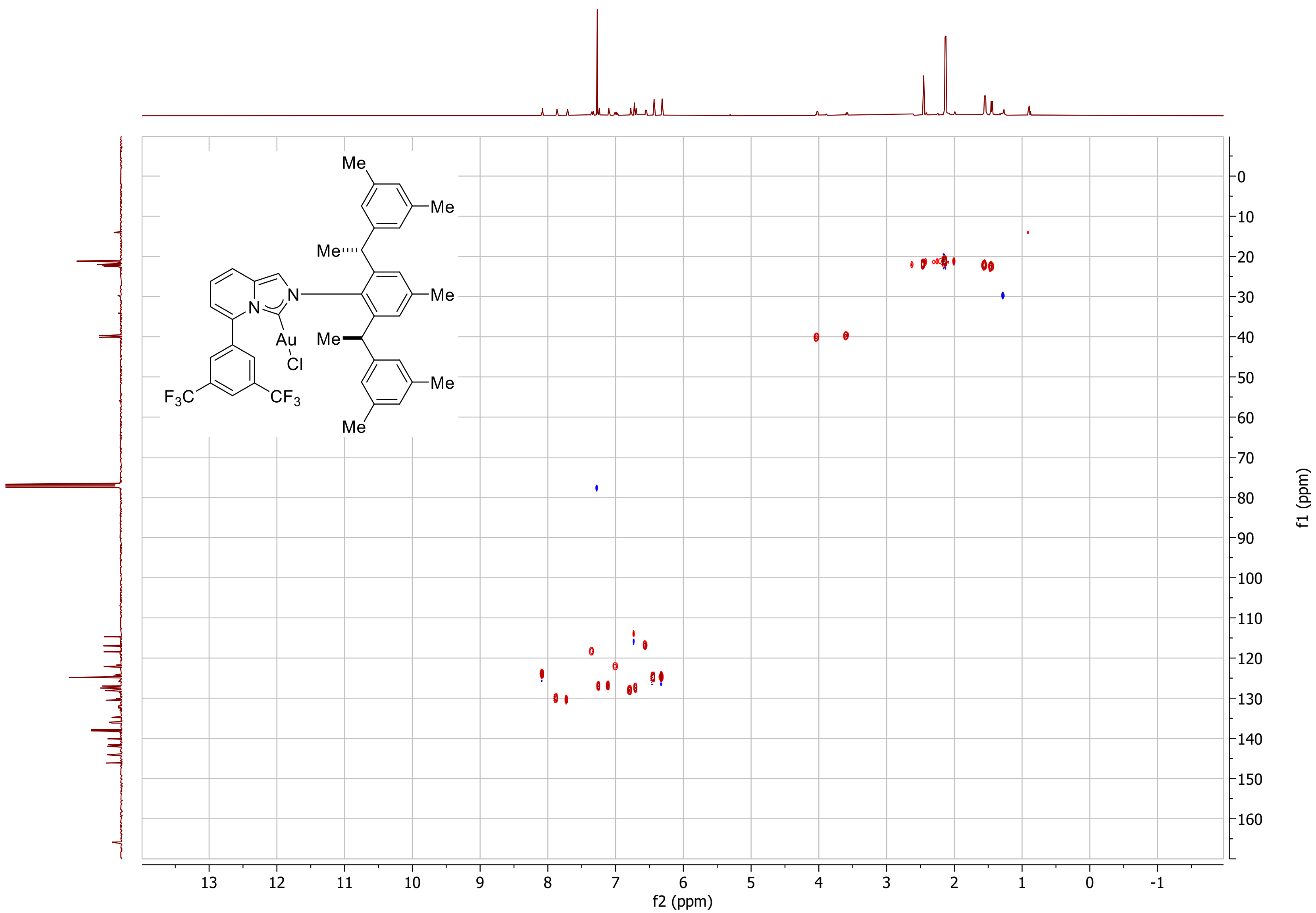

Figure S124. $^{19}F\{^1H\}$ NMR spectrum (282 MHz, 298 K, $CDCl_3$) of (*R*,*R*)-**6g**

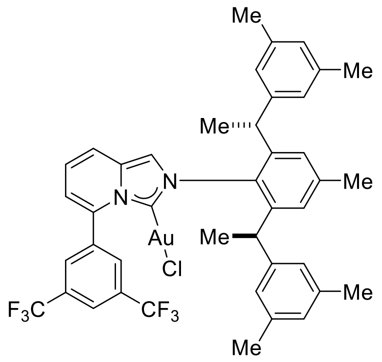

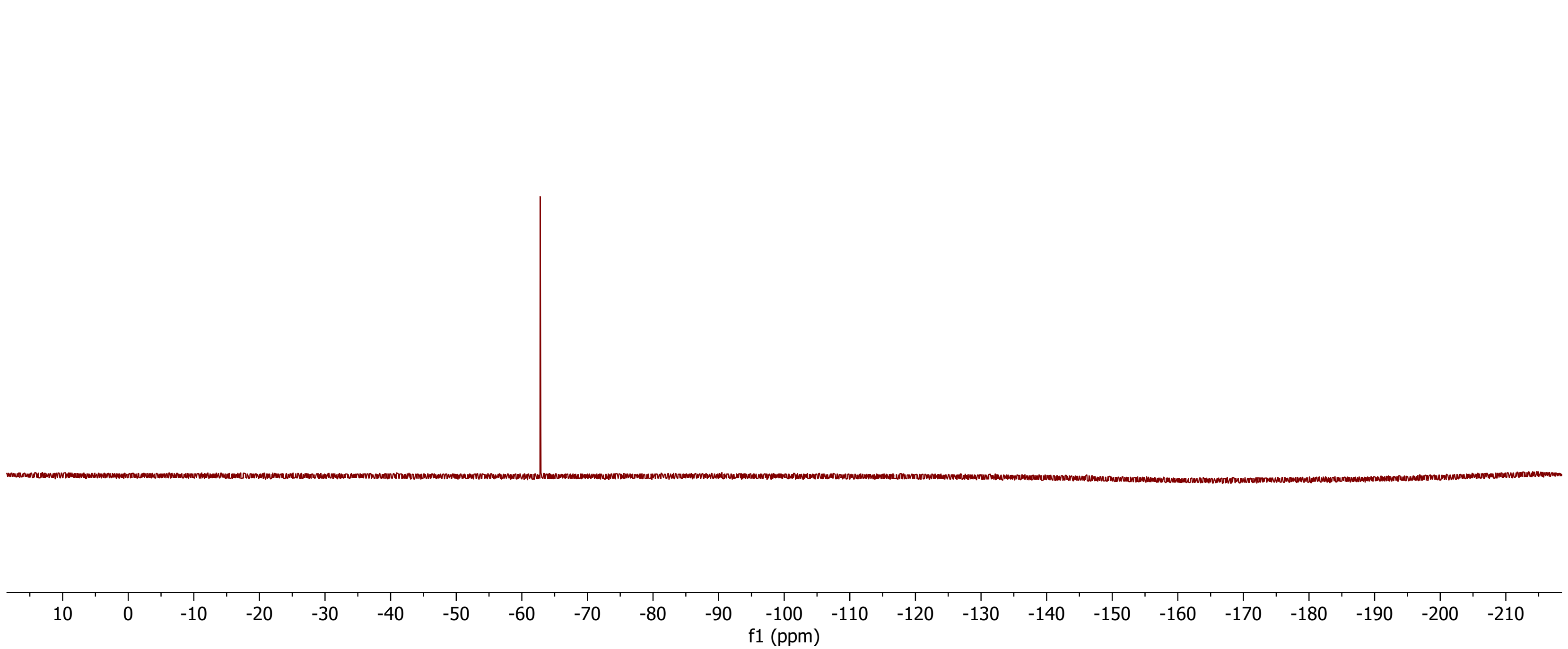

Figure S125. ^{1}H NMR spectrum (400 MHz, 298 K, $CDCl_3$) of (*R*,*R*)-**6h**

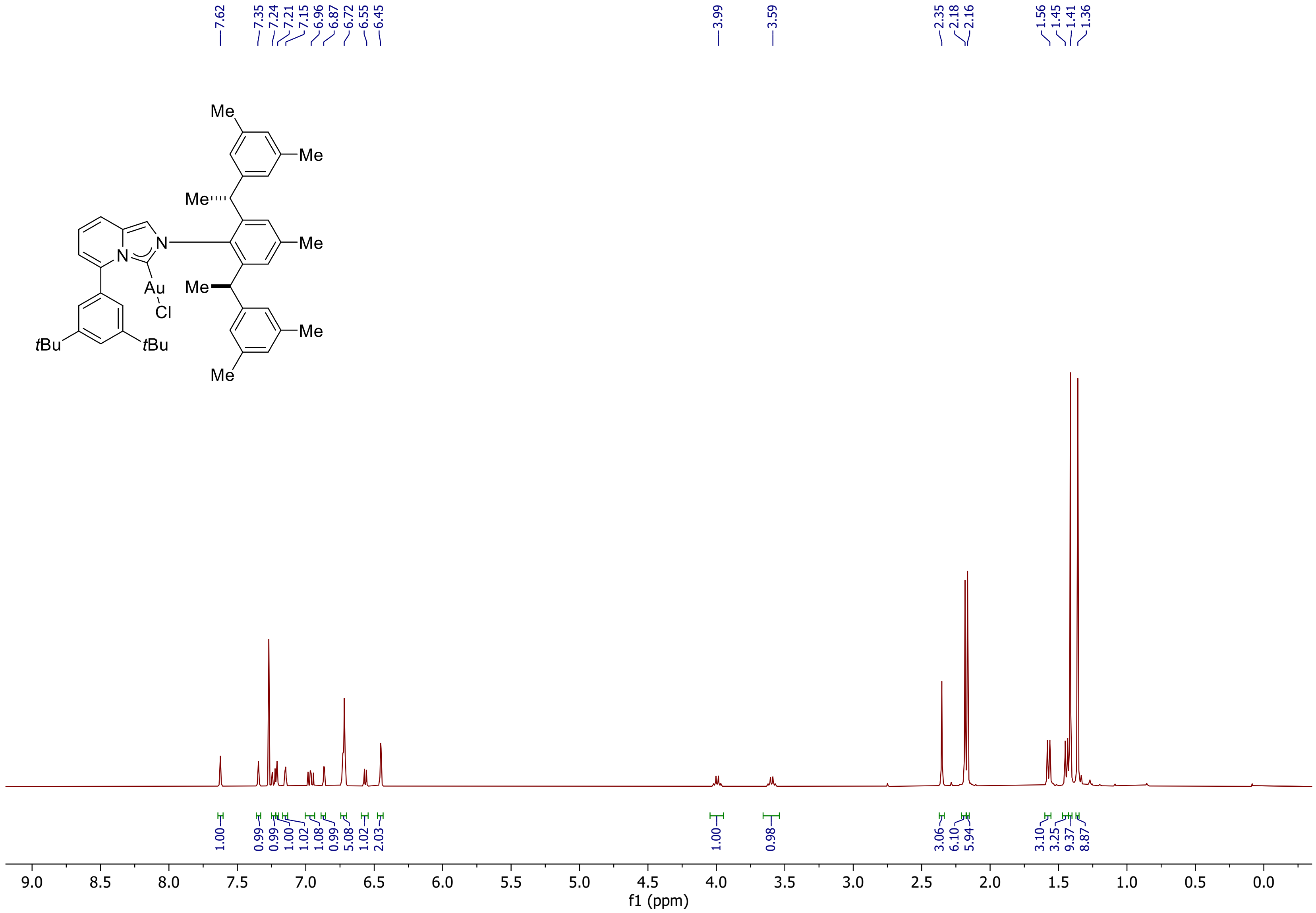

Figure S126. $^{13}C\{^{1}H\}$ NMR spectrum (101 MHz, 298 K, $CDCl_3$) of (*R,R*)-**6h**

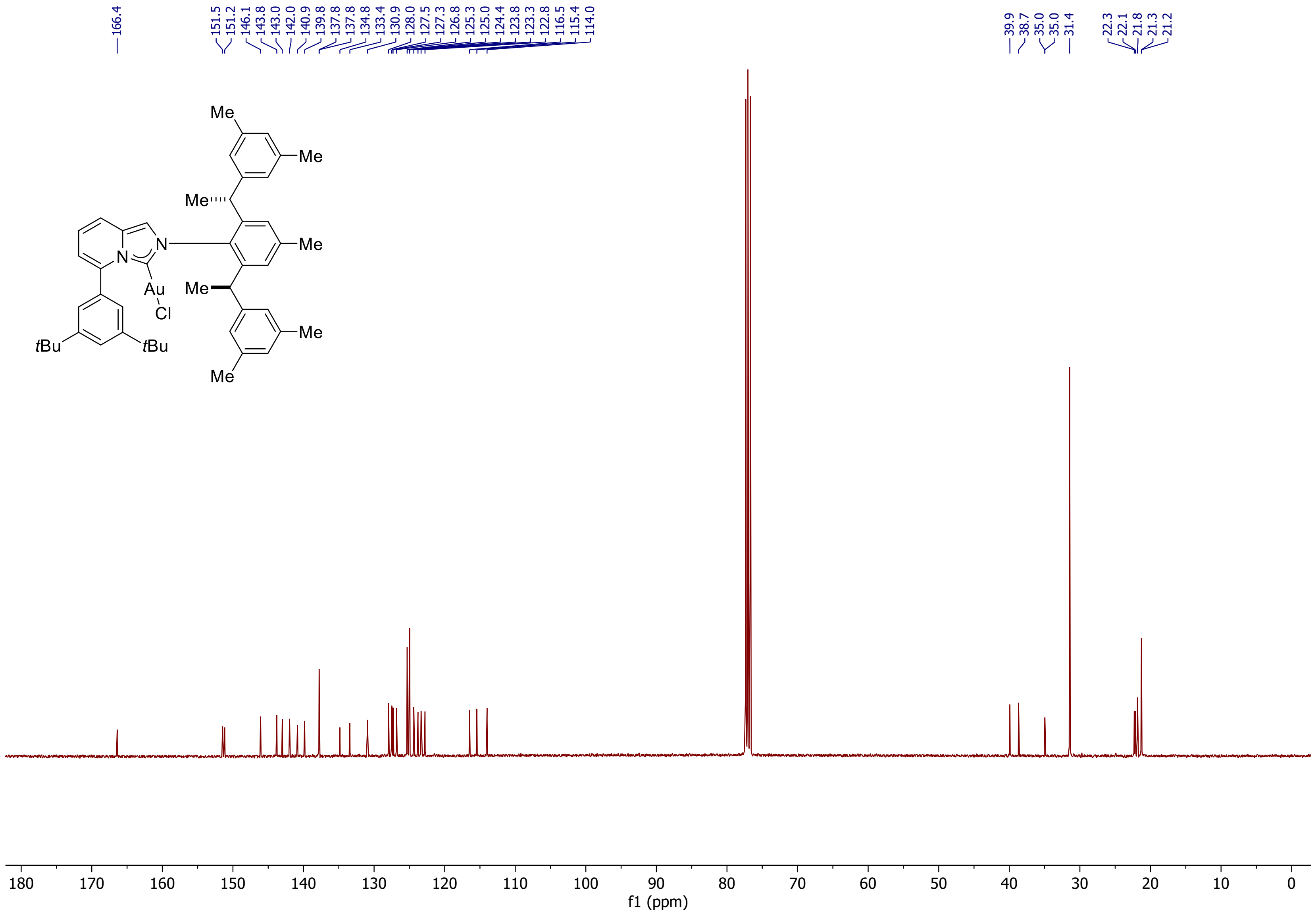

Figure S127. 2D ^{1}H-^{1}H COSY spectrum (298 K, $CDCl_3$) of (*R,R*)-**6h**

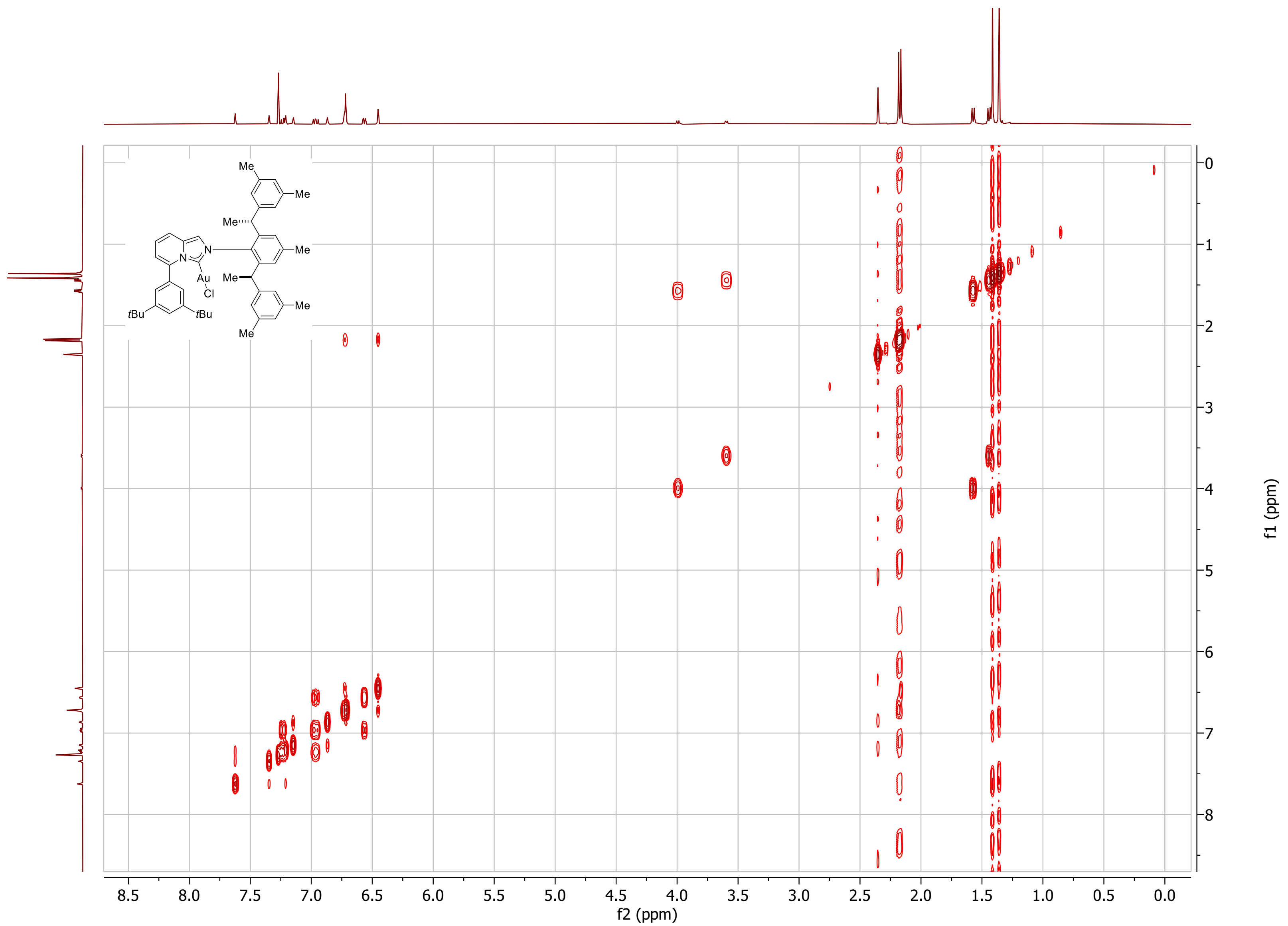

Figure S128. 2D ^{1}H-^{13}C HSQC spectrum (298 K, $CDCl_3$) of (*R,R*)-**6h**

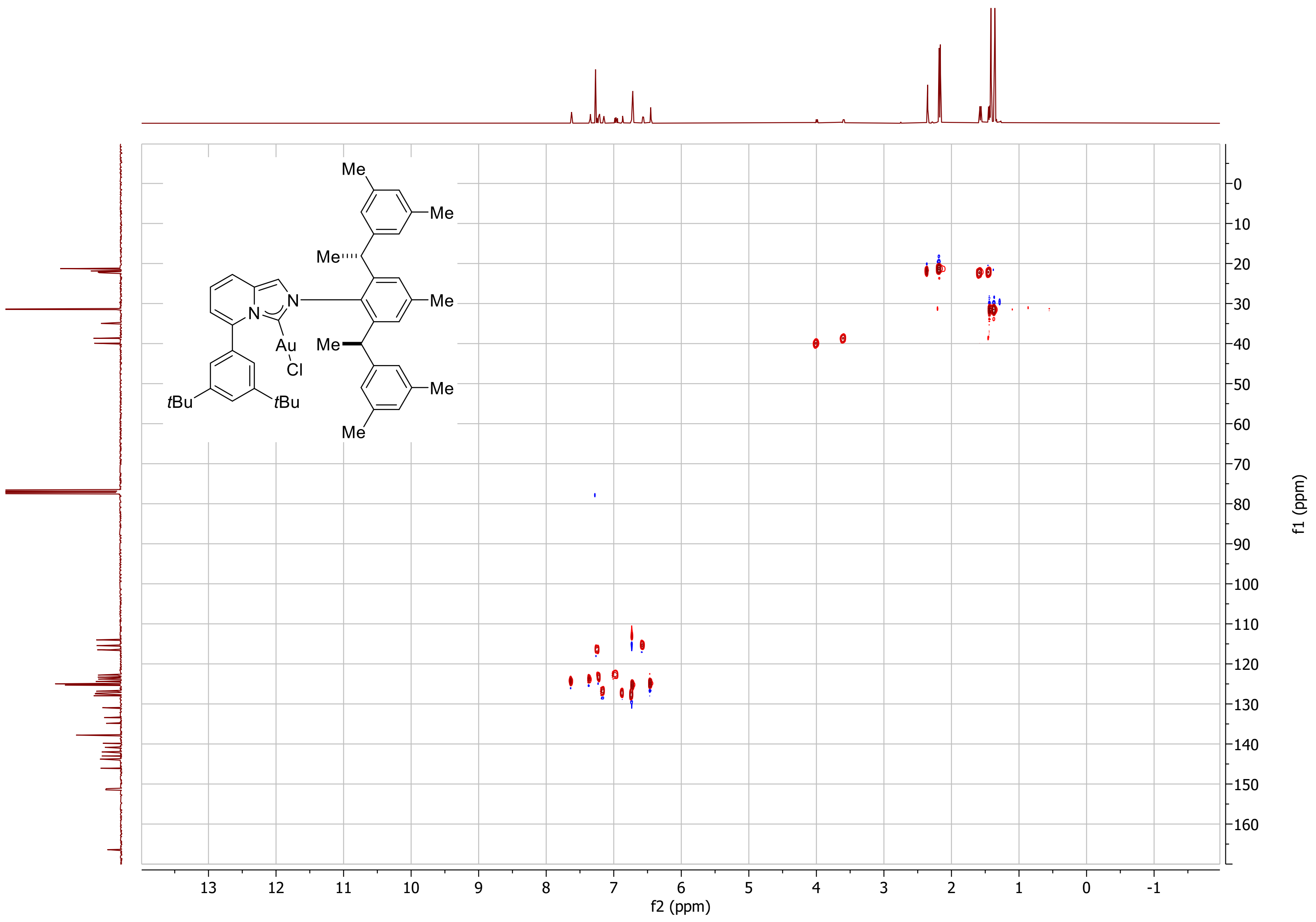

Figure S129. ^{1}H NMR spectrum (400 MHz, 298 K, $CDCl_3$) of (*R,R*)-**6i**

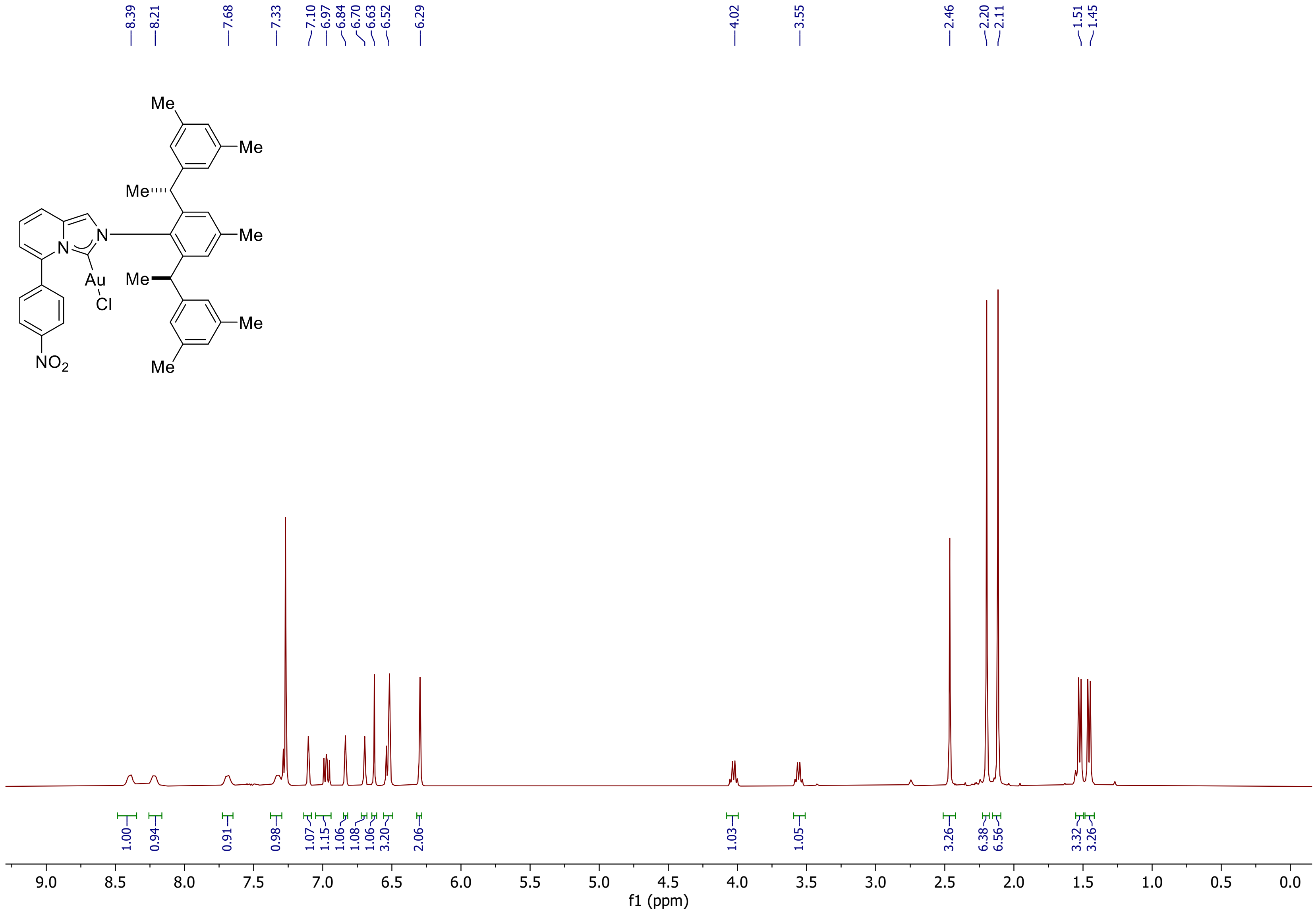

Figure S130. $^{13}C\{^{1}H\}$ NMR spectrum (101 MHz, 298 K, $CDCl_3$) of (*R,R*)-**6i**

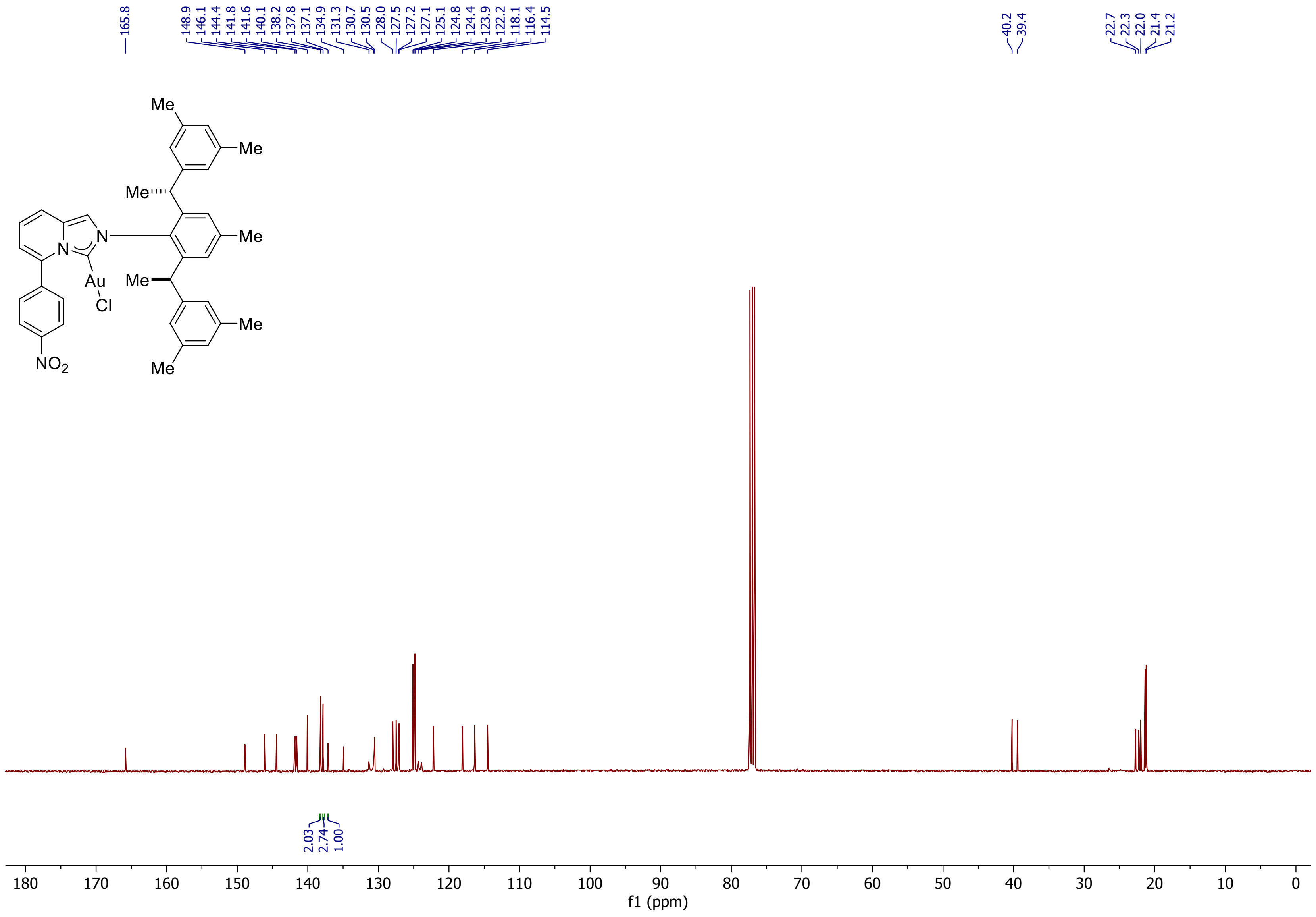

Figure S131. 2D ^1H-^1H COSY spectrum (298 K, $CDCl_3$) of (*R,R*)-**6i**

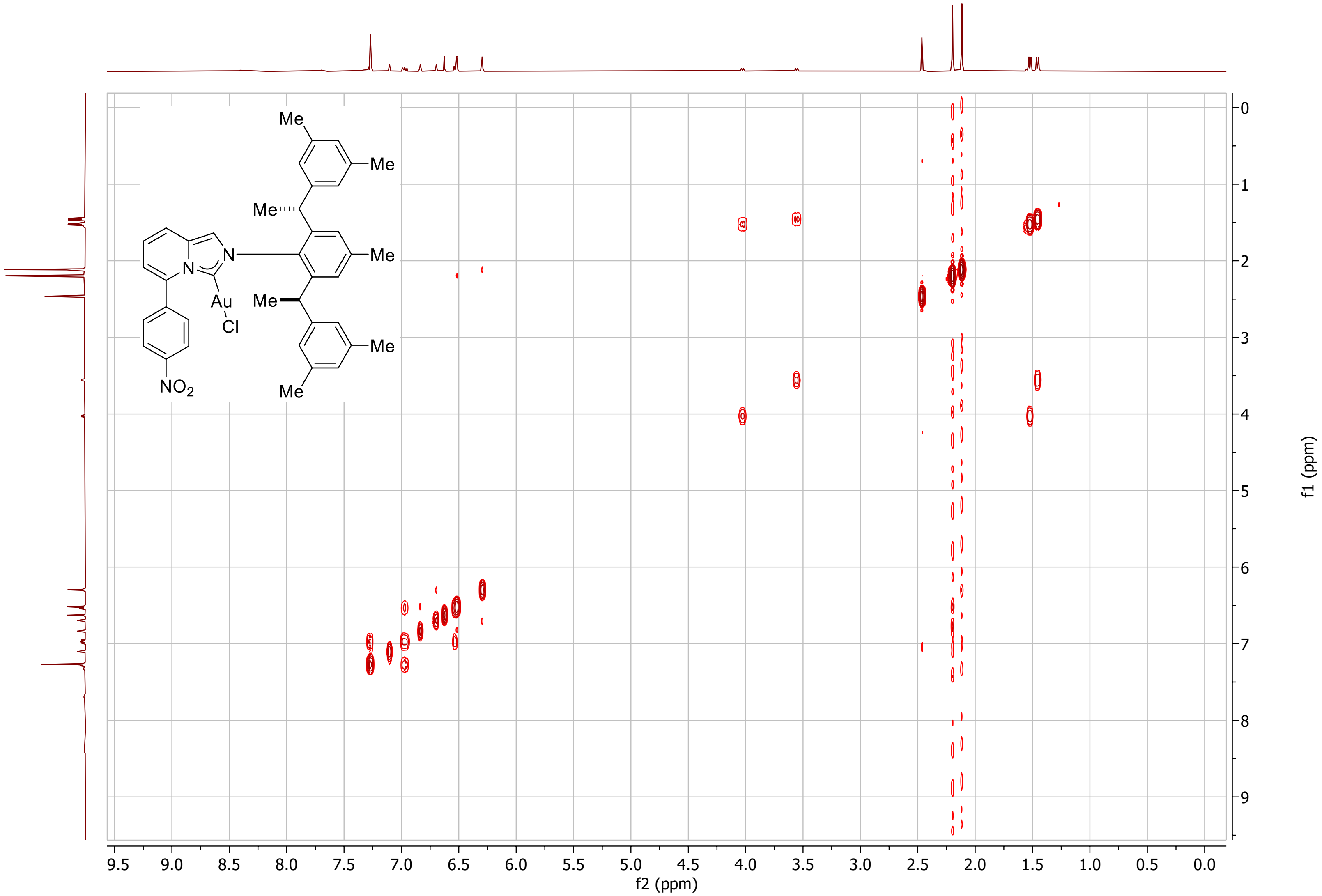

Figure S132. 2D ^{1}H-^{13}C HSQC spectrum (298 K, $CDCl_3$) of (*R,R*)-**6i**

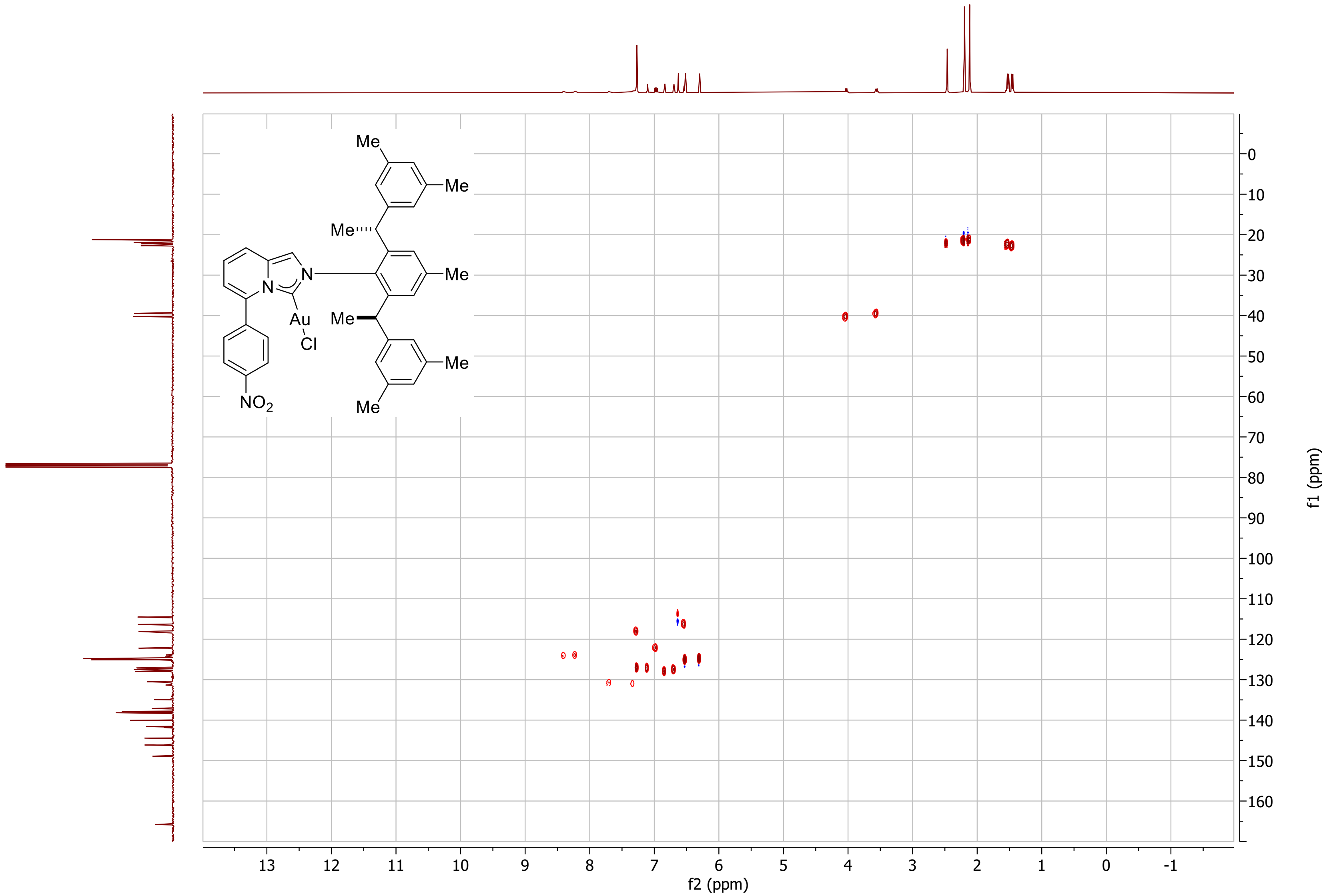

Figure S133. ^{1}H NMR spectrum (400 MHz, 298 K, $CDCl_3$) of (*R,R*)-**6j**

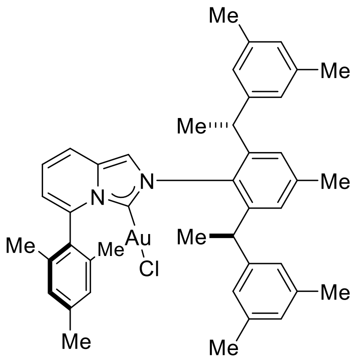

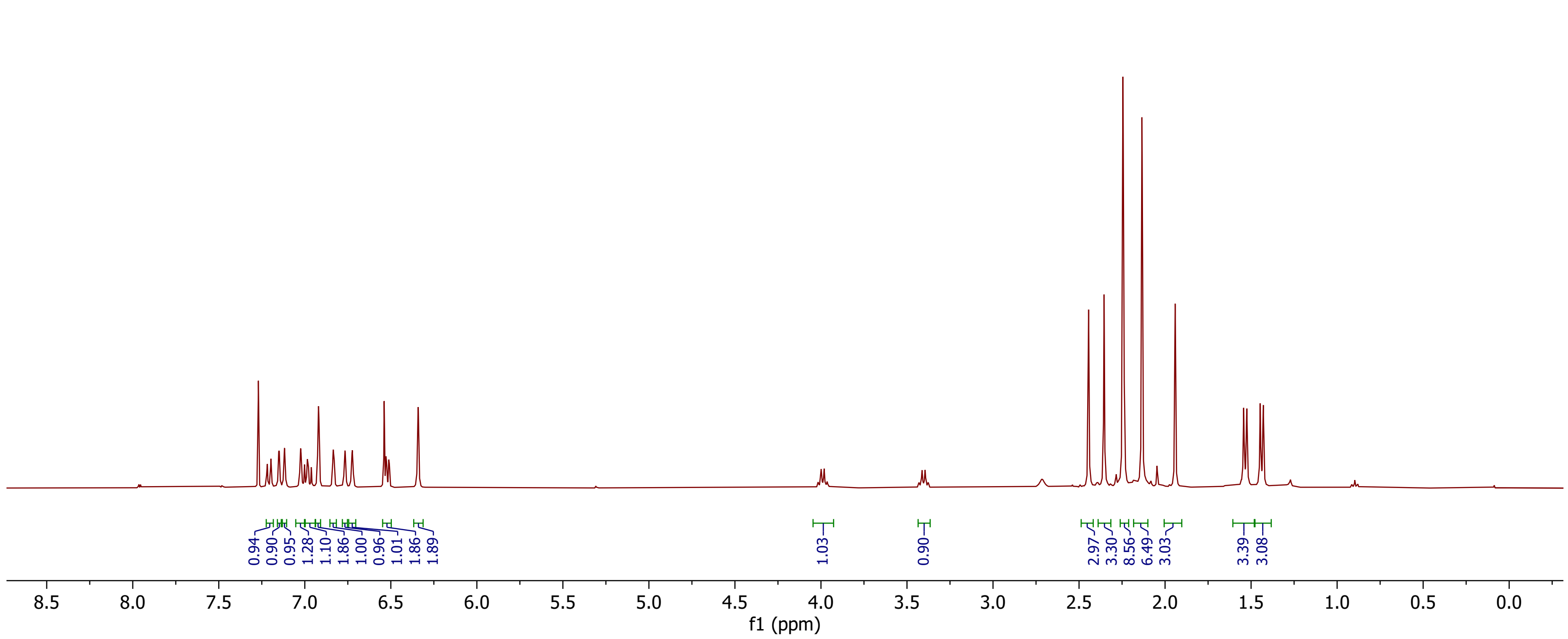

Figure S134. $^{13}C\{^{1}H\}$ NMR spectrum (101 MHz, 298 K, $CDCl_3$) of (*R,R*)-**6j**

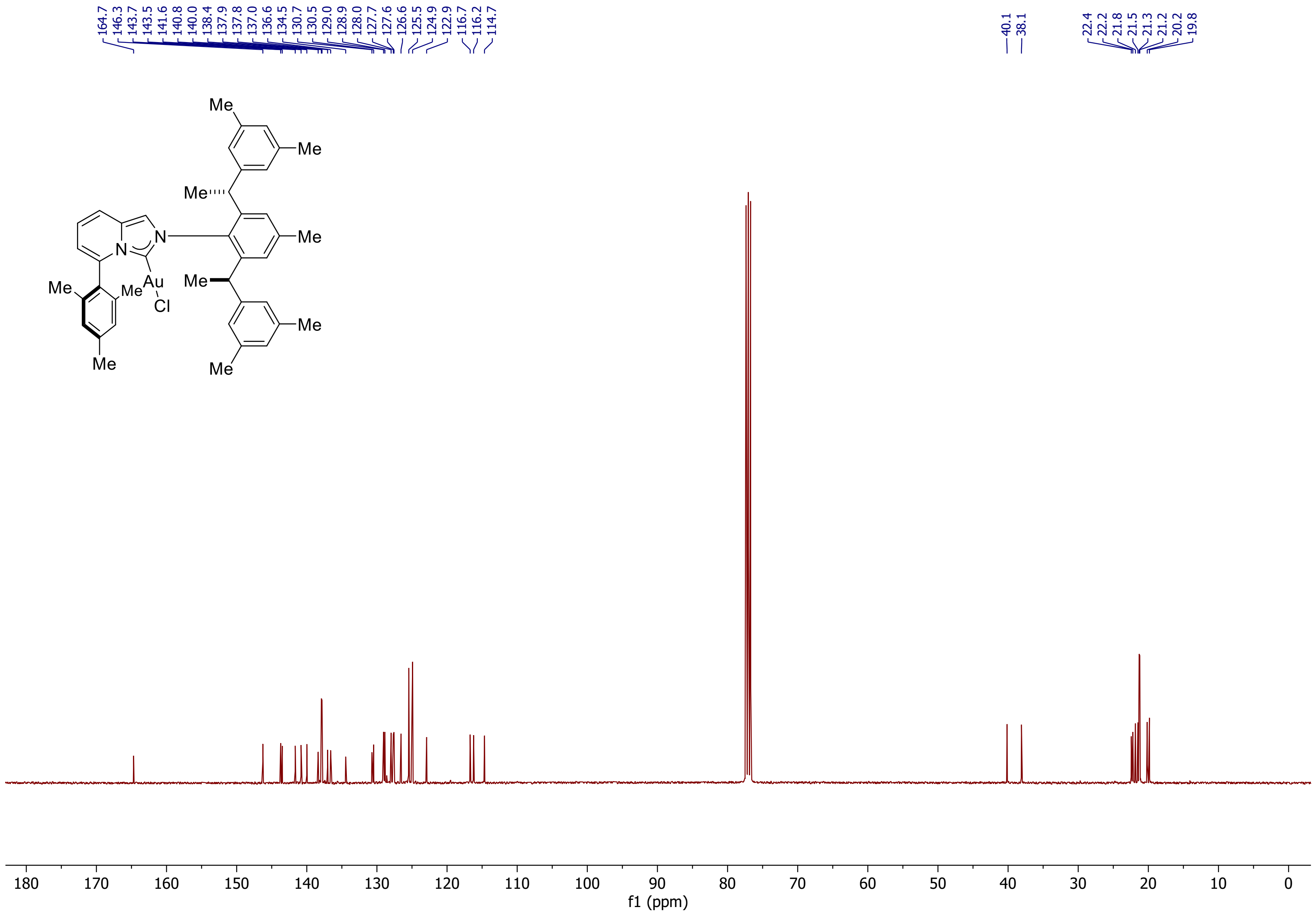

Figure S135. 2D ^{1}H-^{13}C HSQC spectrum (298 K, $CDCl_3$) of (*R*,*R*)-**6j**

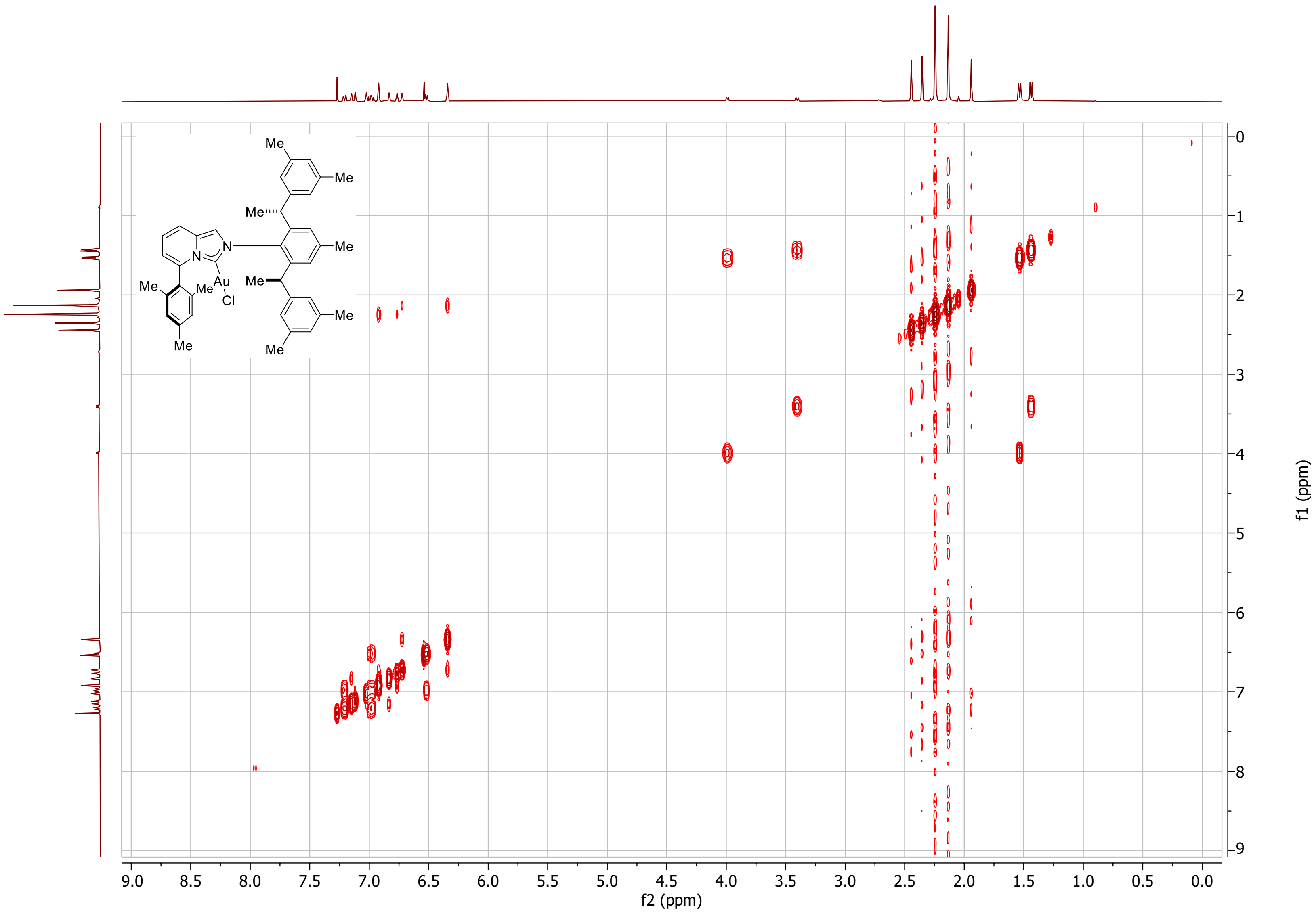

Figure S136. 2D ^1H-^{13}C HSQC spectrum (298 K, $CDCl_3$) of (*R,R*)-**6j**

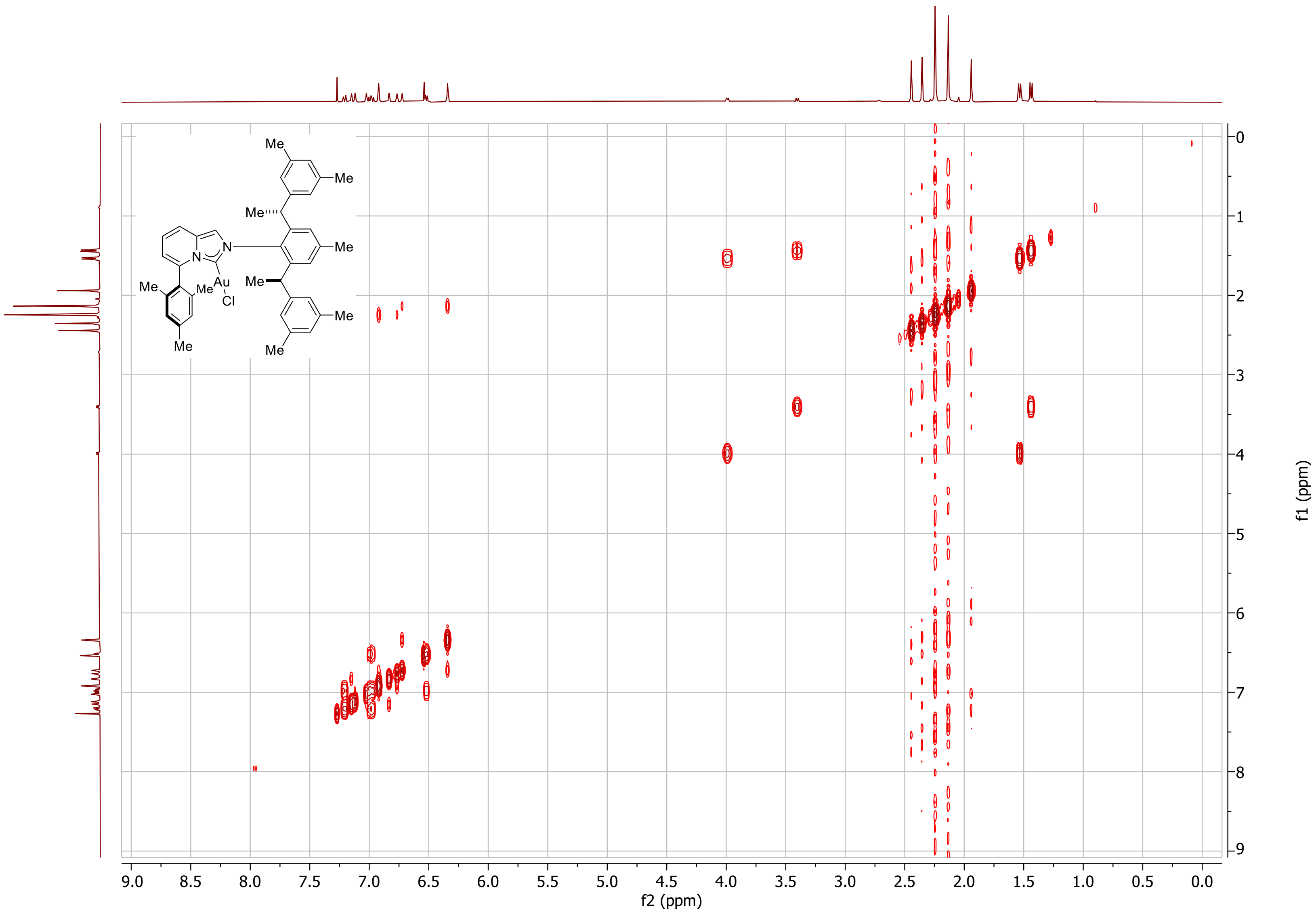

Figure S137. ^{1}H NMR spectrum (400 MHz, 298 K, $CDCl_3$)

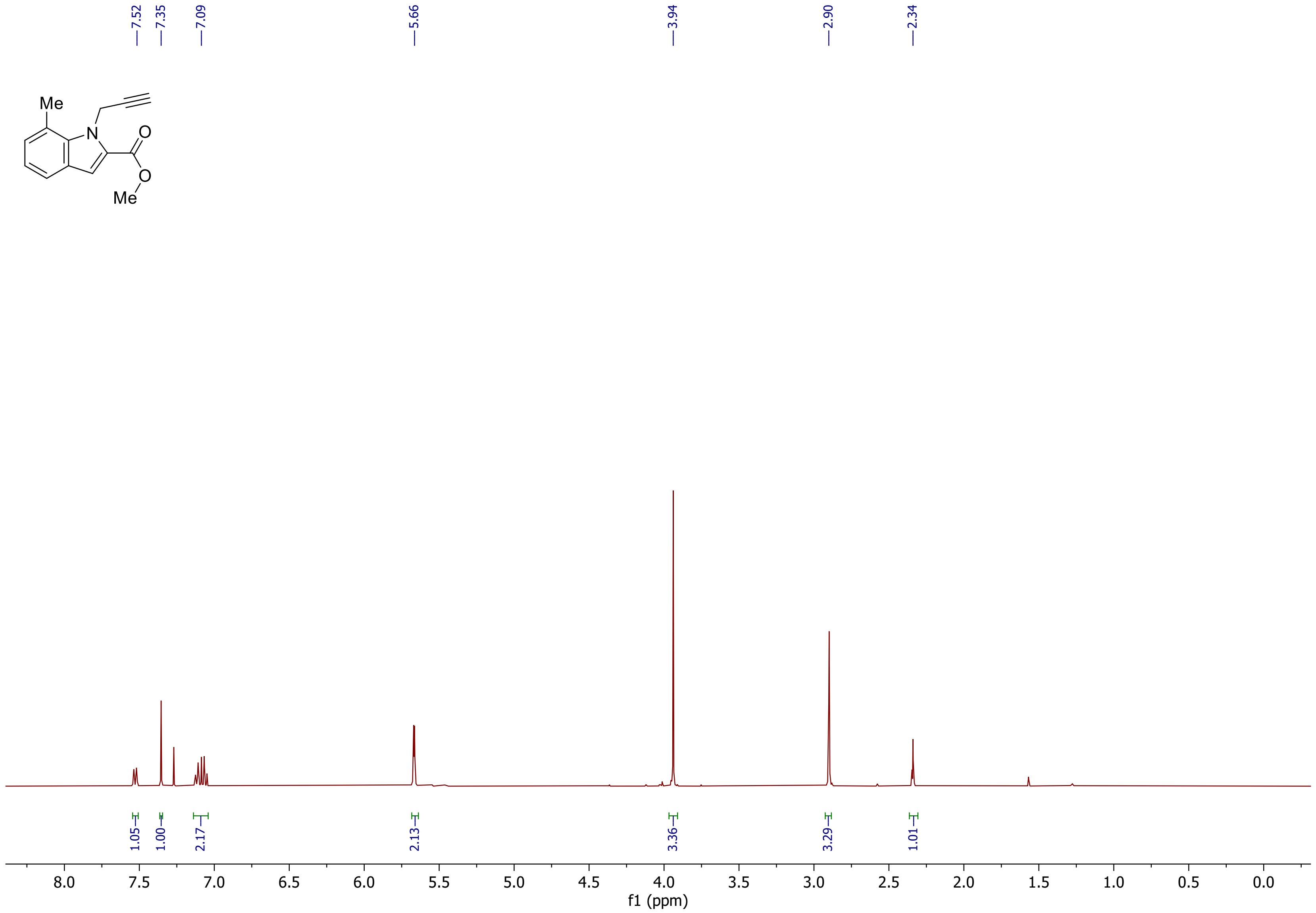

Figure S138. $^{13}C\{^{1}H\}$ NMR spectrum (101 MHz, 298 K, $CDCl_3$)

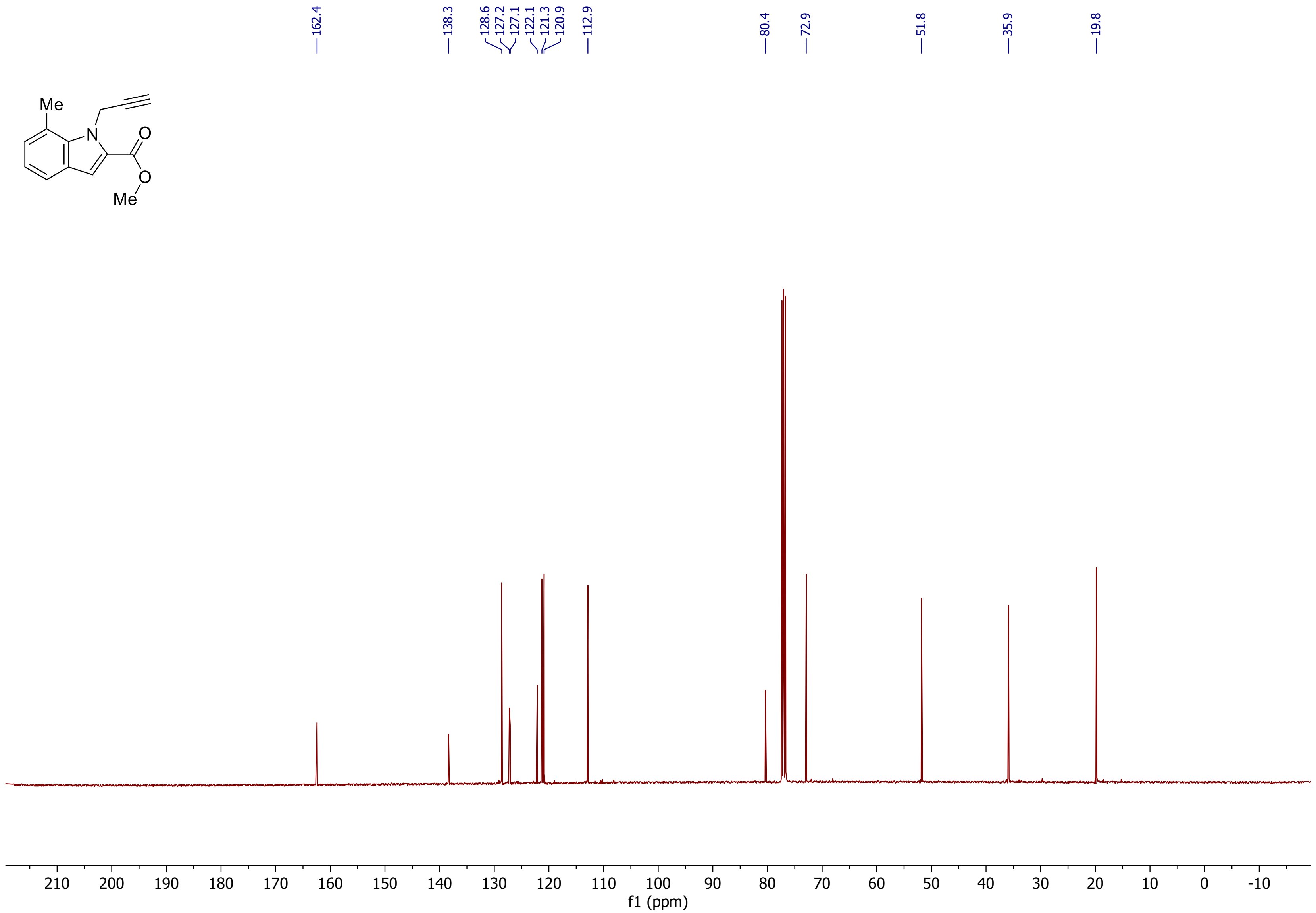

Figure S139. 2D ^1H-^1H COSY spectrum (298 K, $CDCl_3$)

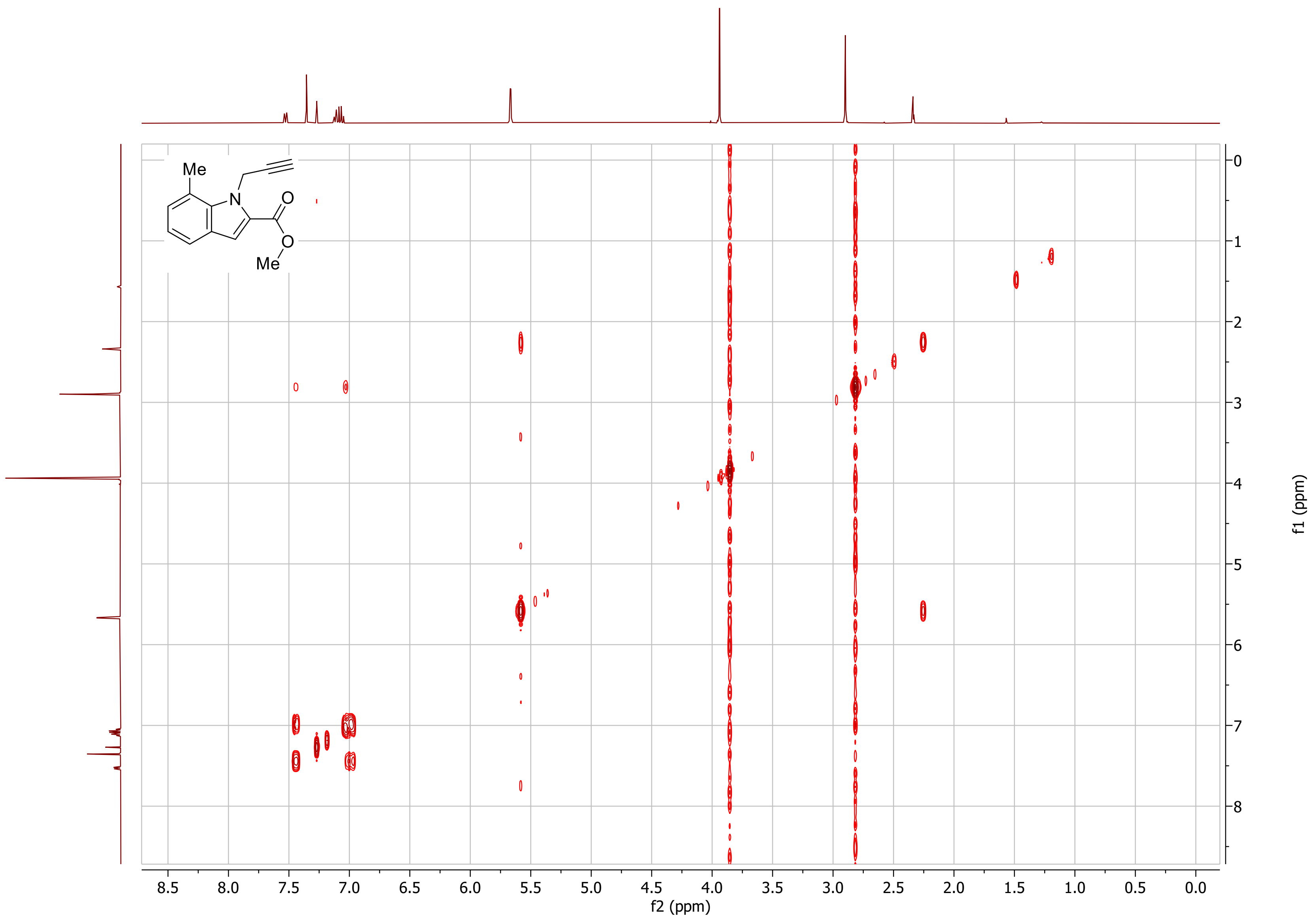

Figure S140. 2D ^{1}H-^{13}C HSQC spectrum (298 K, $CDCl_3$)

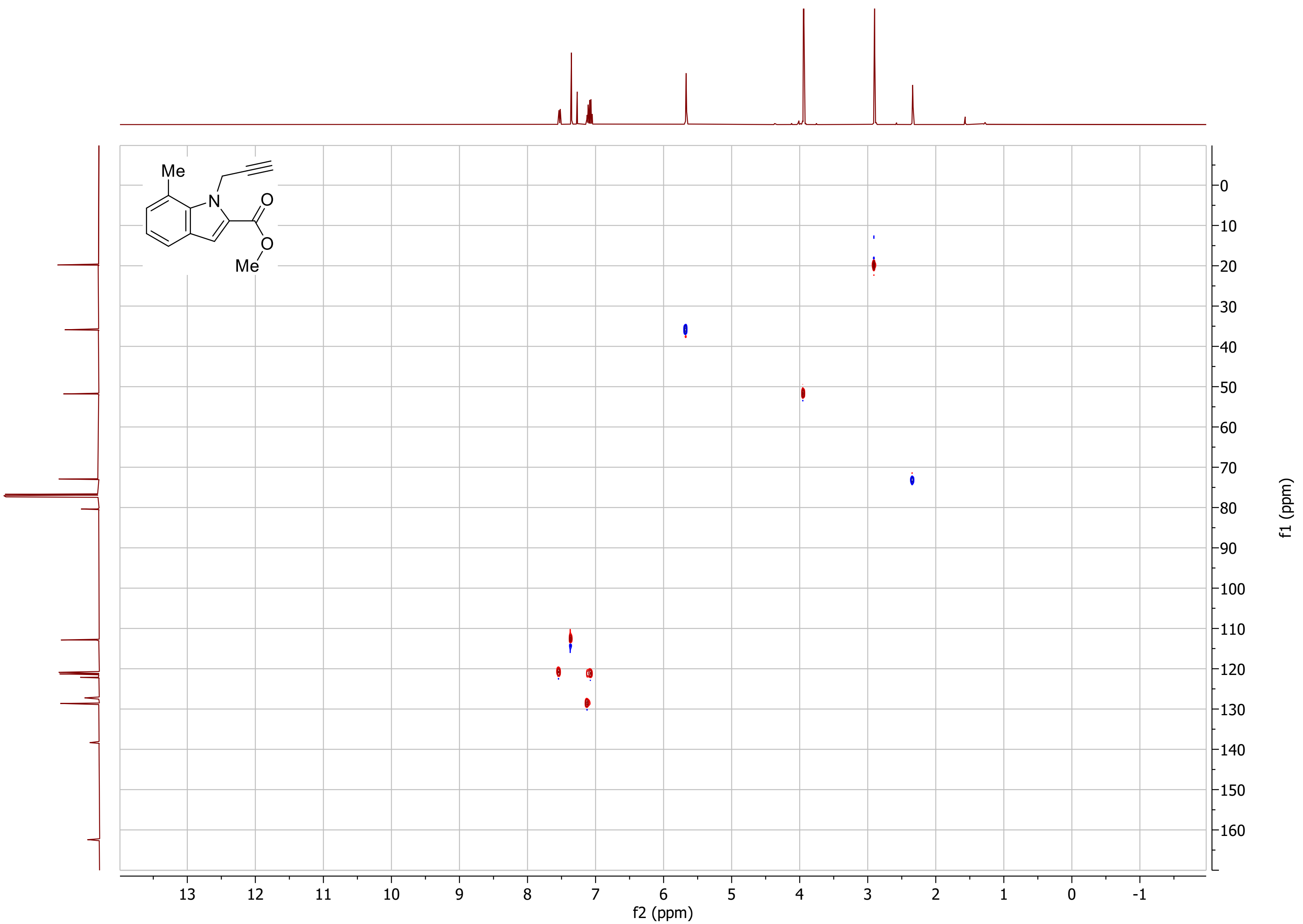

Figure S141. ^{1}H NMR spectrum (400 MHz, 298 K, $CDCl_3$)

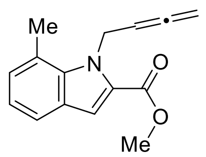

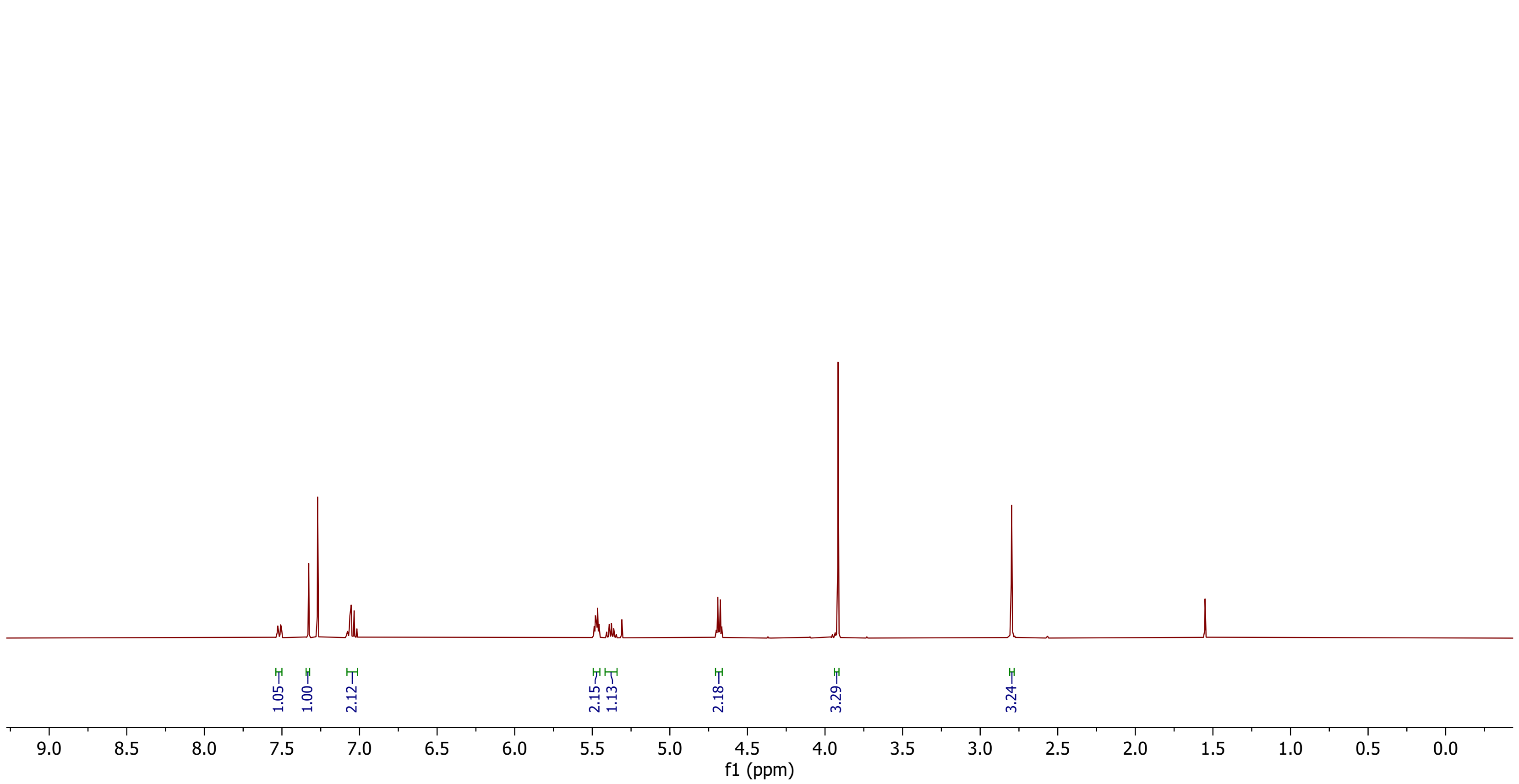

Figure S142. $^{13}C\{^{1}H\}$ NMR spectrum (101 MHz, 298 K, $CDCl_3$)

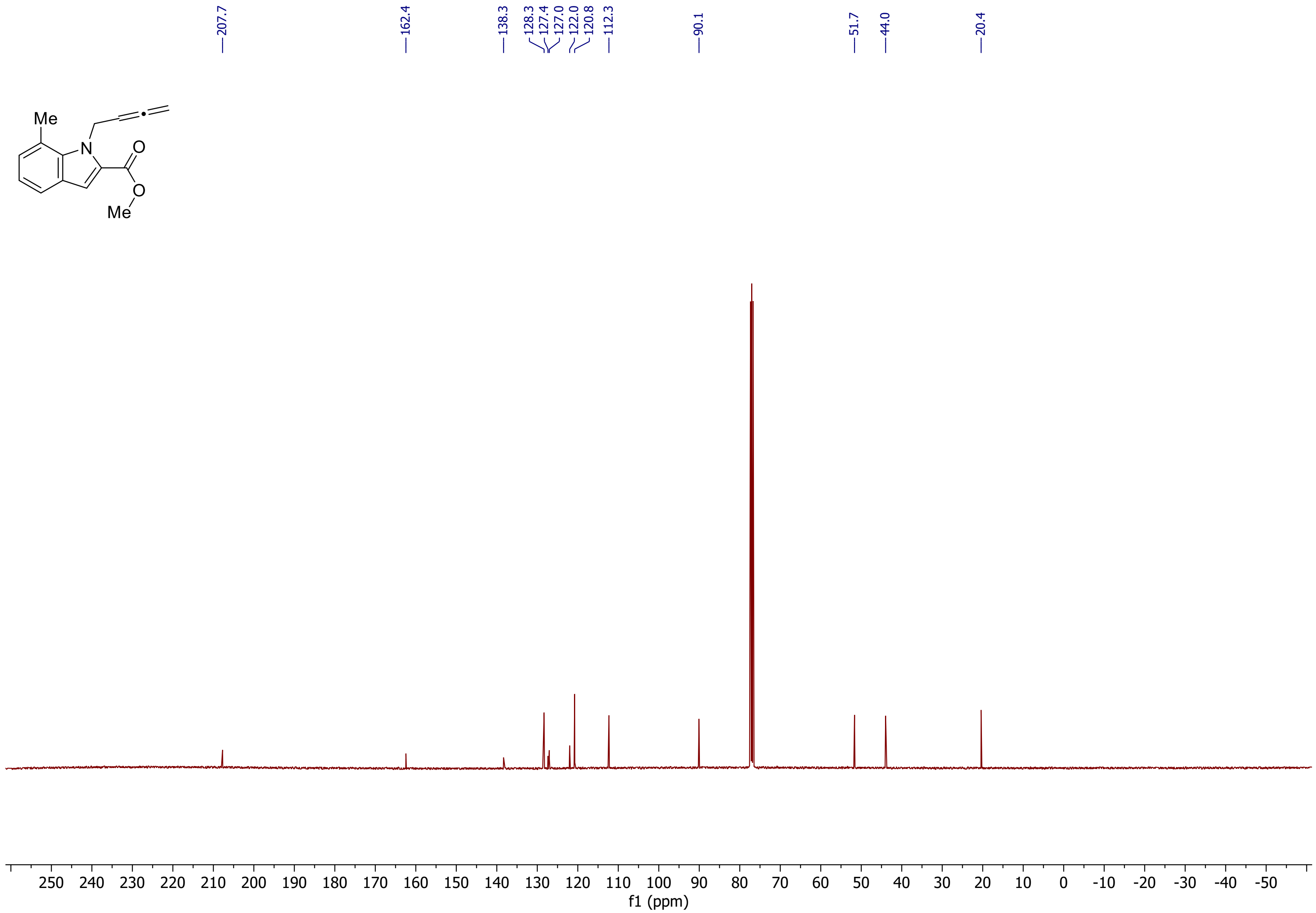

Figure S143. 2D ^{1}H-^{1}H COSY spectrum (298 K, $CDCl_3$)

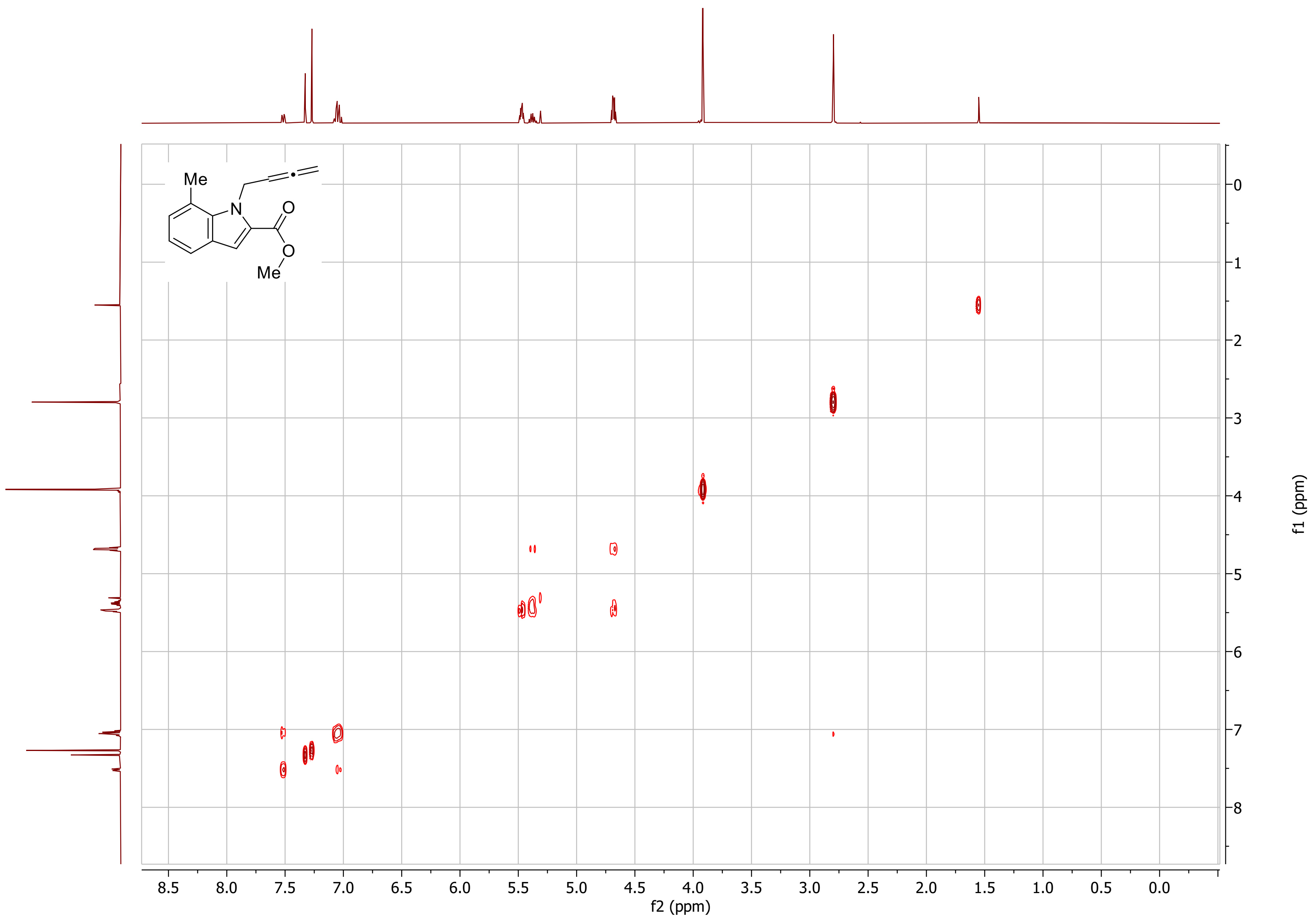

Figure S144. 2D ^1H-^{13}C HSQC spectrum (298 K, $CDCl_3$)

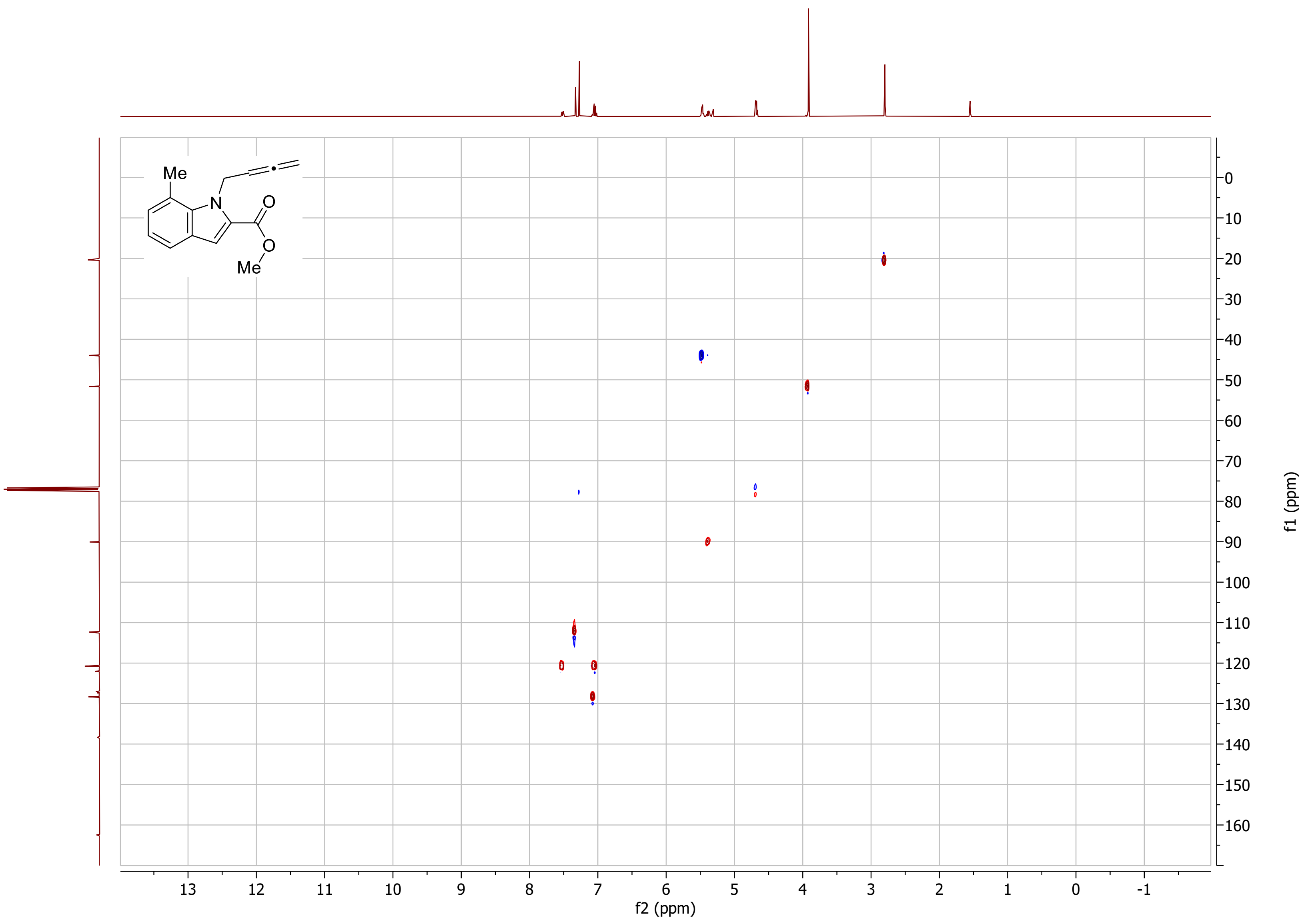

Figure S145. ^{1}H NMR spectrum (400 MHz, 298 K, $CDCl_3$) of **7e**

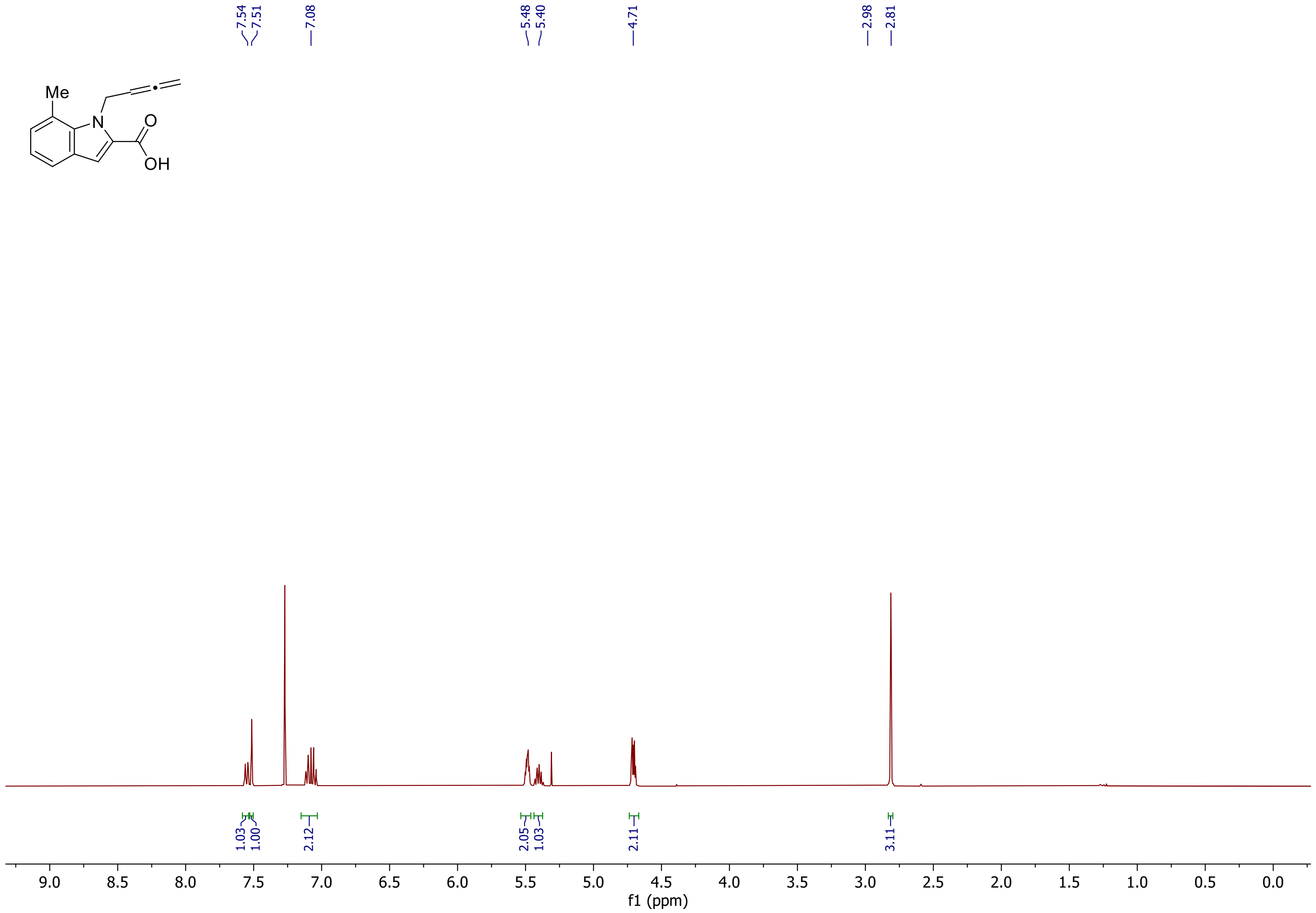

Figure S146. $^{13}C\{^{1}H\}$ NMR spectrum (101 MHz, 298 K, $CDCl_3$) of **7e**

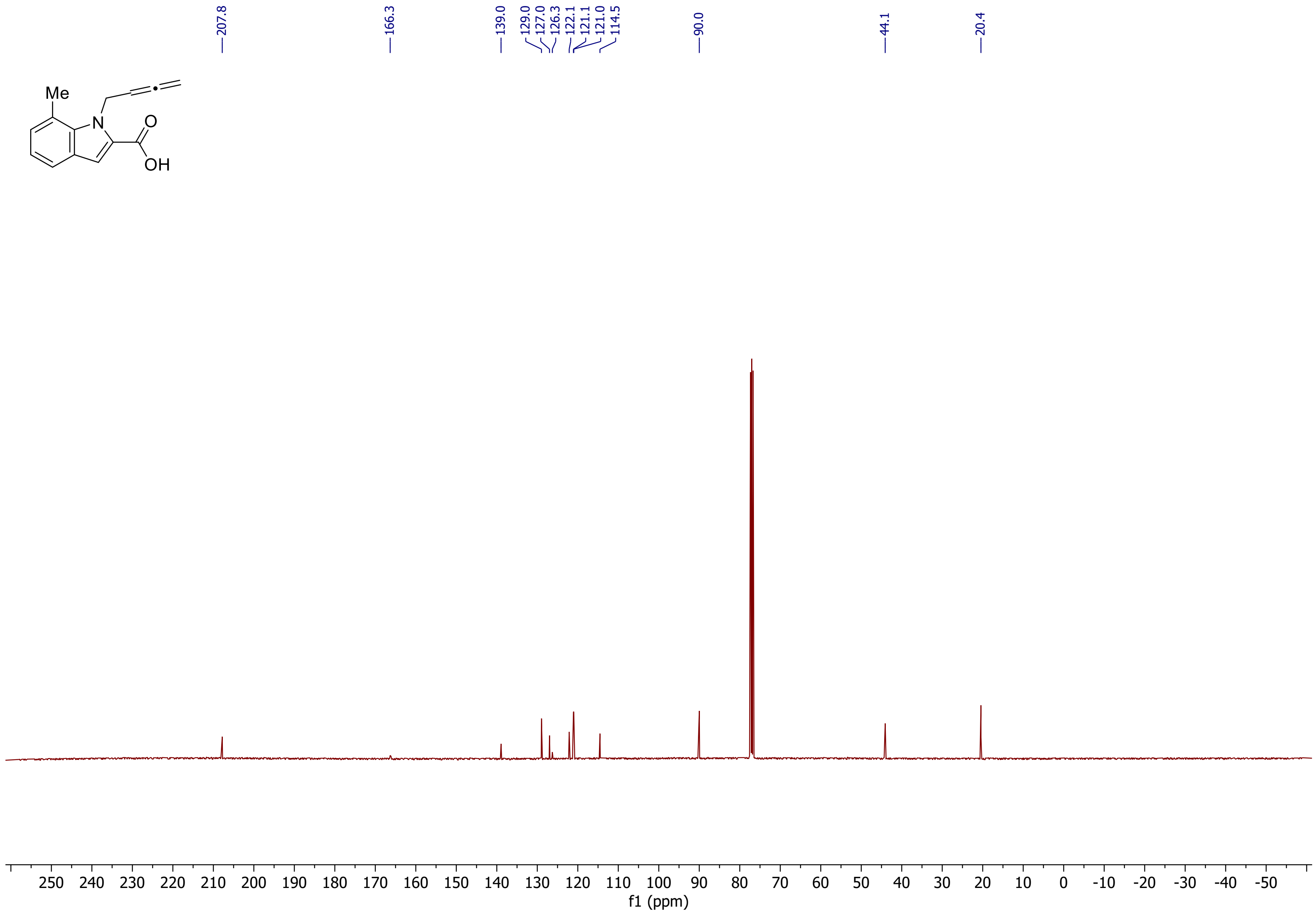

Figure S147. 2D ^{1}H-^{1}H COSY spectrum (298 K, $CDCl_3$) of **7e**

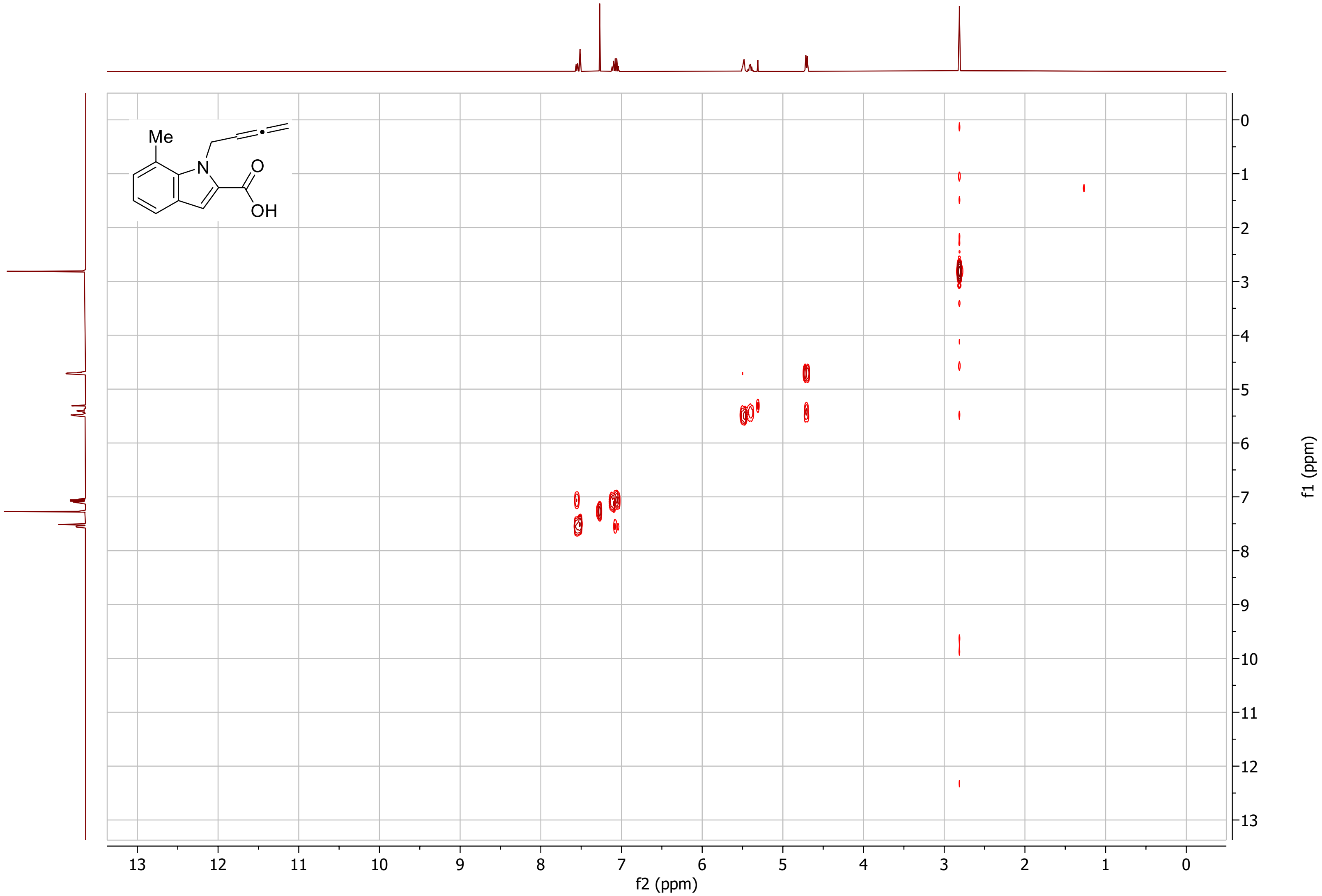

Figure S148. 2D ^{1}H-^{13}C HSQC spectrum (298 K, $CDCl_3$) of **7e**

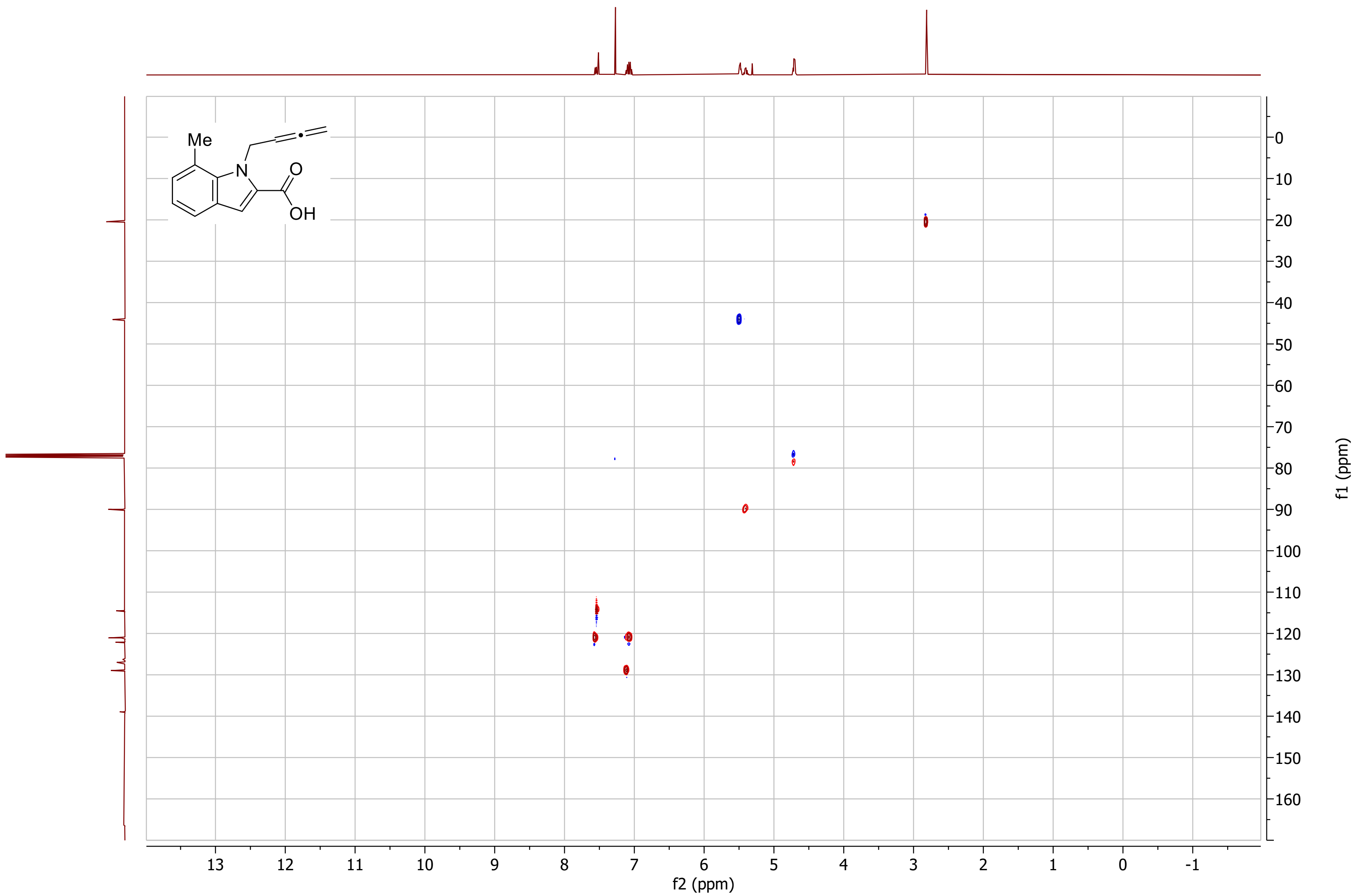

Figure S149. ^{1}H NMR spectrum (400 MHz, 298 K, $CDCl_3$) of (*R*)-**8e**

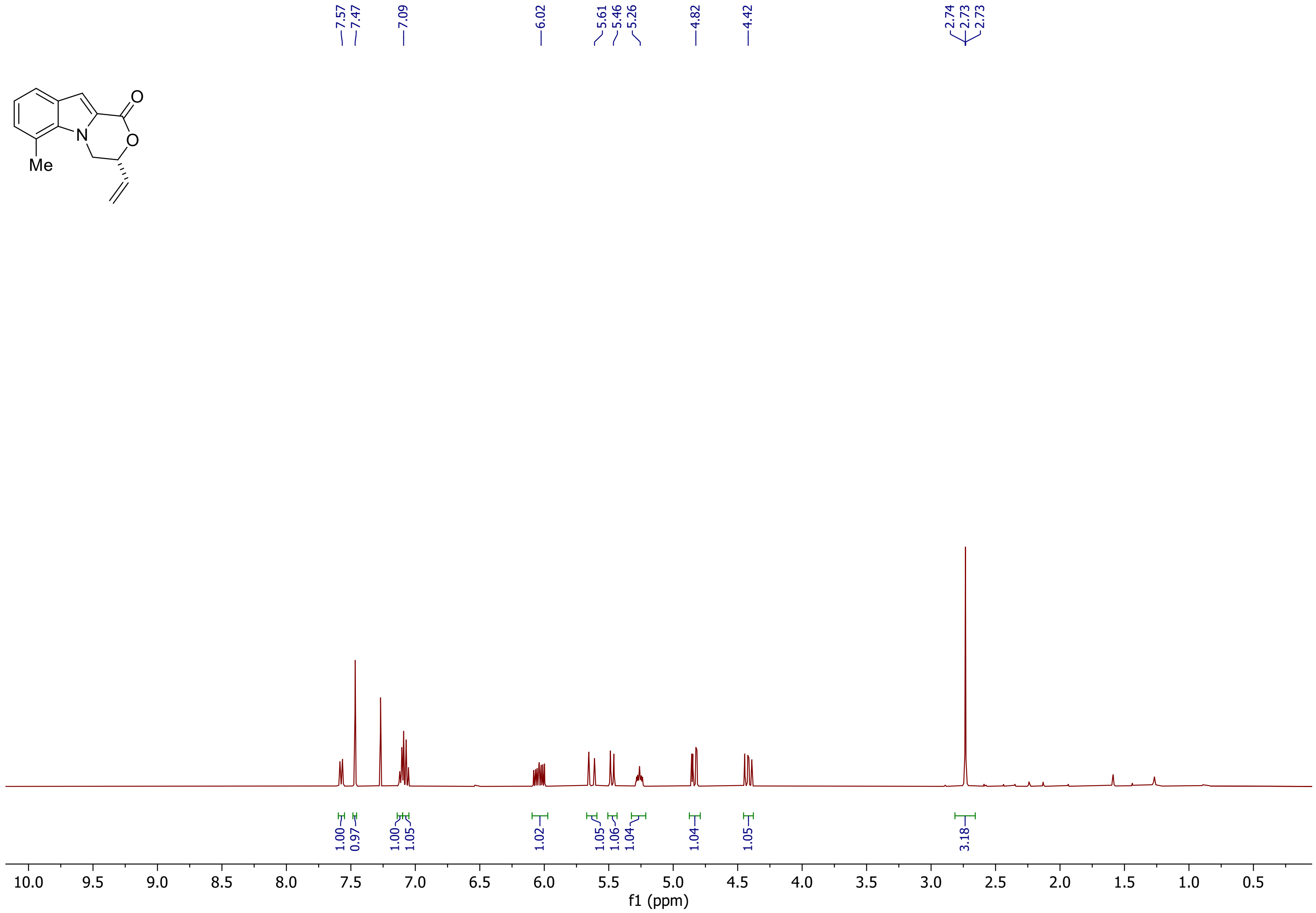

Figure S150. $^{13}C\{^{1}H\}$ NMR spectrum (101 MHz, 298 K, $CDCl_3$) of (*R*)-**8e**

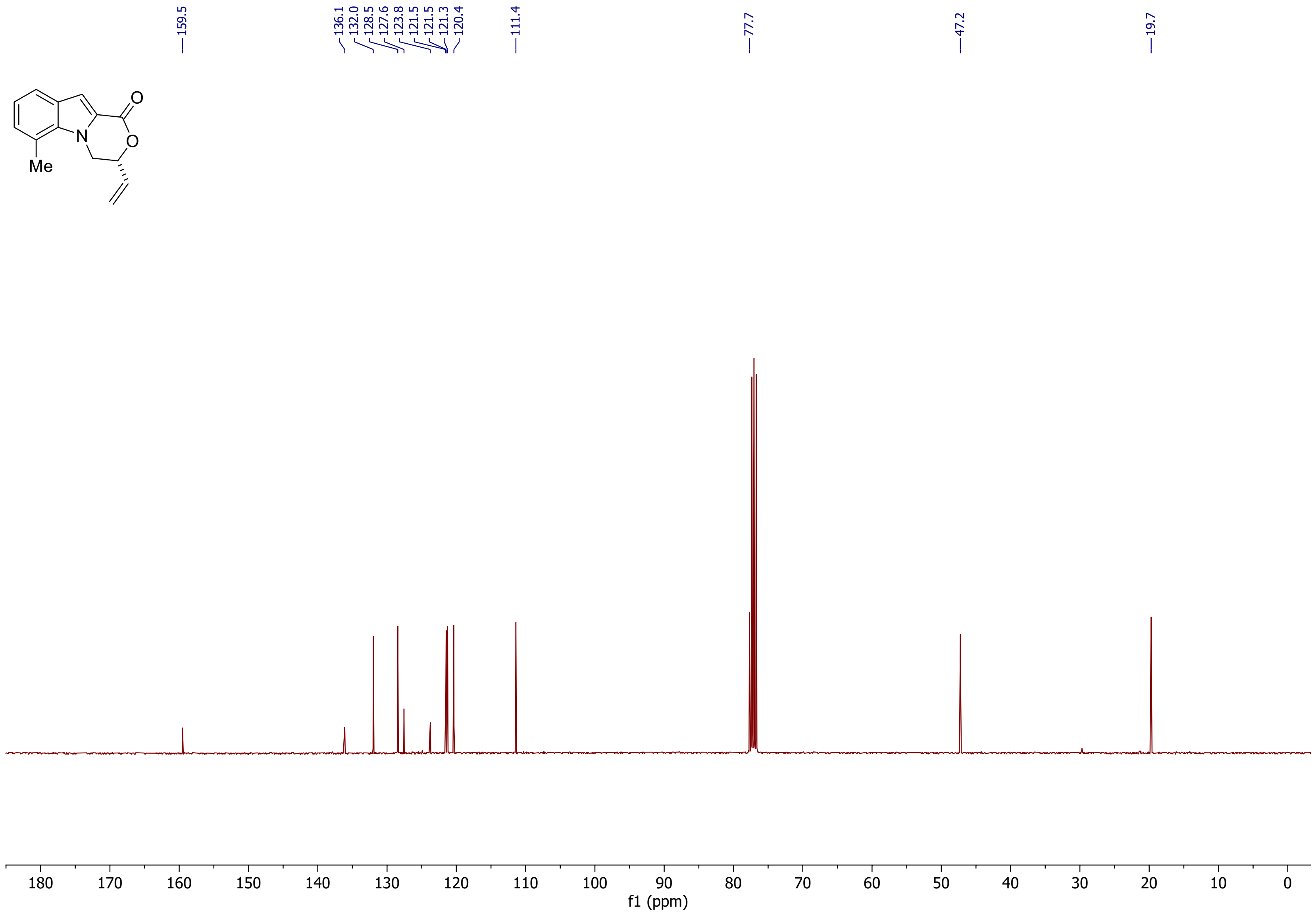

Figure S151. 2D ^{1}H-^{1}H COSY spectrum (298 K, $CDCl_3$) of (*R*)-**8e**

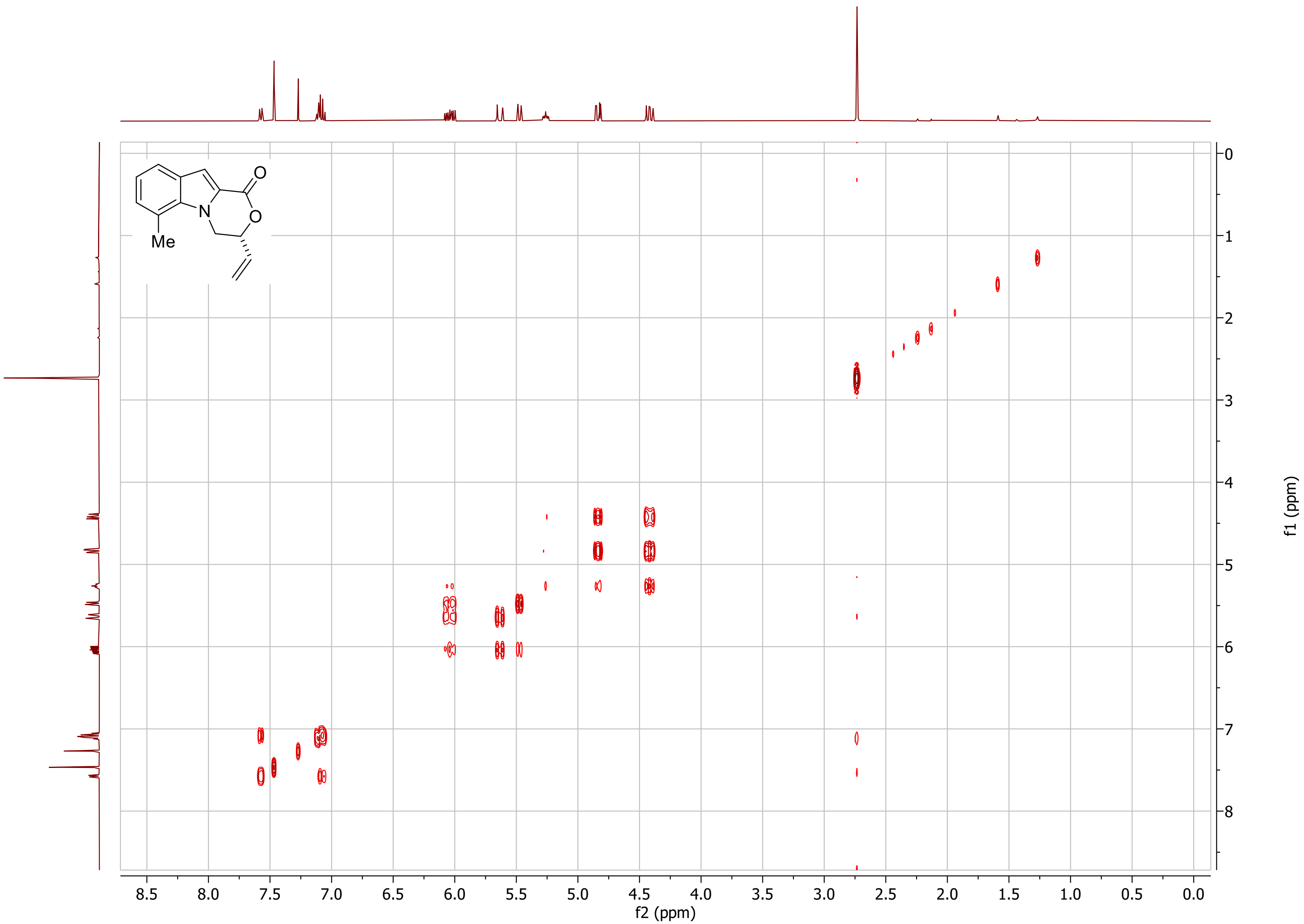

Figure S152. 2D ^1H-^{13}C HSQC spectrum (298 K, $CDCl_3$) of (*R*)-**8e**

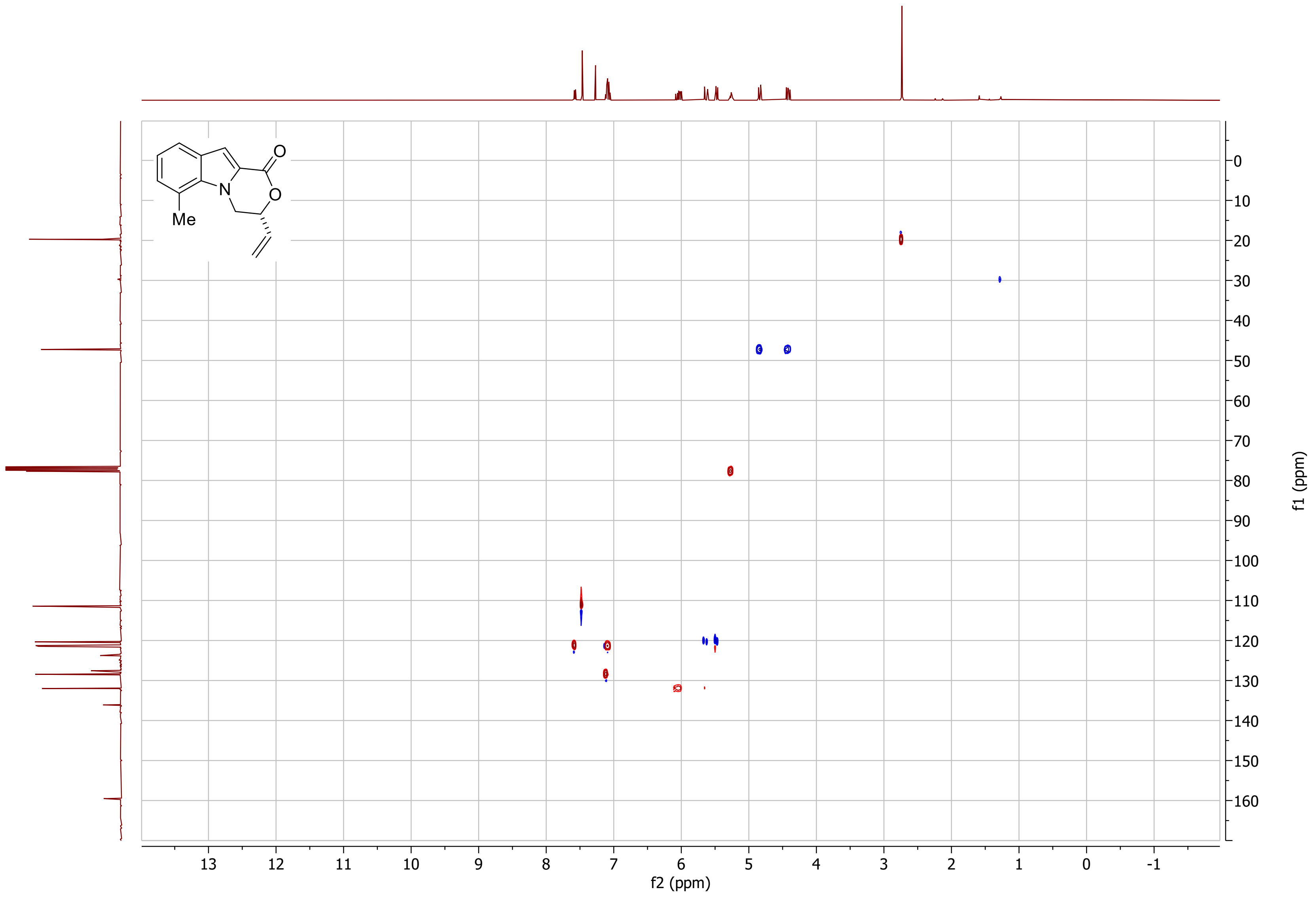

Supplement: Supplementary file 1 [file au5c00885_si_001.pdf]
